# Bioinspired desaturation of alcohols enabled by photoredox proton-coupled electron transfer and cobalt dual catalysis

**DOI:** 10.1038/s41467-022-28441-2

**Published:** 2022-02-10

**Authors:** Long Huang, Tengfei Ji, Chen Zhu, Huifeng Yue, Nursaya Zhumabay, Magnus Rueping

**Affiliations:** 1 Institute of Organic Chemistry, RWTH Aachen University, Landoltweg 1, 52074 Aachen, Germany; 2 Kaust Catalysis Center (KCC), King Abdullah University of Science and Technology (KAUST), Thuwal, 23955-6900 Saudi Arabia

**Keywords:** Synthetic chemistry methodology, Homogeneous catalysis

## Abstract

In the biosynthesis sterols an enzyme-catalyzed demethylation is achieved via a stepwise oxidative transformation of alcohols to olefins. The overall demethylation proceeds through two sequential monooxygenation reactions and a subsequent dehydroformylative saturation. To mimic the desaturation processes observed in nature, we have successfully integrated photoredox proton-coupled electron transfer (PCET) and cobaloxime chemistry for the acceptorless dehydrogenation of alcohols. The state-of-the-art remote and precise desaturation of ketones proceeds efficiently through the activation of cyclic alcohols using bond-dissociation free energy (BDFE) as thermodynamic driving force. The resulting transient alkoxyl radical allows C-C bond scission to generate the carbon-centered radical remote to the carbonyl moiety. This key intermediate is subsequently combined with cobaloxime photochemistry to furnish the alkene. Moreover, the mild protocol can be extended to desaturation of linear alcohols as well as aromatic hydrocarbons. Application to bioactive molecules and natural product derivatives is also presented.

## Abstract (web summary)

Dehydrogenative reactions can provide alkenes, which are among the most useful handles for synthetic organic chemists. Here the authors integrated photoredox proton-coupled electron transfer and cobaloxime chemistry for the acceptorless dehydrogenation of alkyl alcohols.

## Introduction

**Fig. 1:**
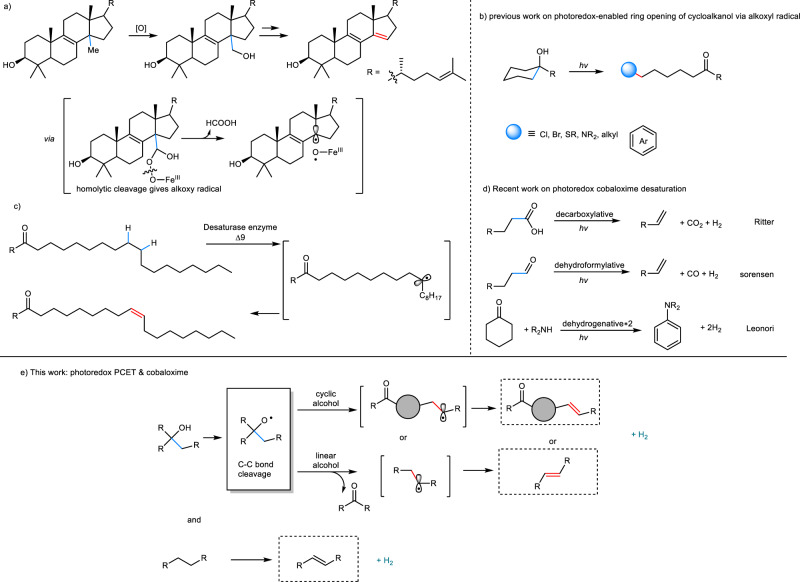
Our inspiration from nature and previous work. **a** 14α-Demethylase (**CYP51**)-catalyzed demethylation of lanosterol and possible reaction mechanism. The **CYP51** belongs to the family of cytochrome P450 enzymes. The demethylation proceeds through two sequential monooxygenation reactions and subsequently a dehydroformylation. **b** Ring-opening and functionalization of cycloalkanols enabled by photoredox catalysis. **c** Possible mechanism for stearoyl Δ9 desaturation. **d** Recent notable work on dehydrogenation via photoredox cobaloxime dual catalysis. **e** Desaturation of alcohols and aliphatic C–H bond via PCET/cobaloxime dual catalysis.

In the biosynthesis of sterols, a central reaction is the enzyme-catalyzed demethylation that involves a stepwise oxidative transformation of alcohols to olefins^1–4^. To trigger the C–C bond cleavage, the generation of an alkoxyl radical is believed to be crucial (Fig. 1a). Inspired by this intriguing enzymatic process, we sought an analogous methodology for desaturation of alcohols in organic synthesis. The access of alkoxyl radicals *via* hydrogen atom transfer from the hydroxyl groups of aliphatic alcohols is straightforward but remarkably challenging. This is mainly attributed to the intrinsic difficulty of activating the kinetically inert O–H bonds^5^. In recent years, visible light photoredox catalysis has emerged as a powerful technique in organic synthesis that relies upon energetic electron transfer processes to facilitate previously thermally inaccessible or kinetically inert transformations^6–10^. In this context, the activation of O–H bonds has found broad utility in a number of reactions for the construction of C–C ^11–16^, C–N ^17–19^, C–S ^20^, and C–X ^21,22^ bonds (Fig. 1b). Despite these efforts, it is surprising to consider that the bioinspired olefin synthesis through acceptorless desaturation of alcohols remains an unmet challenge, to the best of our knowledge^23^.

Unsaturated carbonyl compounds are not only versatile synthetic building blocks, but also ubiquitous in natural products and biologically relevant molecules^24–29^. Although protocols for carbonyl desaturation at the adjacent sites (α-/β-) have been widely established, a mild and general strategy for remote site desaturation of ketone would be very appealing, but has not been reported. In nature, desaturase enzyme fascinates us with the regio- and stereoselective olefinic bond formation during the biosynthesis of fatty acid (Fig. 1c)^30^. Notably, Baran^31^ and Gevorgyan^32,33^ recently reported auxiliaries assisted remote desaturation of alcohols and amines, through TEMPO mediated process or palladium photoredox catalysis, respectively. Given state-of-the-art remote functionalization, we considered that it is highly desirable to exploit readily accessible reaction partners with efficient catalyst systems to produce unsaturated ketones that are difficult to synthesize via conventional pathways.

Recently, our group established a dual Nickel photoredox catalysis reaction to enable the remote cross coupling of tertiary alcohols^16^. This catalytic manifold provides a general and facile access to carbon-centered radicals remote to the carbonyl moiety via multiple site proton-coupled electron transfer (PCET)^34,35^. In line with the insight gained from this dual catalytic system, as well as recent developments on the dehydrogenative functionalization^36–41^, we envisioned the alkyl radical generated in this manner could in principle be merged with metallaphotoredox to realize the bioinspired acceptorless desaturation of alcohols. Enlighted by the most recent development on cobaloxime-based photoredox catalysis by Ritter, Sorensen and Leonori groups (Fig. 1d)^42–55^, we became interested in the combination of photoredox PCET with the proton reduction reactivity of cobaloximes (Fig. 1e). Here, we describe the development of a bioinspired acceptorless remote desaturation of tertiary, as well as secondary alcohols, via the photoredox PCET and cobalt synergistic catalysis and the extension to the desaturation to aromatic hydrocarbons, as well as silyl enol ethers.

## Results

### Rational design

**Fig. 2:**
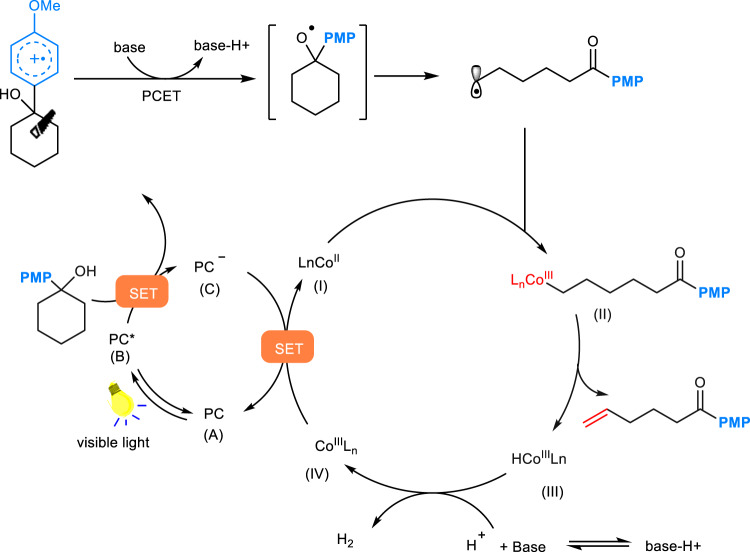
Our envisioned mechanism proposal. Photoredox PCET/cobaloxime dual catalysis enabled desaturation of alcohols. PMP *p*-methoxy phenyl group.

Our mechanistic proposal is shown in Fig. 2. Upon visible light irradiation, a single electron transfer from the cyclohexanol derivative (*E*_p/2_ = 1.57 V vs. SCE)^16^ to the highly oxidizing singlet excited state *Mes–**Acr**–**Me**^+^ (*E*_1/2_^red^ = +2.06 V vs. SCE)^6^ would generate the corresponding arene radical cation along with the reduced form of the photocatalyst **Mes**–**Acr**–**Me**•. Subsequent multiple site PCET reaction between the hydroxyl group and the radical cation in the presence of base would give the key alkoxyl radical species, which readily cleaves into a carbonyl moiety and a distal carbon-centered radical through β scission of the neighboring C–C bond. The C-centered radical would subsequently be intercepted by the Co^II^ species (**I**) to yield an alkyl–Co^III^ intermediate (**II**), which can undergo C-cobalt bond homolysis upon light irradiation. Next, a, β-hydrogen abstraction by Co^II^ at this stage would deliver the desired olefin and a cobalt^III^ hydride species (**III**). Hydrogen gas evolves through the interaction between (**III**) and a proton generated in the PCET step. The cobalt and photoredox catalytic cycles then simultaneously complete via a single electron transfer event between Co^III^ intermediate (**IV**) (*E*_1/2_^red^ = –0.68 V vs. SCE) and reduced form of photocatalyst **C**^56,57^.

### Optimization of the reaction conditions

The reaction conditions optimization of the synergistic combination of photoredox and cobalt catalysis is briefly summarized in Table 1. The initial evaluation focused on readily available substrate **1a** to mimic the enzymatic process, namely Δ9 desaturation of stearoyl-CoA (Fig. 1c). Optimized reaction conditions were readily established, using 7.5 mol% **PC**-**I** Mes–Acr–Me^+^, 10 mol% Co(dmgH)_2_(py)_2_PF_6_ and 2 equiv. 2,4,6-collidine in a 0.1 M solution of 1,2-dichloroethane (DCE) at room temperature with blue light-emitting diodes (LEDs) irradiation. Under these conditions, the desired product was formed in 93% NMR yield and very good selectivity (22:1) (Table 1, entry 1). Use of less oxidizing photosensitizers such as [Ru(bpy)_3_](PF_6_)_2_ (**PC**-**II**, *E*_1/2_[Ru^*III^/Ru^II^ = + 0.77 V vs. SCE in CH_3_CN])^58^ and [Ir(dF(CF_3_)ppy)_2_(bpy)]PF_6_ (**PC**-**III**, *E*_1/2_[Ir^*III^/Ir^II^ = + 1.21 V vs. SCE in CH_3_CN])^59^ resulted in no product or trace amounts of product, while the strong oxidizing [Ir(dF(CF_3_)ppy)_2_(5,5’(CF_3_)bpy)]PF_6_ (**PC**-**IV**, *E*_1/2_[Ir^*III^/Ir^II^ = + 1.68 V vs. SCE in CH_3_CN]) gave **2a** in 71% yield (entries 2 to 4). A screening of different organic and inorganic bases revealed that collidine was the best choice (entries 5 and 6). The use of Co(dmgH)(dmgH_2_)Cl_2_ and Co(dmgH)_2_PyCl as cobaloxime sources leads to a decrease of yields and selectivities (entries 7 and 8). When the reaction was conducted in HFIP or MeCN, the amount of product could be negligible. The reaction efficiency diminished dramatically when toluene was employed, while a comparable result was observed using DCM (entries 9–12). The inverse relationship between solvent polarity and yield can be taken as support for the intermediacy of hydrogen bonding, because the polar interaction is generally disfavored in polar solvent. It is not surprising to observe that control experiments performed without photocatalyst, Co catalyst, base or light each failed to give any desired product (Table 1, entry 13).

**Table 1:** Optimization of the reaction conditions^a,b^.


Entry	Change from standard conditions	Yield [%] (2a/2a’)
1	No change	93 (22:1)
2^c^	**PC**-**II** as photocatalyst	0 (-)
3^c^	**PC**-**III** as photocatalyst	1 (-)
4^c^	**PC**-**IV** as photocatalyst	71 (25:1)
5	K_3_PO_4_ as base	2 (-)
6	2,6-lutidine as base	86 (20:1)
7	Co(dmgH)(dmgH_2_)Cl_2_ as cobalt source	60 (6:1)
8	Co(dmgH)_2_PyCl as cobalt source	88 (24:1)
9	HFIP as solvent	0 (-)
10	MeCN as solvent	6 (-)
11	Toluene as solvent	42 (19:1)
12	DCM as solvent	92 (19:1)
13	No light or [Co] or collidine or **PC**-**I**	0 (-)

^a^Reaction conditions: **1a** (0.1 mmol, 1 equiv.), 2,4,6-collidine (0.2 mmol, 2 equiv.), **PC**-**I** (7.5 mol%), Co(dmgH)_2_(py) _2_PF_6_ (10 mol%), DCE (1.0 mL, 0.1 M), rt, blue LEDs, 36 h.

^b^Yields and ratios were determined by 1H NMR of the crude mixture using 1,3,5-trimethoxybenzene as an internal standard.

^c^1 mol% photocatalyst was used.

### Substrate scope

**Fig. 3:**
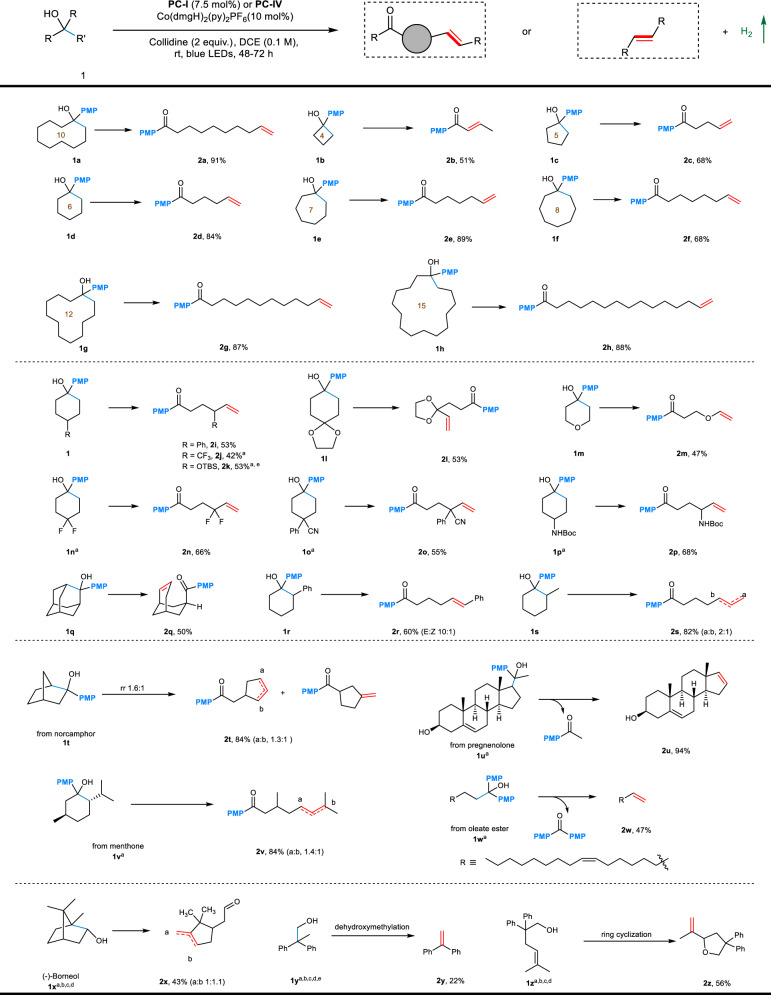
Scope of cyclic and linear alcohols. General reaction conditions: **1** (1 equiv.), 2,4,6-collidine (2 equiv.), **PC**-**I** (7.5 mol%), Co(dmgH)_2_(py)_2_PF_6_ (10 mol%), DCE (0.1 M), rt, blue LEDs, 48–72 h. **a** 1 or 3 mol% [Ir(dF(CF_3_)ppy)_2_(5,5′d(CF_3_)bpy)](PF_6_) **PC-IV** was used. **b** NBu_4_OP(O)(OPh)_2_ as base. **c** Toluene or PhCF_3_ as solvent. **d** 5 mol% Co catalyst was used in **2u** to **2z**. **e** ^1^H NMR yield with internal standard.

As illustrated in Fig. 3, we found the desaturation protocol was tolerant to a wide range of alcohols and gave the corresponding olefinic products in moderate to very good yields. To begin with, it was found that cyclic tertiary alcohols with different ring sizes (**1a**–**1h**) all reacted smoothly to selectively generate remote desaturated ketones regardless of their ring strains (**2a**–**h**, 51–91% yields). Notably, due to the base mediated isomerization, the α-/β-sites desaturation product was obtained exclusively with prolonged reaction time in the reaction of cyclobutanol **1b**. Symmetrical substituted cyclohexanols (**1i** to **1p**) behaved well to give products **2i** to **2p** in moderate yields (42–68%). Functional groups including trifluoromethyl, silyl ether, geminal difluoride, nitrile and amide could be well tolerated. The ring-opening of unsymmetrical cyclohexanols **1r** and **1s** took place regioselectively and, the remarkable selectivity can be explained by C–C bond cleavage favors the formation of more stabilized carbon-centered radical (**2r** and **2s**, 60% and 82% yields).

For the norcamphor-derived bridged bicyclic substrate **1t**, mixture of isomers was observed as a result of poor selectivity in the ring-opening. In contrast, a menthone derivative displays excellent regioselectivity in the ring-opening step (**1v**, 84%). Moreover, we successfully extended the scope to linear tertiary alcohols derived from pregnenolone and oleate ester, affording the corresponding olefins **2u** and **2w** in moderate to excellent yields. In addition to tertiary alcohols, naturally occurring secondary alcohol **1x** proved to be competent substrate for the PCET enabled regioselective ring-opening/desaturation sequence. Remarkably, the dehydroxymethylative desaturation of 2,2-diphenylpropan-1-ol could take place, affording the diphenylethylene in 22% yield. Next, we found that the cascade ring cyclization/desaturation was feasible, a moderate yield of cyclized product was obtained (**2z**, 56%).

**Fig. 4:**
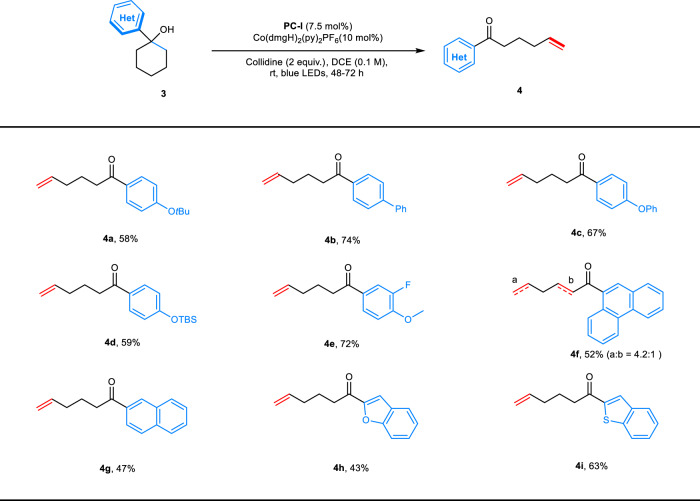
Scope of arene substituents. Reaction conditions: **3** (0.2 mmol, 1 equiv.), 2,4,6-collidine (0.4 mmol, 2 equiv.), **PC**-**I** (7.5 mol%), Co(dmgH)_2_(py)_2_PF_6_ (10 mol%), DCE (2.0 mL, 0.1 M), rt, blue LEDs, 48–72 h.

With the above success, we next examined the generality of the photoredox-cobalt desaturation with respect to the arene substituent on the cyclic ring. As shown in Fig. 4, a variety of arene-substituted cyclohexanols performed well. Monosubstituted aromatics such as *tert*-butyl–, phenyl–, and *tert*-butyldimethylsilyl (TBS)-protected phenols as well as biphenyl are suitable candidates (**4a**–**4d**, 58–74% yields). Disubstituted anisole derivative was also suitable substrate for this transformation (**4e**, 72% yield). Both naphthalene- and phenanthrene-substituted cyclohexanols smoothly underwent ring-opening/desaturation under these conditions (**4f** and **4g**, 52% and 47% yields). Interestingly, minor amount of α-/β-sites desaturation product was obtained in the case of **4f**, this can be accounted for by Co^III^-hydride mediated chain walking process^51^. Following the success, privileged heteroaromatics including benzofuran and thiobenzofuran could also be used (**4h** and **4i**, 43% and 63% yields).

**Fig. 5:**
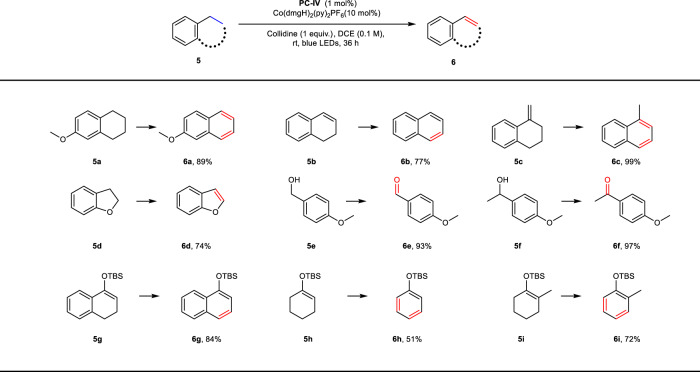
Scope of aromatic hydrocarbons and silyl enol ether. Reaction conditions: **5** (0.2 mmol, 1 equiv.), 2,4,6-collidine (0.2 mmol, 1 equiv.), [Ir(dF(CF_3_)ppy)_2_(5,5′d(CF_3_)bpy)](PF_6_) **PC-IV**(1 mol%), Co(dmgH)_2_(py)_2_PF_6_ (10 mol%), DCE (1.0 mL, 0.2 M), rt, blue LEDs, 36 h.

To further highlight the robustness of this protocol, tetrahydronaphthalene **5a** was subjected to the reaction conditions (Fig. 5). The desaturation takes place selectively to release 2 molar equiv. hydrogen gas and no dihydronaphthalene product was observed, indicating the second desaturation should be easier than the initial one. Therefore, two styrene derivatives were examined next, to give products such as **6b** and **6c** in good to excellent yields. Heterocycle such as **6d** can also be prepared in good yield (74%). Moreover, when benzylic alcohols were employed in the PCET enabled acceptorless desaturation, the corresponding aldehyde and ketone products were obtained in excellent yields (**6e** and **6f**). Importantly, this protocol was able to transform silyl enol ethers into silyloxyarenes in moderate to good yields^60^. For example, desaturation of **5g** leads to the formation of *tert*-butyl(dimethyl)silyl (TBS) ether of α-naphthol with excellent efficiency (**6g**, 84% yield). Silyl enol ethers derived from cyclohexanones were also amenable to the current protocol via the removal of two molecules of hydrogen gas, with no detection of α, β-desaturated ketone product (**6h** and **6i**, 51% and 72% yields, respectively). This intriguing selectivity stands in stark contrast to previous dehydrogenation reactions that exclusively affording cyclohexenones^61,62^.

**Fig. 6:**
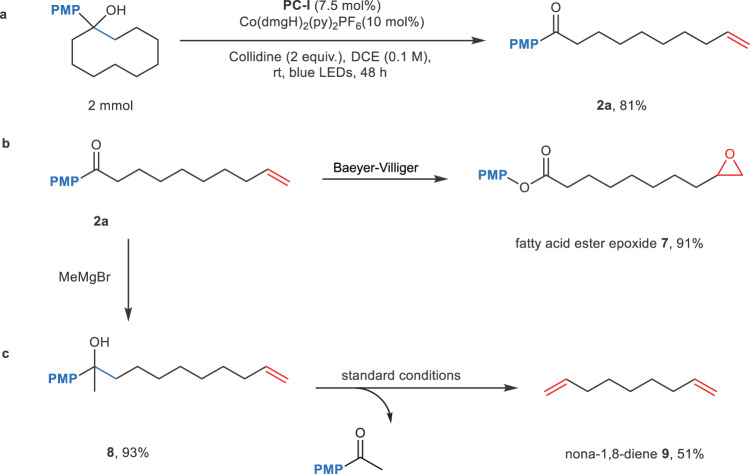
Scale up and synthetic utility. **a** Reaction on large scale, under standard conditions: tertiary alcohol (1 equiv.), 2,4,6-collidine (2 equiv.), **PCI** (7.5 mol%), Co(dmgH)_2_(py) _2_PF_6_ (10 mol%), DCE (0.1 M), rt, blue LEDs, 48 h. **b** Baeyer-villiger oxidation. **c** sequential desaturation to synthesize diene.

Next, we carried out the synthesis of remote unsaturated ketone **2a** on a preparative scale (2 mmol) under our optimal conditions, providing the expected olefinic product **1a** in 81% yield (Fig. 6a). To showcase the synthetic utility of the product provided by this methodology, **2a** was converted efficiently to a fatty acid ester epoxide during a Baeyer-Villiger oxidation (Fig. 6b). Following a Grignard reaction of **2a**, the linear product **8** was subjected further to the reaction conditions, an interesting diene **9** was isolated in 51% yield (Fig. 6c).

**Fig. 7:**
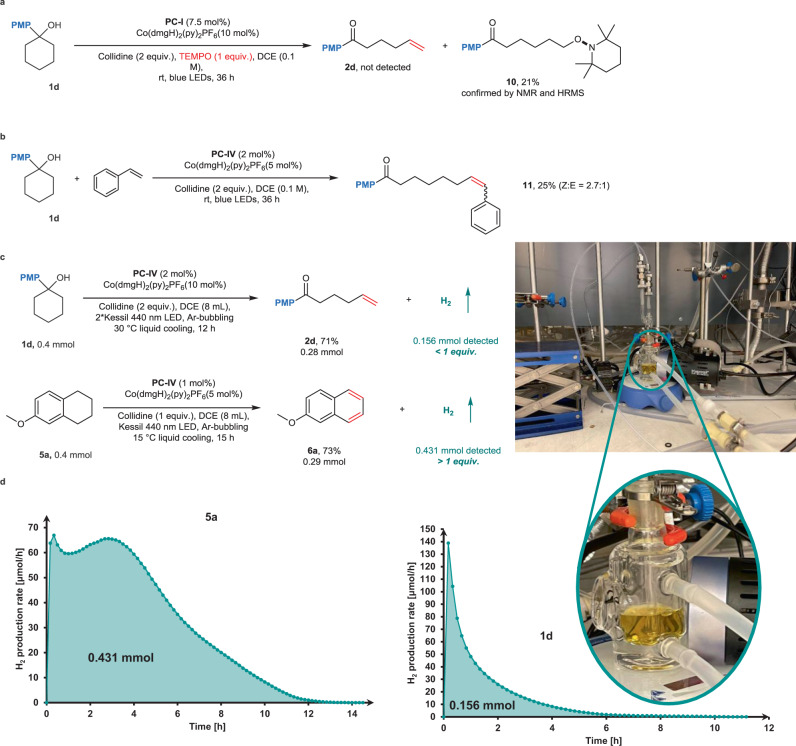
Preliminary experiments on the reaction mechanism. **a** Radical-trapping experiment with free-radical scavenger. **b** Heck-Type coupling with styrene. **c** Reaction conditions and results for H_2_ production of substrates **1d** and **5a**. **d** H_2_ production rate monitoring and quantification for substrates **1d** and **5a**.

Apart from the control experiments shown in Table 1, we conducted some preliminary mechanistic experiments to gain some insight of the metallaphotoredox desaturation protocol. When the reaction mixture was subjected to a radical scavenger 2,2,6,6-Tetramethyl-1-piperidinyloxy (TEMPO, 1 equiv.) under the standard conditions, no product was detected. A remote TEMPO-trapped ketone **10** was instead formed, implying that a radical process is involved in the catalytic cycle (Fig. 7a). The generation of carbon-centered radical remote to the carbonyl group was further supported by the Heck-Type coupling with styrene, affording **11** (Fig. 7b). To gain more insight into the reaction, the formation of molecular hydrogen was quantitatively analyzed by gas chromatography. Importantly, we observed that more than 1 equiv. hydrogen gas was produced with substrate **5a**, in contrast the generation of H_2_ is less than 1 equiv. in the case of **1d**. The kinetic profile of H_2_ evolution of substrate **1d** shows a fast gas production rate in the first hours, however, it becomes very sluggish after 6 h (Fig. 7c, d). This result is consistent with our observation that the desaturation of alcohols generally required long reaction time, we assume that the increasing amount of free base has a marked effect on the hydrogen production^63^.

## Discussion

In conclusion, we have developed a bioinspired acceptorless desaturation of tertiary as well as secondary alcohols *via* the photoredox PCET and cobalt synergistic catalysis. The manifold provides a concise access to remotely dehydrogenated ketones that are difficult to synthesize with current methods, through ring-opening/desaturation of cyclic alcohols. We also demonstrated the strategy could be applied to linear alcohol, aromatic hydrocarbons as well as silyl enol ethers. Importantly, a variety of bioactive molecules and natural product derivatives were all well tolerated under such mild conditions. In consideration with numerous findings about the essential role of PCET in biological redox processes, this contribution expands the less-developed applications of PCET in organic synthesis.

## Methods

### General procedure for bioinspired dehydrogenation of alcohols

To a 15 mL vial equipped with a stir bar was added Co(dmgH)_2_(py)_2_PF_6_ (12 mg, 0.02 mmol, 10 mol%), and photocatalyst (9-mesityl-10-methylacridinium perchlorate 7.5 mol% or [Ir(dF(CF_3_)ppy)_2_(5,5′d(CF_3_)bpy)](PF_6_) 1 mol%), collidine (1 or 2 equiv.) and tertiary alcohol (0.2 mmol, 1 equiv.). The vial was sealed, evacuated and backfilled with Argon three times, then 2 mL of DCE was added. After degassing with Freeze–Pump–Thaw methods for three cycles, it was stirred and irradiated with the corresponding blue LEDs photoreactor. Upon completion, the reaction mixture was concentrated in vacuo and purified with column chromatography to afford the desired product.

## Supplementary information

Supplementary Information

## Data Availability

The authors declare that all data generated in this study are available within the article and the Supplementary Information.
