# Supplementary material for: Bioinspired desaturation of alcohols enabled by photoredox proton-coupled electron transfer and cobalt dual catalysis
Source: Nat Commun. 2022 Feb 10;13:809. doi: 10.1038/s41467-022-28441-2 (PMC8831637; doi:10.1038/s41467-022-28441-2)
Supplement: Supplementary file 1 — Supplementary Information [file 41467_2022_28441_MOESM1_ESM.pdf]

Supplemental Information for

Bioinspired Desaturation of Alcohols Enabled by Photoredox Proton-coupled Electron Transfer and Cobalt Dual Catalysis

Long Huang*[1], Tengfei Ji[2], Chen Zhu[2], Huifeng Yue[2], Nursaya Zhumabay[2] and Magnus Rueping*[2]

[1]RWTH Aachen University, Institute of Organic Chemistry, Landoltweg 1, D-52074 Aachen, Germany

[2]King Abdullah University of Science and Technology (KAUST), KAUST Catalysis Center (KCC), Thuwal, 23955-6900 (Saudi Arabia)

[*]Corresponding Authors

long.huang@rwth-aachen.de; magnus.rueping@kaust.edu.sa

Table of Contents

Supplementary Methods

**1.**	**General Information**	**S2**
**2.**	**Preparation and Characterization of Tertiary Alcohols**	**S3**
**3.**	**Cyclic Voltammetry Data**	**S8**
**4.**	**General Procedure for the Cross Coupling Reaction and Reaction Setup**	**S9**
**5.**	**Quantification of Photocatalytic Hydrogen Gas Production**	**S11**
**6.**	**Characterization of the Products**	**S13**
**7.**	**NMR Spectra**	**S35**

Supplementary References **S100**

Supplementary Methods

1. General Information

Unless otherwise noted, all commercially available compounds were used as provided without further purification. Solvents for chromatography were technical grade and distilled prior to use. Dry toluene, 1, 2-dichloroethane (DCE), chloroform, chlorobenzene used in reactions were obtained by distilling over calcium hydride and were stored over activated molecular sieves (4 Å). Dry isopropyl ether ($^{i}Pr_2O$), diethyl ether (Et_2O), tetrahydrofuran (THF) and toluene used in reactions were obtained by distilling over sodium-benzophenone ketyl. Analytical thin-layer chromatography (TLC) was performed on Macherey-Nagel silica gel 60 aluminium plates with F-254 indicator, visualised by UV irradiation. Column chromatography was performed using MN silica gel (particle size 0.040-0.063 mm). ^{1}H-NMR and ^{13}C-NMR spectra were recorded on a vnmrs-400 or vnmrs-600 spectrometer in $CDCl_3$ or C_6D_6 with residual proton signal of the deuterated solvents as the internal reference (δH = 7.26 ppm and δC = 77.16 ppm for $CDCl_3$, δH = 7.16 ppm and δC = 128.06 ppm for C_6D_6). Data are reported in the following order: chemical shift (δ) in ppm; multiplicities of ^{1}H NMR are indicated s (singlet), d (doublet), t (triplet), q (quartet), m (multiplet), dd (doublet of doublet), tt (triplet of triplet), dt (doublet of triplet), td (triplet of doublet); coupling constants (*J*) are in Hertz (Hz). All ^{13}C NMR spectra were measured with ^{1}H decoupling. Carbon assignment was determined by APT spectra: s stands for quarternary carbon, d stands for CH-group, t stands for CH_2-group, q stands for CH_3-group. IR spectra were recorded on a Jasco FT/IR-420 spectrometer and are reported in terms of frequency of absorption (cm^{-1}). Mass spectra were acquired on a Finnigan SSQ7000 (EI/CI) spectrometer and high resolution mass spectra on a Finnigan MAT 95 (EI/CI) or on a ThermoFisher Scientific LTQOrbitrap XL (ESI). The Blue LED strips 24 V (19.2 W/m, with emission maximum at λ_{max} = 467 nm) were purchased from Ledxon GmbH (Germany).

2. General Procedure for the Synthesis of Tertiary Alcohols

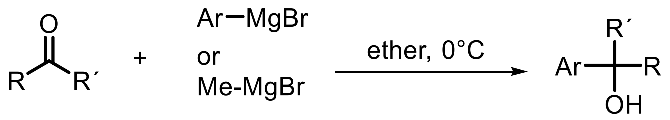

General procedure A with Grignard reaction: A flame-dried round-bottom flask equipped with a stir bar under argon was charged with 4-methoxyphenylmagnesium bromide solution (1.5 equiv., 1.0 M in 2-MeTHF) in ether. Then the reaction mixture was cooled at 0 °C with an ice bath and the ketone (1.0 equiv.) was added dropwise. The reaction mixture was stirred for 0.5-1.5 h at the same temperature and monitored by TLC until the complete consumption of ketone, followed by quenching with ice water. After drying over anhydrous Na_2SO_4 it was filtered and concentrated in vacuo. The crude product was purified by neutral alumina flash column chromatography to afford the corresponding product.

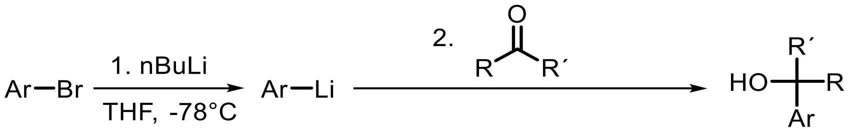

General procedure B with lithiation reaction: *n*-Butyllithium (1.6 M solution in hexane, 1.1 equiv.) was added slowly to a stirred solution of arylbromide (1.0 equiv.) in THF at –78 °C. The mixture was stirred for 1–2 h at the same temperature and then the corresponding ketone (1 equiv.) was added dropwise *via* syringe. The mixture was stirred for another 1–1.5 h, followed by quenching with ice water. After drying over anhydrous Na_2SO_4, it was filtered and concentrated in vacuo. The crude product was purified by neutral alumina flash column chromatography to obtain the pure alcohol.

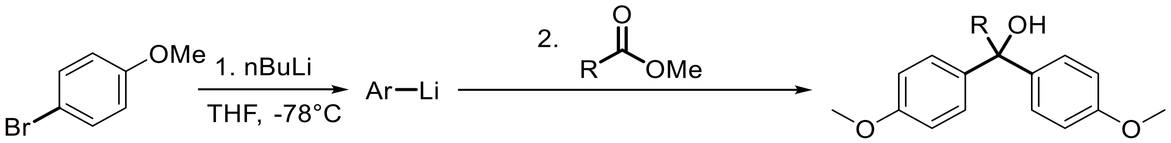

General procedure C with lithiation reaction: *n*-Butyllithium (1.6 M solution in hexane, 4 equiv.) was added slowly to a stirred solution of arylbromide (4.0 equiv.) in THF at –78 °C. The mixture was stirred for 1–2 h at the same temperature and then the corresponding ester (1 equiv.) was added dropwise *via* syringe. The mixture was stirred and slowly warmed to room temperature overnight, followed by quenching with ice water. After drying over anhydrous Na_2SO_4, it was filtered and concentrated in vacuo. The crude product was purified by neutral alumina flash column chromatography to obtain the pure alcohol.

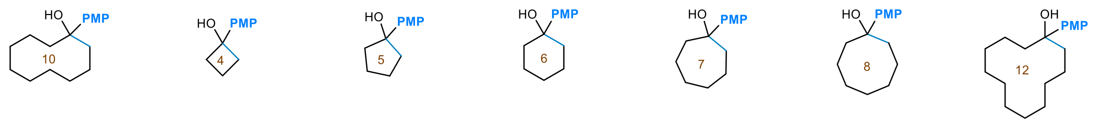

1a, *CAS RN* 2032262-47-6 **1b**, *CAS RN* 164171-80-6 **1c**, *CAS RN* 78195-96-7 **1d**, *CAS RN* 17138-79-3 **1e**, *CAS RN* 77826-30-3 **1f**, *CAS RN* 1392008-96-6 **1g**, *CAS RN* 32960-61-5

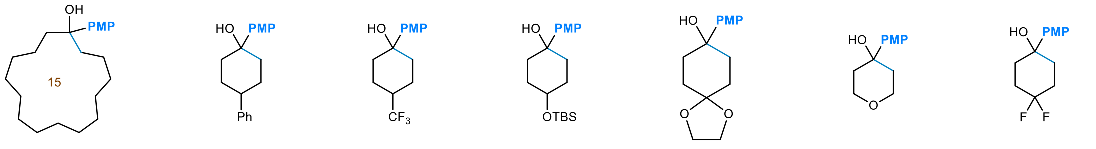

1h, *CAS RN* 2410095-74-6 **1i**, *CAS RN* 1183077-22-6 **1j**, unknown **1k**, unknown **1l**, *CAS RN* 67019-51-6 **1m**, *CAS RN* 1023937-37-2 **1n**, *CAS RN* 2259643-09-7

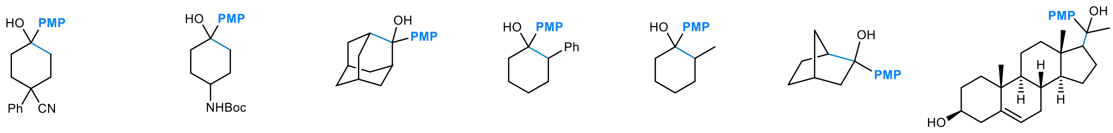

1o, *CAS RN* 1092531-68-4 **1p**, unknown **1q**, *CAS RN* 76481-43-1 **1r**, unknown **1s**, *CAS RN* 97704-25-1 **1t**, *CAS RN* 78195-90-1 from norcamphor **1u**, from pregnenolone

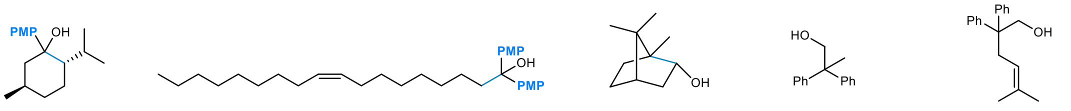

1v, *CAS RN* 2047808-65-9 from menthone **1w**, unknown from oleate ester **1x**, *CAS RN* 464-45-9 (-)-Borneol **1y**, *CAS RN* 74421-26-4 **1z**, *CAS RN* 752206-82-9

Supplementary Figure 1. Substrates of Fig. 3. All substrates were synthesized according to the above general procedures, (**1a-g**, **1u** and **1v**)[1] (**1h-i**, **1n**, **1s** and **1t**),[2] **1l**,[3] **1m**[4], **1q**[5] and **1z**[6] were known in the literatures.

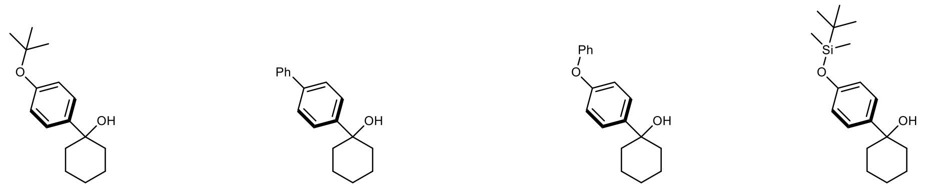

3a, *CAS RN* 2410095-76-8 **3b**, *CAS RN* 10468-72-1 **3c**, *CAS RN* 2410095-77-9 **3d**, *CAS RN* 2410095-78-0

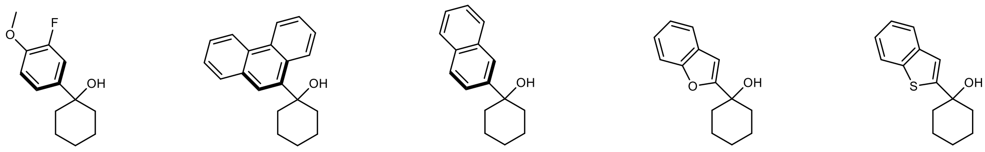

3e, *CAS RN* 1344022-16-7 **3f**, *CAS RN* 10468-81-2 **3g**, *CAS RN* 1481847-92-0 **3h**, *CAS RN* 1488411-94-4 **3i**, *CAS RN* 6774-43-2

Supplementary Figure 2. Substrates of Fig. 4. All substrates were synthesized according to the above general procedures, (**3a**, **3b**, **3e** to **3f**, **3h**),[2] **3c**,[7] **3g**,[8] and **3i**[9] were known in the literatures.

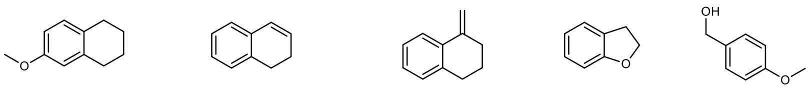

5a, *CAS RN* 1730-48-9 commercially available | **5b**, *CAS RN* 447-53-0 commercially available | **5c**, *CAS RN* 25108-63-8 | **5d**, *CAS RN* 496-16-2 commercially available | **5e**, *CAS RN* 105-13-5 commercially available

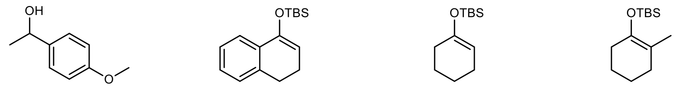

5f, *CAS RN* 3319-15-1 commercially available | **5g**, *CAS RN* 133464-90-1 | **5h**, *CAS RN* 62791-22-4 | **5i**, *CAS RN* 20152-33-4

Supplementary Figure 3. Substrates of Fig. 5. 5c,[10] 5g,[11] 5h[12] and **5i[13]** were all synthesized according to published procedures.

1-(4-methoxyphenyl)-4-(trifluoromethyl)cyclohexan-1-ol, **1j**: the title compound was

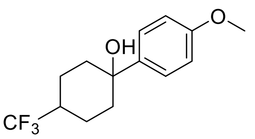

prepared following the general procedure B for the synthesis of tertiary alcohols. The crude product was purified by flash column chromatography to afford **1j** as a white solid in 77% yield (mixture of diastereomers, 1.5:1 ratio). Characterization is given for the major diastereomer.

^{1}H NMR (600 MHz, C_6D_6): δ 7.19 (d, J = 8.8 Hz, 2H), 6.76 (d, J = 8.8 Hz, 2H), 3.33 (s, 3H), 2.10 (t, J = 9.0 Hz, 2H), 1.86 – 1.73 (m, 1H), 1.69 (t, J = 12.0 Hz, 2H), 1.47 – 1.35 (m, 4H), 1.09 (s, 1H).

^{13}C NMR (151 MHz, C_6D_6): δ 159.3, 138.0, 128.8 (q, $J_{C\text{-}F}$ = 279.7 Hz), 127.1, 114.1, 71.8, 54.8, 39.6 (q, $J_{C\text{-}F}$ = 26.3 Hz), 36.1, 22.0.

^{19}F NMR (565 MHz, C_6D_6): δ -70.9.

IR (ATR) ν 3470, 3367, 2951, 1720, 1608, 1510, 1461, 1340, 1248, 1140, 1034, 951, 827 cm^{-1};

HRMS (ESI) for $C_{14}H_{17}F_3O_2Na^+$ $(M+Na)^+$: 297.1073; Found : 297.1069.

4-((tert-butyldimethylsilyl)oxy)-1-(4-methoxyphenyl)cyclohexan-1-ol, **1k**: the title

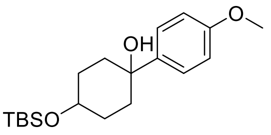

compound was prepared following the general procedure B for the synthesis of tertiary alcohols. The crude product was purified by flash column chromatography to afford **1k** as a colorless oil in 84% yield (mixture of diastereomers, 8:1 ratio).

^{1}H NMR (600 MHz, C_6D_6, mixture of diastereomers): δ 7.46 (d, J = 8.4 Hz, 0.24H_{minor}), 7.29 (d, J = 8.4 Hz, 2H_{major}), 6.84 – 6.80 (m, 2.24$H_{major+minor}$), 3.93 (brs, 0.12H_{minor}), 3.65 – 3.57 (m, 1H_{major}), 3.37 (s, 3H_{major}), 3.35 (s, 0.36H_{minor}), 2.41 – 2.33 (m, 0.24H_{minor}), 2.03 – 1.93 (m, 2.24$H_{major+minor}$), 1.74 – 1.54 (m, 6.48$H_{major+minor}$), 1.11 (s, 1H_{major}), 1.04 (s, 9H_{major}), 0.99 (s, 1.08H_{minor}), 0.13 (s, 6H_{major}), 0.05 (s, 0.72$H_{major+minor}$).

^{13}C NMR (151 MHz, C_6D_6, mixture of diastereomers): major isomer δ 159.0, 141.6, 126.2, 113.8, 71.5, 71.4, 54.9, 37.7, 32.1, 26.2, 18.4, -4.2.

IR (ATR) ν 3426, 2932, 2856, 1735, 1610, 1511, 1464, 1248, 1094, 1038, 964, 830, 773 cm^{-1};

HRMS (ESI) for $C_{19}H_{32}O_3SiNa^+$ $(M+Na)^+$: 359.2013; Found : 359.2006.

4-hydroxy-4-(4-methoxyphenyl)-1-phenylcyclohexane-1-carbonitrile, **1o**: the title

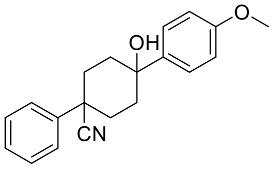

compound was prepared following the general procedure B for the synthesis of tertiary alcohols. The crude product was purified by flash column chromatography to afford **1o** as a colorless oil in 76% yield (containing small amount of ethyl acetate).

^{1}H NMR (400 MHz, C_6D_6): δ 7.25 – 7.17 (m, 4H), 7.08 – 6.96 (m, 3H), 6.77 (d, J = 8.4 Hz, 2H), 3.36 (s, 3H), 2.25 (s, 1H), 2.18 – 1.99 (m, 6H), 1.89 – 1.71 (m, 2H).

^{13}C NMR (101 MHz, C_6D_6): δ 159.3, 139.6, 137.3, 129.2, 127.3, 126.3, 123.5, 114.1, 71.5, 54.9, 42.0, 35.4, 33.8.

IR (ATR) ν 3457, 2950, 1608, 1511, 1450, 1299, 1249, 1181, 1032, 966, 831, 758 cm^{-1};

HRMS (ESI) for $C_{20}H_{21}NO_2Na^+$ $(M+Na)^+$: 330.1465; Found : 330.1462.

***tert*-butyl (4-hydroxy-4-(4-methoxyphenyl)cyclohexyl)carbamate**, **1p**: the title compound

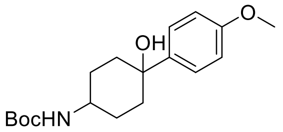

was prepared following the general procedure B for the synthesis of tertiary alcohols with arylbromide (1.8 equiv.) and n-BuLi (1.8 equiv.). The crude product was purified by flash column chromatography to afford **1p** as a white solid in 56% yield (mixture of diastereomers 2:1 ratio, containing small amount of inseparable impurity).

^{1}H NMR (600 MHz, C_6D_6): δ 7.30 – 7.26 (m, 2H), 6.80 (d, J = 8.2 Hz, 2H), 4.59 (brs, $0.33H_{minor}$), 4.48 (brs, $0.66H_{major}$), 3.87 (brs, $0.33H_{minor}$), 3.59 (brs, $0.66H_{major}$), 3.39 – 3.37 (m, 3H), 2.19 – 1.93 (m, 2H), 1.85 – 1.75 (m, 2H), 1.64 – 1.53 (m, 5H), 1.45 – 1.42 (m, 9H).

^{13}C NMR (151 MHz, C_6D_6): δ 158.6, 158.5, 155.0, 141.7, 126.4, 125.8, 113.5, 113.4, 78.3, 78.1, 71.5, 71.2, 54.5, 48.9, 37.8, 31.7, 28.8, 28.3, 28.2, 27.2.

IR (ATR) ν 3361, 3316, 2929, 1679, 1511, 1246, 1169, 1041, 961, 830 cm^{-1};

HRMS (ESI) for $C_{18}H_{27}NO_4Na^+$ $(M+Na)^+$: 344.1832; Found : 344.1824.

1-(4-methoxyphenyl)-2-phenylcyclohexan-1-ol, **1r**: the title compound was prepared

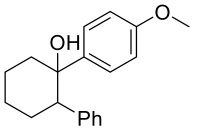

following the general procedure B for the synthesis of tertiary alcohols. The crude product was purified by flash column chromatography to afford **1r** as a single isomer in 31% yield.

^{1}H NMR (600 MHz, C_6D_6): δ 7.07 – 6.96 (m, 6H), 6.95 – 6.89 (m, 1H), 6.67 (d, J = 8.7 Hz, 2H), 3.24 (s, 3H), 2.80 (dd, J = 12.8, 3.6 Hz, 1H), 2.32 – 2.16 (m, 1H), 1.91 – 1.77 (m, 3H), 1.75 – 1.65 (m, 2H), 1.59 – 1.49 (m, 1H), 1.43 – 1.30 (m, 2H).

^{13}C NMR (101 MHz, C_6D_6): δ 158.4, 142.6, 140.6, 129.5, 127.9, 126.4, 126.3, 113.3, 75.5, 54.6, 53.6, 41.0, 29.2, 26.8, 22.3.

IR (ATR) ν 3533, 2929, 2845, 1607, 1511, 1146, 1353, 1291, 1245, 1181, 1036, 988, 831, 762 cm^{-1};

HRMS (ESI) for $C_{19}H_{22}O_2Na^+$ $(M+Na)^+$: 305.1512; Found : 305.1507.

(*Z*)-1,1-bis(4-methoxyphenyl)octadec-9-en-1-ol, **1w**: the title compound was prepared following the general procedure using C.

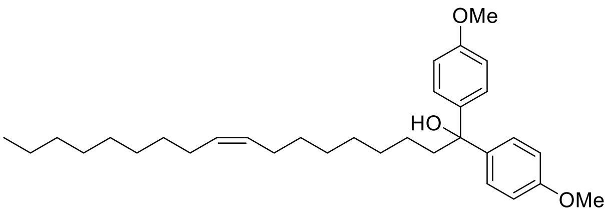

^{1}H NMR (600 MHz, C_6D_6): δ 7.34 (d, *J* = 9.1 Hz, 4H), 6.76 (d, *J* = 8.9 Hz, 4H), 5.50 – 5.41 (m, 2H), 3.27 (s, 6H), 2.15 – 2.10 (m, 2H), 2.09 – 2.01 (m, 4H), 1.54 (s, 1H), 1.38 – 1.18 (m, 22H), 0.86 (t, *J* = 6.8 Hz, 3H).

^{13}C NMR (151 MHz, C_6D_6): δ 158.5 (s), 140.2 (s), 129.8 (d, 2C),127.4 (d), 113.3 (d), 77.3 (s), 54.4 (q), 42.5 (t), 31.9 (t), 30.2 (t), 29.9 (t), 29.6 (t), 29.4 (t, 2C), 27.3 (t), 23.9 (t), 22.7 (t), 14.0 (q).

IR (ATR) ν 3497, 3002, 2924, 2853, 1608, 1584, 1372, 1299, 1246, 1175, 1036, 828, 723 cm^{-1};

HRMS (ESI) for $C_{32}H_{48}O_3Na^+$ $(M+Na)^+$: 503.3496; Found : 503.3494.

2-(4-methoxyphenyl)undec-10-en-2-ol, **8**: the title compound was prepared following the general procedure A for the synthesis of tertiary alcohols. Using 1-(4-methoxyphenyl)dodec-11-en-1-one **2a** (216 mg, 0.83 mmol, 1 equiv.) and methylmagnesium chloride (1.66 mmol, 2 equiv.).

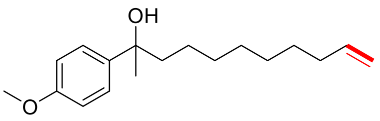

The crude product was purified by flash column chromatography to afford **8** as a colorless oi in 93% yield (214 mg).

^{1}H NMR (600 MHz, C_6D_6): δ 7.29 (d, *J* = 8.3 Hz, 2H), 6.80 (d, *J* = 8.4 Hz, 2H), 5.83 – 5.67 (m, 1H), 5.04 – 4.88 (m, 2H), 3.31 (s, 3H), 1.96 – 1.87 (m, 2H), 1.71 – 1.57 (m, 2H), 1.35 (s, 3H), 1.31 – 1.20 (m, 4H), 1.18 – 1.09 (m, 7H).

^{13}C NMR (151 MHz, C_6D_6): δ 158.4 (s), 140.5 (s), 138.9 (d), 126.0 (d), 114.1 (t), 113.3 (d), 73.7 (s), 54.4 (q), 44.5 (t), 33.8 (t), 30.3 (q), 30.0 (t), 29.4 (t), 29.1 (t), 28.9 (t), 24.0 (t).

IR (ATR) ν 3425, 3073, 2926, 2853, 2326, 2111, 1639, 1610, 1510, 1459, 1370, 1298, 1245, 1177, 1034, 996, 909, 831, 727 cm^{-1};

HRMS (ESI) for $C_{18}H_{28}O_2Na^+$ $(M+Na)^+$: 299.1982; Found : 299.1981.

3. Cyclic Voltammetry Data

Cyclic voltammetry was performed using a PGSTAT101 from Metrohm Autolab with a platinum working electrode, a Ag^{+} (0.01 M $AgNO_3$, 0.1 M NBu_4PF_6, MeCN)/Ag as reference electrode, a platinum wire counter electrode. All measurements were taken in N_2-sparged MeCN with 0.1 M NBu_4PF_6 as supporting electrolyte where the tertiary alcohol concentration was 1 mM. The sweep rate was 10 mV/s and no reversible electrochemical event was observed in all cases.

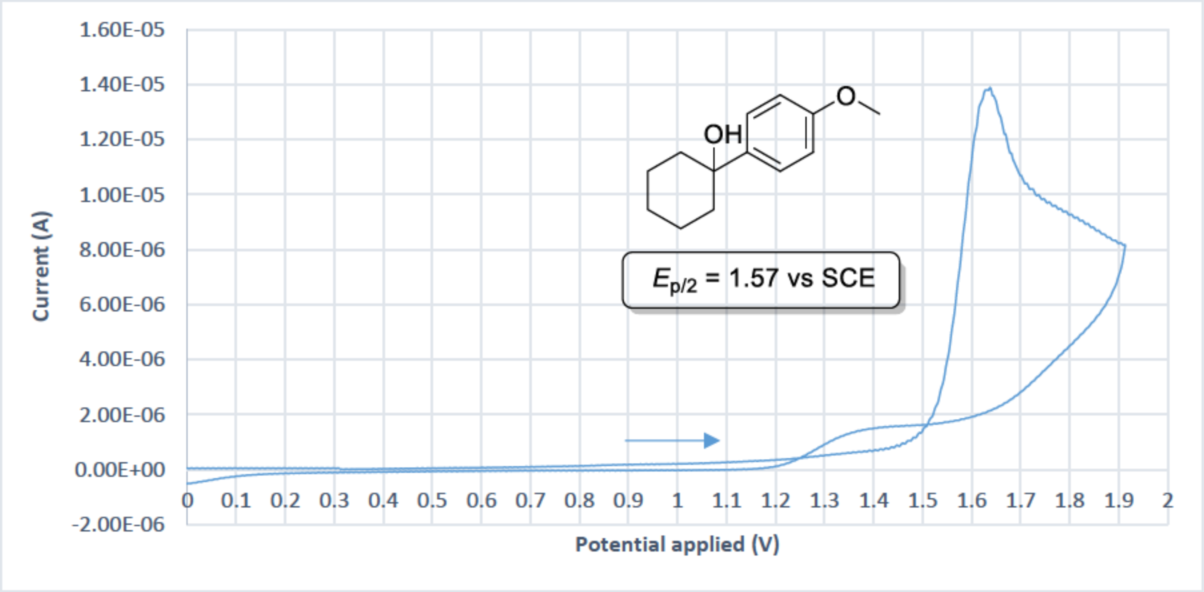

Supplementary Figure 4. Cyclic voltammogram of 1-(4-methoxyphenyl)cyclohexan-1-ol in MeCN shows an irreversible oxidation event at 1.64 V vs. SCE

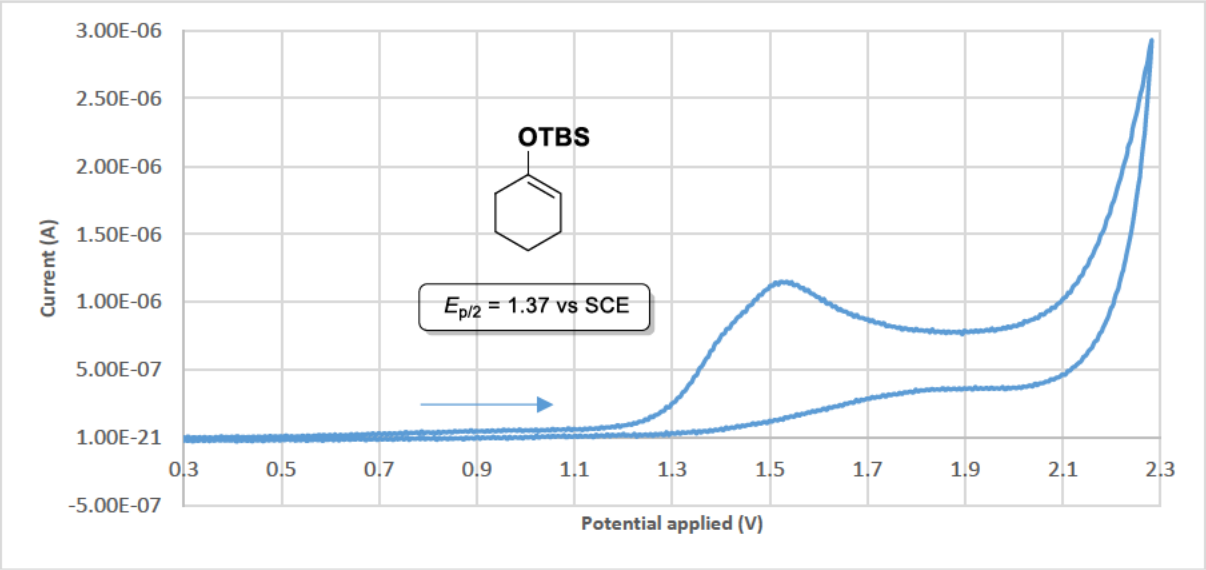

Supplementary Figure 5. Cyclic voltammogram of *tert*-butyl(cyclohex-1-en-1-yloxy)dimethylsilane in MeCN shows an irreversible oxidation event at 1.52 V vs. SCE

4. General Procedure for the Cross Coupling Reaction and Reaction Setup

General Procedure for desaturation enabled by photoredox PCET&cobaloxime dual catalysis

To a 15 mL vial equipped with a stir bar was added $Co(dmgH)_2(py)_2PF_6$ (12 mg, 0.02 mmol, 10 mol%), and photocatalyst (9-mesityl-10-methylacridinium perchlorate 7.5 mol% or $[Ir(dF(CF_3)ppy)_2(5,5'd(CF_3)bpy)](PF_6)$ 1 mol%), collidine (1 or 2 equiv.) and tertiary alcohol (0.2 mmol, 1 equiv.). The vial was sealed, evacuated and backfilled with Argon three times, then 2 mL of DCE was added. After degassing with Freeze-Pump-Thaw methods for three cycles, it was stirred and irradiated with the corresponding blue LEDs photoreactor. Upon completion, the reaction mixture was concentrated in vacuo and purified with column chromatography to afford the desired product.

General procedure for the 2 mmol scale-up cross coupling reaction

To a 100 mL flask equipped with a stir bar was added $Co(dmgH)_2(py)_2PF_6$ (120 mg, 0.2 mmol, 10 mol%), 9-mesityl-10-methylacridinium perchlorate (61.8 mg, 0.15 mmol, 7.5 mol%), collidine (485 mg (~520 μL), 4 mmol, 2 equiv.) and 1-(4-methoxyphenyl)cyclododecan-1-ol (581 mg, 2 mmol, 1 equiv.). The flask was sealed, evacuated and backfilled with Argon three times, 20 mL of DCE were added. After degassing with Freeze-Pump-Thaw methods for three cycles, it was stirred and irradiated with the corresponding blue LEDs photoreactor. 48 h later, the crude product was purified by flash column chromatography to afford **2a** as a white solid in 81% yield (467 mg).

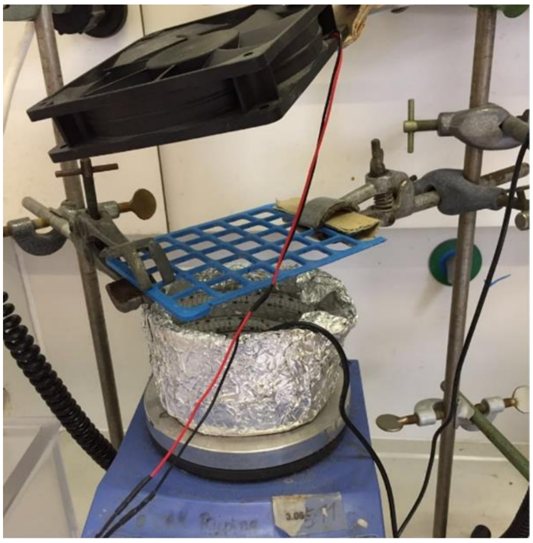

Photoreactor 1

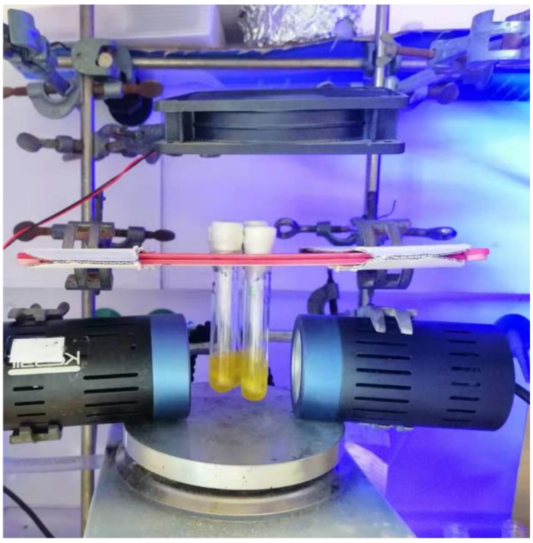

Photoreactor 2

Reaction setup: **Photoreactor 1** was assembled by wrapping the 2 m Blue LED strips (24 V) inside of a Pyrex crystallizing dish (~ 12 cm diameter). **Photoreactor 2** was assembled with 2 Kessil H-150 blue lamps (34W each). The sample was positioned 2-3 cm from strips or lamps. A fan was installed above to ensure reactions remained near rt (typically 5-10 ºC above) when using LEDs.

5. Quantification of Photocatalytic Hydrogen Gas Production

The calibration of the H_2 production rate

A dry quartz batch reactor filled with 8 mL DCE was bubbled with a continuous Ar flow (5 mL/min) for 30 min to exclude the oxygen from the reactor, then the bubbling flow was changed to the mixture gas (0.11% H_2 + 1.05 % N_2 + 1.10 % O_2 in Ar) and the reactor was cooled using the liquid cooling at 15 °C. The flow was maintained at 5 mL/min. The evolved amount of H_2 was analyzed by an online gas chromatograph (Shimadzu model GC-8A, TCD detector, molecular sieve 5A, argon carrier gas). This H_2 production rate was used for the calibration.

The hydrogen production detection for 1d as starting material

A dry quartz batch reactor equipped with a stirring bar (10mm*3mm) was charged with [Ir(dF(CF$_3$)ppy)$_2$(5,5′d(CF$_3$)bpy)](PF$_6$) **PC-IV** (9.0 mg, 0.008 mmol, 2 mol%), Co(dmgH)$_2$(py)$_2$PF$_6$ (23.7 mg, 0.04 mmol, 10 mol%) in glovebox. **1d** (82.5 mg, 0.4 mmol, 1 equiv.), 2,4,6-collidine (105 uL, 0.8 mmol, 2 equiv.), and anhydrous and degassed DCE (8.0 mL) were added subsequently via syringe. The quartz batch reactor was then transferred out of the glovebox and connected with a continuous Ar flow (5 mL/min). The reaction mixture was bubbled for 30 min to exclude the oxygen from the reactor. Next, the reactor was irradiated with 2*Kessil PR-160 440 nm LED lamps with the liquid cooling at 30 °C under continuous Ar flow (5 mL/min) for 12 h. The evolved amount of H_2 was analyzed by an online gas chromatograph (Shimadzu model GC-8A, TCD detector, molecular sieve 5A, argon carrier gas).

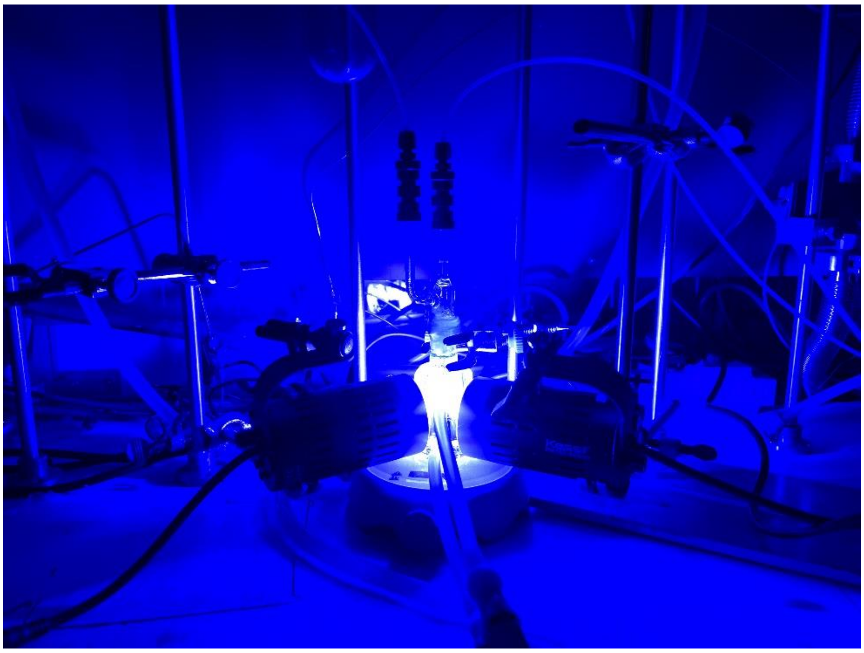

The hydrogen production detection for 5a as starting material

A dry quartz batch reactor equipped with a stirring bar (10mm*3mm) was charged with

[Ir(dF(CF$_3$)ppy)$_2$(5,5′d(CF$_3$)bpy)](PF$_6$) **PC-IV** (4.5 mg, 0.004 mmol, 1 mol%), Co(dmgH)$_2$(py)$_2$PF$_6$ (11.9 mg, 0.02 mmol, 5 mol%) in glovebox. **5a** (64.9 mg, 0.4 mmol, 1 equiv.), 2,4,6-collidine (52.7 uL, 0.4 mmol, 1 equiv.), and anhydrous and degassed DCE (8.0 mL) were added subsequently via syringe. The quartz batch reactor was then transferred out of the glovebox and connected with a continuous Ar flow (5 mL/min). The reaction mixture was bubbled for 30 min to exclude the oxygen from the reactor. Next, the reactor was irradiated with 1*Kessil PR-160 440 nm LED lamps with the liquid cooling at 15 °C under continuous Ar flow (5 mL/min) for 15 h. The evolved amount of H_2 was analyzed by an online gas chromatograph (Shimadzu model GC-8A, TCD detector, molecular sieve 5A, argon carrier gas).

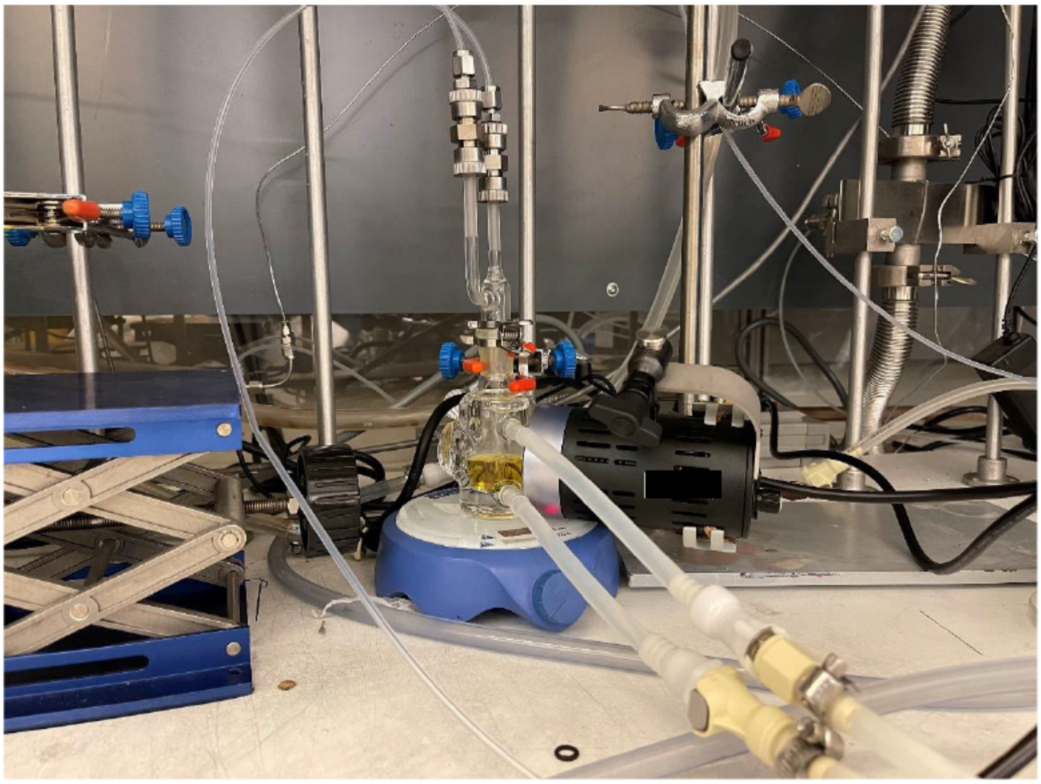

6. Characterization of the Products

1-(4-methoxyphenyl)dodec-11-en-1-one, **2a**: the title compound was prepared following the general procedure using $Co(dmgH)_2(py)_2PF_6$ (12 mg, 0.02 mmol, 10 mol%), 9-mesityl-10-methylacridinium perchlorate (6.2 mg, 0.015 mmol, 7.5 mol%), collidine (48.5 mg (~52 µL), 0.4 mmol, 2 equiv.) and 1-(4-methoxyphenyl)cyclododecan-1-ol (58.1 mg, 0.2 mmol, 1 equiv.). The reaction mixture was stirred in DCE (2 mL) and irradiated with a 2 m blue LED strip at room temperature for 48 h. The crude product was purified by flash column chromatography to afford **2a** as a white solid in 87% yield (50.3 mg).

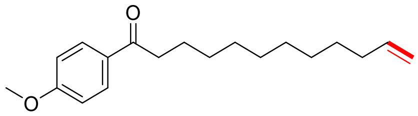

^{1}H NMR (400 MHz, $CDCl_3$): δ 7.94 (d, J = 8.8 Hz, 2H), 6.92 (d, J = 8.9 Hz, 2H), 5.87 – 5.73 (m, 1H), 5.05 – 4.86 (m, 2H), 3.86 (s, 3H), 2.90 (t, J = 7.5 Hz, 2H), 2.09 – 1.97 (m, 2H), 1.78 – 1.65 (m, 2H), 1.43 – 1.22 (m, 12H);

^{13}C NMR (101 MHz, $CDCl_3$): δ 199.3 (s), 163.4 (s), 139.3 (d), 130.4 (d), 130.3 (s), 114.2 (t), 113.8 (d), 55.5 (q), 38.4 (t), 33.9 (t), 29.6 (t, 4C), 29.2 (t), 29.0 (t), 24.8 (t);

IR (ATR) ν 3007, 2917, 2848, 1675, 1642, 1603, 1577, 1509, 1462, 1414, 1372, 1307, 1255, 1210, 1174, 1106, 1030, 999, 970, 914, 841, 809, 772 cm^{-1};

HRMS (ESI) for $C_{19}H_{28}O_2Na^+$ $(M+Na)^+$: 311.19815; Found : 311.19794.

(*E*)-1-(4-methoxyphenyl)but-2-en-1-one, **2b**:[14] the title compound was prepared following the general procedure using $Co(dmgH)_2(py)_2PF_6$ (12 mg, 0.02 mmol, 10 mol%), 9-mesityl-10-methylacridinium perchlorate (6.2 mg, 0.015 mmol, 7.5 mol%), collidine (48.5 mg (~52 µL), 0.4 mmol, 2 equiv.) and 1-(4-methoxyphenyl)cyclobutan-1-ol (35.6 mg, 0.2 mmol, 1 equiv.). The reaction mixture was stirred in DCE (2 mL) and irradiated with a 2 m blue LED strip at room temperature for 48 h. The crude product was purified by flash column chromatography to afford **2b** as a colorless oi in 51% yield (17.9 mg).

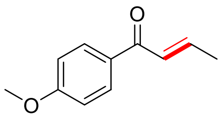

^{1}H NMR (600 MHz, $CDCl_3$): δ 7.95 (d, J = 8.8 Hz, 2H), 7.05 (dq, J = 14.9, 6.8 Hz, 1H), 6.97 – 6.87 (m, 3H), 3.87 (s, 3H), 1.99 (dd, J = 6.9, 1.6 Hz, 3H).

^{13}C NMR (151 MHz, $CDCl_3$): δ 189.1 (s), 163.4 (s), 144.1 (d), 130.9 (d), 130.8 (s), 127.2 (d), 113.8 (d), 55.6 (q), 18.7 (q).

1-(4-methoxyphenyl)pent-4-en-1-one, **2c**:[15] the title compound was prepared following the general procedure using $Co(dmgH)_2(py)_2PF_6$ (12 mg, 0.02 mmol, 10 mol%), 9-mesityl-10-methylacridinium perchlorate (6.2 mg, 0.015 mmol, 7.5 mol%), collidine (48.5 mg (~52 µL), 0.4 mmol, 2 equiv.) and 1-(4-

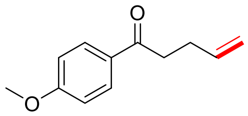

methoxyphenyl)cyclopentan-1-ol (38.5 mg, 0.2 mmol, 1 equiv.). The reaction mixture was stirred in DCE (2 mL) and irradiated with a 2 m blue LED strip at room temperature for 48 h. The crude product was purified by flash column chromatography to afford **2c** as a colorless oil in 68% yield (25.8 mg).

^{1}H NMR (400 MHz, CDCl$_3$): δ 7.94 (d, J = 8.9 Hz, 2H), 6.93 (d, J = 8.9 Hz, 2H), 5.99 – 5.81 (m, 1H), 5.16 – 4.91 (m, 2H), 3.86 (s, 3H), 3.07 – 2.97 (m, 2H), 2.54 – 2.43 (m, 2H).

^{13}C NMR (101 MHz, CDCl$_3$): δ 198.2 (s), 163.5 (s), 137.6 (d), 130.4 (d), 130.2 (s), 115.3 (t), 113.8 (d), 55.6 (q), 37.5 (t), 28.5 (t).

1-(4-methoxyphenyl)hex-5-en-1-one, **2d**:[16] the title compound was prepared following the

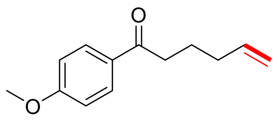

general procedure using Co(dmgH)$_2$(py)$_2$PF$_6$ (12 mg, 0.02 mmol, 10 mol%), 9-mesityl-10-methylacridinium perchlorate (6.2 mg, 0.015 mmol, 7.5 mol%), collidine (48.5 mg (~52 µL), 0.4 mmol, 2 equiv.) and 1-(4-methoxyphenyl)cyclohexan-1-ol (41.3 mg, 0.2 mmol, 1 equiv.). The reaction mixture was stirred in DCE (2 mL) and irradiated with a 2 m blue LED strip at room temperature for 48 h. The crude product was purified by flash column chromatography to afford **2d** as a colorless oil in 84% yield (34.3 mg).

^{1}H NMR (600 MHz, CDCl$_3$): δ 7.94 (d, *J* = 8.9 Hz, 2H), 6.93 (d, *J* = 8.9 Hz, 2H), 5.82 (td, *J* = 16.9, 6.7 Hz, 1H), 5.09 – 4.95 (m, 2H), 3.86 (s, 3H), 2.92 (t, *J* = 7.4 Hz, 2H), 2.19 – 2.10 (m, 2H), 1.88 – 1.79 (m, 2H).

^{13}C NMR (151 MHz, CDCl$_3$): δ 199.0 (s), 163.4 (s), 138.3 (d), 130.4 (d), 130.3 (s), 115.3 (t), 113.8 (d), 55.6 (q), 37.5 (t), 33.4 (t), 23.7 (t).

1-(4-methoxyphenyl)hept-6-en-1-one, **2e**:[17] the title compound was prepared following the

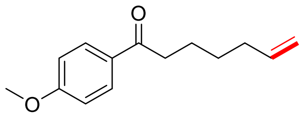

general procedure using Co(dmgH)$_2$(py)$_2$PF$_6$ (12 mg, 0.02 mmol, 10 mol%), 9-mesityl-10-methylacridinium perchlorate (6.2 mg, 0.015 mmol, 7.5 mol%), collidine (48.5 mg (~52 µL), 0.4 mmol, 2 equiv.) and 1-(4-methoxyphenyl)cycloheptan-1-ol (44.1 mg, 0.2 mmol, 1 equiv.). The reaction mixture was stirred in DCE (2 mL) and irradiated with a 2 m blue LED strip at room temperature for 48 h. The crude product was purified by flash column chromatography to afford **xx** as a colorless oi in 89% yield (38.7 mg).

^{1}H NMR (400 MHz, CDCl$_3$): δ 7.93 (d, *J* = 8.9 Hz, 2H), 6.92 (d, *J* = 8.9 Hz, 2H), 5.90 – 5.73 (m, 1H), 5.11 – 4.87 (m, 2H), 3.86 (s, 3H), 2.98 – 2.85 (m, 2H), 2.18 – 2.04 (m, 2H), 1.82 – 1.69 (m, 2H), 1.55 – 1.42 (m, 2H);

^{13}**C NMR (101 MHz, CDCl$_3$):** δ 199.1 (s), 163.4 (s), 138.7 (d), 130.4 (d), 130.3 (s), 114.7 (t), 113.8 (d), 55.6 (q), 38.2 (t), 33.7 (t), 28.8 (t), 24.2 (t);

1-(4-methoxyphenyl)oct-7-en-1-one, **2f**: the title compound was prepared following the general

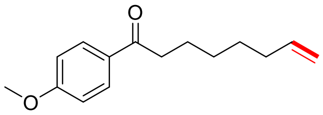

procedure using Co(dmgH)$_2$(py)$_2$PF$_6$ (12 mg, 0.02 mmol, 10 mol%), 9-mesityl-10-methylacridinium perchlorate (6.2 mg, 0.015 mmol, 7.5 mol%), collidine (48.5 mg (~52 µL), 0.4 mmol, 2 equiv.) and 1-(4-methoxyphenyl)cyclooctan-1-ol (46.9 mg, 0.2 mmol, 1 equiv.). The reaction mixture was stirred in DCE (2 mL) and irradiated with a 2 m blue LED strip at room temperature for 48 h. The crude product was purified by flash column chromatography to afford **2f** as a white solid in 68% yield (31.7 mg).

^{1}**H NMR (400 MHz, CDCl$_3$):** δ 7.94 (d, J = 8.9 Hz, 2H), 6.93 (d, J = 8.9 Hz, 2H), 5.88 – 5.73 (m, 1H), 5.08 – 4.86 (m, 2H), 3.86 (s, 3H), 2.91 (t, J = 7.4 Hz, 2H), 2.15 – 2.00 (m, 2H), 1.80 – 1.67 (m, 2H), 1.50 – 1.31 (m, 4H).;

^{13}**C NMR (101 MHz, CDCl$_3$):** δ 199.2 (s), 163.4 (s), 139.0 (d), 130.4 (s), 130.3 (s), 114.5 (t), 113.8 (d), 55.6 (q), 38.4 (t), 33.8 (t), 29.0 (t), 28.9 (t), 24.6 (t);

IR (ATR) ν 3077, 2970, 3004, 2929, 2850, 1669, 1642, 1600, 1575, 1508, 1462, 1414, 1370, 1311, 1281, 1250, 1173, 1108, 1029, 973, 915, 845, 815, 760 cm^{-1};

HRMS (ESI) for $C_{15}H_{20}O_2Na^+$ $(M+Na)^+$: 255.13555; Found : 255.13551.

1-(4-methoxyphenyl)dodec-11-en-1-one, **2g**: the title compound was prepared following the

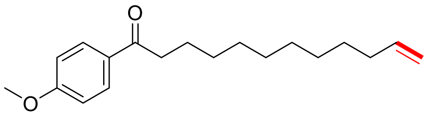

general procedure using Co(dmgH)$_2$(py)$_2$PF$_6$ (12 mg, 0.02 mmol, 10 mol%), 9-mesityl-10-methylacridinium perchlorate (6.2 mg, 0.015 mmol, 7.5 mol%), collidine (48.5 mg (~52 µL), 0.4 mmol, 2 equiv.) and 1-(4-methoxyphenyl)cyclododecan-1-ol (58.1 mg, 0.2 mmol, 1 equiv.). The reaction mixture was stirred in DCE (2 mL) and irradiated with a 2 m blue LED strip at room temperature for 48 h. The crude product was purified by flash column chromatography to afford **2g** as a white solid in 87% yield (50.3 mg).

^{1}**H NMR (400 MHz, CDCl$_3$):** δ 7.94 (d, J = 8.8 Hz, 2H), 6.92 (d, J = 8.9 Hz, 2H), 5.87 – 5.73 (m, 1H), 5.05 – 4.86 (m, 2H), 3.86 (s, 3H), 2.90 (t, J = 7.5 Hz, 2H), 2.09 – 1.97 (m, 2H), 1.78 – 1.65 (m, 2H), 1.43 – 1.22 (m, 12H);

^{13}**C NMR (101 MHz, CDCl$_3$):** δ 199.3 (s), 163.4 (s), 139.3 (d), 130.4 (d), 130.3 (s), 114.2 (t), 113.8 (d), 55.5 (q), 38.4 (t), 33.9 (t), 29.6 (t, 4C), 29.2 (t), 29.0 (t), 24.8 (t);

IR (ATR) ν 3007, 2917, 2848, 1675, 1642, 1603, 1577, 1509, 1462, 1414, 1372, 1307, 1255, 1210, 1174, 1106, 1030, 999, 970, 914, 841, 809, 772 cm^{-1};

HRMS (ESI) for $C_{19}H_{28}O_2Na^+$ $(M+Na)^+$: 311.19815; Found : 311.19794.

1-(4-methoxyphenyl)pentadec-14-en-1-one, **2h**: the title compound was prepared following the general procedure using $Co(dmgH)_2(py)_2PF_6$ (12 mg, 0.02 mmol, 10 mol%), 9-mesityl-10-methylacridinium perchlorate (6.2 mg, 0.015 mmol, 7.5 mol%), collidine (48.5 mg (~52 µL), 0.4 mmol, 2 equiv.) and 1-(4-methoxyphenyl)cyclopentadecan-1-ol (66.5 mg, 0.2 mmol, 1 equiv.). The reaction mixture was stirred in DCE (2 mL) and irradiated with a 2 m blue LED strip at room temperature for 48 h. The crude product was purified by flash column chromatography to afford **2h** as a white solid in 88% yield (58.4 mg).

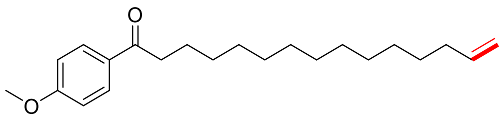

^{1}H NMR (400 MHz, $CDCl_3$): δ 7.94 (d, J = 8.9 Hz, 2H), 6.92 (d, J = 8.9 Hz, 2H), 5.88 – 5.73 (m, 1H), 5.05 – 4.83 (m, 2H), 3.86 (s, 3H), 2.90 (t, J = 7.4 Hz, 2H), 2.10 – 1.97 (m, 2H), 1.77 – 1.65 (m, 2H), 1.45 – 1.16 (m, 18H);

^{13}C NMR (101 MHz, $CDCl_3$): δ 199.3 (s), 163.4 (s), 139.4 (d), 130.4 (d), 130.3 (s), 114.2 (t), 113.8 (d), 55.5 (q), 38.4 (t), 33.9 (t), 29.8 (t), 29.7 (t), 29.6 (t), 29.3 (t), 29.1 (t), 24.8 (t);

IR (ATR) ν 2916, 2848, 1676, 1642, 1604, 1579, 1509, 1463, 1415, 1372, 1307, 1254, 1175, 1106, 1030, 992, 971, 913, 842, 813, 766 cm^{-1};

HRMS (ESI) for $C_{22}H_{34}O_2Na^+$ $(M+Na)^+$: 353.24510; Found : 353.24493.

1-(4-methoxyphenyl)-4-phenylhex-5-en-1-one, **2i**:[18] the title compound was prepared following the general procedure using $Co(dmgH)_2(py)_2PF_6$ (12 mg, 0.02 mmol, 10 mol%), 9-mesityl-10-methylacridinium perchlorate (6.2 mg, 0.015 mmol, 7.5 mol%), collidine (48.5 mg (~52 µL), 0.4 mmol, 2 equiv.) and 1-(4-methoxyphenyl)-4-phenylcyclohexan-1-ol (56.5 mg, 0.2 mmol, 1 equiv.). The reaction mixture was stirred in DCE (2 mL) and irradiated with a 2 m blue LED strip at room temperature for 48 h. The crude product was purified by flash column chromatography to afford **2i** as a white solid in 53% yield (29.8 mg).

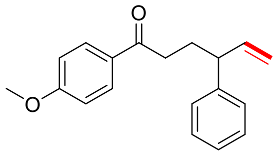

^{1}H NMR (400 MHz, $CDCl_3$): δ 7.87 (d, J = 8.8 Hz, 2H), 7.36 – 7.27 (m, 2H), 7.26 – 7.17 (m, 3H), 6.90 (d, J = 8.8 Hz, 2H), 5.99 (ddd, J = 16.8, 10.3, 7.6 Hz, 1H), 5.15 – 5.01 (m, 2H), 3.86 (s, 3H), 3.41 – 3.31 (m, 1H), 2.96 – 2.78 (m, 2H), 2.25 – 2.08 (m, 2H);

^{13}C NMR (101 MHz, $CDCl_3$): δ 198.8 (s), 163.5 (s), 143.8 (s), 141.8 (d), 130.4 (d), 130.2 (s), 128.7 (d), 127.8 (d), 126.5 (d), 114.8 (t), 113.8 (d), 55.6 (q), 49.4 (d), 36.2 (t), 29.9 (t);

1-(4-methoxyphenyl)-4-(trifluoromethyl)hex-5-en-1-one, **2j**: the title compound was prepared following the general procedure using Co(dmgH)$_2$(py)$_2$PF$_6$ (12 mg, 0.02 mmol, 10 mol%), [Ir(dF(CF$_3$)ppy)$_2$(5,5′d(CF$_3$)bpy)](PF$_6$) (2.29 mg, 0.002 mmol, 1 mol%), collidine (48.5 mg (~52 μL), 0.4 mmol, 2 equiv.) and 1-(4-methoxyphenyl)-4-(trifluoromethyl)cyclohexan-1-ol (54.9 mg, 0.2 mmol, 1 equiv.). The reaction mixture was stirred in DCE (2 mL) and irradiated with two Kessil 34 W lamps at room temperature for 72 h. The crude product was purified by flash column chromatography to afford **2j** as a colorless oil in 42% yield (22.7 mg).

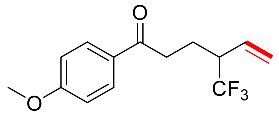

^{1}H NMR (600 MHz, CDCl$_3$): δ 7.92 (d, J = 8.6 Hz, 2H), 6.93 (d, J = 8.8 Hz, 2H), 5.66 (dt, J = 17.0, 9.7 Hz, 1H), 5.33 (d, J = 10.3 Hz, 1H), 5.27 (d, J = 17.1 Hz, 1H), 3.87 (s, 3H), 3.10 – 2.80 (m, 3H), 2.35 – 2.16 (m, 1H), 1.93 – 1.79 (m, 1H).

^{13}C NMR (151 MHz, CDCl$_3$): δ 197.4, 163.7, 131.5, 130.3, 130.0, 126.9 (q, $J_{C\text{-}F}$ = 280.5 Hz), 121.9, 113.9, 55.6, 47.9 (q, $J_{C\text{-}F}$ = 26.7 Hz), 34.6, 22.1.

^{19}F NMR (564 MHz, CDCl$_3$): δ -70.9.

IR (ATR) ν 2939, 1676, 1600, 1510, 1419, 1368, 1307, 1252, 1216, 1166, 1103, 1028, 988, 936, 836 cm^{-1};

HRMS (ESI) for $C_{14}H_{15}F_3O_2Na^+$ $(M+Na)^+$: 295.0916; Found : 295.0911.

4-((*tert*-butyldimethylsilyl)oxy)-1-(4-methoxyphenyl)hex-5-en-1-one, **2k**: the title compound was prepared following the general procedure using Co(dmgH)$_2$(py)$_2$PF$_6$ (12 mg, 0.02 mmol, 10 mol%), [Ir(dF(CF$_3$)ppy)$_2$(5,5′d(CF$_3$)bpy)](PF$_6$) (2.29 mg, 0.002 mmol, 1 mol%), collidine (48.5 mg (~52 μL), 0.4 mmol, 2 equiv.) and 4-((*tert*-butyldimethylsilyl)oxy)-1-(4-methoxyphenyl)cyclohexan-1-ol (67.3 mg, 0.2 mmol, 1 equiv.). The reaction mixture was stirred in DCE (2 mL) and irradiated with two Kessil 34 W lamps at room temperature for 72 h. Due to the difficulty in separation between the desired olefin product and ring-opened side product, the yield was determined to be 53% according to ^{1}H NMR analysis of the crude mixture using CH_2Br_2 as an internal standard.

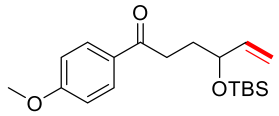

^{1}H NMR (600 MHz, CDCl$_3$): δ 7.94 (d, J = 8.4 Hz, 2H), 6.93 (d, J = 9.1 Hz, 2H), 5.89 – 5.75 (m, 1H), 5.20 (d, J = 17.8 Hz, 1H), 5.07 (d, J = 9.5 Hz, 1H), 4.31 – 4.23 (m, 1H), 3.87 (s, 3H), 3.03 – 2.91 (m, 2H), 2.00 – 1.91 (m, 1H), 1.92 – 1.83 (m, 1H), 0.90 (s, 9H), 0.04 (s, 6H).

^{13}C NMR (151 MHz, CDCl$_3$): δ 199.0, 163.5, 141.3, 130.4, 130.3, 114.4, 113.8, 72.8, 55.6, 33.6, 32.3, 26.0, 18.4, -4.3, -4.7.

IR (ATR) ν 2931, 2856, 1677, 1600, 1510, 1463, 1417, 1362, 1315, 1252, 1170, 1118, 1075, 1028, 925, 834 cm^{-1};

HRMS (ESI) for $C_{19}H_{30}O_3SiNa^+$ $(M+Na)^+$: 357.1856; Found : 357.1852.

1-(4-methoxyphenyl)-3-(2-vinyl-1,3-dioxolan-2-yl)propan-1-one, **2l**: the title compound was prepared following the general procedure using $Co(dmgH)_2(py)_2PF_6$ (12 mg, 0.02 mmol, 10 mol%), 9-mesityl-10-methylacridinium perchlorate (6.2 mg, 0.015 mmol, 7.5 mol%), collidine (48.5 mg (~52 μL), 0.4 mmol, 2 equiv.) and 8-(4-methoxyphenyl)-1,4-dioxaspiro[4.5]decan-8-ol (52.9 mg, 0.2 mmol, 1 equiv.). The reaction mixture was stirred in DCE (2 mL) and irradiated with a 2 m blue LED strip at room temperature for 48 h. The crude product was purified by flash column chromatography to afford **2l** as a white solid in 53% yield (27.8 mg).

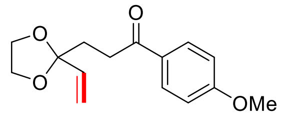

^{1}H NMR (400 MHz, $CDCl_3$): δ 7.93 (d, J = 8.8 Hz, 2H), 6.90 (d, J = 8.9 Hz, 2H), 5.74 (dd, J = 17.2, 10.6 Hz, 1H), 5.38 (dd, J = 17.2, 1.8 Hz, 1H), 5.17 (dd, J = 10.6, 1.8 Hz, 1H), 3.97 – 3.86 (m, 4H), 3.84 (s, 3H), 3.05 – 2.96 (m, 2H), 2.23 – 2.12 (m, 2H);

^{13}C NMR (101 MHz, $CDCl_3$): δ 198.4 (s), 163.4 (s), 137.6 (d), 130.4 (d), 130.3 (s), 116.0 (t), 113.8 (d), 108.7 (d), 64.8 (t), 55.6 (q), 32.4 (t), 32.3 (t);

IR (ATR) ν 2952, 2892, 2323, 2074, 1920, 1674, 1597, 1510, 1456, 1416, 1310, 1254, 1170, 1033, 939, 902, 833 cm^{-1};

HRMS (ESI) for $C_{15}H_{18}O_4Na^+$ $(M+Na)^+$: 285.1097; Found : 285.1096.

1-(4-methoxyphenyl)-3-(vinyloxy)propan-1-one, **2m**: the title compound was prepared following the general procedure using $Co(dmgH)_2(py)_2PF_6$ (12 mg, 0.02 mmol, 10 mol%), 9-mesityl-10-methylacridinium perchlorate (6.2 mg, 0.015 mmol, 7.5 mol%), collidine (48.5 mg (~52 μL), 0.4 mmol, 2 equiv.) and 4-(4-methoxyphenyl)tetrahydro-2H-pyran-4-ol (41.7 mg, 0.2 mmol, 1 equiv.). The reaction mixture was stirred in DCE (2 mL) and irradiated with a 2 m blue LED strip at room temperature for 48 h. The crude product was purified by flash column chromatography to afford **2m** as a white solid in 47% yield (19.3 mg).

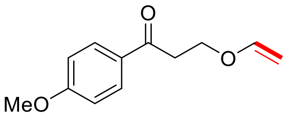

^{1}H NMR (400 MHz, $CDCl_3$): δ 7.95 (d, J = 8.9 Hz, 2H), 6.94 (d, J = 8.9 Hz, 2H), 6.46 (dd, J = 14.3, 6.8 Hz, 1H), 4.25 (dd, J = 14.4, 2.1 Hz, 1H), 4.14 (t, J = 6.6 Hz, 2H), 4.03 (dd, J = 6.8, 2.1 Hz, 1H), 3.87 (s, 3H), 3.29 (t, J = 6.6 Hz, 2H);

^{13}C NMR (101 MHz, $CDCl_3$): δ 196.3 (s), 163.8 (s), 151.7 (d), 130.5 (d), 130.1 (s), 113.9 (d), 87.1 (t), 63.6 (t), 55.6 (q), 37.6 (t);

IR (ATR) ν 3011, 2933, 2842, 1664, 1602, 1509, 1464, 1419, 1384, 1322, 1254, 1199, 1167, 1104, 1029, 969, 846, 801, 757 cm^{-1};

HRMS (ESI) for $C_{12}H_{14}O_3Na^+$ $(M+Na)^+$: 229.08352; Found : 229.08331.

4,4-difluoro-1-(4-methoxyphenyl)hex-5-en-1-one, **2n**: the title compound was prepared

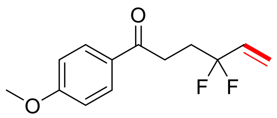

following the general procedure using $Co(dmgH)_2(py)_2PF_6$ (12 mg, 0.02 mmol, 10 mol%), $[Ir(dF(CF_3)ppy)_2(5,5'd(CF_3)bpy)](PF_6)$ (2.29 mg, 0.002 mmol, 1 mol%), collidine (48.5 mg (~52 μL), 0.4 mmol, 2 equiv.) and 4,4-difluoro-1-(4-methoxyphenyl)cyclohexan-1-ol (48.5 mg, 0.2 mmol, 1 equiv.). The reaction mixture was stirred in DCE (2 mL) and irradiated with two Kessil 34 W lamps at room temperature for 72 h. The crude product was purified by flash column chromatography to afford **2n** as a colorless oil in 66% yield (31.5 mg).

^{1}H NMR (600 MHz, $CDCl_3$): δ 7.95 (d, J = 8.3 Hz, 2H), 6.94 (d, J = 8.4 Hz, 2H), 6.02 – 5.85 (m, 1H), 5.66 (d, J = 17.4 Hz, 1H), 5.44 (d, J = 10.9 Hz, 1H), 3.87 (s, 3H), 3.17 – 3.10 (m, 2H), 2.44 – 2.32 (m, 2H).

^{13}C NMR (151 MHz, $CDCl_3$): δ 196.6, 163.8, 133.0 (t, $J_{C\text{-}F}$ = 27.8 Hz), 130.4, 129.8, 121.2 (t, $J_{C\text{-}F}$ = 238.1 Hz), 119.6 (t, $J_{C\text{-}F}$ = 9.6 Hz), 113.9, 55.6, 31.5 (t, $J_{C\text{-}F}$ = 26.7 Hz), 31.1 (t, $J_{C\text{-}F}$ = 3.6 Hz).

^{19}F NMR (564 MHz, $CDCl_3$): δ -98.7.

IR (ATR) ν 3469, 2948, 1676, 1603, 1511, 1450, 1302, 1251, 1176, 1031, 964, 832 cm^{-1};

HRMS (ESI) for $C_{13}H_{14}F_2O_2Na^+$ $(M+Na)^+$: 263.0854; Found : 263.0851.

5-(4-methoxyphenyl)-5-oxo-2-phenyl-2-vinylpentanenitrile, **2o**: the title compound was

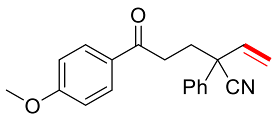

prepared following the general procedure using $Co(dmgH)_2(py)_2PF_6$ (12 mg, 0.02 mmol, 10 mol%), $[Ir(dF(CF_3)ppy)_2(5,5'd(CF_3)bpy)](PF_6)$ (2.29 mg, 0.002 mmol, 1 mol%), collidine (48.5 mg (~52 μL), 0.4 mmol, 2 equiv.) and 4-hydroxy-4-(4-methoxyphenyl)-1-phenylcyclohexane-1-carbonitrile (61.5 mg, 0.2 mmol, 1 equiv.). The reaction mixture was stirred in DCE (2 mL) and irradiated with two Kessil 34 W lamps at room temperature for 72 h. The crude product was purified by flash column chromatography to afford **2o** as a white solid in 55% yield (33.3 mg).

^{1}H NMR (600 MHz, $CDCl_3$): δ 7.90 (d, J = 8.5 Hz, 2H), 7.49 (d, J = 7.7 Hz, 2H), 7.41 (t, J = 7.6 Hz, 2H), 7.33 (t, J = 7.4 Hz, 1H), 6.91 (d, J = 8.4 Hz, 2H), 5.96 (dd, J = 17.0, 10.2 Hz, 1H), 5.60 (d, J = 17.0 Hz, 1H), 5.35 (d, J = 10.2 Hz, 1H), 3.86 (s, 3H), 3.22 – 3.10 (m, 1H), 3.02 – 2.92 (m, 1H), 2.58 – 2.46 (m, 2H).

^{13}C NMR (151 MHz, $CDCl_3$): δ 196.8, 163.8, 138.2, 137.4, 130.4, 129.3, 128.4, 126.2, 120.5, 116.9, 113.9, 55.6, 50.0, 34.3, 33.8;

IR (ATR) ν 2919, 1667, 1598, 1507, 1414, 1368, 1312, 1256, 1171, 1028, 987, 938, 837, 767 cm^{-1};

HRMS (ESI) for $C_{20}H_{19}NO_2Na^+$ $(M+Na)^+$: 328.1308; Found : 328.1302.

***tert*-butyl (6-(4-methoxyphenyl)-6-oxohex-1-en-3-yl)carbamate**, **2p**: the title compound was prepared following the general procedure using Co(dmgH)$_2$(py)$_2$PF$_6$ (12 mg, 0.02 mmol, 10 mol%), [Ir(dF(CF$_3$)ppy)$_2$(5,5′d(CF$_3$)bpy)](PF$_6$) (2.29 mg, 0.002 mmol, 1 mol%), collidine (48.5 mg (~52 μL), 0.4 mmol, 2 equiv.) and *tert*-butyl (4-hydroxy-4-(4-methoxyphenyl)cyclohexyl)carbamate (64.3 mg, 0.2 mmol, 1 equiv.). The reaction mixture was stirred in DCE (2 mL) and irradiated with two Kessil 34 W lamps at room temperature for 72 h. The crude product was purified by flash column chromatography to afford **2p** as a white solid in 68% yield (43.5 mg).

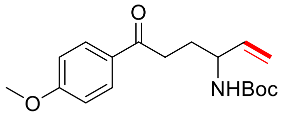

^{1}H NMR (600 MHz, CDCl$_3$): δ 7.92 (d, J = 8.5 Hz, 2H), 6.92 (d, J = 8.5 Hz, 2H), 5.85 – 5.69 (m, 1H), 5.19 (d, J = 17.2 Hz, 1H), 5.11 (d, J = 10.4 Hz, 1H), 4.61 (s, 1H), 4.17 (s, 1H), 3.85 (s, 3H), 2.99 (t, J = 7.3 Hz, 2H), 2.04 – 1.95 (m, 1H), 1.91 (s, 1H), 1.40 (s, 9H).

^{13}C NMR (151 MHz, CDCl$_3$): δ 198.4, 163.6, 155.6, 138.8, 130.4, 130.1, 115.0, 113.8, 79.5, 55.6, 52.8, 34.8, 29.4, 28.5.

IR (ATR) ν 3366, 2976, 1671, 1594, 1509, 1259, 1163, 1013, 908, 838 cm^{-1};

HRMS (ESI) for $C_{18}H_{25}NO_4Na^+$ $(M+Na)^+$: 342.1676; Found : 342.1671.

((1S,3S,5R)-Bicyclo[3.3.1]non-6-en-3-yl)(4-methoxyphenyl)methanone, **2q**: the title compound was prepared following the general procedure using Co(dmgH)$_2$(py)$_2$PF$_6$ (6 mg, 0.01 mmol, 5 mol%), 9-mesityl-10-methylacridinium perchlorate (6.2 mg, 0.015 mmol, 7.5 mol%), collidine (48.5 mg (~52 μL), 0.4 mmol, 2 equiv.) and (1R,3S,5r,7r)-2-(4-methoxyphenyl)adamantan-2-ol (51.7 mg, 0.2 mmol, 1 equiv.). The reaction mixture was stirred in DCE (2 mL) and irradiated with a 2 m blue LED strip at room temperature for 72 h. The crude product was purified by flash column chromatography to afford **2q** as a colorless solid in 50% yield (25.8 mg).

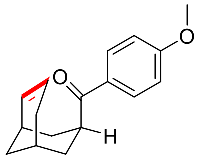

^{1}H NMR (600 MHz, CDCl$_3$): δ 7.65 (d, J = 8.2 Hz, 2H), 6.89 (d, J = 8.3 Hz, 2H), 5.55 – 5.49 (m, 1H), 5.47 (t, J = 7.9 Hz, 1H), 3.84 (s, 3H), 3.30 – 3.22 (m, 1H), 2.31 (dd, J = 14.1, 4.1 Hz, 1H), 2.28 – 2.19 (m, 2H), 2.18 – 2.12 (m, 2H), 2.11 – 2.06 (m, 1H), 1.99 (dt, J = 13.7, 6.5 Hz, 1H), 1.84 (dt, J = 13.2, 6.4 Hz, 1H), 1.65 – 1.58 (m, 2H).

^{13}C NMR (151 MHz, CDCl$_3$): δ 203.6 (s), 162.1 (s), 130.8 (s), 130.5 (d), 130.2 (d), 128.8 (d), 113.4 (d), 55.4 (q), 39.3 (d), 32.9 (t), 30.7 (t), 30.0 (t), 30.0 (t), 28.0 (d), 25.7 (d).

IR (ATR) ν 3020, 2909, 1672, 1598, 1508, 1446, 1318, 1238, 1171, 1111, 1021, 968, 922, 835, 773, 707 cm^{-1};

HRMS (ESI) for $C_{17}H_{20}O_2Na^+$ $(M+Na)^+$: 279.1356; Found : 279.1356.

1-(4-Methoxyphenyl)-6-phenylhex-5-en-1-one, **2r**:[16] the title compounds were prepared following the general procedure using $Co(dmgH)_2(py)_2PF_6$ (12 mg, 0.02 mmol, 10 mol%), 9-mesityl-10-methylacridinium perchlorate (6.2 mg, 0.015 mmol, 7.5 mol%), collidine (48.5 mg (~52 μL), 0.4 mmol, 2 equiv.) and 1-(4-methoxyphenyl)-2-phenylcyclohexan-1-ol (56.5 mg, 0.2 mmol, 1 equiv.). The reaction mixture was stirred in DCE (2 mL) and irradiated with a 2 m blue LED strip at room temperature for 48 h. The crude product was purified by flash column chromatography to afford **2r** as a white solid in 60% yield (*E*:*Z* ratio = 10:1, 33.5 mg).

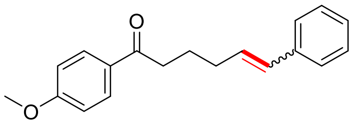

E:*Z* = 10:1

^{1}H NMR (400 MHz, $CDCl_3$): *E* isomer δ 7.93 (d, *J* = 8.9 Hz, 2H), 7.36 – 7.25 (m, 4H), 7.18 (t, *J* = 7.1 Hz, 1H), 6.91 (d, *J* = 8.8 Hz, 2H), 6.40 (d, *J* = 15.8 Hz, 1H), 6.21 (dt, *J* = 15.6, 6.9 Hz, 1H), 3.85 (s, 3H), 2.96 (t, *J* = 7.3 Hz, 2H), 2.30 (td, $J_1 = J_2$ = 7.1 Hz, 2H), 1.98 – 1.86 (m, 2H); *Z* isomer δ 6.71 (d, *J* = 8.9 Hz, 2H), 6.45 (d, *J* = 12.2 Hz, 1H), 5.67 (dt, *J* = 11.6, 7.2 Hz, 1H), 3.73 (s, 3H), 2.42 (m, 2H), the remaining resonances are insufficiently resolved from those of the *E* isomer to be reported.

^{13}C NMR (101 MHz, $CDCl_3$): only *E* isomer was shown δ 198.9 (s), 163.5 (s), 137.8 (s), 130.8 (d), 130.4 (d), 130.3 (s), 130.2 (d), 128.6 (d), 127.1 (d), 126.1 (d), 113.8 (d), 55.6 (q), 37.5 (t), 32.6 (t), 24.1 (t).

Mixture of 1-(4-methoxyphenyl)hept-6-en-1-one and 1-(4-methoxyphenyl)hept-5-en-1-one, **2s**: the title compounds were prepared following the general procedure using $Co(dmgH)_2(py)_2PF_6$ (6 mg, 0.01 mmol, 5 mol%), 9-mesityl-10-methylacridinium perchlorate (6.2 mg, 0.015 mmol, 7.5 mol%), collidine (48.5 mg (~52 μL), 0.4 mmol, 2 equiv.) and 1-(4-methoxyphenyl)-2-methylcyclohexan-1-ol (44.1 mg, 0.2 mmol, 1 equiv.). The reaction mixture was stirred in DCE (2 mL) and irradiated with a 2 m blue LED strip at room temperature for 72 h. The crude product was purified by flash column chromatography to afford **2s** as a white solid in 82% yield (35.9 mg).

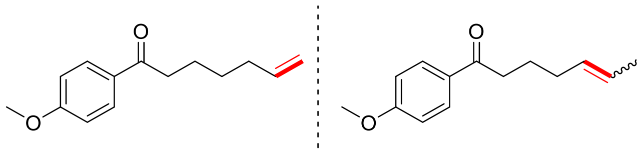

marjor ismer / minor isomer = 2:1 (minor *E*/*Z* 2:1)

^{1}H NMR (600 MHz, $CDCl_3$): δ 7.99 – 7.88 (m, 3$H_{major+minor}$), 6.97 – 6.87 (m, 3$H_{major+minor}$), 5.81 (ddt, J = 16.9, 10.2, 6.7 Hz, 1H_{major}), 5.52 – 5.36 (m, 1H_{minor}), 5.08 – 4.88 (m, 2H_{major}), 3.86 (s, 4.5$H_{major+minor}$), 2.96 – 2.85 (m, 3$H_{major+minor}$), 2.17 – 2.02 (m, 3$H_{major+minor}$), 1.83 – 1.68 (m, 3$H_{major+minor}$), 1.64 (d, *J* = 4.9 Hz, 1$H_{minor\ E}$), 1.61 – 1.57 (dt, *J* = 6.7, 0.9 Hz, 1$H_{minor\ Z}$), 1.51 – 1.43 (m, 2H_{major});

^{13}C NMR (151 MHz, $CDCl_3$): δ 199.2 (s), 199.1 (s), 199.1 (s), 163.4 (s, 2C), 138.7 (d), 130.7 (d), 130.4 (d), 130.3 (s), 130.2 (s), 129.9 (s), 125.8 (d), 124.9 (d), 114.7 (s), 113.8 (d), 113.7 (s), 55.6 (q), 38.2 (t), 37.6 (t, 2C), 33.7 (t), 32.2 (t), 28.8 (t), 26.5 (t), 24.4 (t, 2C), 24.2 (t), 18.1 (q, minor isomer *E*), 13.0 (q, minor isomer *Z*);

IR (ATR) ν 2930, 2850, 1674, 1596, 1510, 1451, 1421, 1363, 1311, 1248, 1169, 1027, 979, 910, 830 cm^{-1};

HRMS (ESI) for $C_{14}H_{18}O_2Na^+$ $(M+Na)^+$: 241.1199; Found : 241.1200.

Mixture of 2-(cyclopent-2-en-1-yl)-1-(4-methoxyphenyl)ethan-1-one, **2-(cyclopent-3-en-1-yl)-1-(4-methoxyphenyl)ethan-1-one and (4-methoxyphenyl)(3-methylenecyclopentyl)methanone (ratio 1:1.3:1.4) 2t**: the title compounds were prepared following the general procedure using $Co(dmgH)_2(py)_2PF_6$ (12 mg, 0.02 mmol, 10 mol%), 9-mesityl-10-methylacridinium perchlorate (6.2 mg, 0.015 mmol, 7.5 mol%), collidine (48.5 mg (~52 µL), 0.4 mmol, 2 equiv.) and (1S,4R)-2-(4-methoxyphenyl)bicyclo[2.2.1]heptan-2-ol (43.7 mg, 0.2 mmol, 1 equiv.). The reaction mixture was stirred in DCE (2 mL) and irradiated with a 2 m blue LED strip at room temperature for 48 h. The crude product was purified by flash column chromatography to afford **2t** as a colorless oil in 84% yield (43.6 mg).

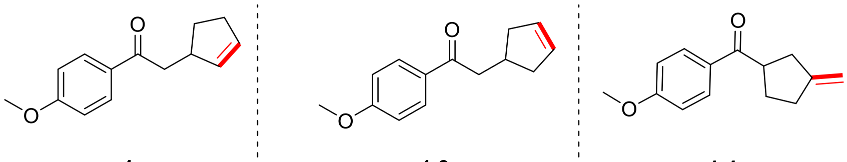

1 1.3 1.4

^{1}H NMR (400 MHz, $CDCl_3$): δ 8.00 – 7.85 (m, $2H_{isomers\ a,b,c}$), 6.98 – 6.85 (m, $2H_{isomers\ a,b,c}$), 5.80 – 5.68 (m, $0.54H_{isomer\ a}$), 5.68 – 5.62 (m, $0.7H_{isomer\ b}$), 4.87 (s, $0.78H_{isomer\ c}$), 3.84 (s, $3H_{isomers\ a,b,c}$), 3.81 – 3.69 (m, $0.39H_{isomer\ c}$), 3.30 – 3.17 (m, $0.27H_{isomer\ a}$), 3.03 – 2.88 (m, $1.24H_{isomers\ a,b,c}$), 2.88 – 2.78 (m, $0.35H_{isomer\ b}$), 2.71 – 2.24 (m, $2.8H_{isomers\ a,b,c}$), 2.22 – 2.10 (m, $0.27H_{isomer\ a}$), 2.08 – 1.90 (m, $1.4H_{isomers\ a,b,c}$), 1.50 – 1.39 (m, $0.27H_{isomer\ a}$), (caculated according with isomers ratio a:b:c = 0.27:0.35:0.39).

^{13}C NMR (101 MHz, $CDCl_3$): δ 200.3 (s), 198.8 (s), 198.5 (s), 163.5 (s), 151.1 (s), 134.5 (d), 131.2 (d), 130.8 (d), 130.5 (d), 130.4 (d), 129.8 (d), 129.7 (s), 113.9 (d), 113.8 (d), 105.8 (t), 55.6 (q), 46.5 (d), 45.0 (t), 44.5 (t), 41.7 (d), 39.2 (t), 36.7 (t), 33.3 (d), 32.6 (t), 32.0 (t), 30.4 (t), 30.1 (t).

IR (ATR) ν 3341, 2940, 2841, 1670, 1595, 1509, 1421, 1362, 1311, 1251, 1168, 1024, 831 cm^{-1};

HRMS (ESI) for $C_{14}H_{16}O_2Na^+$ $(M+Na)^+$: 239.1043; Found : 239.1041.

(3S,8S,9S,10R,13R,14S)-10,13-dimethyl-2,3,4,7,8,9,10,11,12,13,14,15-dodecahydro-1H-cyclopenta[a]phenanthren-3-ol, 2u:[19] the title compounds were prepared following the general procedure using $Co(dmgH)_2(py)_2PF_6$ (6 mg, 0.01 mmol, 5 mol%), $[Ir(dF(CF_3)ppy)_2(5,5'd(CF_3)bpy)](PF_6)$ (2.29 mg, 0.002 mmol, 1 mol%), collidine (48.5 mg (~52 µL), 0.4 mmol, 2 equiv.) and (3S,8S,9S,10R,13S,14S)-17-(1-hydroxy-1-(4-methoxyphenyl)ethyl)-10,13-dimethyl-2,3,4,7,8,9,10,11,12,13,14,15,16,17-tetradecahydro-1H-cyclopenta[a]phenanthren-3-ol (84.9 mg, 0.2 mmol, 1 equiv.). The reaction mixture was stirred in DCE (2 mL) and irradiated with a

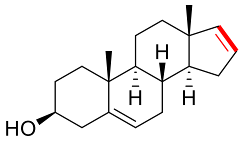

2 m blue LED strip at room temperature for 48 h. The crude product was purified by flash column chromatography to afford **2u** as a white solid in 94% yield (51.4 mg). known compound

^{1}H NMR (400 MHz, $CDCl_3$): δ 5.84 (d, J = 5.6 Hz, 1H), 5.71 (d, J = 3.2 Hz, 1H), 5.36 (d, J = 4.9 Hz, 1H), 3.61 – 3.45 (m, 1H), 2.36 – 2.18 (m, 2H), 2.11 (ddd, J = 15.1, 6.9, 2.9 Hz, 1H), 2.02 (dt, J = 13.0, 4.7 Hz, 1H), 1.92 (t, J = 13.3 Hz, 1H), 1.87 – 1.73 (m, 3H), 1.71 – 1.47 (m, 6H), 1.46 – 1.36 (m, 1H), 1.36 – 1.24 (m, 1H), 1.15 – 0.97 (m, 5H), 0.79 (s, 3H).

^{13}C NMR (101 MHz, $CDCl_3$): δ 143.9 (d), 141.2 (s), 129.5 (d), 121.6 (d), 71.9 (d), 56.3 (d), 51.1 (d), 45.5 (s), 42.5 (t), 37.3 (t), 36.9 (s), 36.0 (t), 32.2 (t), 31.9 (t), 31.8 (t), 30.6 (d), 21.0 (t), 19.5 (q), 17.0 (q).

Mixture of 1-(4-methoxyphenyl)-3,7-dimethyloct-5-en-1-one and 1-(4-methoxyphenyl)-3,7-dimethyloct-6-en-1-one, **2v**: the title compounds were prepared following the general procedure using $Co(dmgH)_2(py)_2PF_6$ (6 mg, 0.01 mmol, 5 mol%), $[Ir(dF(CF_3)ppy)_2(5,5'd(CF_3)bpy)](PF_6)$ (2.29 mg, 0.002 mmol, 1 mol%), collidine (48.5 mg (~52 μL), 0.4 mmol, 2 equiv.) and (2S,5R)-2-isopropyl-1-(4-methoxyphenyl)-5-methylcyclohexan-1-ol (52.5 mg, 0.2 mmol, 1 equiv.). The reaction mixture was stirred in DCE (2 mL) and irradiated with a 2 m blue LED strip at room temperature for 72 h. The crude product was purified by flash column chromatography to afford **2v** as a colorless oil in 84% yield (43.6 mg).

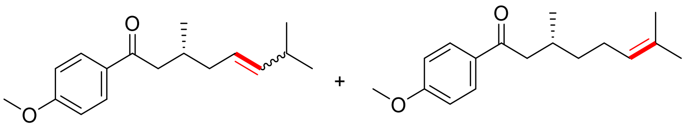

1.4 1

marjor ismer / minor isomer = 1.4:1 (major *E*/*Z* 6:1)

^{1}H NMR (400 MHz, $CDCl_3$): δ 7.93 (d, *J* = 9.0 Hz, $4.8H_{major+minor}$), 6.92 (d, *J* = 8.4 Hz, $4.8H_{major+minor}$), 5.45 – 5.30 (m, $1.2H_{major\ E}$), 5.30 – 5.20 (m, $0.2H_{major\ Z}$), 5.10 (t, *J* = 7.2 Hz, $1H_{minor}$), 3.86 (s, $7.2H_{major+minor}$), 3.00 – 2.84 (m, $2.4H_{major+minor}$), 2.74 – 2.57 (m, $2.4H_{major+minor}$), 2.31 – 1.91 (m, $7.2H_{major+minor}$), 1.67 (s, $3H_{minor}$), 1.60 (s, $3H_{minor}$), 1.46 – 1.10 (m, $4.8H_{major+minor}$), 1.02 – 0.90 (m, $12.6H_{major}$), 0.86 (d, *J* = 6.6 Hz, $3H_{minor}$).

^{13}C NMR (101 MHz, $CDCl_3$): δ 199.2, 199.1 (2C), 163.4 , 140.1, 139.3, 131.5, 130.7 (2C), 130.5 , 124.9, 124.6, 113.8, 55.6, 45.8, 45.7, 44.9, 40.2, 39.2, 37.6, 37.4, 31.2, 30.4, 30.2, 29.9, 28.1, 25.8, 25.7, 24.9, 22.8, 22.8, 22.7, 20.2, 20.1, 17.8, the multiplicities of ^{13}C NMR spectra were not determined due to the complexity.

IR (ATR) ν 3424, 2958, 1674, 1601, 1510, 1459, 1419, 1367, 1310, 1258, 1172, 1112, 1030, 974, 941, 831 cm^{-1};

HRMS (EI) for $C_{17}H_{24}O^+$ $(M)^+$: 260.1771; Found : 260.1761.

(*Z*)-heptadeca-1,8-diene, **2w**:[20] the title compounds were prepared following the general procedure using $Co(dmgH)_2(py)_2PF_6$ (6 mg, 0.01 mmol, 5

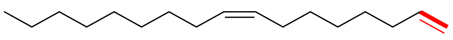

mol%), [Ir(dF(CF_3)ppy)$_2$(5,5′d(CF_3)bpy)](PF_6) (4.6 mg, 0.002 mmol, 2 mol%), collidine (48.5 mg (~52 μL), 0.4 mmol, 2 equiv.) and (*Z*)-1,1-bis(4-methoxyphenyl)octadec-9-en-1-ol (96.1 mg, 0.2 mmol, 1 equiv.). The reaction mixture was stirred in DCE (2 mL) and irradiated with a 2 m blue LED strip at room temperature for 72 h. The crude product was purified by flash column chromatography to afford **2w** as a colorless oil in 47% yield (22.0 mg).

^{1}H NMR (600 MHz, $CDCl_3$): 5.89 – 5.75 (m, 1H), 5.40 – 5.32 (m, 2H), 5.07 – 4.90 (m, 2H), 2.08 – 1.97 (m, 6H), 1.42 – 1.22 (m, 18H), 0.88 (t, *J* = 6.9 Hz, 3H).

^{13}C NMR (151 MHz, $CDCl_3$): δ 139.3 (d), 130.2 (d), 129.9 (d), 114.3 (t), 34.0 (t), 32.1 (t), 29.9 (t), 29.8 (t), 29.7 (t), 29.5 (t), 29.0 (t, 2C), 27.4 (t), 27.3 (t), 22.9 (t), 14.3 (q).

Mixture of 2-(2,2-dimethyl-3-methylenecyclopentyl)acetaldehyde (a) and 2-(2,2,3-trimethylcyclopent-3-en-1-yl)acetaldehyde (b) (isomer ratio a:b = 1:1.1) 2x:[21] the title compounds were prepared following the general procedure using Co(dmgH)$_2$(py)$_2$$PF_6$ (6 mg, 0.01 mmol, 5 mol%), [Ir(dF(CF_3)ppy)$_2$(5,5′d(CF_3)bpy)](PF_6) (6.87 mg, 0.006 mmol, 3 mol%), $PBu_4^+(PhO)_2POO^-$ (25.4 mg, 0.05 mmol, 25 mol%) and (-)-borneol (30.9 mg, 0.2 mmol, 1 equiv.). The reaction mixture was stirred in toluene (2 mL) and irradiated with a 2 m blue LED strip at room temperature for 72 h. The crude product was purified by flash column chromatography to afford **2x** as a colorless oil in 43% yield (13.2 mg).

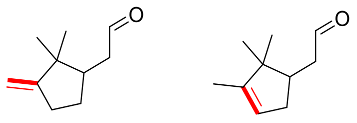

isomer a : b = 1:1.1

^{1}H NMR (600 MHz, $CDCl_3$): δ 9.80 (s, $2H_{isomers\ a,b}$), 5.24 (s, $1H_{isomer\ b}$), 4.80 (d, *J* = 14.1 Hz, $2H_{isomer\ a}$), 2.56 – 2.44 (m, 3H), 2.44 – 2.33 (m, 3H), 2.31 – 2.21 (m, 2H), 2.08 – 2.01 (m, 1H), 1.96 – 1.85 (m, 2H), 1.62 (d, *J* = 2.2 Hz, $3H_{isomer\ b}$), 1.38 – 1.29 (m, 1H), 1.07 (s, $3H_{isomer\ a}$), 1.00 (s, $3H_{isomer\ b}$), 0.84 (s, $3H_{isomer\ a}$), 0.79 (s, $3H_{isomer\ b}$).

^{13}C NMR (151 MHz, $CDCl_3$): δ 203.2 (d), 202.8 (d), 160.8 (s), 148.1 (s), 121.7 (d), 103.9 (t), 47.1 (s), 45.3 (t), 45.0 (t), 44.4 (d, 2C), 44.0 (s), 35.7 (t), 30.7 (t), 28.5 (t), 26.7 (q), 25.8 (q), 23.7 (q), 20.2 (q), 12.8 (q).

Ethene-1,1-diyldibenzene, **2y**:[22] the title compound was prepared following the general procedure using Co(dmgH)$_2$(py)$_2$$PF_6$ (3 mg, 0.005 mmol, 5 mol%), [Ir(dF(CF_3)ppy)$_2$(5,5′d(CF_3)bpy)](PF_6) (3.5 mg, 0.003 mmol, 3 mol%), $PBu_4^+(PhO)_2POO^-$ (40.7 mg, 0.04 mmol, 40 mol%) and 2,2-diphenylpropan-1-ol (21.2 mg, 0.1 mmol, 1 equiv.). The reaction mixture was stirred in $PhCF_3$ (2 mL) and irradiated with two Kessil 34 W lamps at room temperature for 72 h. The yield was determined to be 22% according to ^{1}H NMR analysis of the crude mixture using CH_2Br_2 as an internal standard.

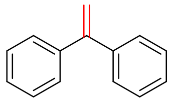

4,4-Diphenyl-2-(prop-1-en-2-yl)tetrahydrofuran, **2z**:[23] the title compound was prepared following the general procedure using Co(dmgH)2(py)2PF6 (3 mg, 0.005 mmol, 5 mol%), [Ir(dF(CF3)ppy)2(5,5′d(CF3)bpy)](PF6) (3.5 mg, 0.003 mmol, 3 mol%), $PBu_4^+(PhO)_2POO^-$ (20.3 mg, 0.04 mmol, 40 mol%) and 5-methyl-2,2-diphenylhex-4-en-1-ol (26.7 mg, 0.1 mmol, 1 equiv.). The reaction mixture was stirred in $PhCF_3$ (2 mL) and irradiated with two Kessil 34 W lamps at room temperature for 72 h. The crude product was purified by flash column chromatography to afford **2z** as a colorless oil in 56% yield (14.9 mg).

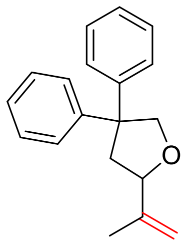

^{1}H NMR (600 MHz, $CDCl_3$): δ 7.35 (d, *J* = 7.3 Hz, 2H), 7.32 – 7.27 (m, 4H), 7.24 – 7.17 (m, 4H), 5.02 (s, 1H), 4.81 (s, 1H), 4.76 (d, *J* = 8.7 Hz, 1H), 4.41 (dd, *J* = 10.4, 5.9 Hz, 1H), 4.12 (d, *J* = 8.7 Hz, 1H), 2.61 (dd, *J* = 12.0, 5.3 Hz, 1H), 2.49 (dd, *J* = 12.1, 10.3 Hz, 1H), 1.74 (s, 3H).

^{13}C NMR (101 MHz, $CDCl_3$): δ 146.2, 145.7, 145.6, 128.6, 128.5, 127.3, 127.3, 126.7, 126.4, 110.5, 81.7, 77.5, 56.3, 44.1, 18.0.

1-(4-(*tert*-Butoxy)phenyl)cyclohexan-1-ol, **4a**: the title compound was prepared following the general procedure using Co(dmgH)2(py)2PF6 (12 mg, 0.02 mmol, 10 mol%), 9-mesityl-10-methylacridinium perchlorate (6.2 mg, 0.015 mmol, 7.5 mol%), collidine (48.5 mg (~52 µL), 0.4 mmol, 2 equiv.) and 1-(4-(*tert*-butoxy)phenyl)cyclohexan-1-ol (49.7 mg, 0.2 mmol, 1 equiv.). The reaction mixture was stirred in DCE (2 mL) and irradiated with a 2 m blue LED strip at room temperature for 72 h. The crude product was purified by flash column chromatography to afford **4a** as a colorless oil in 58% yield (28.5 mg).

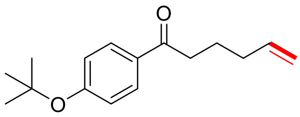

^{1}H NMR (600 MHz, $CDCl_3$): δ 7.89 (d, *J* = 8.7 Hz, 2H), 7.02 (d, *J* = 8.7 Hz, 2H), 5.89 – 5.75 (m, 1H), 5.13 – 4.95 (m, 2H), 2.93 (t, *J* = 7.4 Hz, 2H), 2.20 – 2.10 (m, 2H), 1.89 – 1.77 (m, 2H), 1.41 (s, 9H).

^{13}C NMR (151 MHz, $CDCl_3$): δ 199.3 (s), 160.3 (s), 138.3 (d), 131.8 (s), 129.6 (t), 122.6 (t), 115.4 (t), 79.7 (s), 37.6 (t), 33.4 (t), 29.1 (q), 23.6 (t).

IR (ATR) ν 2976, 2934, 2316, 2088, 1677, 1595, 1503, 1365, 1307, 1243, 1157, 990, 903, 843 cm^{-1};

HRMS (ESI) for $C_{16}H_{22}O_2Na^+$ $(M+Na)^+$: 269.1512; Found : 269.1512.

1-([1,1'-biphenyl]-4-yl)hex-5-en-1-one, **4b**: the title compound was prepared following the general procedure using Co(dmgH)2(py)2PF6 (12 mg, 0.02 mmol, 10 mol%), 9-mesityl-10-methylacridinium perchlorate (6.2 mg, 0.015 mmol, 7.5 mol%), collidine (48.5 mg (~52 µL), 0.4 mmol, 2 equiv.) and 1-([1,1'-biphenyl]-4-yl)cyclohexan-1-ol (50.5 mg, 0.2 mmol, 1 equiv.). The reaction mixture was stirred in DCE (2 mL) and irradiated with a 2 m blue LED strip at room temperature for 48 h.

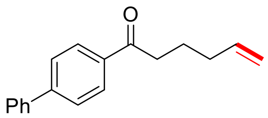

The crude product was purified by flash column chromatography to afford **4b** as a white solid in 74% yield (36.8 mg).

^{1}H NMR (400 MHz, CDCl$_3$): δ 8.04 (d, *J* = 8.4 Hz, 2H), 7.68 (d, *J* = 8.4 Hz, 2H), 7.63 (d, *J* = 7.0 Hz, 2H), 7.48 (t, *J* = 7.4 Hz, 2H), 7.44 – 7.37 (m, 1H), 5.85 (ddt, *J* = 16.9, 10.1, 6.7 Hz, 1H), 5.19 – 4.95 (m, 2H), 3.01 (t, *J* = 7.4 Hz, 2H), 2.26 – 2.14 (m, 2H), 1.97 – 1.84 (m, 2H);

^{13}C NMR (101 MHz, CDCl$_3$): δ 199.9 (s), 145.7 (s), 140.0 (s), 138.2 (d), 135.9 (s), 129.1 (d), 128.8 (d), 128.3 (d), 127.4 (d), 127.3 (d), 115.4 (t), 37.9 (t), 33.4 (t), 23.5 (t);

IR (ATR) ν 3071, 2934, 2883, 2324, 2088, 1679, 1599, 1452, 1400, 1337, 1256, 1190, 982, 905, 825, 745 cm^{-1};

HRMS (ESI) for $C_{18}H_{18}ONa^+$ $(M+Na)^+$: 273.1250; Found : 273.1250.

1-(4-Phenoxyphenyl)hex-5-en-1-one, **4c**: the title compound was prepared following the

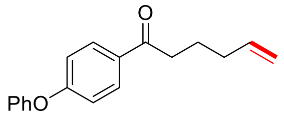

general procedure using Co(dmgH)$_2$(py)$_2$PF$_6$ (12 mg, 0.02 mmol, 10 mol%), 9-mesityl-10-methylacridinium perchlorate (6.2 mg, 0.015 mmol, 7.5 mol%), collidine (48.5 mg (~52 μL), 0.4 mmol, 2 equiv.) and 1-(4-phenoxyphenyl)cyclohexan-1-ol (53.7 mg, 0.2 mmol, 1 equiv.). The reaction mixture was stirred in DCE (2 mL) and irradiated with a 2 m blue LED strip at room temperature for 48 h. The crude product was purified by flash column chromatography to afford **4c** as a white solid in 67% yield (35.8 mg).

^{1}H NMR (600 MHz, CDCl$_3$): δ 7.94 (d, *J* = 8.8 Hz, 2H), 7.43 – 7.36 (m, 2H), 7.20 (td, *J* = 7.4, 1.1 Hz, 1H), 7.07 (dd, *J* = 8.6, 1.2 Hz, 2H), 7.00 (d, *J* = 8.8 Hz, 2H), 5.82 (ddt, *J* = 17.0, 10.3, 6.7 Hz, 1H), 5.08 – 4.96 (m, 2H), 2.93 (t, *J* = 7.4 Hz, 2H), 2.19 – 2.12 (m, 2H), 1.88 – 1.81 (m, 2H).

^{13}C NMR (151 MHz, CDCl$_3$): δ 199.0 (s), 161.9 (s), 155.6 (s), 138.2 (d), 131.9 (s), 130.4 (d), 130.2 (d), 124.7 (d), 120.3 (d), 117.4 (d), 115.4 (t), 37.6 (t), 33.4 (t), 23.6 (t).

IR (ATR) ν 3062, 1670, 1579, 1486, 1416, 1365, 1335, 1241, 1199, 1163, 977, 912, 863, 823, 743 cm^{-1};

HRMS (ESI) for $C_{18}H_{18}O_2Na^+$ $(M+Na)^+$: 289.1199; Found : 2289.1199.

1-(4-((tert-butyldimethylsilyl)oxy)phenyl)hex-5-en-1-one, **4d**: the title compound was

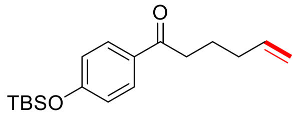

prepared following the general procedure using Co(dmgH)$_2$(py)$_2$PF$_6$ (12 mg, 0.02 mmol, 10 mol%), 9-mesityl-10-methylacridinium perchlorate (6.2 mg, 0.015 mmol, 7.5 mol%), collidine (48.5 mg (~52 μL), 0.4 mmol, 2 equiv.) and 1-(4-((tert-butyldimethylsilyl)oxy)phenyl)cyclohexan-1-ol (61.3 mg, 0.2 mmol, 1 equiv.). The reaction mixture was stirred in DCE (2 mL) and irradiated with a 2 m blue LED strip at room temperature for 48 h. The crude product was purified by flash column chromatography to afford **4d** as a white solid in 59% yield (35.8 mg).

^{1}H NMR (600 MHz, CDCl$_3$): δ 7.88 (d, *J* = 8.7 Hz, 2H), 6.86 (d, *J* = 8.7 Hz, 2H), 5.82 (ddt, *J* = 17.0, 10.3, 6.7 Hz, 1H), 5.07 – 4.95 (m, 2H), 2.92 (t, *J* = 7.4 Hz, 2H), 2.17 – 2.10 (m, 2H), 1.86 – 1.79 (m, 2H), 0.98 (s, 9H), 0.23 (s, 6H).

^{13}C NMR (151 MHz, CDCl$_3$): δ 199.2 (s), 160.2 (s), 138.3 (d), 130.8 (s), 130.3 (d), 120.0 (d), 115.3 (t), 37.5 (t), 33.4 (t), 25.7 (q), 23.7 (t), 18.4 (s), -4.2 (q).

IR (ATR) ν 2939, 2863, 1678, 1594, 1508, 1261, 1163, 1106, 994, 903, 829 cm^{-1};

HRMS (ESI) for $C_{18}H_{28}O_2NaSi^+$ $(M+Na)^+$: 327.1751; Found : 327.1751.

1-(3-fluoro-4-methoxyphenyl)hex-5-en-1-one, **4e**: the title compound was prepared following the general procedure using Co(dmgH)$_2$(py)$_2$PF$_6$ (12 mg, 0.02 mmol, 10 mol%), 9-mesityl-10-methylacridinium perchlorate (6.2 mg, 0.015 mmol, 7.5 mol%), collidine (48.5 mg (~52 μL), 0.4 mmol, 2 equiv.) and 1-(3-fluoro-4-methoxyphenyl)cyclohexan-1-ol (44.9 mg, 0.2 mmol, 1 equiv.). The reaction mixture was stirred in DCE (2 mL) and irradiated with a 2 m blue LED strip at room temperature for 48 h. The crude product was purified by flash column chromatography to afford **4e** as a colorless oil in 72% yield (31.8 mg).

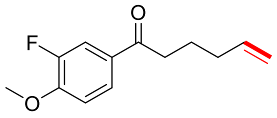

^{1}H NMR (600 MHz, CDCl$_3$): δ 7.77 – 7.71 (m, 1H), 7.71 – 7.64 (m, 1H), 6.98 (t, *J* = 8.3 Hz, 1H), 5.86 – 5.76 (m, 1H), 5.07 – 4.95 (m, 2H), 3.94 (s, 3H), 2.89 (t, *J* = 7.4 Hz, 2H), 2.18 – 2.10 (m, 2H), 1.87 – 1.77 (m, 2H).

^{13}C NMR (151 MHz, CDCl$_3$): δ 198.1 (s), 152.1 (s; d, $J_{C\text{-}F}$ = 248.1 Hz), 151.8 (s; d, $J_{C\text{-}F}$ = 11 Hz), 138.1 (d), 130.5 (s; d, $J_{C\text{-}F}$ = 4.7 Hz), 125.4 (d; d, $J_{C\text{-}F}$ = 3.3 Hz), 115.8 (d; d, $J_{C\text{-}F}$ = 19.2 Hz), 115.5 (t), 112.4 (d), 56.4 (q), 37.5 (t), 33.3 (t), 23.5 (t).

^{19}F NMR (564 MHz, CDCl$_3$): δ -134.4.

IR (ATR) ν 3078, 2933, 1673, 1610, 1518, 1432, 1323, 1271, 1222, 1130, 1009, 904, 809, 754 cm^{-1};

HRMS (ESI) for $C_{13}H_{15}O_2FNa^+$ $(M+Na)^+$: 245.0948; Found : 245.0949.

1-(Phenanthren-9-yl)hex-5-en-1-one, **4f-1**: the title compound was prepared following the general procedure using Co(dmgH)$_2$(py)$_2$PF$_6$ (12 mg, 0.01 mmol, 10 mol%), 9-mesityl-10-methylacridinium perchlorate (6.2 mg, 0.015 mmol, 7.5 mol%), collidine (48.5 mg (~52 μL), 0.4 mmol, 2 equiv.) and 1-(phenanthren-9-yl)cyclohexan-1-oll (55.3 mg, 0.2 mmol, 1 equiv.). The reaction mixture was stirred in DCE (2 mL) and irradiated with a 2 m blue LED strip at room temperature for 72 h. The crude product was purified by flash column chromatography to afford **4f-1** as a colorless oil in 42% yield (23.1 mg).

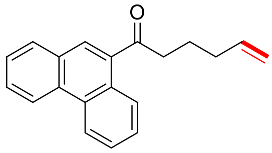

^{1}H NMR (400 MHz, CDCl$_3$): δ 8.73 (d, *J* = 8.4 Hz, 1H), 8.69 (d, *J* = 8.3 Hz, 1H), 8.51 (dd, *J* = 8.3, 1.5 Hz, 1H), 8.08 (s, 1H), 7.95 (d, *J* = 8.1 Hz, 1H), 7.78 – 7.61 (m, 4H), 5.96 – 5.75 (m, 1H), 5.15 – 4.96 (m,

2H), 3.14 (t, $J = 7.4$ Hz, 2H), 2.28 – 2.18 (m, 2H), 2.01 – 1.91 (m, 2H).

^{13}C NMR (101 MHz, CDCl$_3$): δ 205.0 (s), 138.1 (d), 135.8 (s), 131.8 (s), 130.9 (s), 130.2 (s), 129.8 (d), 129.0 (d), 128.8 (d), 128.5 (s), 127.6 (d), 127.2 (d), 126.7 (d), 123.0 (d), 122.8 (d), 115.6 (t), 41.7 (t), 33.4 (t), 23.9 (t).

IR (ATR) ν 3071, 2929, 1678, 1528, 1493, 1445, 1401, 1353, 1294, 1246, 1215, 993, 905, 855, 744 cm^{-1};

HRMS (ESI) for $C_{20}H_{18}ONa^+$ $(M+Na)^+$: 297.1250; Found : 297.1247.

(*E*)-1-(Phenanthren-9-yl)hex-2-en-1-one, **4f-2**: the title compound was prepared following the

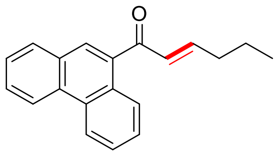

general procedure using $Co(dmgH)_2(py)_2PF_6$ (12 mg, 0.01 mmol, 10 mol%), 9-mesityl-10-methylacridinium perchlorate (6.2 mg, 0.015 mmol, 7.5 mol%), collidine (48.5 mg (~52 μL), 0.4 mmol, 2 equiv.) and 1-(phenanthren-9-yl)cyclohexan-1-oll (55.3 mg, 0.2 mmol, 1 equiv.). The reaction mixture was stirred in DCE (2 mL) and irradiated with a 2 m blue LED strip at room temperature for 72 h. The crude product was purified by flash column chromatography to afford **4f-2** as a colorless oil in 10% yield (5.3 mg).

^{1}H NMR (600 MHz, CDCl$_3$): δ 8.74 (d, $J = 8.3$ Hz, 1H), 8.70 (d, $J = 8.4$ Hz, 1H), 8.24 (d, $J = 8.2$ Hz, 1H), 7.93 (d, $J = 8.1$ Hz, 1H), 7.91 (s, 1H), 7.74 (t, $J = 7.8$ Hz, 1H), 7.69 (t, $J = 7.4$ Hz, 1H), 7.66 – 7.60 (m, 2H), 6.97 – 6.87 (m, 1H), 6.70 (dd, $J = 15.7$, 1.3 Hz, 1H), 2.33 – 2.25 (m, 2H), 1.53 (q, $J = 7.5$ Hz, 2H), 0.96 (t, $J = 7.3$ Hz, 3H).

^{13}C NMR (151 MHz, CDCl$_3$): δ 196.6 (s), 152.2 (d), 136.1 (d), 131.5 (s), 130.8 (s), 130.3 (d), 129.6 (s), 129.1 (d), 128.6 (d), 128.4 (d), 127.3 (d), 127.2 (d), 126.7 (d), 123.0 (d), 122.8 (d), 34.9 (t), 21.5 (t), 13.9 (t).

IR (ATR) ν 3063, 2957, 2929, 2869, 2335, 1650, 1528, 1447, 1307, 1250, 1112, 1044, 978, 890, 734 cm^{-1};

HRMS (ESI) for $C_{20}H_{18}ONa^+$ $(M+Na)^+$: 297.1250; Found : 297.1252.

1-(naphthalen-2-yl)hex-5-en-1-one, **4g**:[24] the title compound was prepared following the

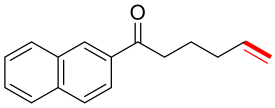

general procedure using $Co(dmgH)_2(py)_2PF_6$ (6 mg, 0.01 mmol, 5 mol%), 9-mesityl-10-methylacridinium perchlorate (6.2 mg, 0.015 mmol, 7.5 mol%), collidine (48.5 mg (~52 μL), 0.4 mmol, 2 equiv.) and 1-(naphthalen-2-yl)cyclohexan-1-ol (45.3 mg, 0.2 mmol, 1 equiv.). The reaction mixture was stirred in DCE (2 mL) and irradiated with a 2 m blue LED strip at room temperature for 72 h. The crude product was purified by flash column chromatography to afford **4g** as a colorless solid in 47% yield (21.1 mg).

^{1}H NMR (400 MHz, CDCl$_3$): δ 8.47 (s, 1H), 8.03 (dd, $J = 8.6$, 1.8 Hz, 1H), 7.97 (d, $J = 8.1$ Hz, 1H), 7.93 – 7.84 (m, 2H), 7.65 – 7.51 (m, 2H), 5.86 (ddt, $J = 16.9$, 10.2, 6.7 Hz, 1H), 5.15 – 4.97 (m, 2H), 3.11 (t, $J = 7.3$ Hz, 2H), 2.27 – 2.16 (m, 2H), 1.99 – 1.86 (m, 2H).

^{13}C NMR (101 MHz, CDCl$_3$): δ 200.3 (s), 138.2 (d), 135.7 (s), 134.5 (s), 132.7 (s), 129.7 (d, 2C), 128.5 (d, 2C), 127.9 (d), 126.8 (d), 124.0 (d), 115.5 (t), 37.9 (t), 33.4 (t), 23.6 (t).

1-(benzo[b]thiophen-2-yl)hex-5-en-1-one, **4h**:[24] the title compound was prepared following the general procedure using Co(dmgH)$_2$(py)$_2$PF$_6$ (6 mg, 0.01 mmol, 5 mol%), 9-mesityl-10-methylacridinium perchlorate (6.2 mg, 0.015 mmol, 7.5 mol%), collidine (48.5 mg (~52 μL), 0.4 mmol, 2 equiv.) and 1-(benzo[b]thiophen-2-yl)cyclohexan-1-ol (46.5 mg, 0.2 mmol, 1 equiv.). The reaction mixture was stirred in DCE (2 mL) and irradiated with a 2 m blue LED strip at room temperature for 72 h. The crude product was purified by flash column chromatography to afford **4h** as a white solid in 63% yield (28.9 mg).

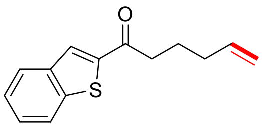

^{1}H NMR (400 MHz, CDCl$_3$): δ 7.95 (s, 1H), 7.88 (t, *J* = 7.6 Hz, 2H), 7.51 – 7.37 (m, 2H), 5.83 (ddt, *J* = 16.9, 10.1, 6.7 Hz, 1H), 5.14 – 4.96 (m, 2H), 3.02 (t, *J* = 7.4 Hz, 2H), 2.25 – 2.14 (m, 2H), 1.98 – 1.85 (m, 2H).

^{13}C NMR (101 MHz, CDCl$_3$): δ 194.8 (s), 144.0 (s), 142.6 (s), 139.3 (s), 138.0 (d), 128.9 (d), 127.4 (d), 126.0 (d), 125.1 (d), 123.1 (d), 115.6 (t), 38.5 (t), 33.3 (t), 23.8 (t).

1-(Benzofuran-2-yl)hex-5-en-1-one, **4i**: the title compound was prepared following the general procedure using Co(dmgH)$_2$(py)$_2$PF$_6$ (6 mg, 0.01 mmol, 5 mol%), 9-mesityl-10-methylacridinium perchlorate (6.2 mg, 0.015 mmol, 7.5 mol%), collidine (48.5 mg (~52 μL), 0.4 mmol, 2 equiv.) and 1-(benzofuran-2-yl)cyclohexan-1-ol (43.3 mg, 0.2 mmol, 1 equiv.). The reaction mixture was stirred in DCE (2 mL) and irradiated with a 2 m blue LED strip at room temperature for 72 h. The crude product was purified by flash column chromatography to afford **4i** as a colorless oil in 43% yield (18.6 mg).

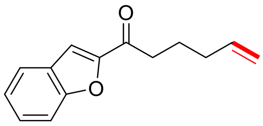

^{1}H NMR (400 MHz, CDCl$_3$): δ 7.71 (d, *J* = 7.9 Hz, 1H), 7.58 (d, *J* = 8.3 Hz, 1H), 7.50 (s, 1H), 7.47 (t, *J* = 7.9 Hz, 1H), 7.31 (t, *J* = 7.5 Hz, 1H), 5.83 (ddt, *J* = 16.8, 10.2, 6.7 Hz, 1H), 5.14 – 4.94 (m, 2H), 2.97 (t, *J* = 7.4 Hz, 2H), 2.25 – 2.14 (m, 2H), 1.96 – 1.84 (m, 2H).

^{13}C NMR (101 MHz, CDCl$_3$): δ 191.5 (s), 155.7 (s), 152.8 (s), 138.0 (d), 128.3 (d), 127.2 (s), 124.0 (d), 123.4 (d), 115.6 (t), 112.7 (d), 112.6 (d), 38.2 (t), 33.3 (t), 23.4 (t).

IR (ATR) ν 3069, 2925, 2860, 1684, 1557, 1455, 1375, 1260, 1140, 993, 911, 877, 829, 744 cm^{-1};

HRMS (ESI) for $C_{14}H_{14}O_2Na^+$ $(M+Na)^+$: 237.0886; Found : 237.0887.

2-Methoxynaphthalene, **6a**:[25] the title compound was prepared following the general procedure

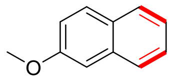

using $Co(dmgH)_2(py)_2PF_6$ (6 mg, 0.01 mmol, 5 mol%), $[Ir(dF(CF_3)ppy)_2(5,5'd(CF_3)bpy)](PF_6)$ (2.3 mg, 0.002 mmol, 1 mol%), collidine (24.2 mg (~26 μL), 0.2 mmol, 1 equiv.) and 6-methoxy-1,2,3,4-tetrahydronaphthalene (32.4 mg, 0.2 mmol, 1 equiv.). The reaction mixture was stirred in DCE (1 mL) and irradiated with a 2 m blue LED strip at room temperature for 36 h. The crude product was purified by flash column chromatography to afford **6a** as a colorless oil in 89% yield (28.3 mg).

^{1}H NMR (600 MHz, $CDCl_3$): δ 7.79 (d, J = 8.2 Hz, 1H), 7.76 (d, J = 8.5 Hz, 2H), 7.49 – 7.43 (m, 1H), 7.35 (t, J = 7.7 Hz, 1H), 7.20 – 7.14 (m, 2H), 3.94 (s, 3H).

^{13}C NMR (151 MHz, $CDCl_3$): δ 157.7 (s), 134.7 (s), 129.5(d), 129.1(s), 127.8 (d), 126.9 (d), 126.5 (d), 123.7 (d), 118.8 (d), 105.9 (d), 55.4 (q).

Naphthalene, **6b**:[26] the title compound was prepared following the general procedure using

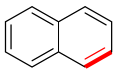

$Co(dmgH)_2(py)_2PF_6$ (6 mg, 0.01 mmol, 5 mol%), $[Ir(dF(CF_3)ppy)_2(5,5'd(CF_3)bpy)](PF_6)$ (2.3 mg, 0.002 mmol, 1 mol%), collidine (24.2 mg (~26 μL), 0.2 mmol, 1 equiv.) and 1,2-dihydronaphthalene (26 mg, 0.2 mmol, 1 equiv.). The reaction mixture was stirred in DCE (1 mL) and irradiated with a 2 m blue LED strip at room temperature for 36 h. The crude product was purified by flash column chromatography to afford **6b** as a colorless oil in 77% yield.

^{1}H NMR (600 MHz, $CDCl_3$): δ 7.91 – 7.85 (m, 1H), 7.54 – 7.49 (m, 1H).

^{13}C NMR (151 MHz, $CDCl_3$): δ 133.6 (s), 128.0 (d), 126.0 (d).

Benzofuran, **6c**:[25] the title compound was prepared following the general procedure using

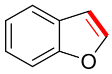

$Co(dmgH)_2(py)_2PF_6$ (6 mg, 0.01 mmol, 5 mol%), $[Ir(dF(CF_3)ppy)_2(5,5'd(CF_3)bpy)](PF_6)$ (2.3 mg, 0.002 mmol, 1 mol%), collidine (24.2 mg (~26 μL), 0.2 mmol, 1 equiv.) and 2,3-dihydrobenzofuran (24 mg, 0.2 mmol, 1 equiv.). The reaction mixture was stirred in DCE (1 mL) and irradiated with a 2 m blue LED strip at room temperature for 36 h. The crude product was purified by flash column chromatography to afford **6c** as a colorless oil in 74% yield.

^{1}H NMR (400 MHz, $CDCl_3$): δ 7.68 – 7.57 (m, 2H), 7.53 (d, J = 8.1 Hz, 1H), 7.31 (t, J = 7.7 Hz, 1H), 7.26 (t, J = 7.4 Hz, 1H), 6.79 (s, 1H).

1-methylnaphthalene, **6d**:[27] the title compound was prepared following the general procedure

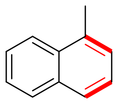

using $Co(dmgH)_2(py)_2PF_6$ (6 mg, 0.01 mmol, 5 mol%), $[Ir(dF(CF_3)ppy)_2(5,5'd(CF_3)bpy)](PF_6)$ (2.3 mg, 0.002 mmol, 1 mol%), collidine (24.2 mg (~26 μL), 0.2 mmol, 1 equiv.) and 1-methylene-1,2,3,4-tetrahydronaphthalene (28.8 mg, 0.2 mmol, 1 equiv.). The reaction mixture was stirred in DCE (1 mL) and irradiated with a 2 m blue LED strip at room temperature for 36 h. The crude product was purified by flash column chromatography to afford **6d** as a colorless oil in 99% yield.

^{1}H NMR (400 MHz, $CDCl_3$): δ 8.04 (d, J = 8.0 Hz, 1H), 7.93 – 7.84 (m, 1H), 7.75 (d, J = 8.0 Hz, 1H), 7.61 – 7.49 (m, 2H), 7.42 (t, J = 7.6 Hz, 1H), 7.36 (d, J = 6.9 Hz, 1H), 2.74 (s, 3H).

^{13}C NMR (101 MHz, $CDCl_3$): δ 134.4 (s), 133.7 (s), 132.7 (s), 128.6 (d), 126.7 (d), 126.5 (d), 125.8 (d), 125.7 (d, 2C), 124.2 (d), 19.5 (q).

4-Methoxybenzaldehyde, **6e**:[28] the title compound was prepared following the general

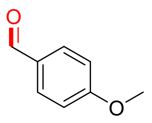

procedure using $Co(dmgH)_2(py)_2PF_6$ (6 mg, 0.01 mmol, 5 mol%), $[Ir(dF(CF_3)ppy)_2(5,5'd(CF_3)bpy)](PF_6)$ (2.3 mg, 0.002 mmol, 1 mol%), collidine (24.2 mg (~26 μL), 0.2 mmol, 1 equiv.) and (4-methoxyphenyl)methanol (27.6 mg, 0.2 mmol, 1 equiv.). The reaction mixture was stirred in DCE (1 mL) and irradiated with a 2 m blue LED strip at room temperature for 36 h. The crude product was purified by flash column chromatography to afford **6f** as a colorless oil in 93% yield.

^{1}H NMR (400 MHz, $CDCl_3$): δ 9.88 (s, 1H), 7.83 (d, J = 8.8 Hz, 2H), 7.00 (d, J = 8.7 Hz, 2H), 3.88 (s, 3H).

^{13}C NMR (101 MHz, $CDCl_3$): δ 190.9 (d), 164.7 (s), 132.1 (d), 130.1 (s), 114.4 (d), 55.7 (q).

1-(4-methoxyphenyl)ethan-1-one, **6f**:[29] the title compound was prepared following the general

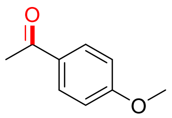

procedure using $Co(dmgH)_2(py)_2PF_6$ (6 mg, 0.01 mmol, 5 mol%), $[Ir(dF(CF_3)ppy)_2(5,5'd(CF_3)bpy)](PF_6)$ (2.3 mg, 0.002 mmol, 1 mol%), collidine (24.2 mg (~26 μL), 0.2 mmol, 1 equiv.) and 1-(4-methoxyphenyl)ethan-1-ol (30.4 mg, 0.2 mmol, 1 equiv.). The reaction mixture was stirred in DCE (1 mL) and irradiated with a 2 m blue LED strip at room temperature for 36 h. The crude product was purified by flash column chromatography to afford **6f** as a colorless oil in 97% yield (29.1 mg).

^{1}H NMR (600 MHz, $CDCl_3$): δ 7.93 (d, J = 8.8 Hz, 2H), 6.92 (d, J = 8.8 Hz, 2H), 3.86 (s, 3H), 2.55 (s, 3H).

^{13}C NMR (151 MHz, $CDCl_3$): δ 196.9 (s), 163.6 (s), 130.7 (d), 130.5 (s), 113.8 (d), 55.6 (q), 26.5 (q).

***tert*-Butyldimethyl(naphthalen-1-yloxy)silane**, **6g**:[30] the title compound was prepared

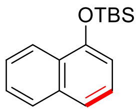

following the general procedure using $Co(dmgH)_2(py)_2PF_6$ (6 mg, 0.01 mmol, 5 mol%), $[Ir(dF(CF_3)ppy)_2(5,5'd(CF_3)bpy)](PF_6)$ (2.3 mg, 0.002 mmol, 1 mol%), collidine (24.2 mg (~26 μL), 0.2 mmol, 1 equiv.) and *tert*-butyl((3,4-dihydronaphthalen-1-yl)oxy)dimethylsilane (52.1 mg, 0.2 mmol, 1 equiv.). The reaction mixture was stirred in DCE (1 mL) and irradiated with a 2 m blue LED strip at room temperature for 36 h. The crude product was purified by flash column chromatography to afford **6g** as a colorless oil in 84% yield (43.6 mg).

^{1}H NMR (600 MHz, $CDCl_3$): δ 8.29 – 8.14 (m, 1H), 7.89 – 7.76 (m, 1H), 7.49 (dd, *J* = 9.0, 5.6 Hz, 3H), 7.35 (t, *J* = 7.8 Hz, 1H), 6.90 (d, *J* = 7.5 Hz, 1H), 1.14 (s, 9H), 0.33 (s, 6H).

^{13}C NMR (151 MHz, $CDCl_3$): δ 151.8, 135.2, 128.1, 127.7, 126.3, 126.0, 125.2, 122.7, 121.0, 112.7, 26.1, 18.6, -4.1.

***tert*-Butyldimethyl(phenoxy)silane**, **6h**:[31] the title compound was prepared following the

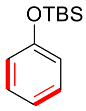

general procedure using $Co(dmgH)_2(py)_2PF_6$ (6 mg, 0.01 mmol, 5 mol%), $[Ir(dF(CF_3)ppy)_2(5,5'd(CF_3)bpy)](PF_6)$ (2.3 mg, 0.002 mmol, 1 mol%), collidine (24.2 mg (~26 μL), 0.2 mmol, 1 equiv.) and *tert*-butyl(cyclohex-1-en-1-yloxy)dimethylsilane (42.5 mg, 0.2 mmol, 1 equiv.). The reaction mixture was stirred in DCE (1 mL) and irradiated with a 2 m blue LED strip at room temperature for 36 h. The crude product was purified by flash column chromatography to afford **6h** as a colorless oil in 51% yield (21.2 mg).

^{1}H NMR (600 MHz, $CDCl_3$): δ 7.23 (t, *J* = 7.9 Hz, 2H), 6.95 (t, *J* = 7.4 Hz, 1H), 6.84 (d, *J* = 8.0 Hz, 2H), 0.99 (s, 9H), 0.20 (s, 6H).

^{13}C NMR (151 MHz, $CDCl_3$): δ 155.8, 129.5, 121.4, 120.3, 25.8, 18.4, -4.3.

1-(4-methoxyphenyl)ethan-1-one, **6h**:[32] the title compound was prepared following the general

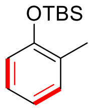

procedure using $Co(dmgH)_2(py)_2PF_6$ (6 mg, 0.01 mmol, 5 mol%), $[Ir(dF(CF_3)ppy)_2(5,5'd(CF_3)bpy)](PF_6)$ (2.3 mg, 0.002 mmol, 1 mol%), collidine (24.2 mg (~26 μL), 0.2 mmol, 1 equiv.) and *tert*-butyldimethyl((2-methylcyclohex-1-en-1-yl)oxy)silane (45.3 mg, 0.2 mmol, 1 equiv.). The reaction mixture was stirred in DCE (1 mL) and irradiated with a 2 m blue LED strip at room temperature for 36 h. The crude product was purified by flash column chromatography to afford **6h** as a colorless oil in 72% yield (31.8 mg).

^{1}H NMR (600 MHz, $CDCl_3$): δ 7.17 (d, *J* = 7.3 Hz, 1H), 7.10 (t, *J* = 7.6 Hz, 1H), 6.89 (t, *J* = 7.4 Hz, 1H), 6.81 (d, *J* = 8.0 Hz, 1H), 2.26 (s, 3H), 1.07 (s, 9H), 0.26 (s, 6H).

^{13}C NMR (151 MHz, $CDCl_3$): δ 154.0, 131.1, 129.1, 126.7, 121.1, 118.6, 25.9, 18.4, 17.0, -4.0.

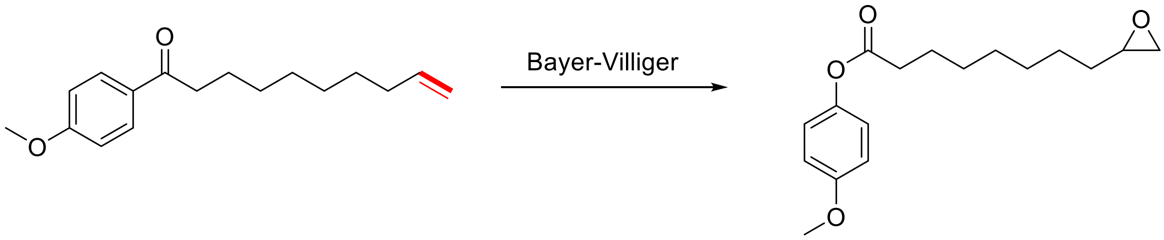

4-Methoxyphenyl 8-(oxiran-2-yl)octanoate, **7**: to a solution of 1-(4-methoxyphenyl)dec-9-en-1-one (0.05 mmol, 13 mg) in DCM (1.5 mL) was added disodium hydrogen phosphate (0.125 mmol, 30.8 mg) and *m*CPBA (0.125 mmol, 31 mg (70%)), the reaction mixture was stirred at room temperature until complete consumption of starting material. The reaction was quenched by adding saturated aq. sodium hydrogen carbonate and dried over sodium sulfate. The crude product was purified by flash column chromatography to afford **7** as a colorless oil in 91% yield (13.3 mg).

^{1}H NMR (400 MHz, CDCl$_3$): δ 6.97 (d, *J* = 9.0 Hz, 2H), 6.86 (d, *J* = 9.0 Hz, 2H), 3.77 (s, 3H), 2.92 – 2.85 (m, 1H), 2.73 (t, *J* = 4.5 Hz, 1H), 2.51 (t, *J* = 7.5 Hz, 2H), 2.44 (dd, *J* = 5.1, 2.7 Hz, 1H), 1.77 – 1.68 (m, 2H), 1.56 – 1.49 (m, 2H), 1.48 – 1.33 (m, 8H).

^{13}C NMR (101 MHz, CDCl$_3$): δ 172.6, 157.1, 144.2, 122.3, 114.4, 55.5, 52.3, 47.1, 34.3, 32.4, 29.2, 29.1, 29.0, 25.9, 24.9.

IR (ATR) ν 2929, 2856, 1754, 1678, 1600, 1504, 1461, 1366, 1298, 1246, 1191, 1135, 1032, 910, 835, 752 cm^{-1};

HRMS (ESI) for $C_{18}H_{18}O_2Na^+$ $(M+Na)^+$: 315.1567; Found : 315.1568.

nona-1,8-diene, **9**:[33] the title compounds were prepared following the general procedure using

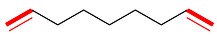

Co(dmgH)$_2$(py)$_2$PF$_6$ (6 mg, 0.01 mmol, 5 mol%), 9-mesityl-10-methylacridinium perchlorate (6.2 mg, 0.015 mmol, 7.5 mol%), collidine (48.5 mg (~52 µL), 0.4 mmol, 2 equiv.) and 2-(4-methoxyphenyl)undec-10-en-2-ol (55.3 mg, 0.2 mmol, 1 equiv.). The reaction mixture was stirred in DCE (2 mL) and irradiated with a 2 m blue LED strip at room temperature for 72 h. The crude product was purified by flash column chromatography to afford **9** as a colorless oil in 51% yield (12.6 mg).

^{1}H NMR (600 MHz, CDCl$_3$): δ 5.90 – 5.72 (m, 2H), 4.99 (dt, *J* = 17.7, 2.0 Hz, 2H), 4.93 (d, *J* = 10.1 Hz, 2H), 2.08 – 2.01 (m, 4H), 1.42 – 1.36 (m, 4H), 1.35 – 1.29 (m, 2H).

^{13}C NMR (151 MHz, CDCl$_3$): δ 139.3 (d), 114.3 (t), 33.9 (t), 28.9 (t), 28.8 (t).

1-(4-methoxyphenyl)-6-((2,2,6,6-tetramethylpiperidin-1-yl)oxy)hexan-1-one, **10**:[2] the title

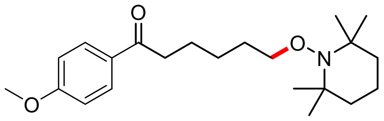

compound was prepared following the general procedure using Co(dmgH)$_2$(py)$_2$PF$_6$ (12 mg, 0.02 mmol, 10 mol%), 9-mesityl-

10-methylacridinium perchlorate (6.2 mg, 0.015 mmol, 7.5 mol%), collidine (48.5 mg (~52 µL), 0.4 mmol, 2 equiv.), 1-(4-methoxyphenyl)cycloheptan-1-ol (44.1 mg, 0.2 mmol, 1 equiv.) and 2,2,6,6-tetramethyl-1-piperidinyloxy (TEMPO, 62.5 mg, 0.4 mmol, 2 equiv.).. The reaction mixture was stirred in DCE (2 mL) and irradiated with a 2 m blue LED strip at room temperature for 48 h. The crude product was purified by flash column chromatography to afford **10** as a colorless oi in 21% yield (15.1 mg).

^{1}H NMR (400 MHz, $CDCl_3$): δ 7.92 (d, *J* = 8.9 Hz, 2H), 6.91 (d, *J* = 8.8 Hz, 2H), 3.84 (s, 3H), 3.71 (t, *J* = 6.5 Hz, 2H), 2.90 (t, *J* = 7.5 Hz, 2H), 1.77 – 1.68 (m, 2H), 1.61 – 1.36 (m, 10H), 1.34 – 1.21 (m, 2H), 1.12 (s, 6H), 1.06 (s, 6H);

^{13}C NMR (101 MHz, $CDCl_3$): δ 199.2 (s), 163.4 (s), 130.4 (d), 130.3 (s), 113.8 (d), 76.7 (t), 59.8 (s), 55.6 (q), 39.7 (t), 38.4 (t), 33.2 (q), 28.8 (t), 26.5 (t), 24.9 (t), 20.3 (q), 17.3 (t);

1-(4-methoxyphenyl)-8-phenyloct-7-en-1-one, **11**: the title compounds were prepared following the general procedure using $Co(dmgH)_2(py)_2PF_6$ (6.0 mg, 0.01 mmol, 5 mol%), $[Ir(dF(CF_3)ppy)_2(5,5'd(CF_3)bpy)](PF_6)$ (4.6 mg, 0.004 mmol, 2 mol%), collidine (24.2 mg (~26 µL), 0.2 mmol, 1 equiv.), styrene (62.4 mg (69 µL), 0.6 mmol, 3 equiv.) and 1-(4-methoxyphenyl)cyclohexan-1-ol (41.2 mg, 0.2 mmol, 1 equiv.). The reaction mixture was stirred in DCE (1 mL) and irradiated with a 2 m blue LED strip at room temperature for 72 h. The crude product was purified by flash column chromatography to afford **11** as a white solid in 25% yield (*Z*:*E* ratio = 2.7:1, 15.1 mg).

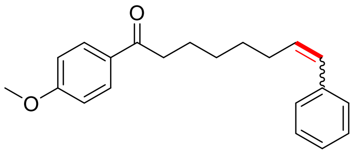

^{1}H NMR (600 MHz, $CDCl_3$): δ 7.97 – 7.92 (m, 2.74H_{E+Z}), 7.36 – 7.31 (m, 2.74H_{E+Z}), 7.30 – 7.26 (m, 2.74H_{E+Z}), 7.23 – 7.17 (m, 1.37H_{E+Z}), 6.92 (d, *J* = 8.3 Hz, 2.74H_{E+Z}), 6.41 (d, *J* = 11.6 Hz, 1H_Z), 6.38 (d, *J* = 15.7 Hz, 0.37H_E), 6.22 (dt, *J* = 15.7, 7.3 Hz, 0.37H_E), 5.66 (dt, J = 11.1, 7.3 Hz, 1H_Z), 3.87 (s, 4.2H_{E+Z}), 2.97 – 2.88 (m, 2.74H_{E+Z}), 2.39 – 2.33 (m, 2H_Z), 2.26 – 2.20 (m, 0.74H_E), 1.79 – 1.70 (m, 2.74H_{E+Z}), 1.55 – 1.48 (m, 2.74H_{E+Z}), 1.47 – 1.39 (m, 2.74H_{E+Z}).

^{13}C NMR (151 MHz, $CDCl_3$): δ 199.2 (s, 2C), 163.4 (s), 138.0 (s), 137.8 (s), 133.1 (d), 131.0 (d), 130.4 (d), 130.3 (s), 130.0 (d), 129.0 (d), 128.9 (d), 128.6 (d), 128.2 (d), 126.9 (d), 126.6 (d), 126.0 (d), 113.8 (d), 55.6 (q), 38.4 (t), 38.3 (t), 33.0 (t), 30.0 (t), 29.3 (t, 2C), 29.1 (t), 28.6 (t), 24.6 (t, 2C).

IR (ATR) ν 3343, 3012, 2927, 2856, 2334, 2051, 1991, 1910, 1744, 1673, 1594, 1510, 1464, 1407, 1336, 1248, 1128, 1021, 969, 827, 749 cm^{-1};

HRMS (ESI) for $C_{21}H_{24}O_2Na^+$ $(M+Na)^+$: 331.1669; Found : 331.1669.

7. NMR Spectra

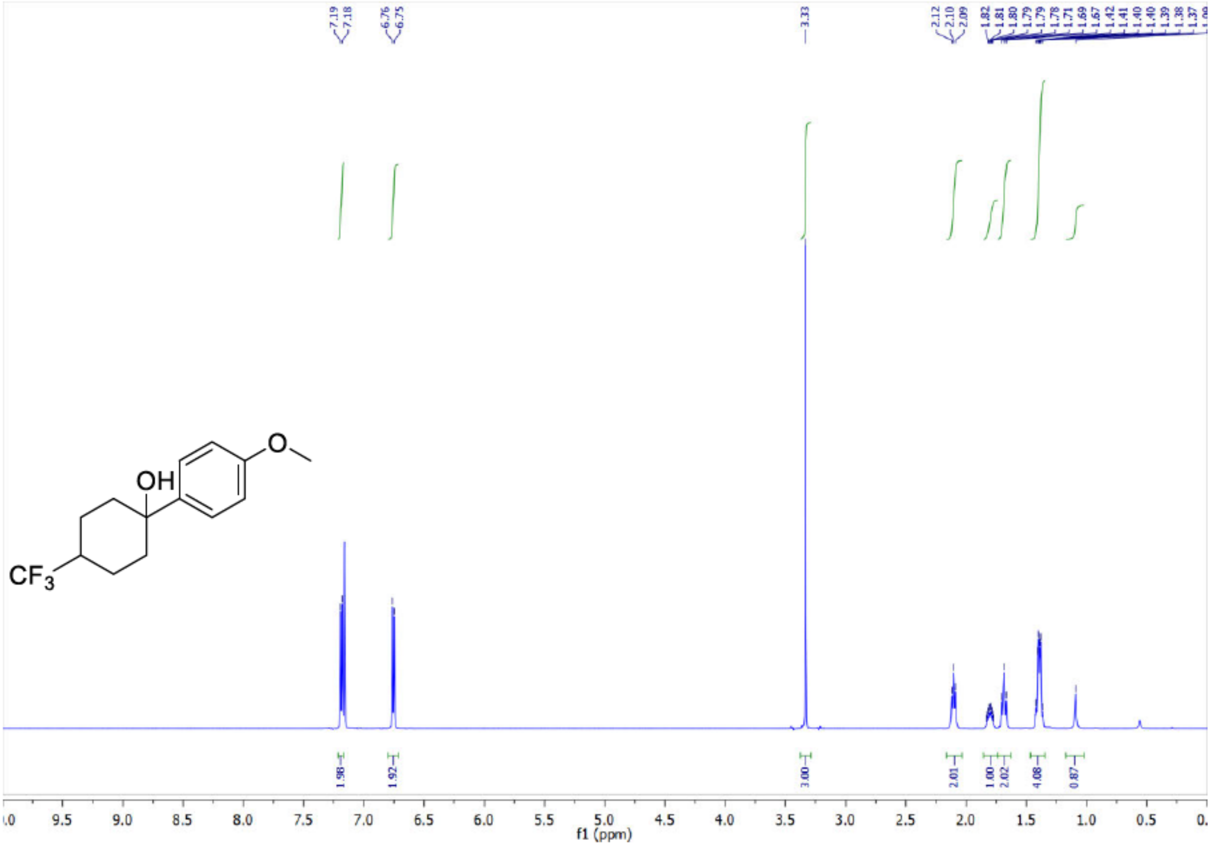

Supplementary Figure 6. ^{1}H NMR spectra of compound of **1j**

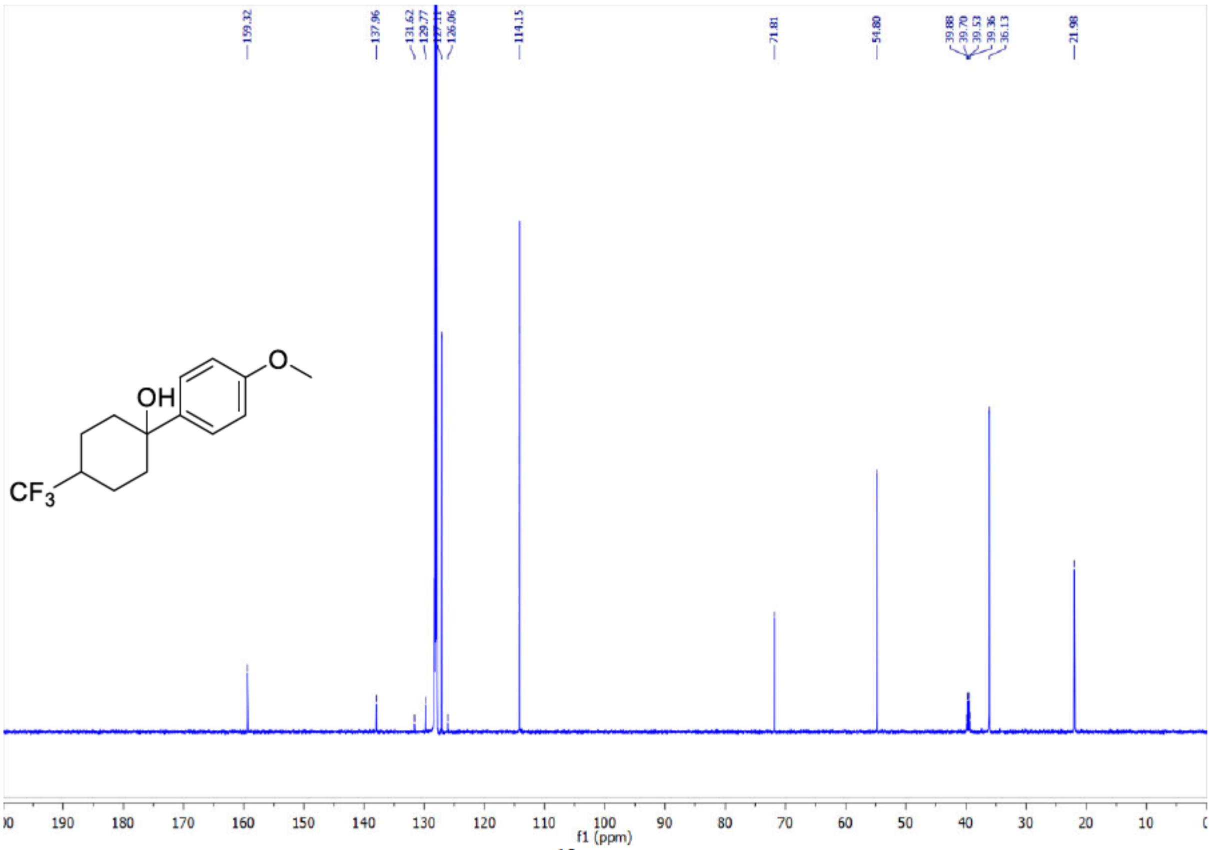

Supplementary Figure 7. ^{13}C NMR spectra of compound of **1j**

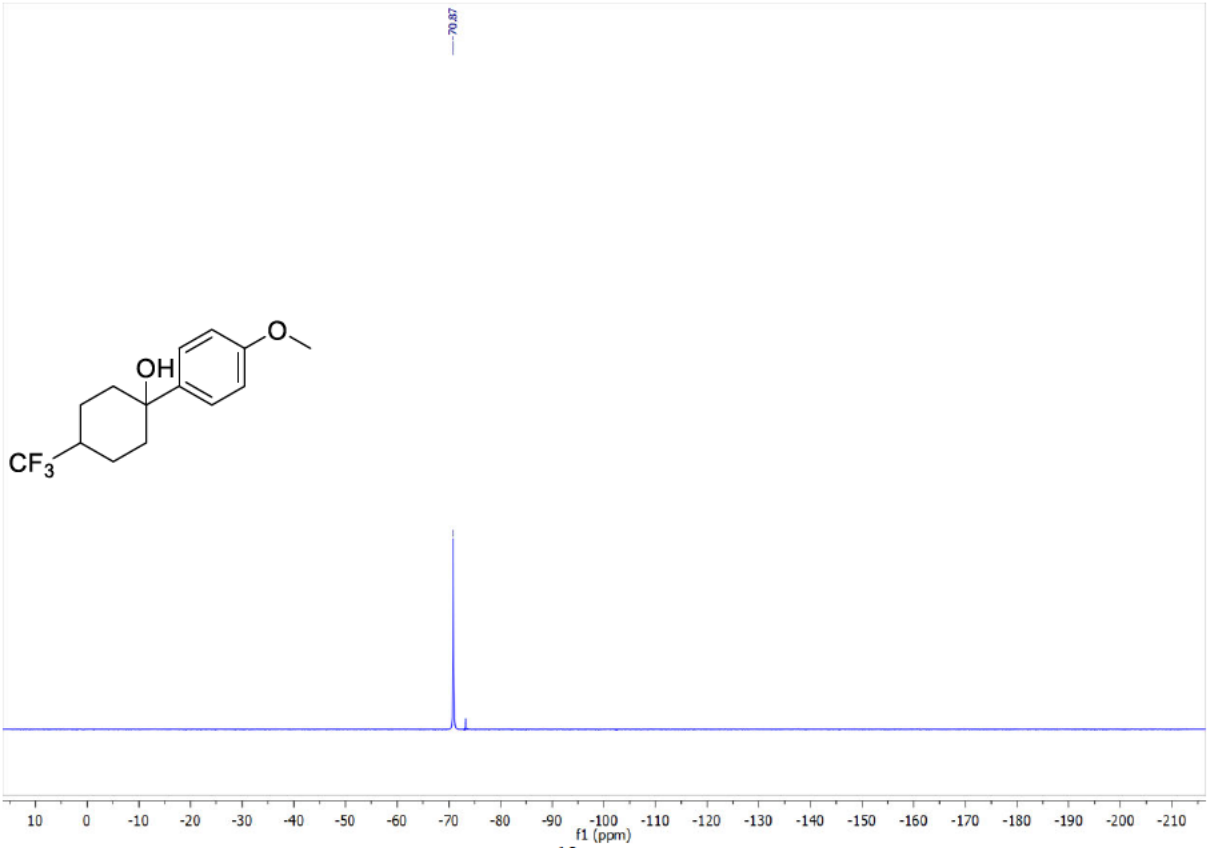

Supplementary Figure 8. ^{19}F NMR spectra of compound of **1j**

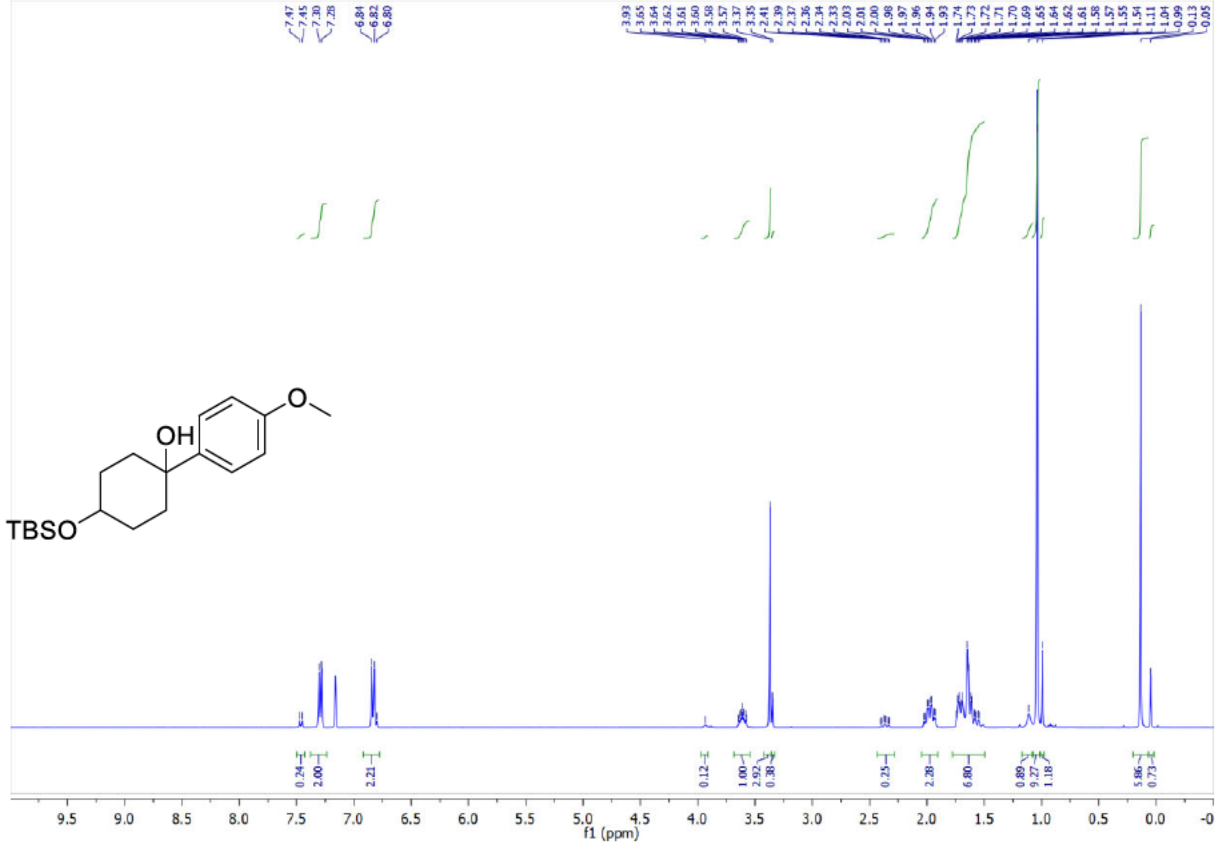

Supplementary Figure 9. ^{1}H NMR spectra of compound of **1k**

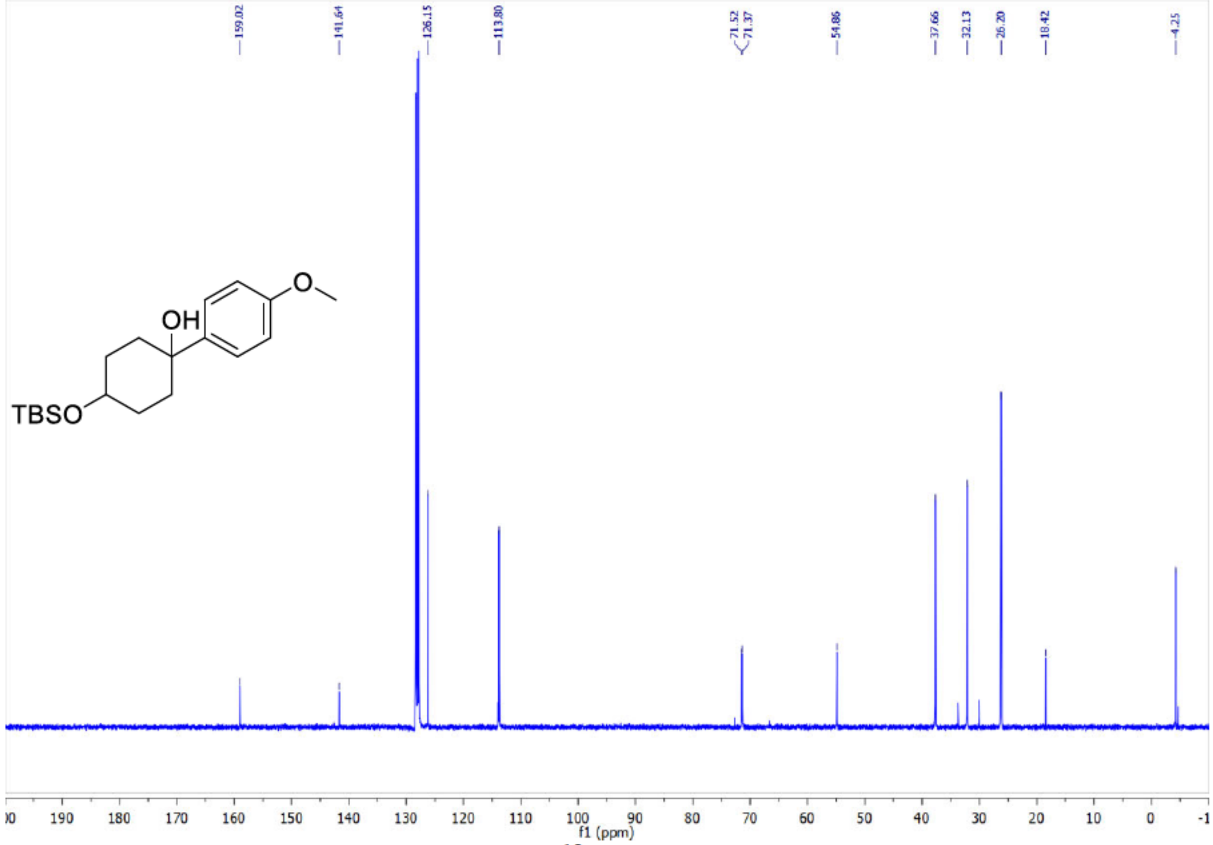

Supplementary Figure 10. ^{13}C NMR spectra of compound of **1k**

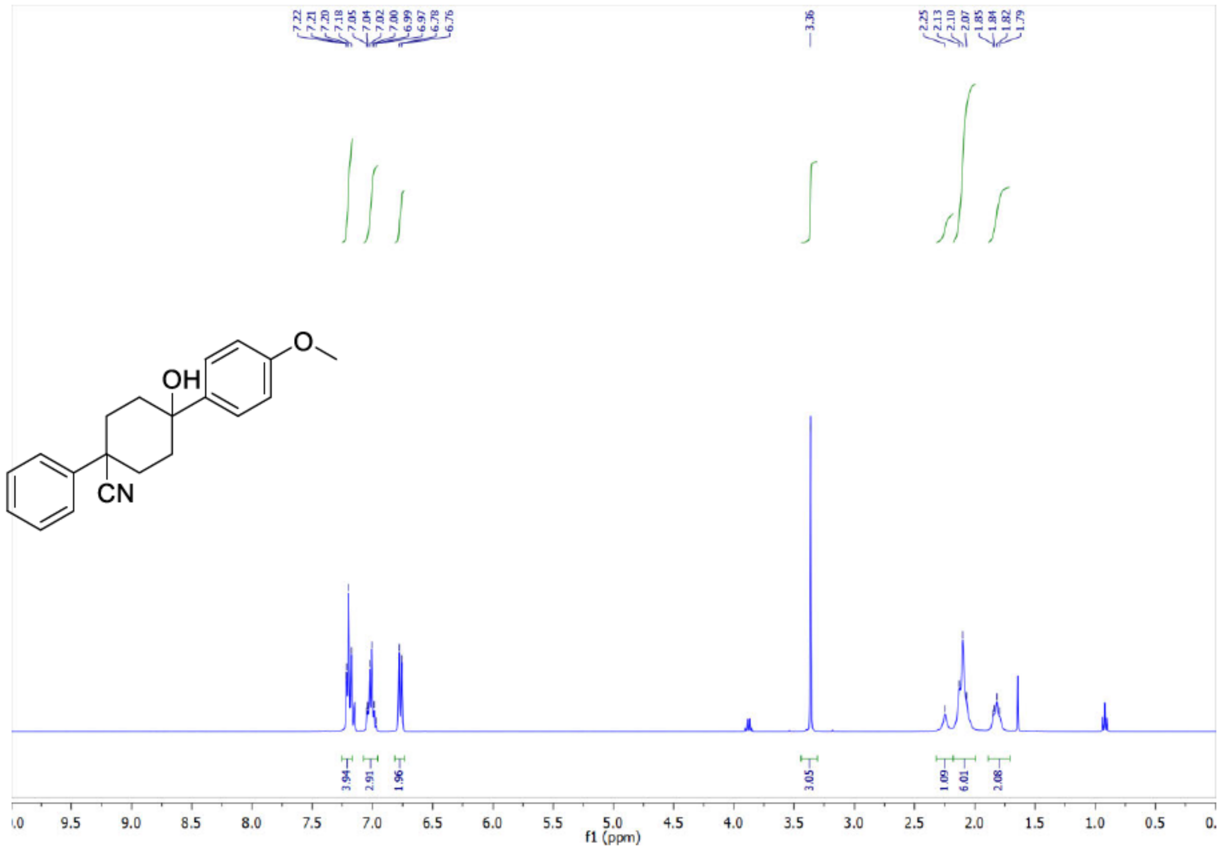

Supplementary Figure 11. ^{1}H NMR spectra of compound of **1o**

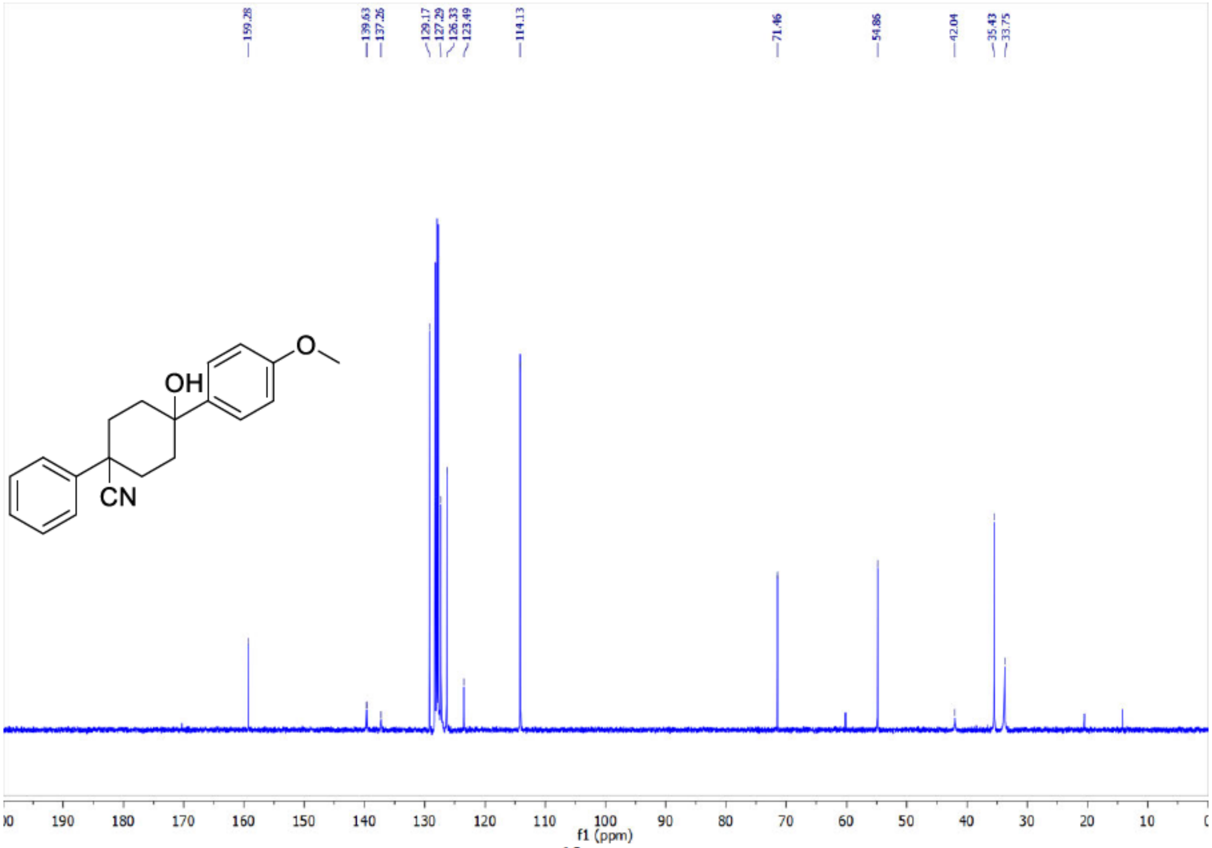

Supplementary Figure 12. ^{13}C NMR spectra of compound of **1o**

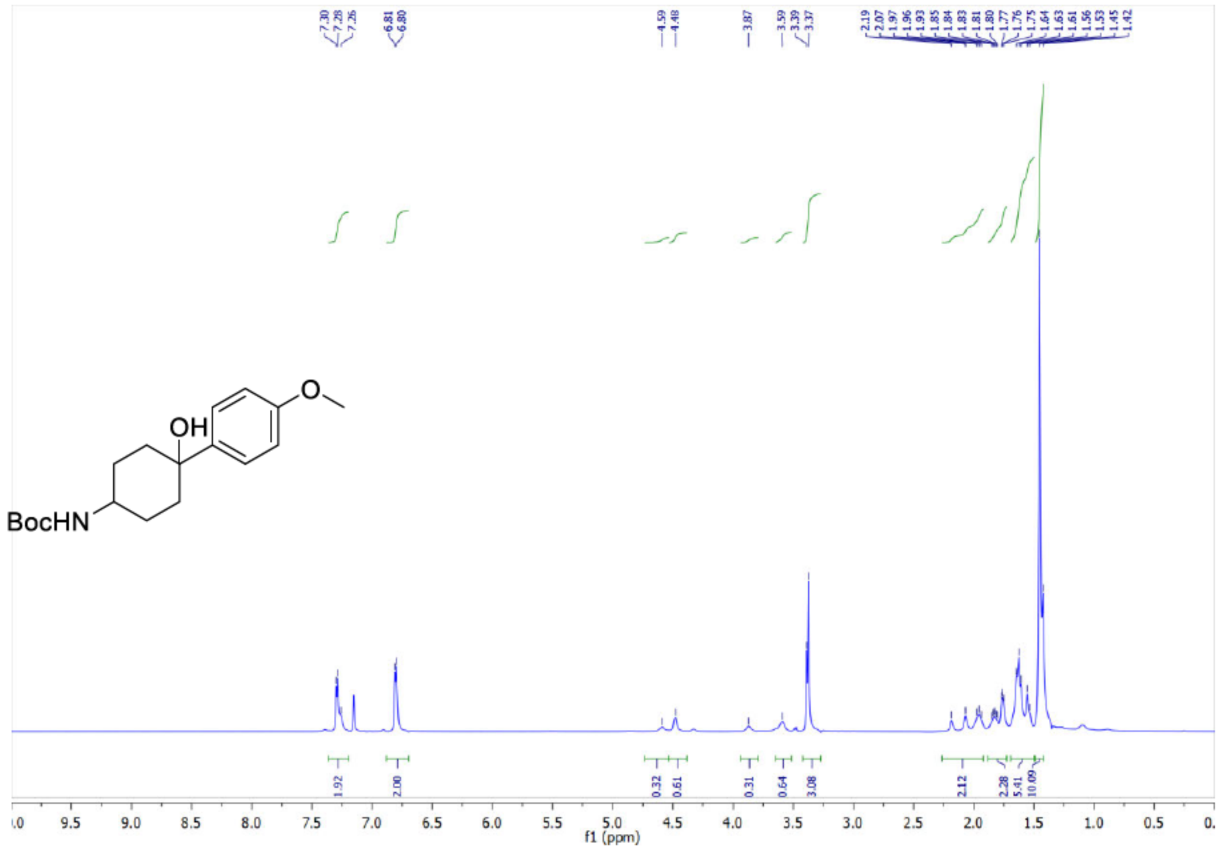

Supplementary Figure 13. ^{1}H NMR spectra of compound of **1p**

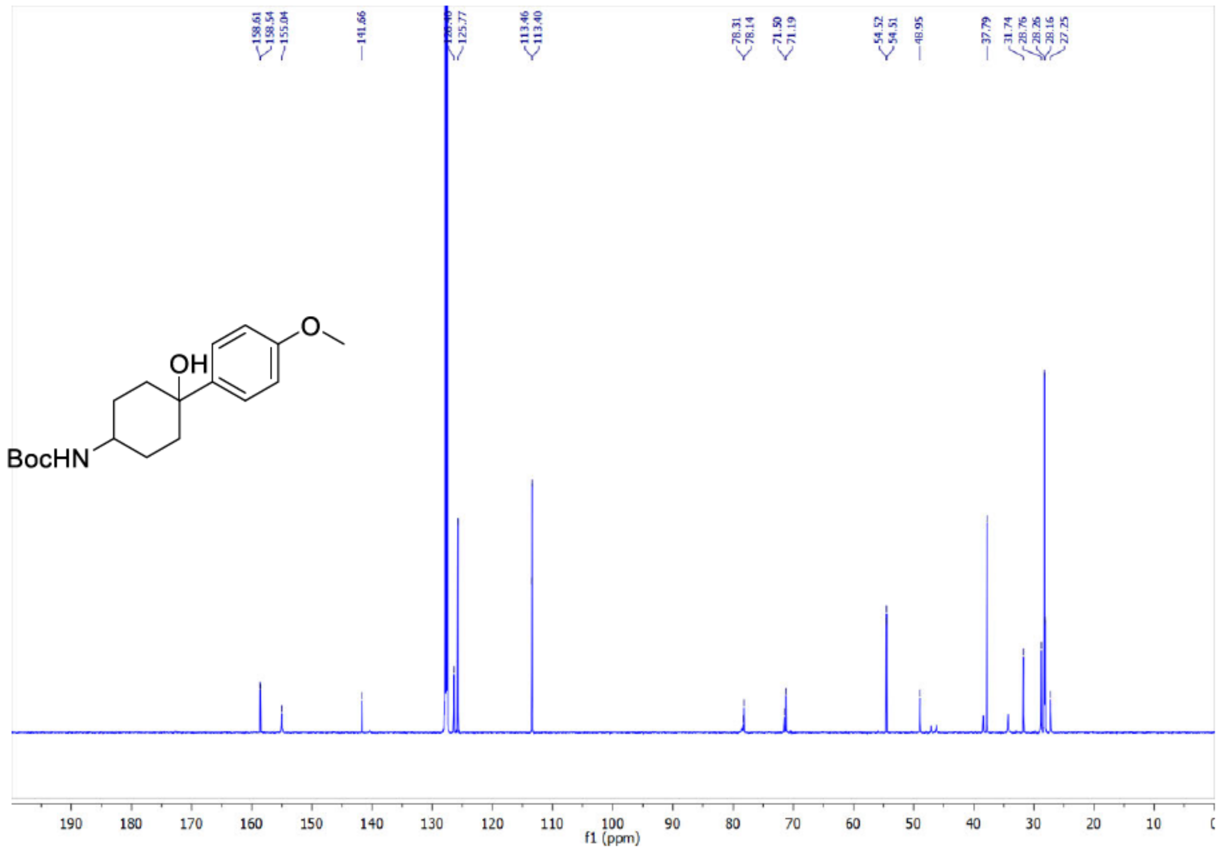

Supplementary Figure 14. ^{13}C NMR spectra of compound of **1p**

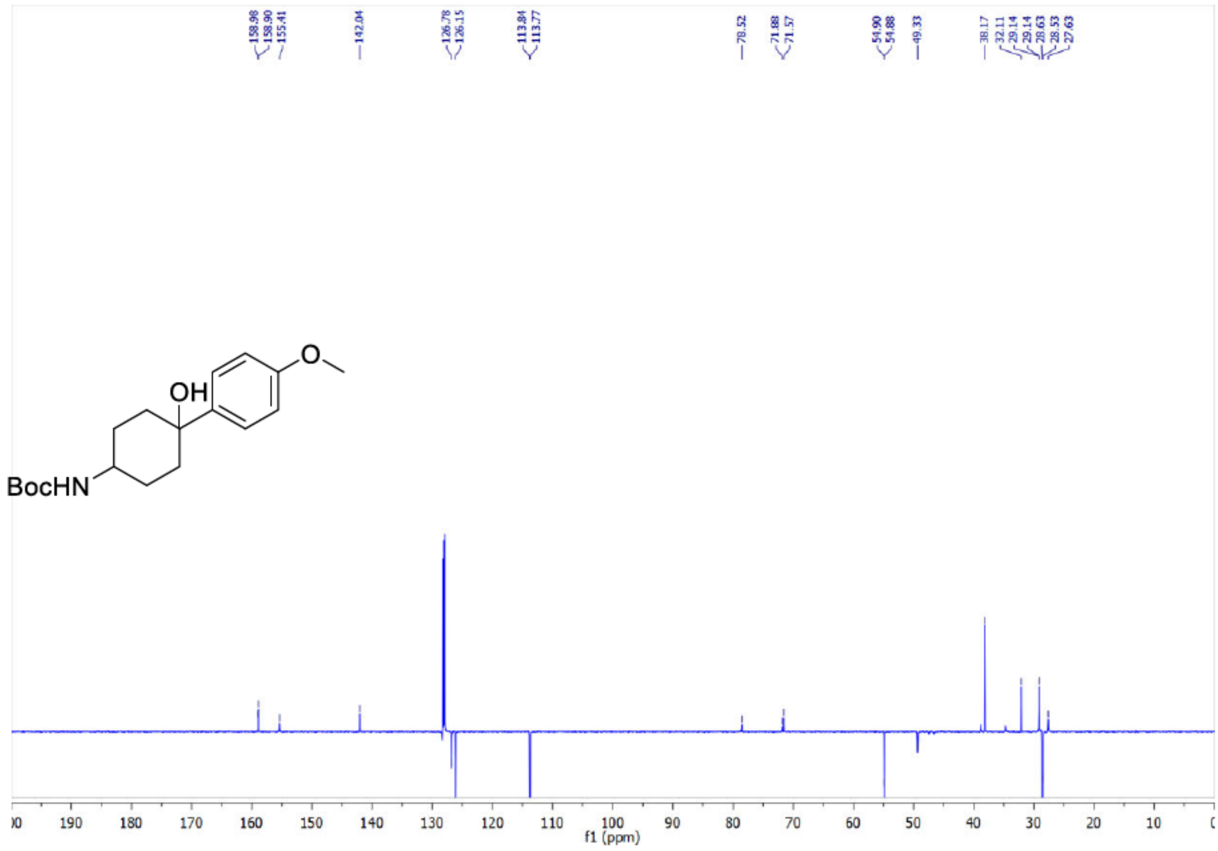

Supplementary Figure 15. ^{13}C APT spectra of compound of **1p**

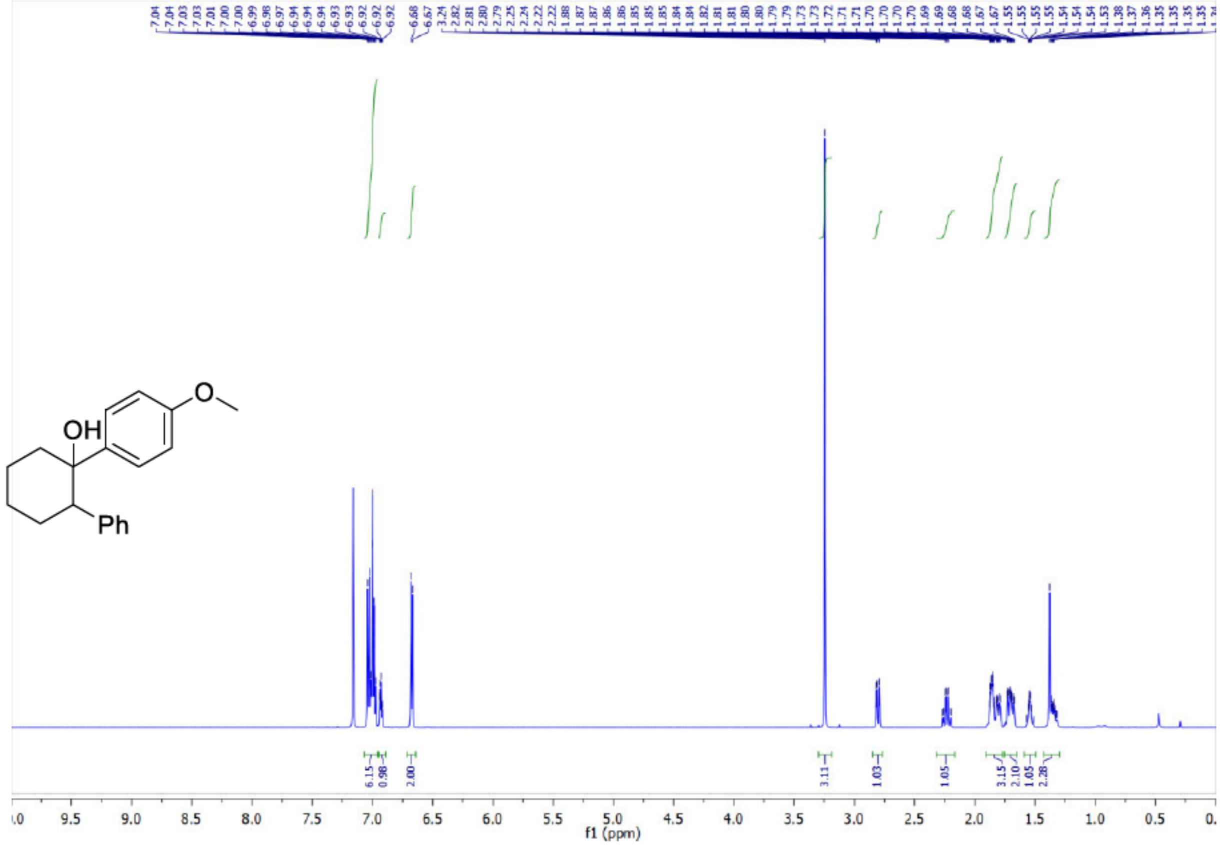

Supplementary Figure 16. ^{1}H NMR spectra of compound of **1r**

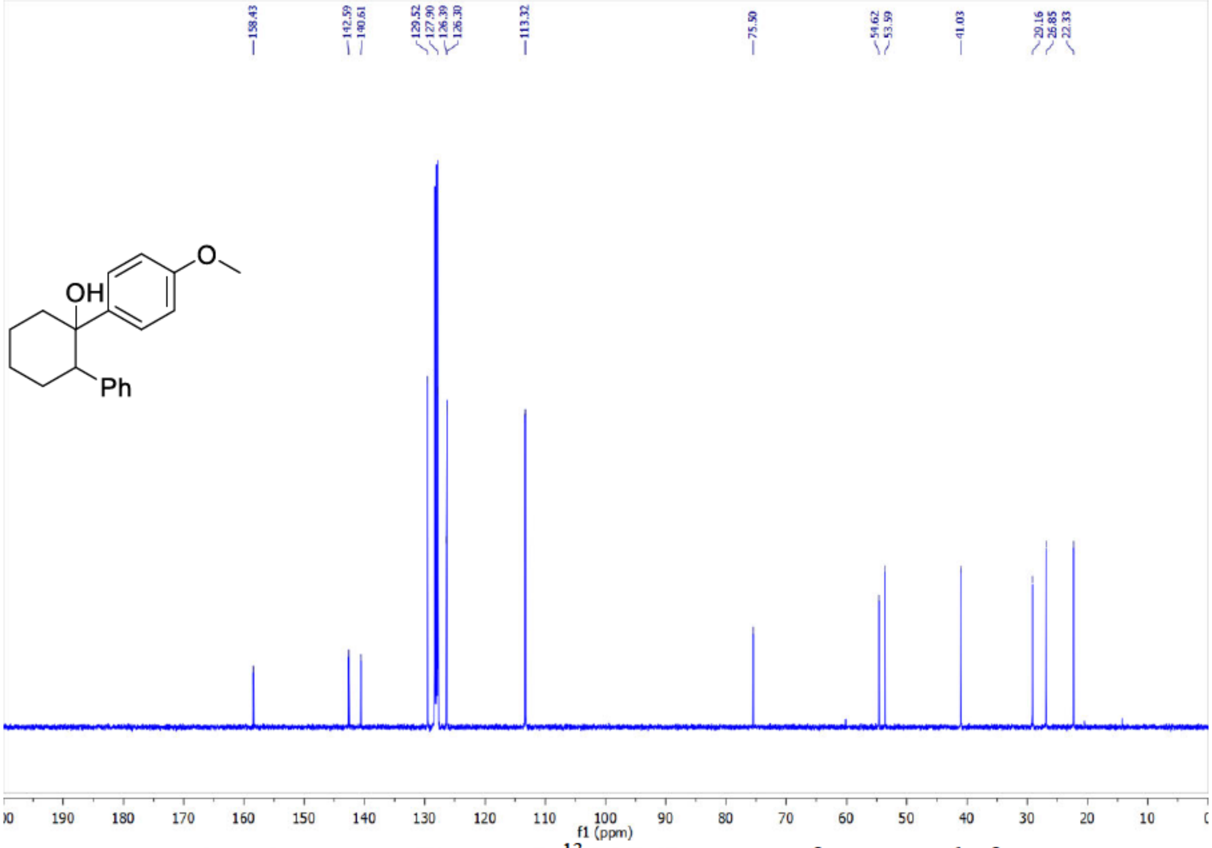

Supplementary Figure 17. ^{13}C NMR spectra of compound of **1r**

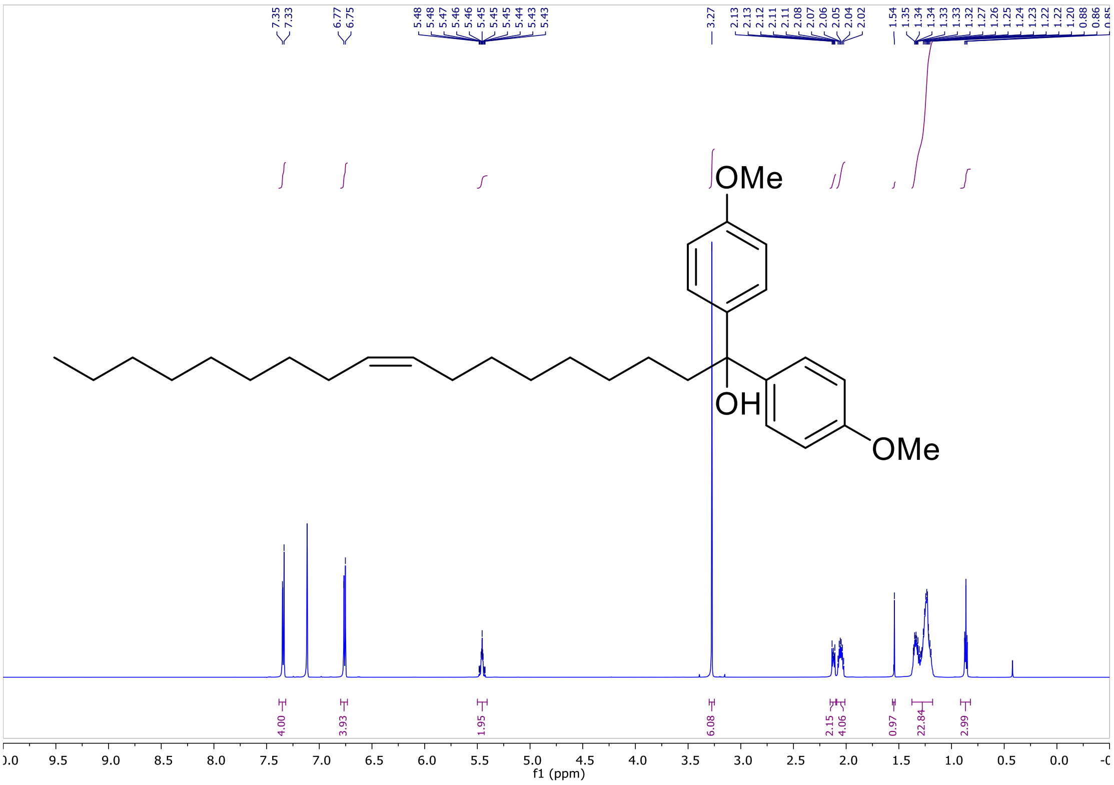

Supplementary Figure 18. ^{1}H NMR spectra of compound of **1w**

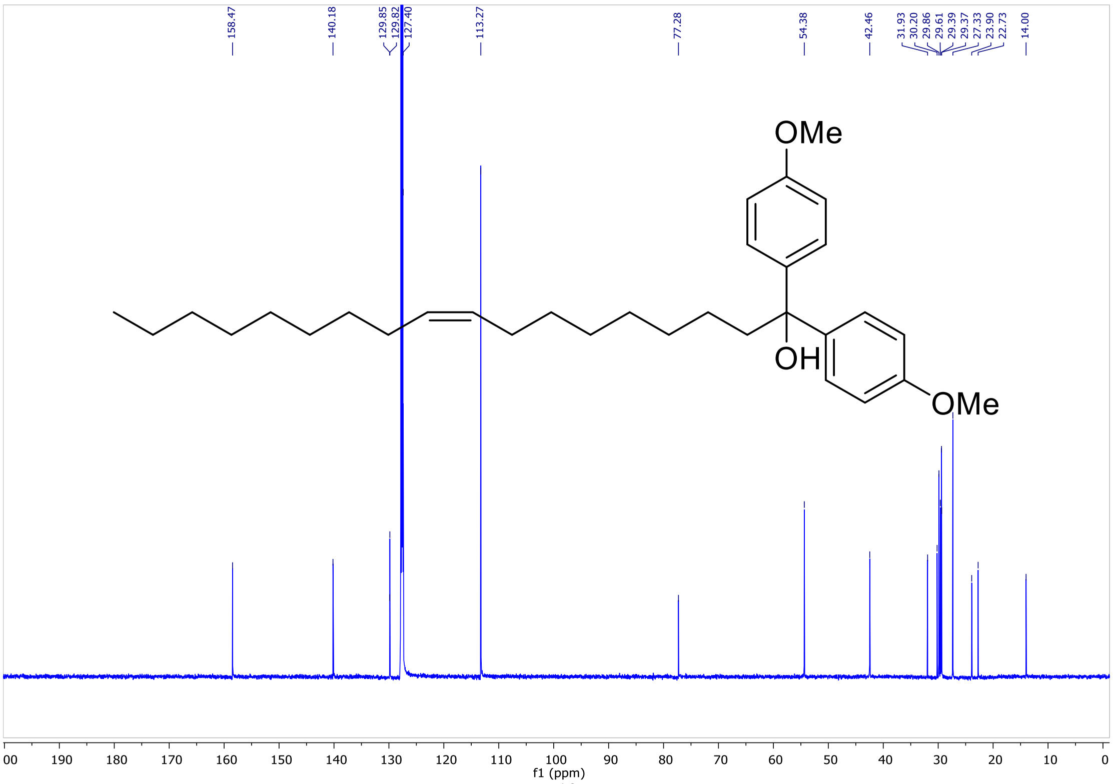

Supplementary Figure 19. ^{13}C NMR spectra of compound of **1w**

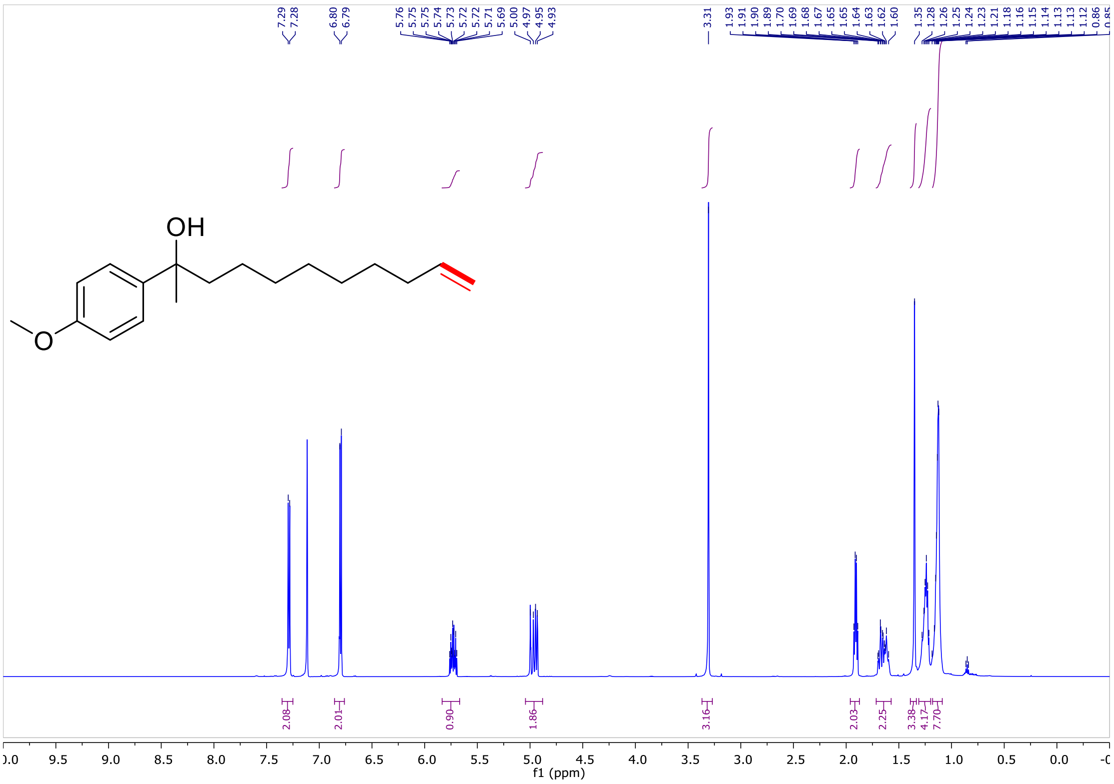

Supplementary Figure 20. ^{1}H NMR spectra of compound of **8**

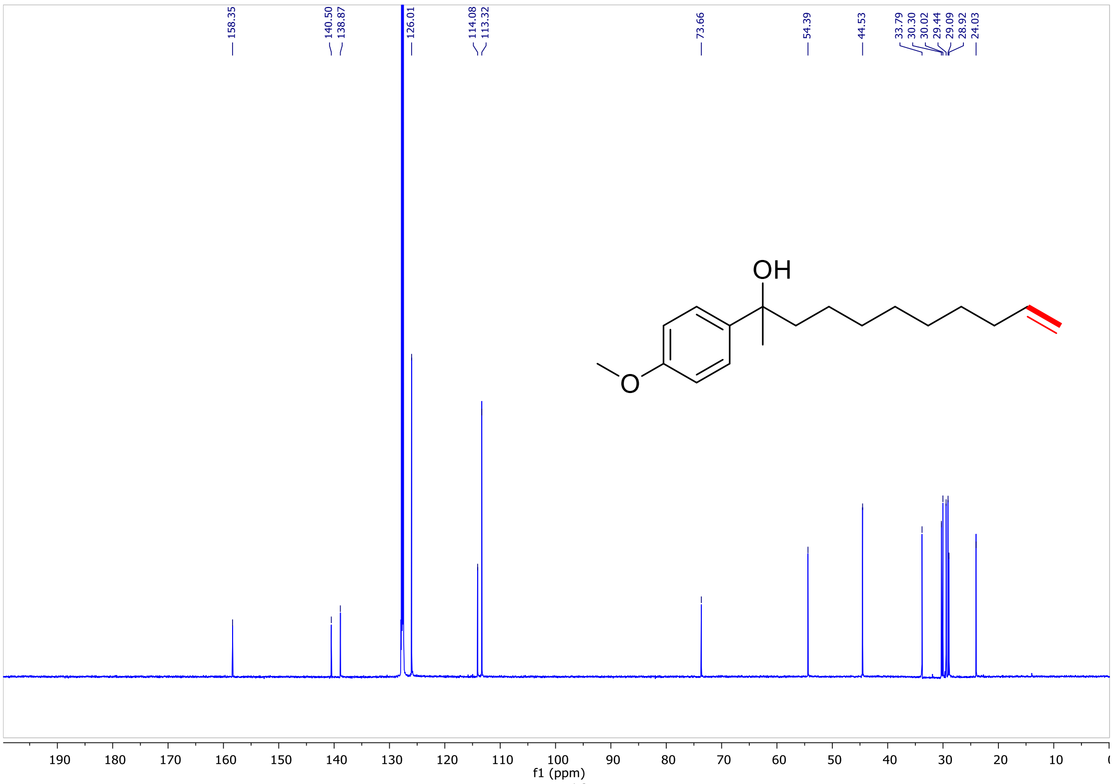

Supplementary Figure 21. ^{13}C NMR spectra of compound of **8**

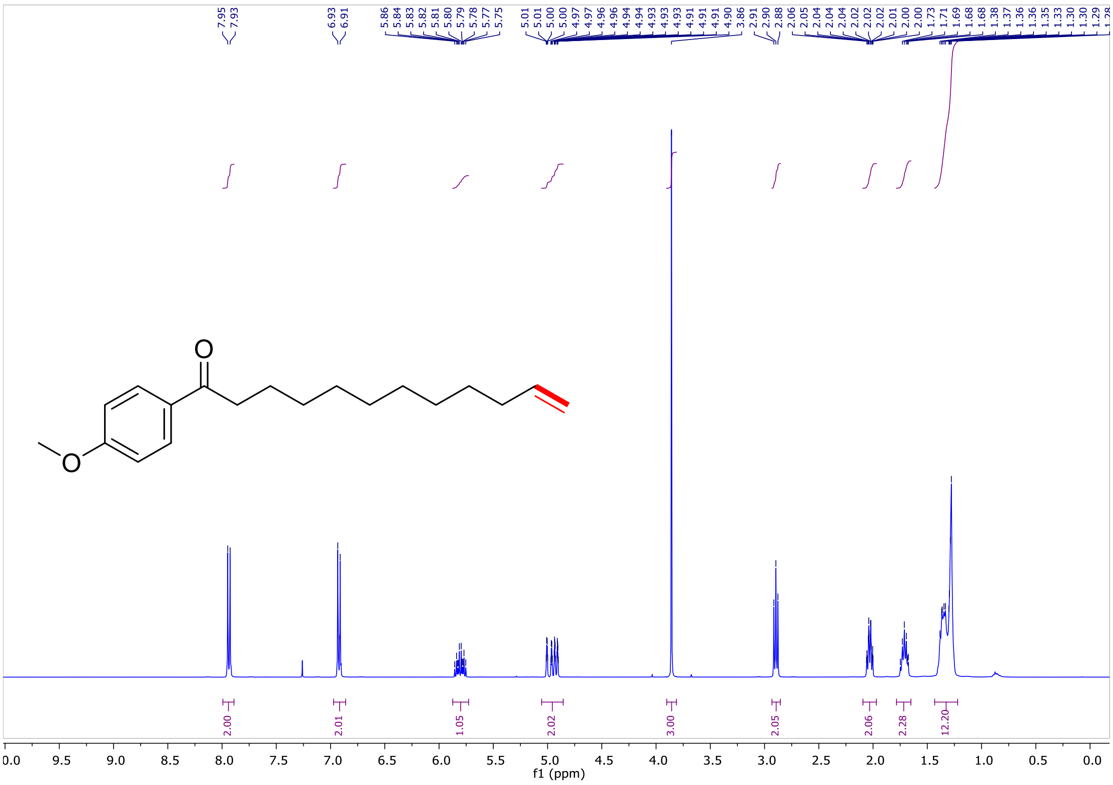

Supplementary Figure 22. ^{1}H NMR spectra of compound of **2a**

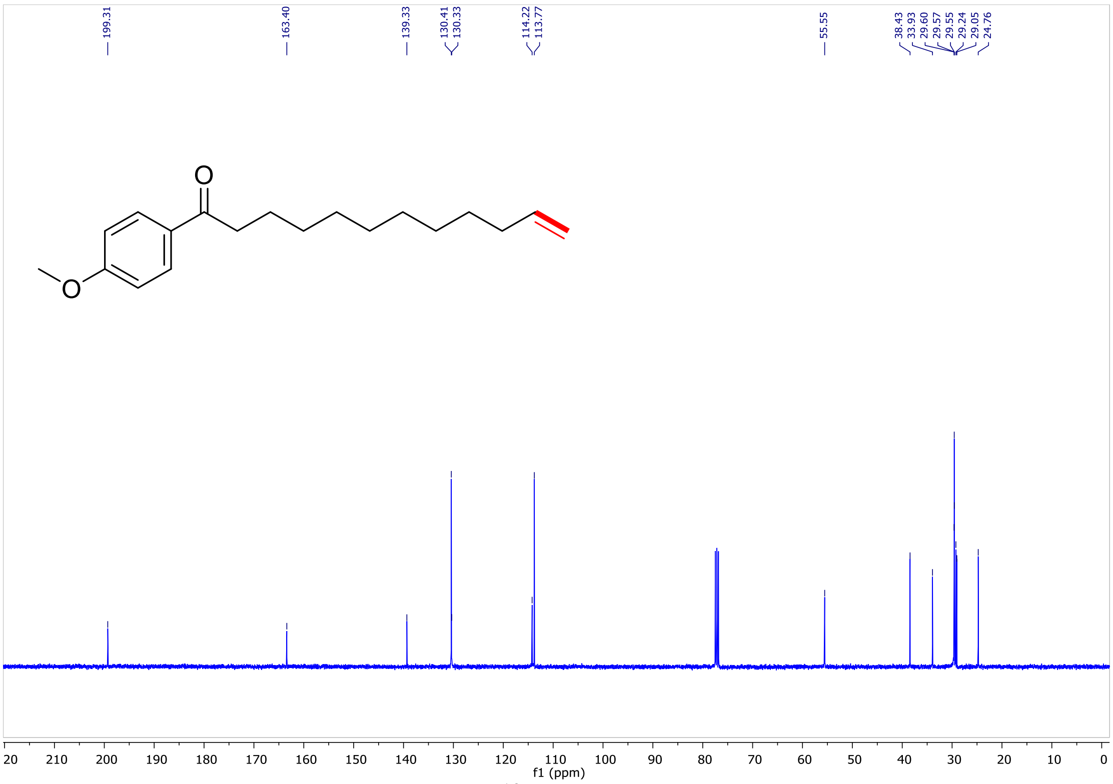

Supplementary Figure 23. ^{13}C NMR spectra of compound of **2a**

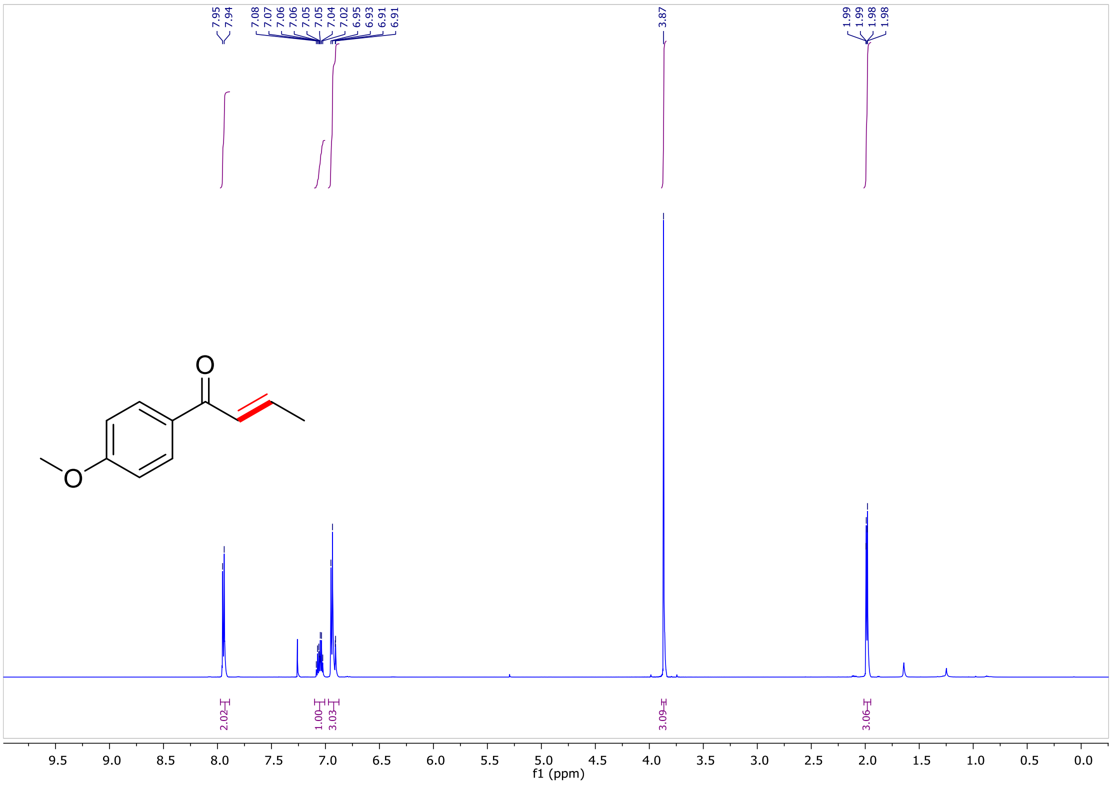

Supplementary Figure 24. ^{1}H NMR spectra of compound of **2b**

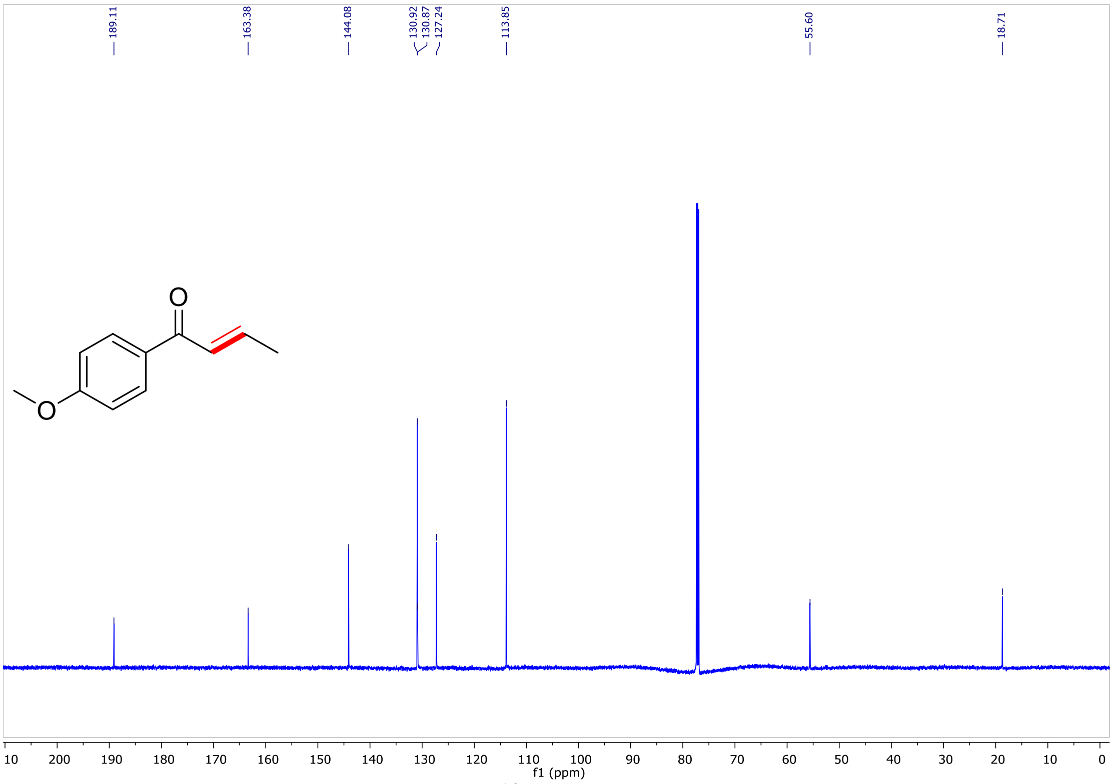

Supplementary Figure 25. ^{13}C NMR spectra of compound of **2b**

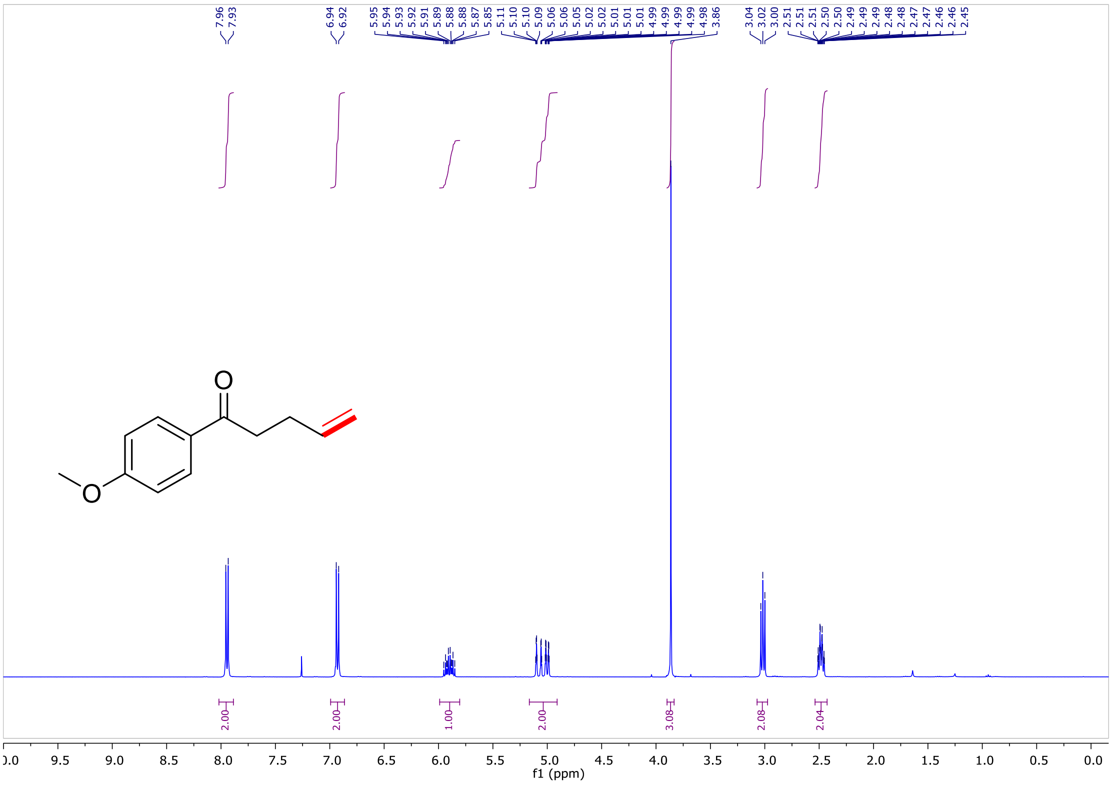

Supplementary Figure 26. ^{1}H NMR spectra of compound of **2c**

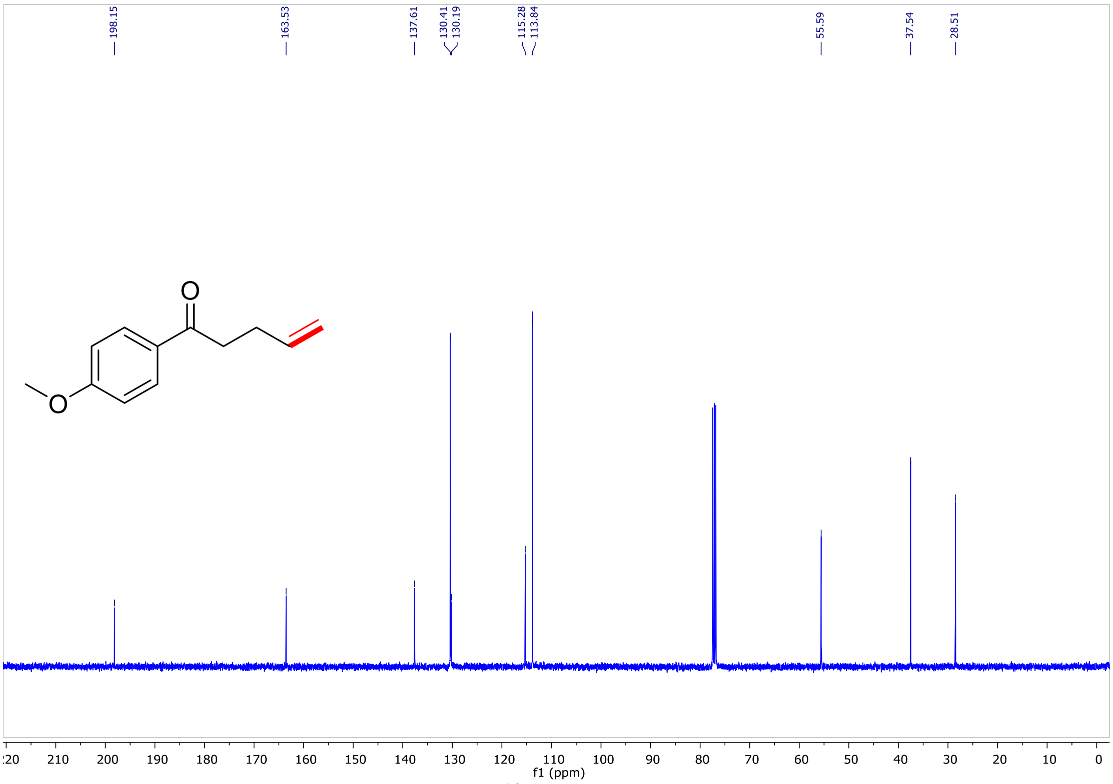

Supplementary Figure 27. ^{13}C NMR spectra of compound of **2c**

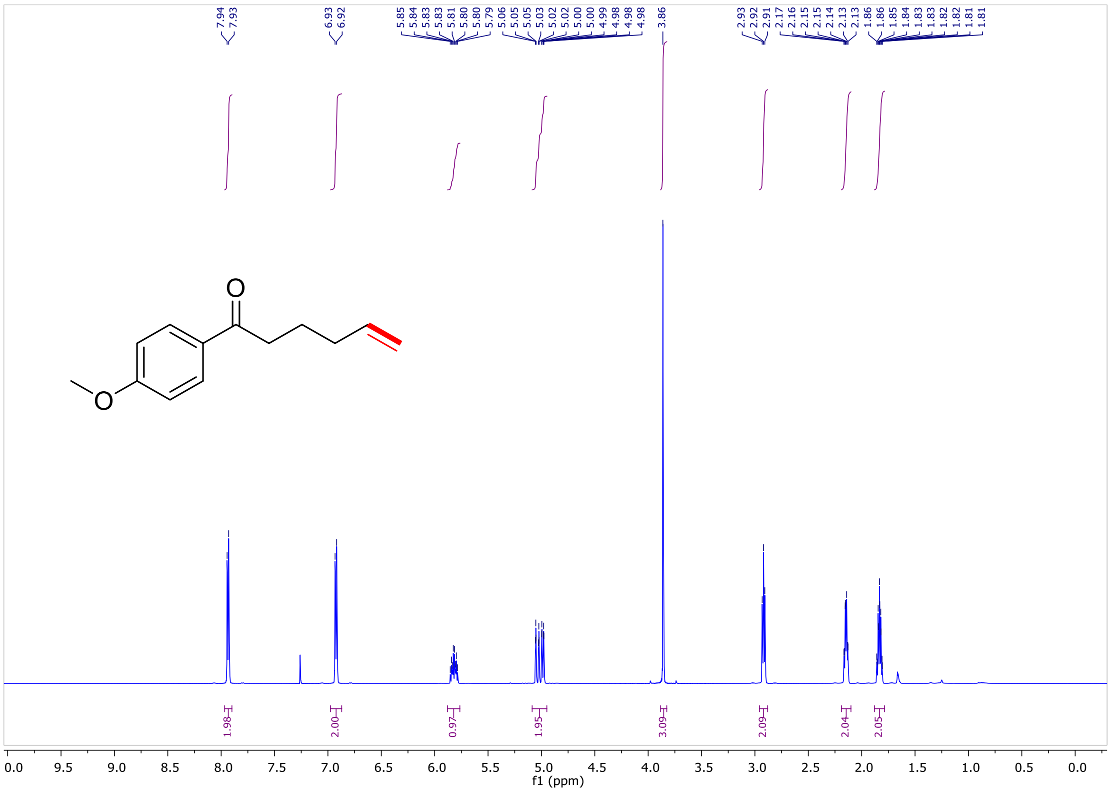

Supplementary Figure 28. ^{1}H NMR spectra of compound of **2d**

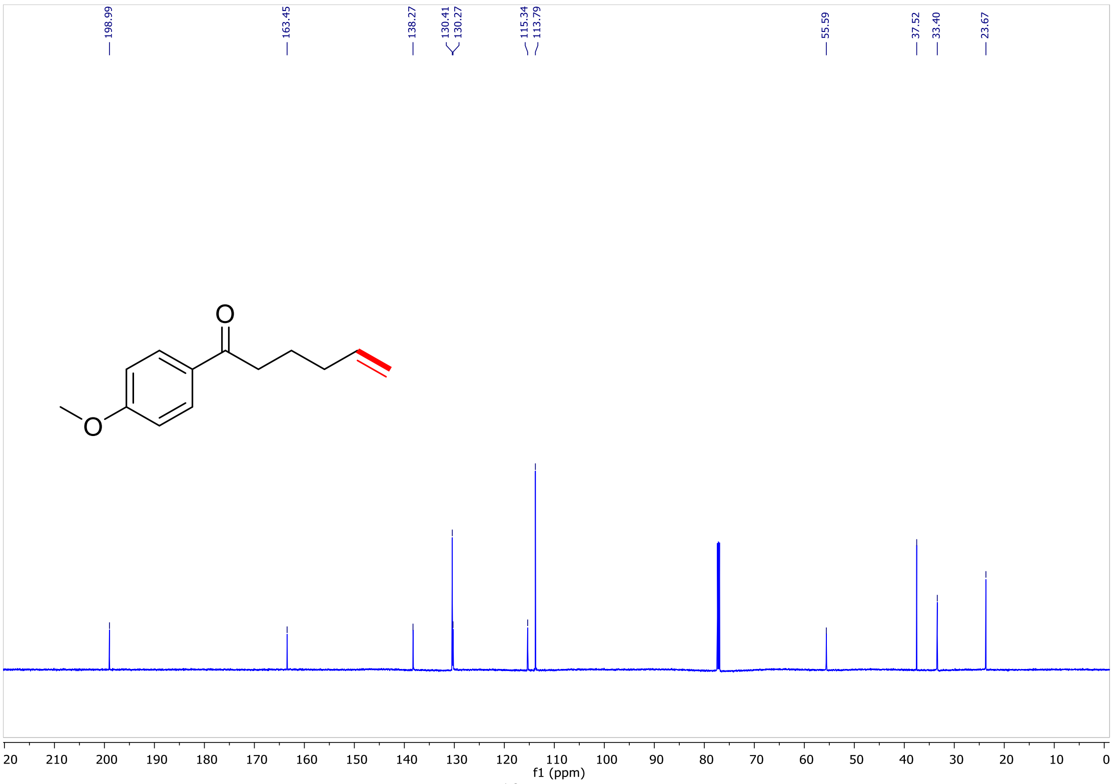

Supplementary Figure 29. ^{13}C NMR spectra of compound of **2d**

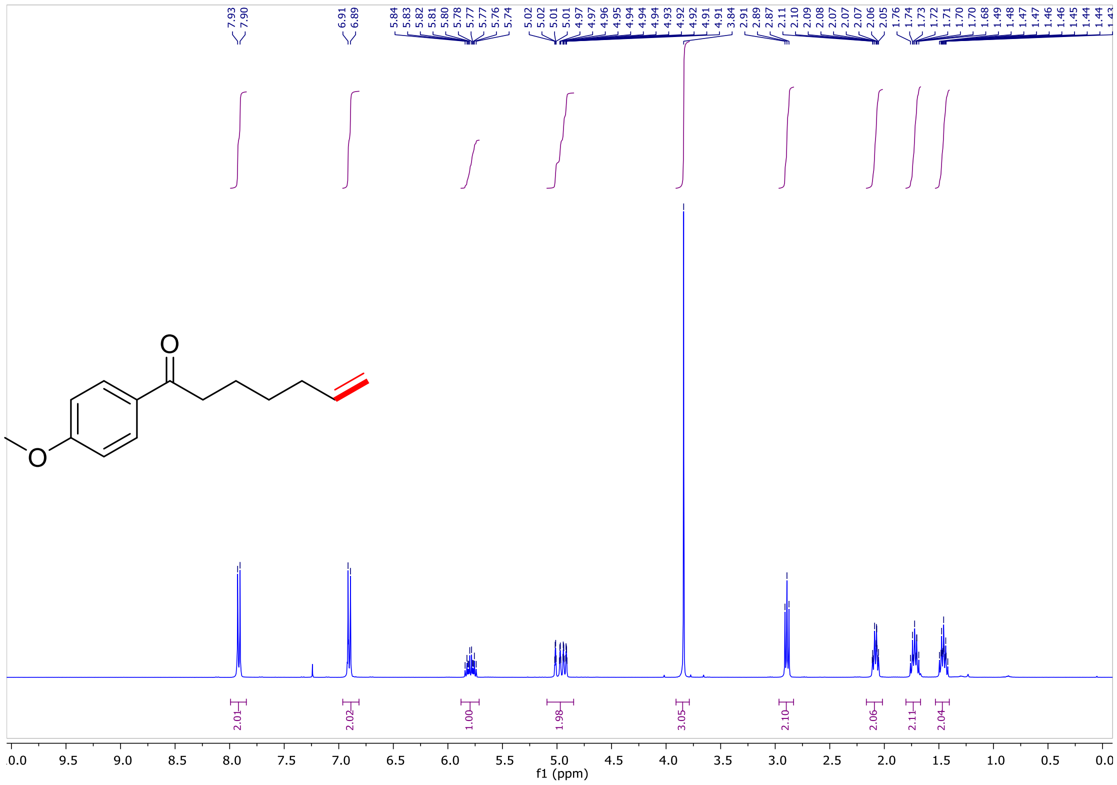

Supplementary Figure 30. ^{1}H NMR spectra of compound of **2e**

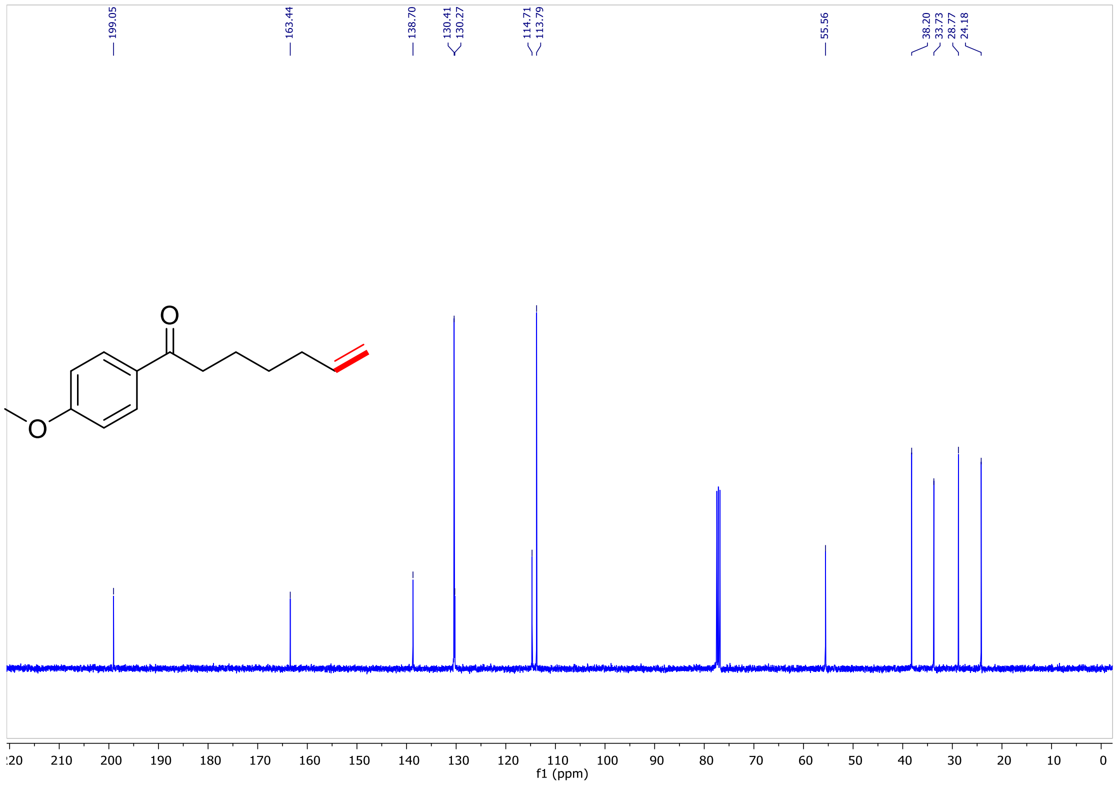

Supplementary Figure 31. ^{13}C NMR spectra of compound of **2e**

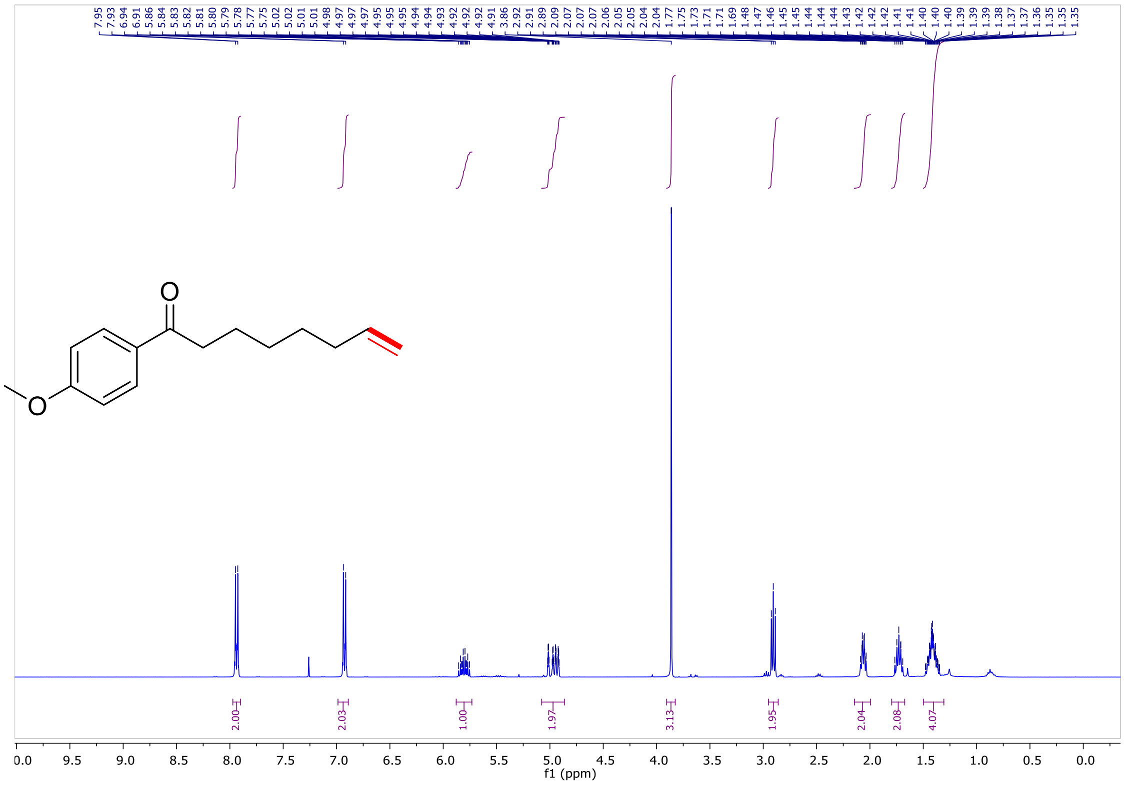

Supplementary Figure 32. ^{1}H NMR spectra of compound of **2f**

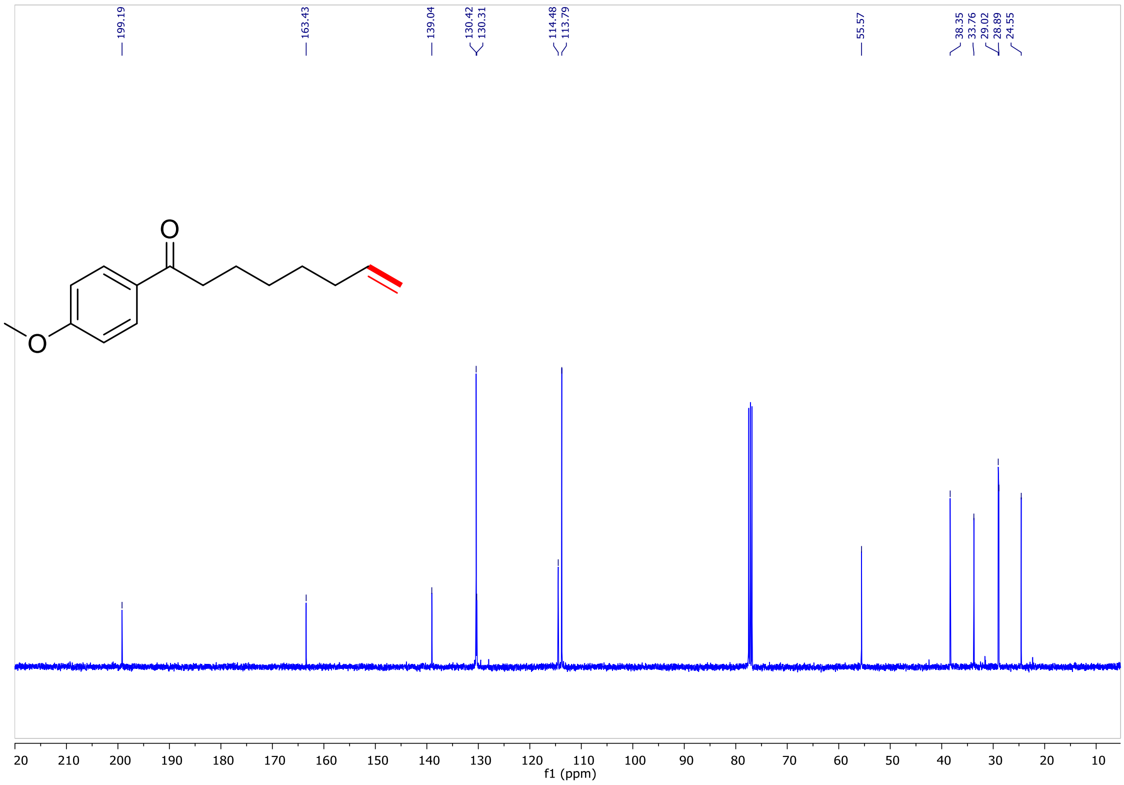

Supplementary Figure 33. ^{13}C NMR spectra of compound of **2f**

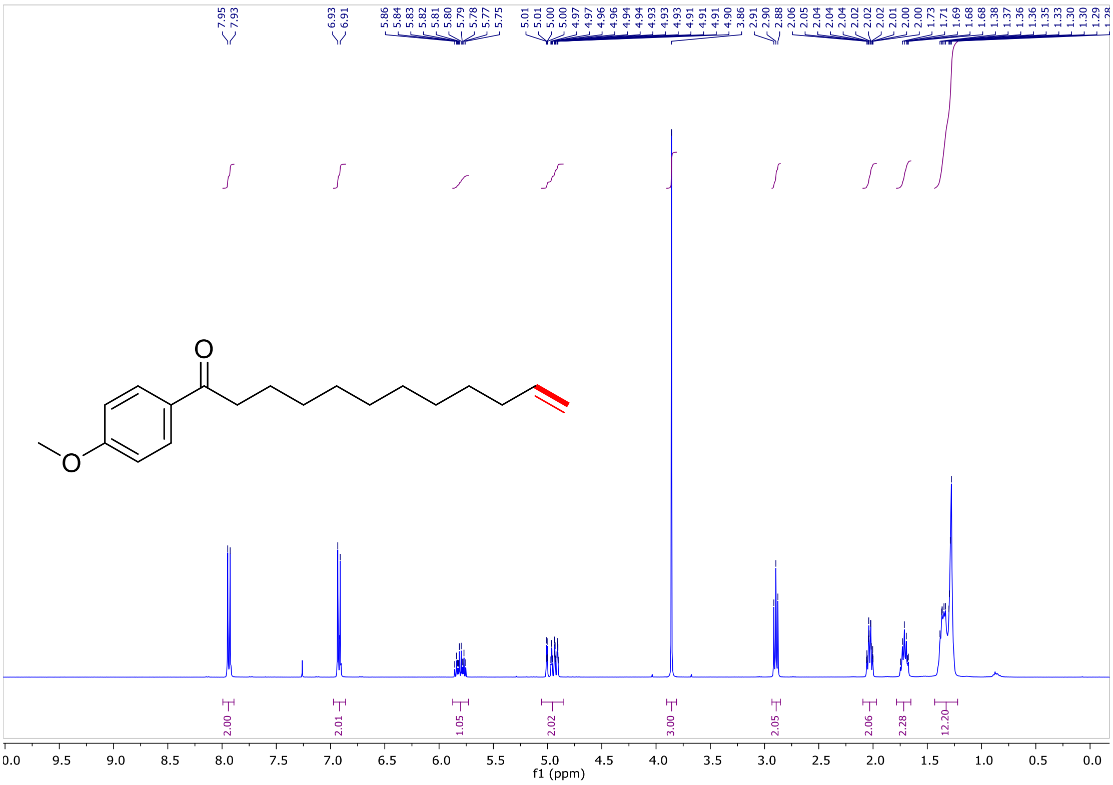

Supplementary Figure 34. ^{1}H NMR spectra of compound of **2g**

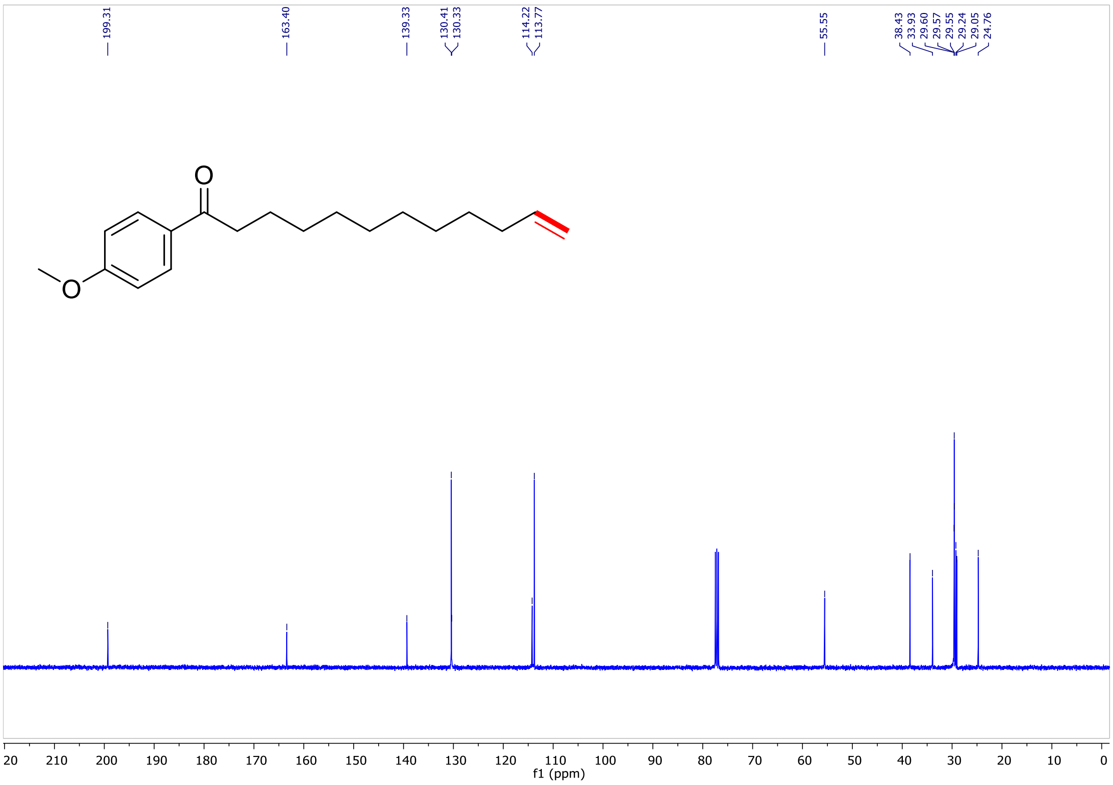

Supplementary Figure 35. ^{13}C NMR spectra of compound of **2g**

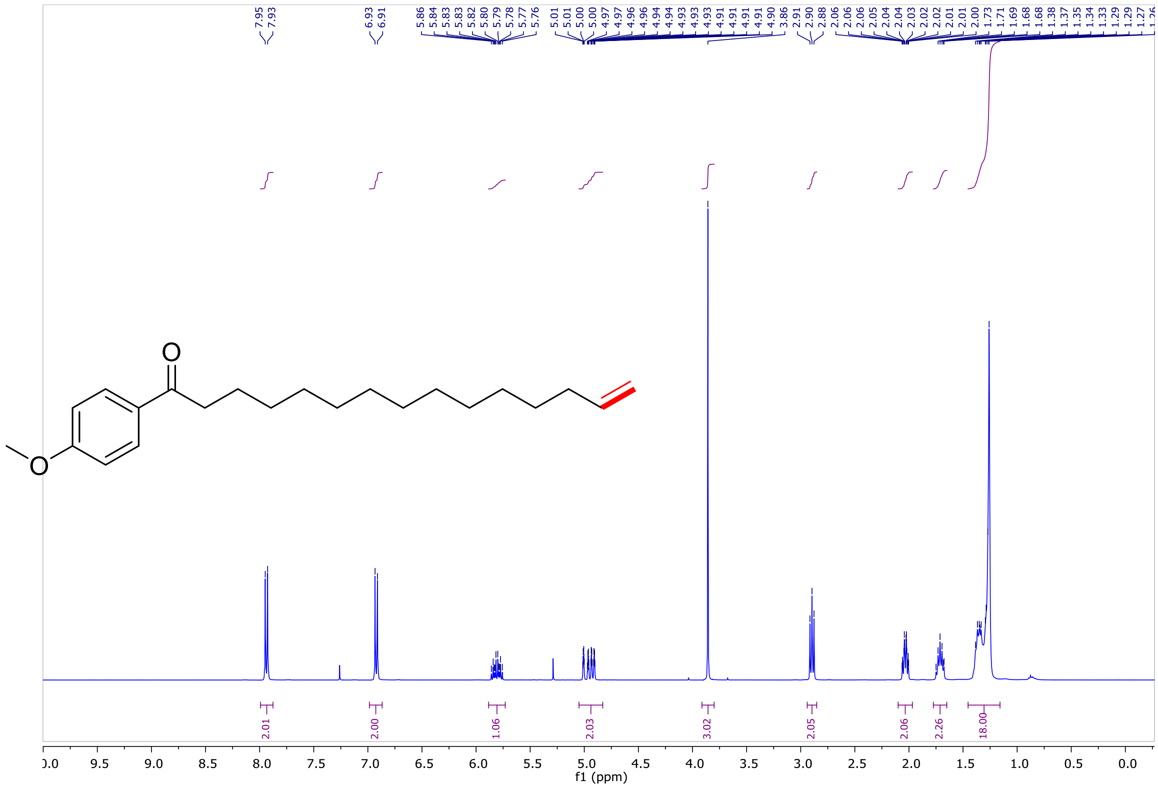

Supplementary Figure 36. ^{1}H NMR spectra of compound of **2h**

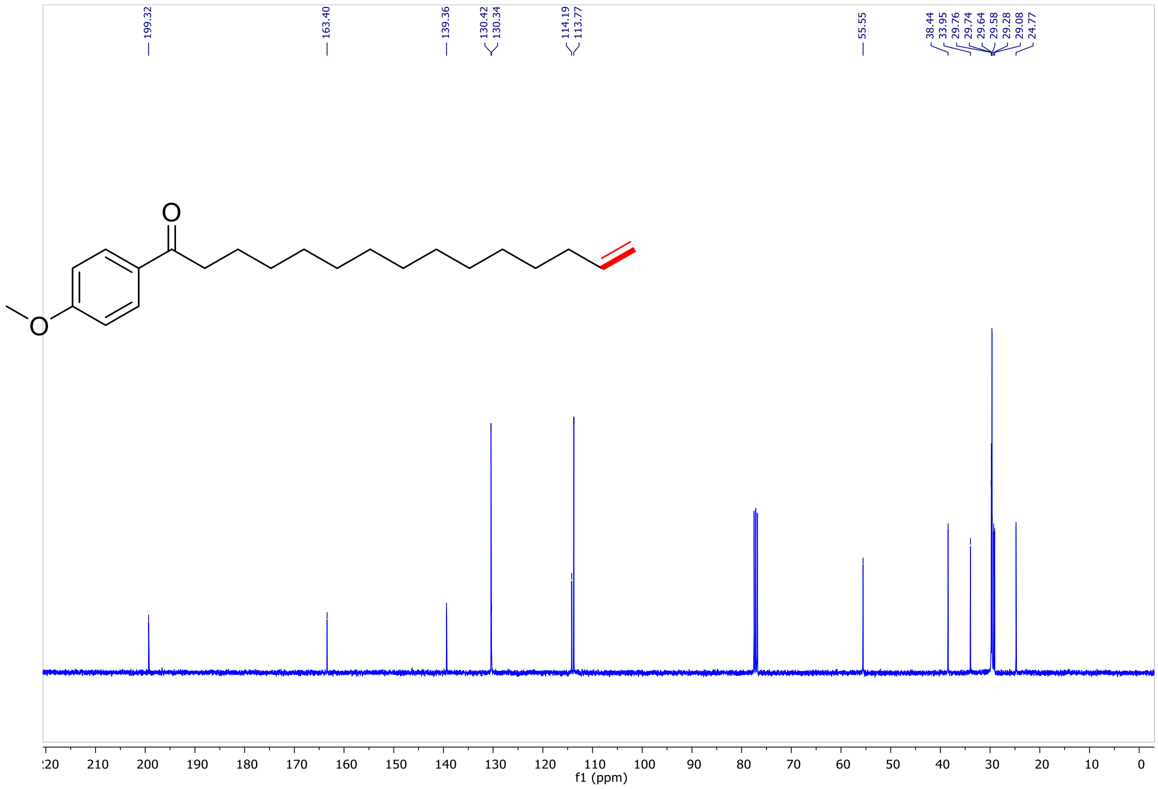

Supplementary Figure 37. ^{13}C NMR spectra of compound of **2h**

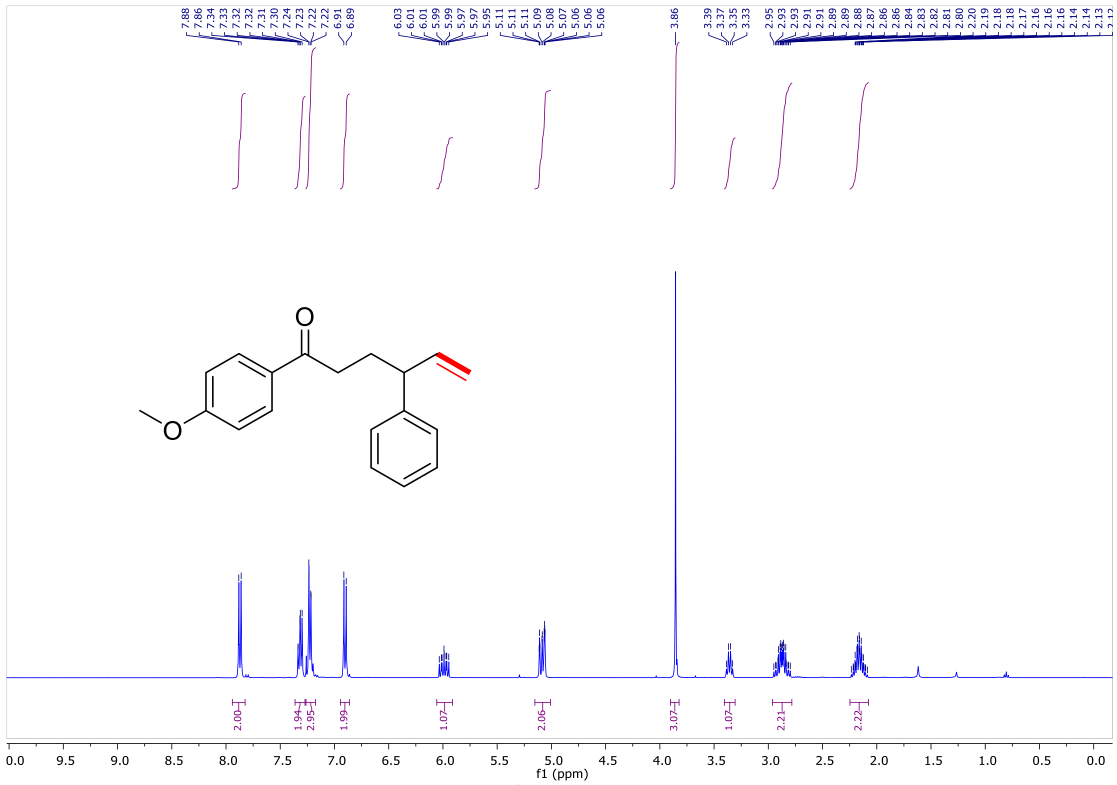

Supplementary Figure 38. ^{1}H NMR spectra of compound of **2i**

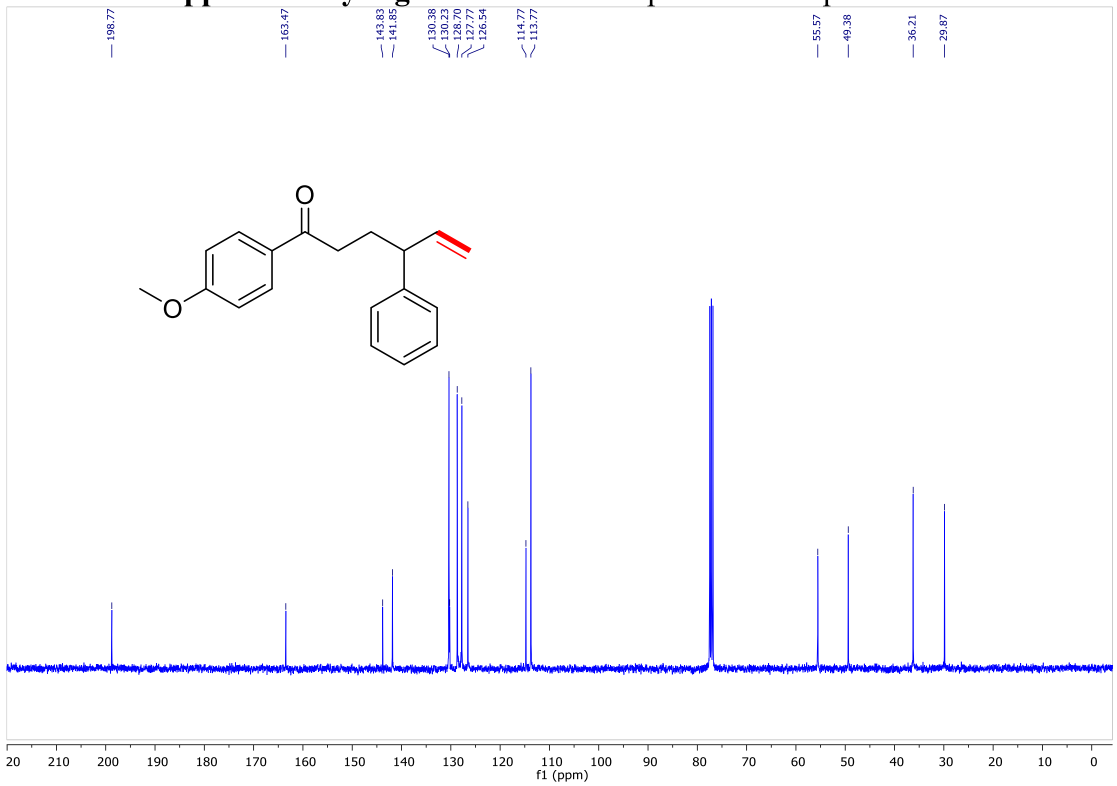

Supplementary Figure 39. ^{13}C NMR spectra of compound of **2i**

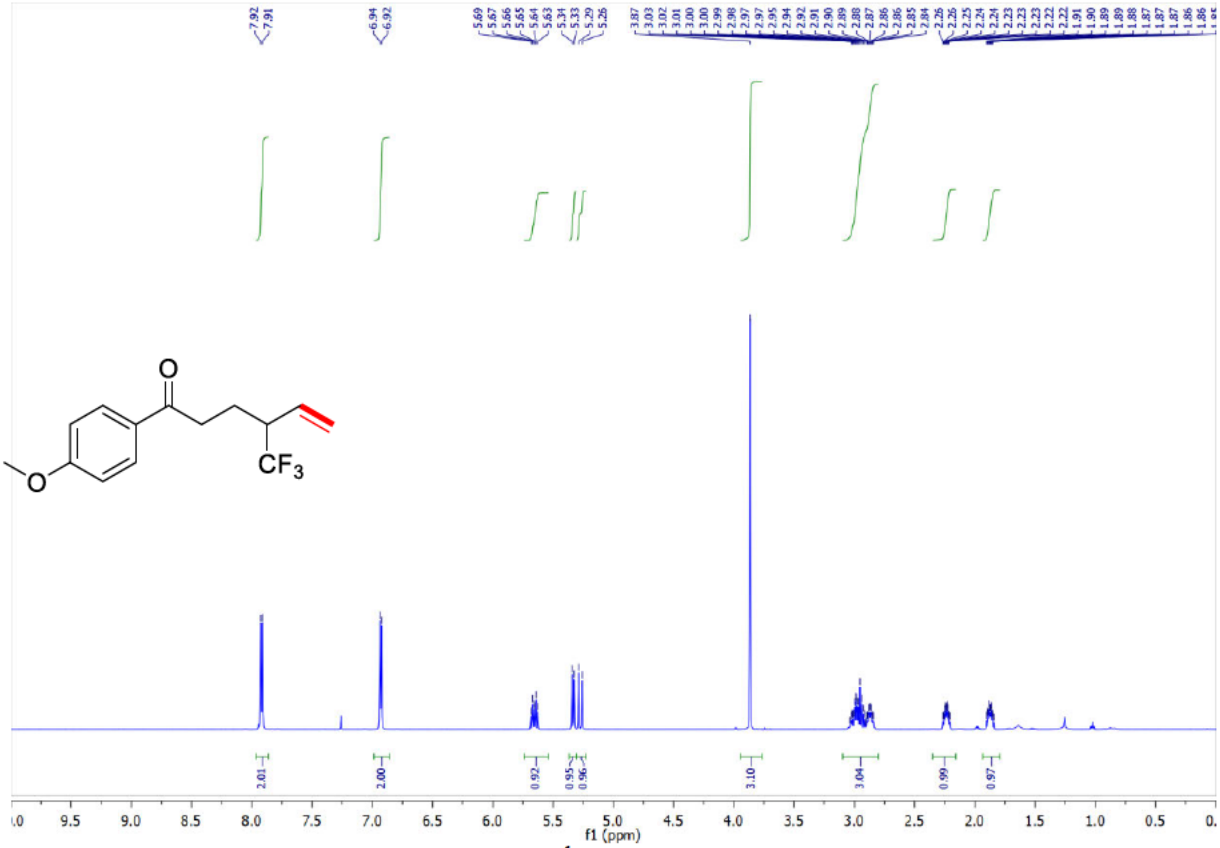

Supplementary Figure 40. ^{1}H NMR spectra of compound of **2j**

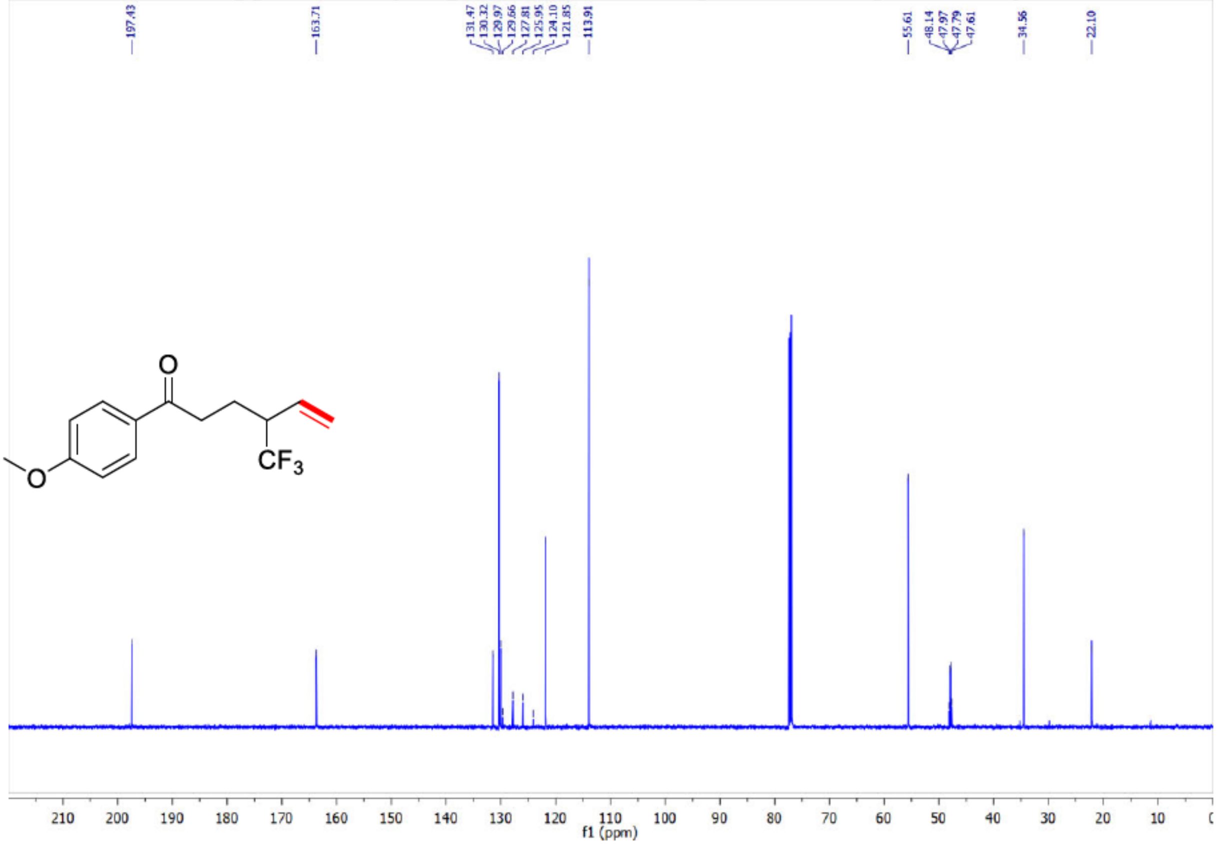

Supplementary Figure 41. ^{13}C NMR spectra of compound of **2j**

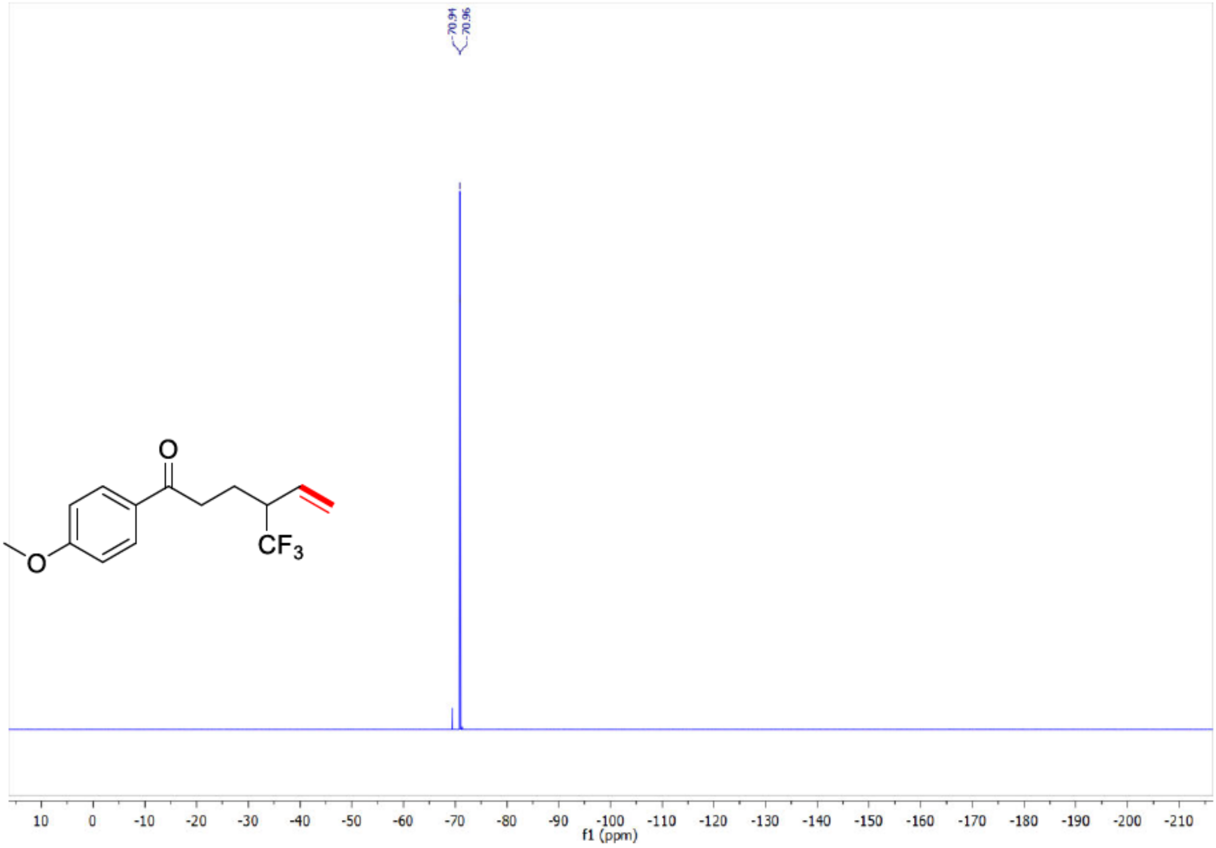

Supplementary Figure 42. ^{19}F NMR spectra of compound of **2j**

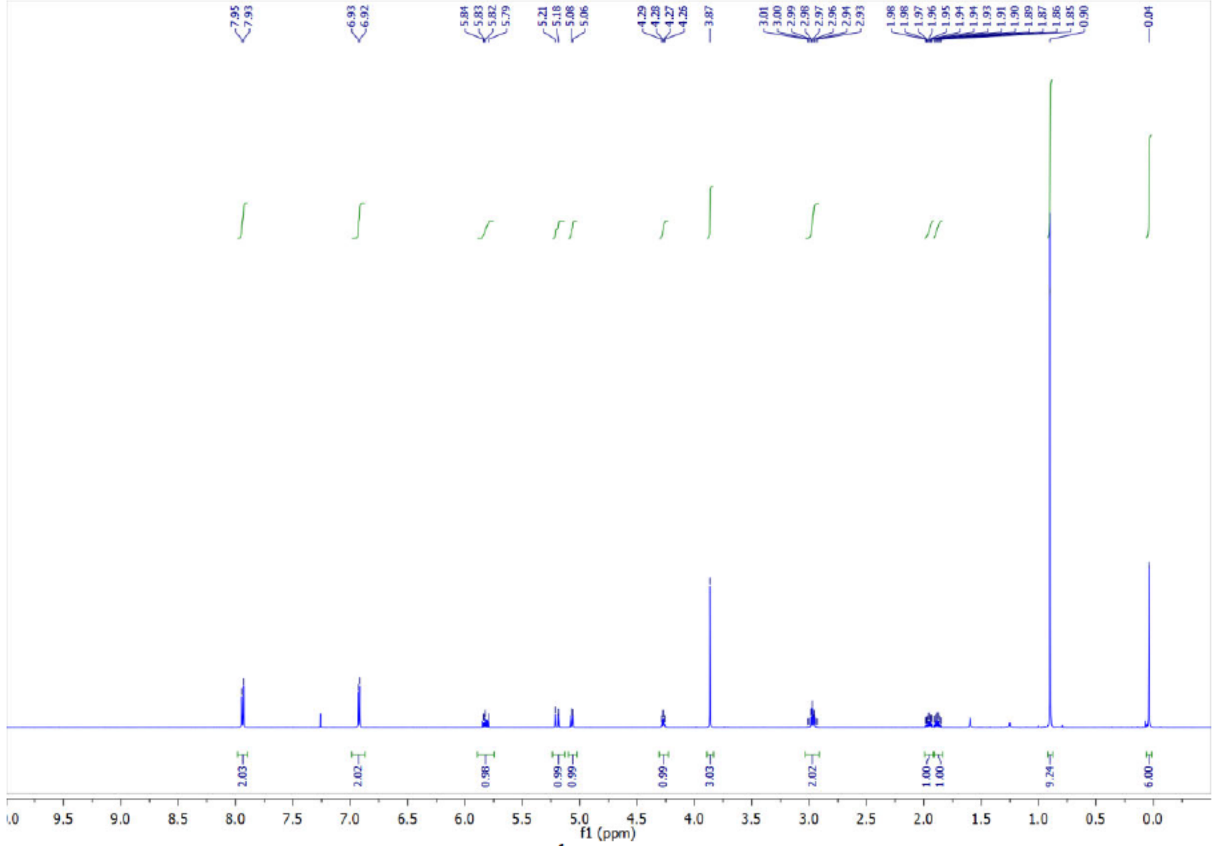

Supplementary Figure 43. ^{1}H NMR spectra of compound of **2k**

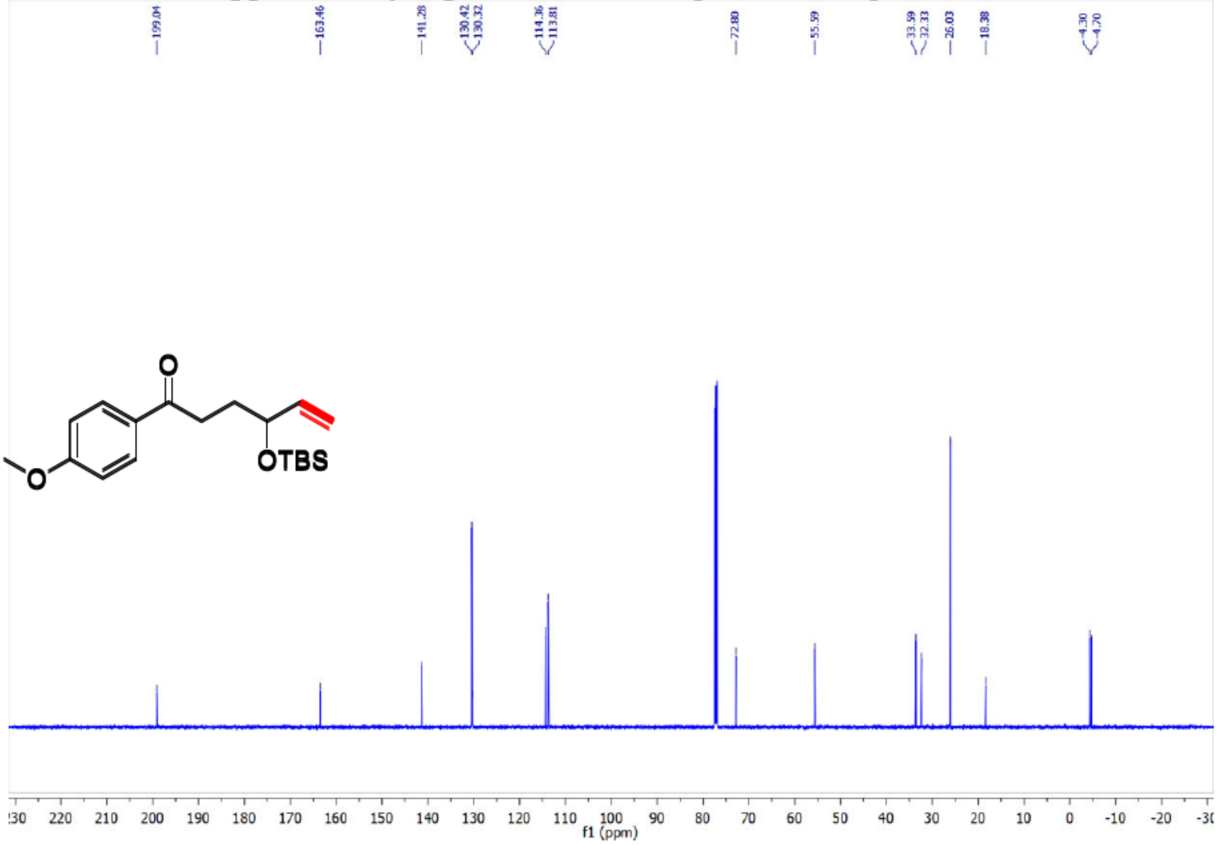

Supplementary Figure 44. ^{13}C NMR spectra of compound of **2k**

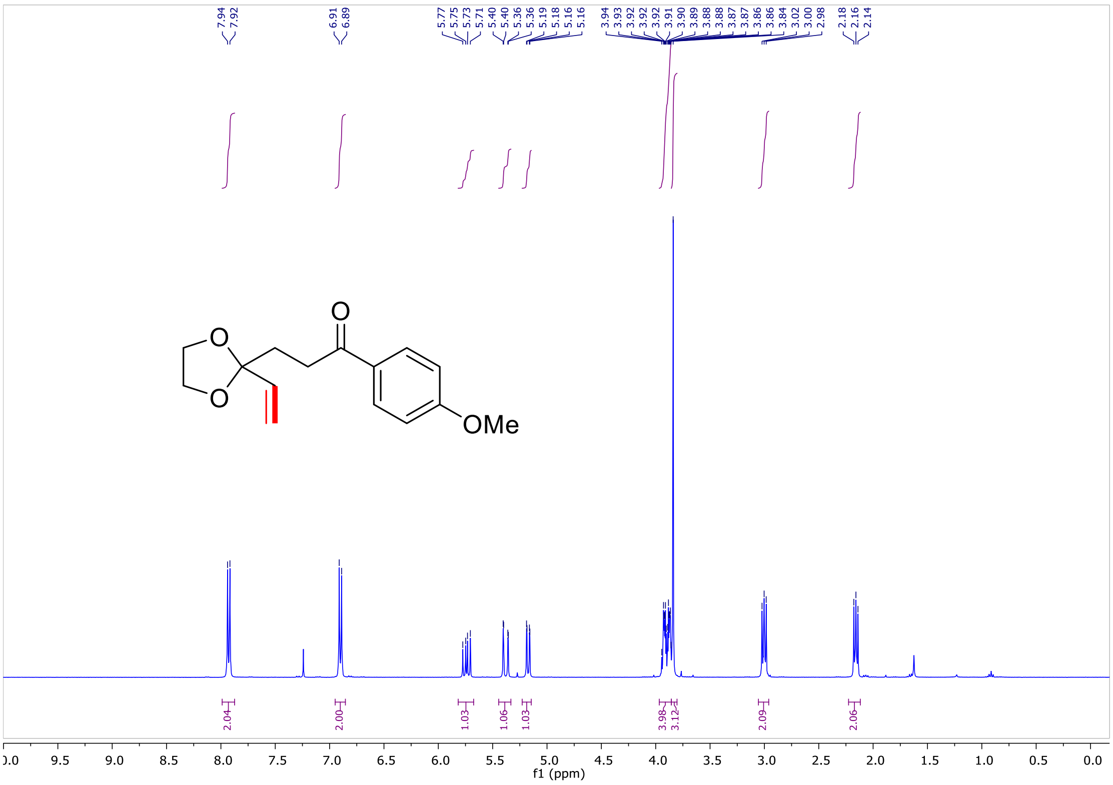

Supplementary Figure 45. ^{1}H NMR spectra of compound of **2l**

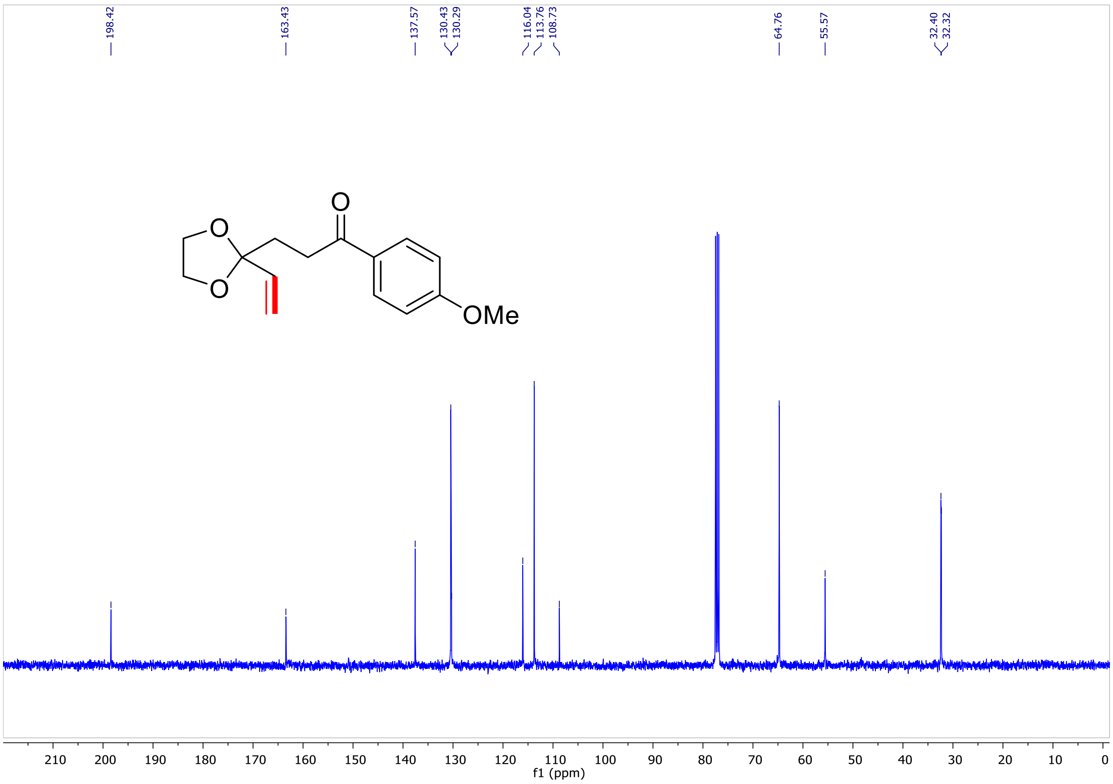

Supplementary Figure 46. ^{13}C NMR spectra of compound of **2l**

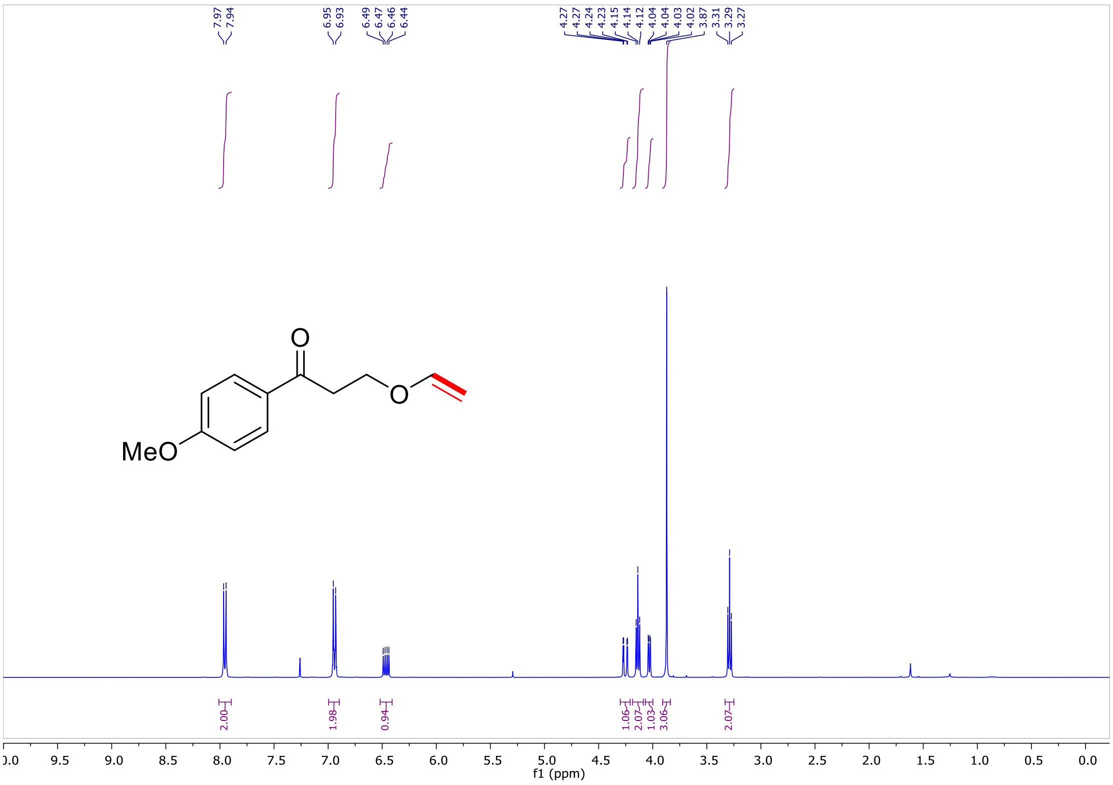

Supplementary Figure 47. ^{1}H NMR spectra of compound of **2m**

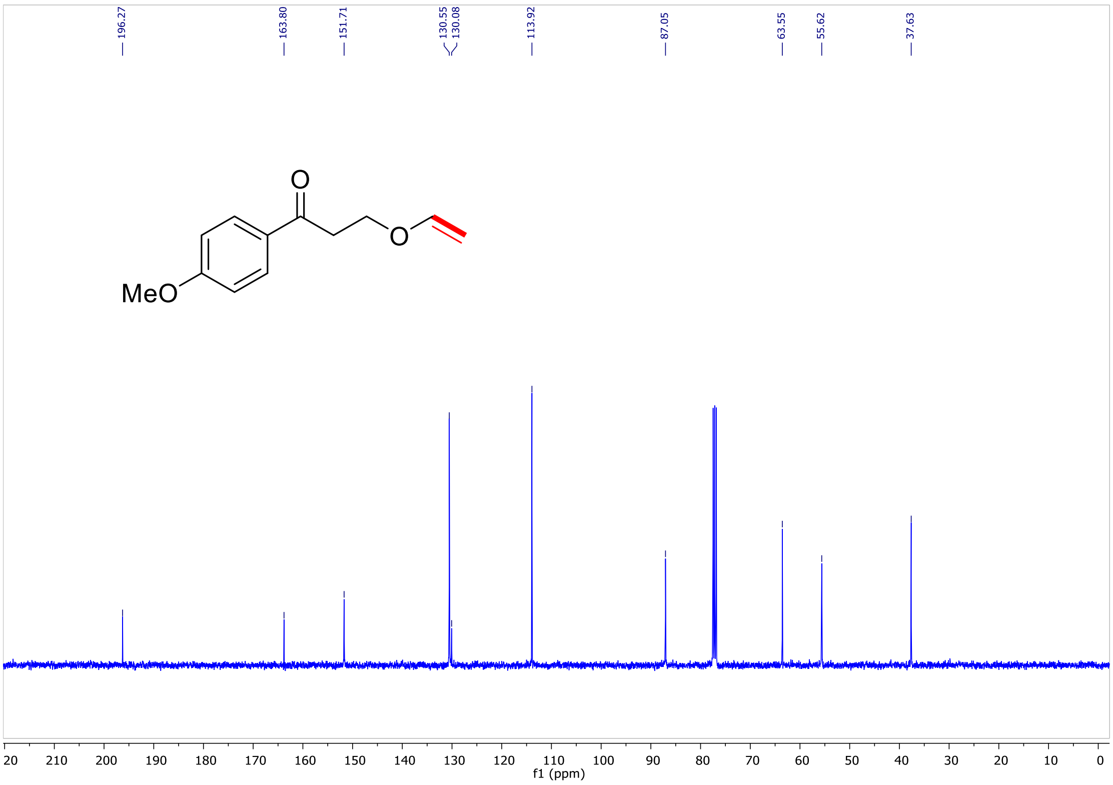

Supplementary Figure 48. ^{13}C NMR spectra of compound of **2m**

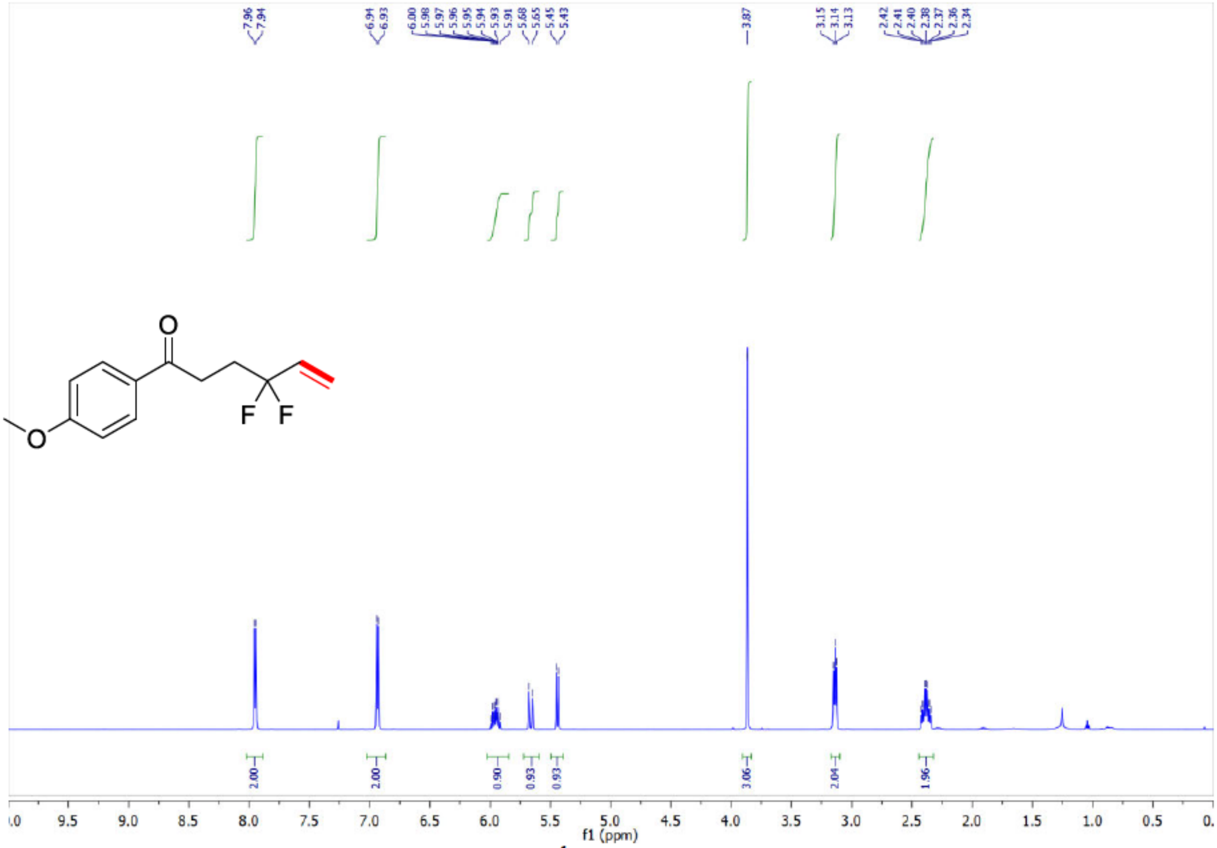

Supplementary Figure 49. ^{1}H NMR spectra of compound of **2n**

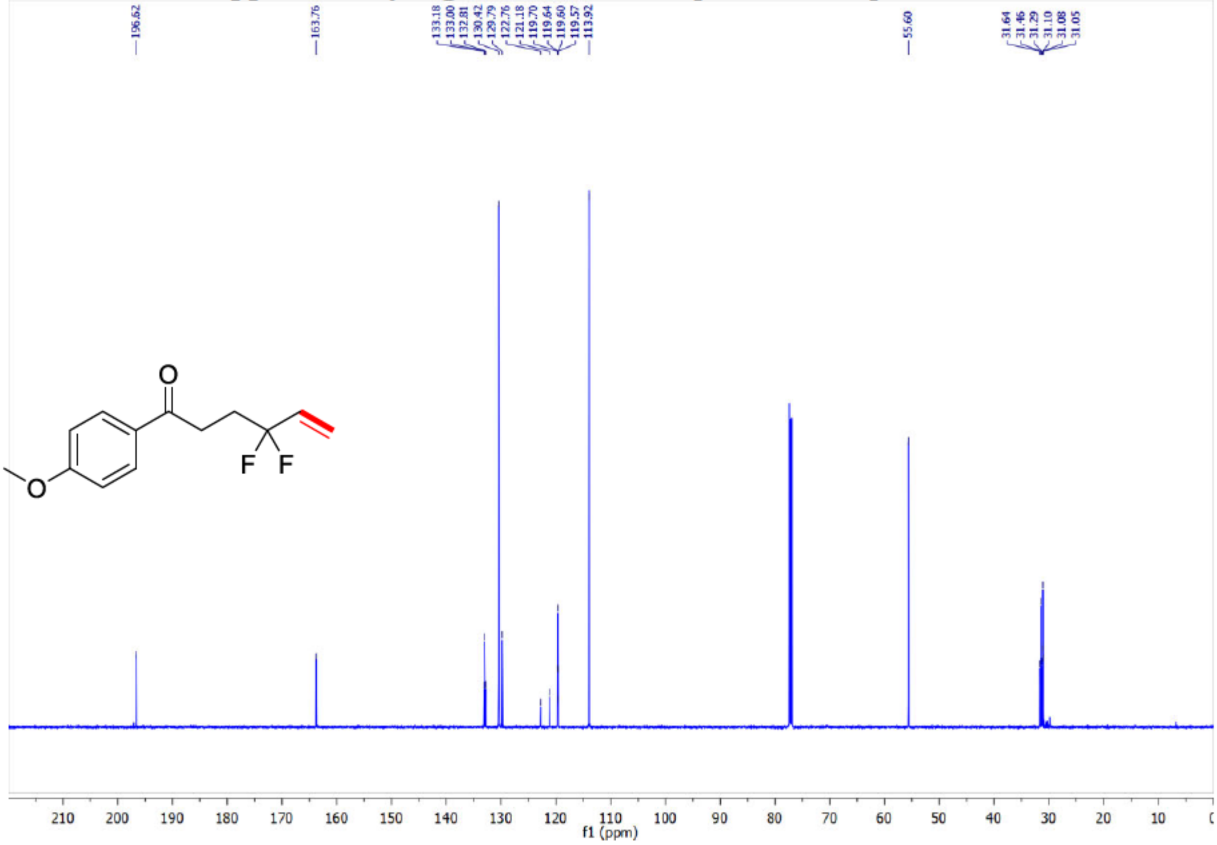

Supplementary Figure 50. ^{13}C NMR spectra of compound of **2n**

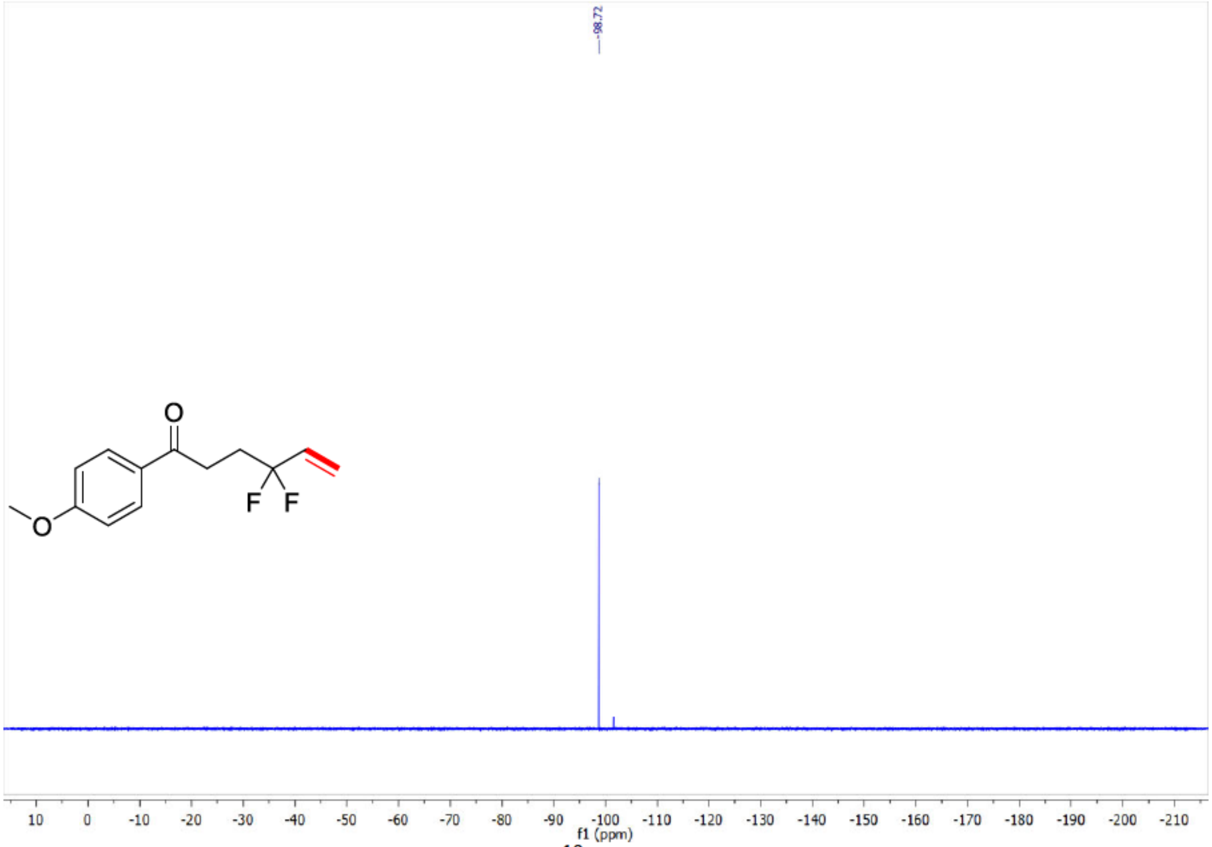

Supplementary Figure 51. ^{19}F NMR spectra of compound of **2n**

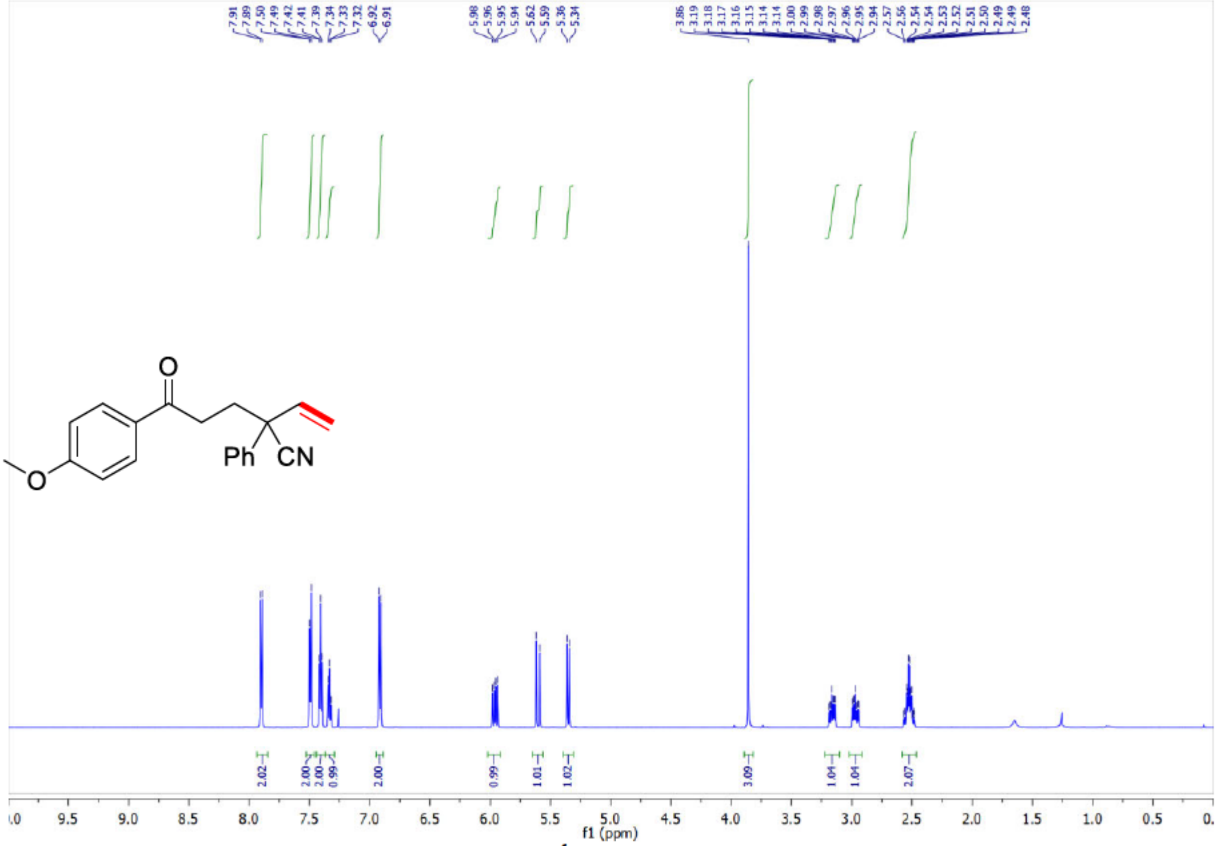

Supplementary Figure 52. ^{1}H NMR spectra of compound of **2o**

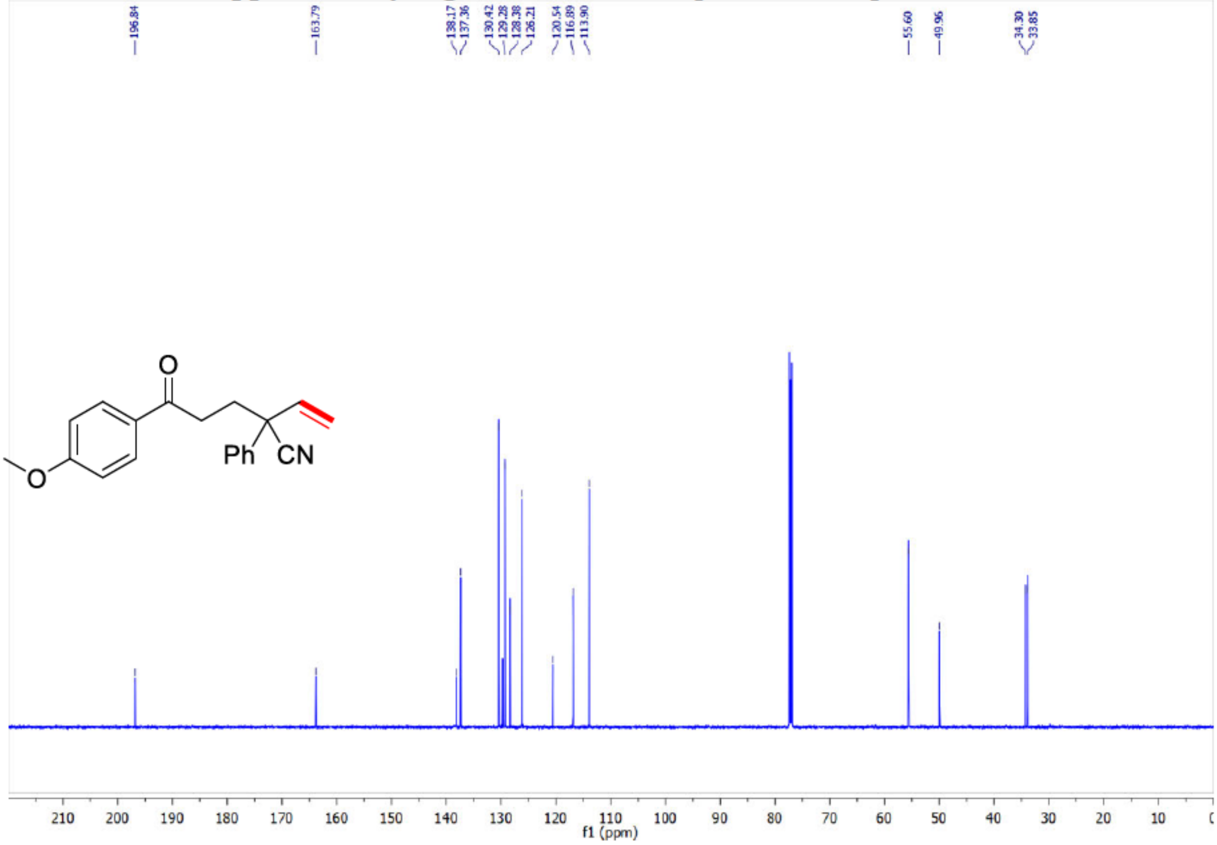

Supplementary Figure 53. ^{13}C NMR spectra of compound of **2o**

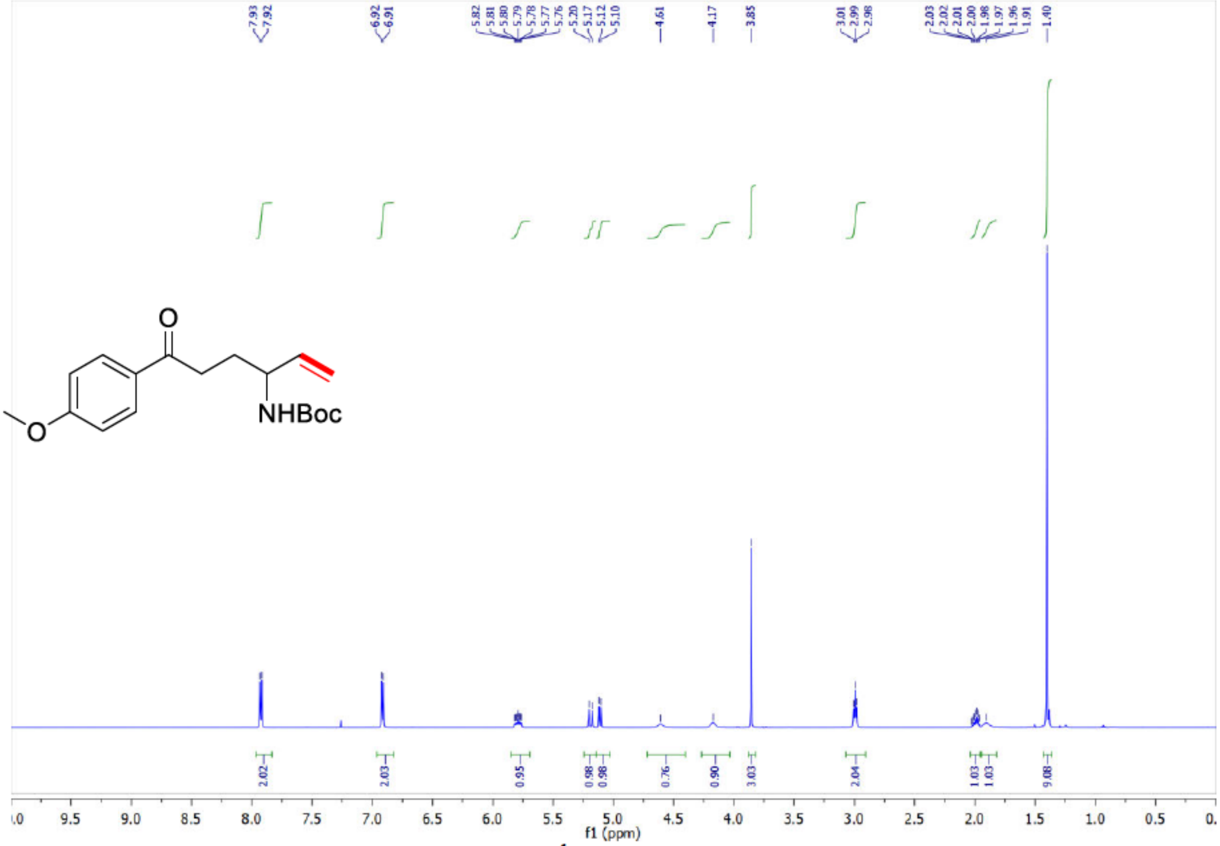

Supplementary Figure 54. ^{1}H NMR spectra of compound of **2p**

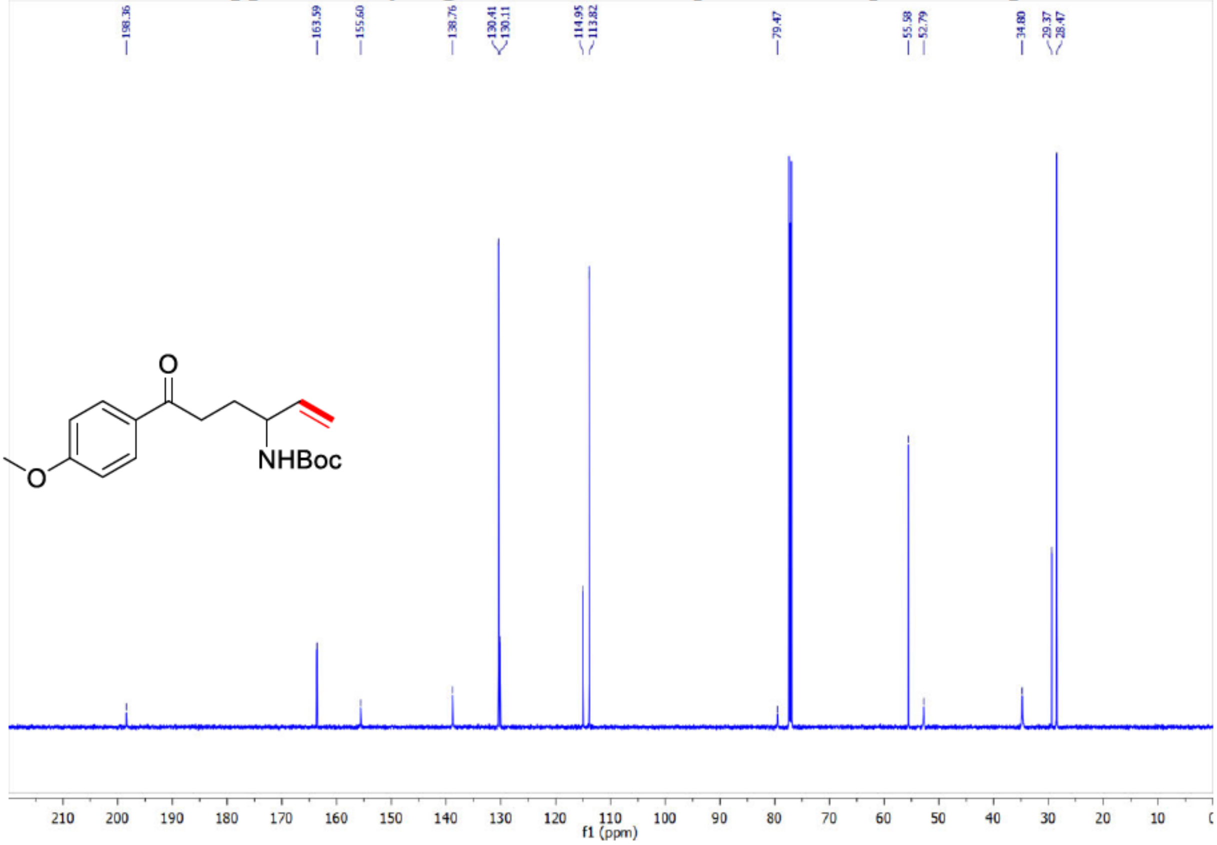

Supplementary Figure 55. ^{13}C NMR spectra of compound of **2p**

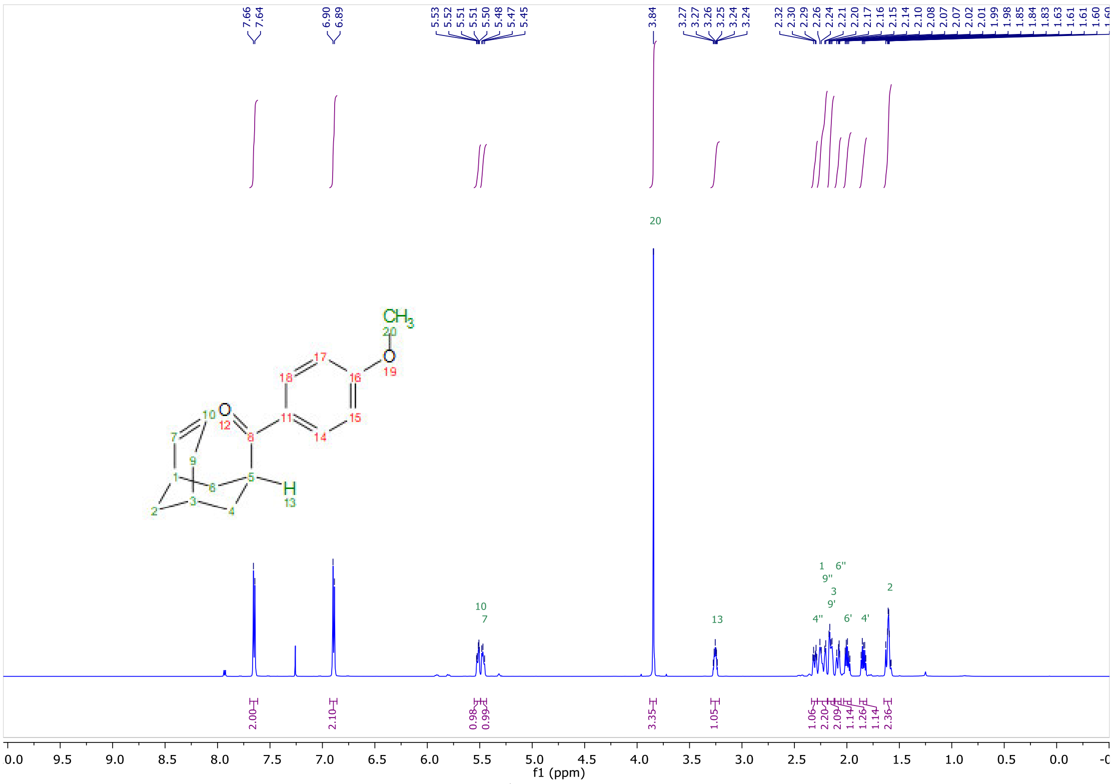

Supplementary Figure 56. ^{1}H NMR spectra of compound of **2q**

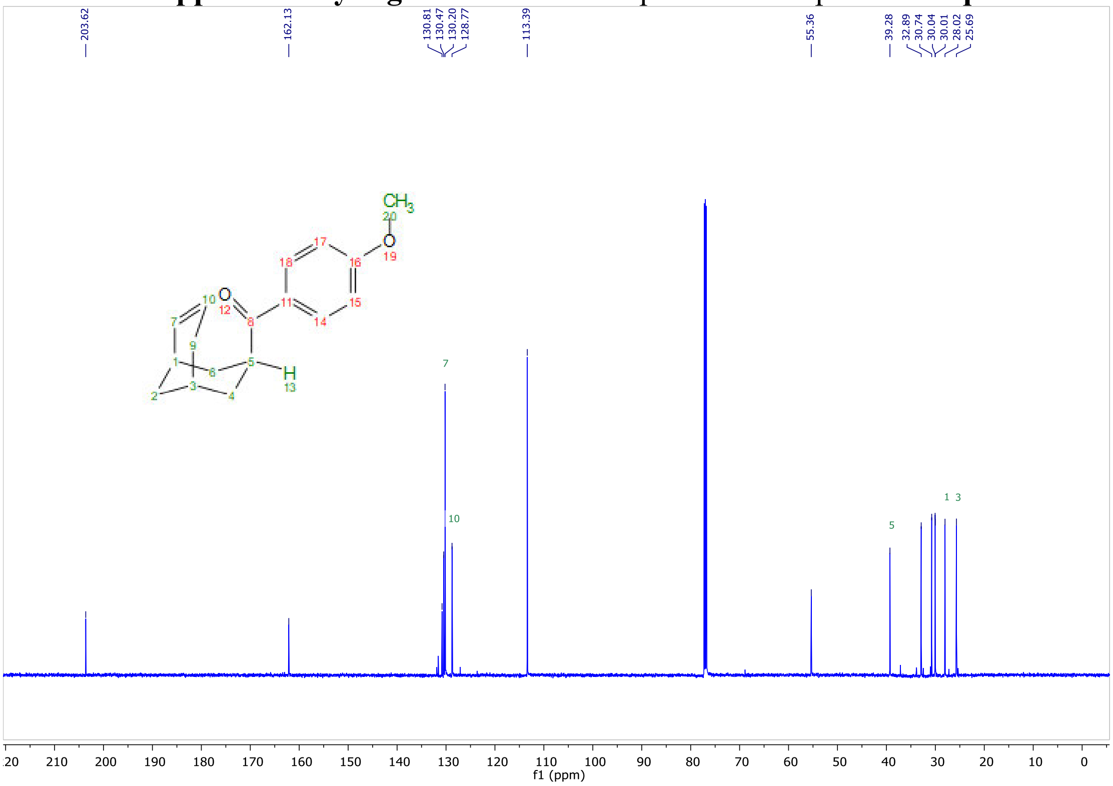

Supplementary Figure 57. ^{13}C NMR spectra of compound of **2q**

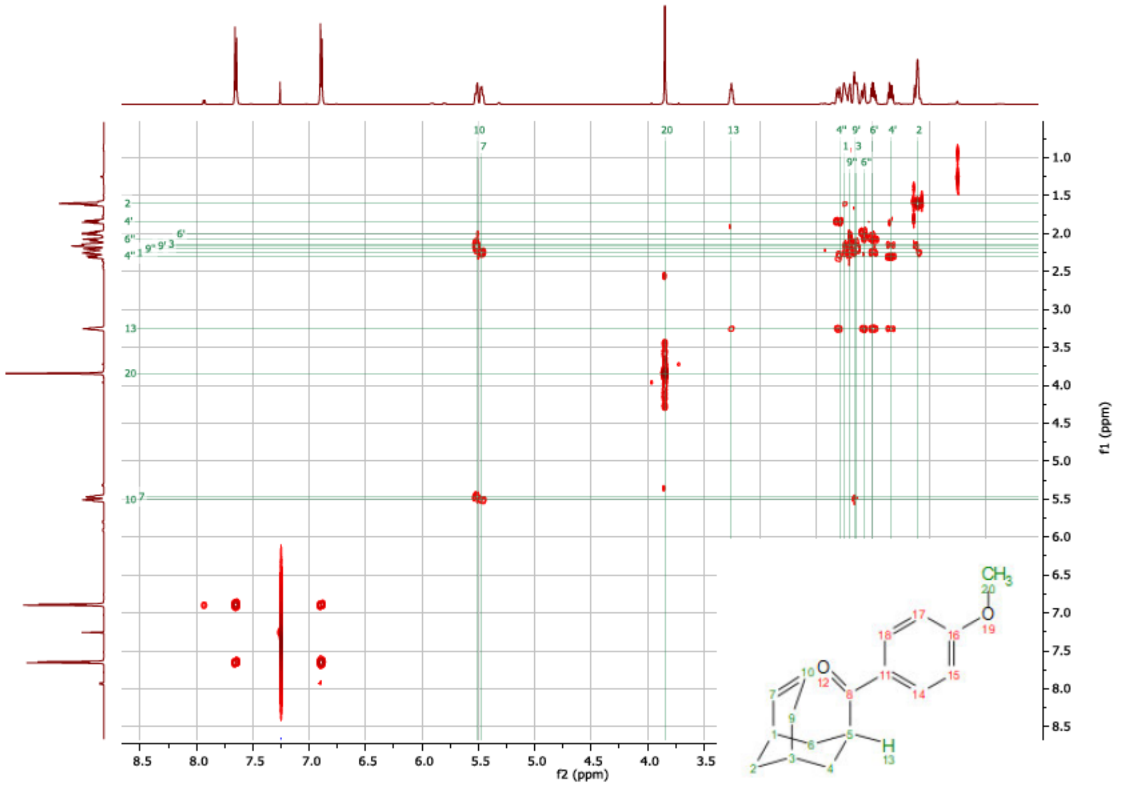

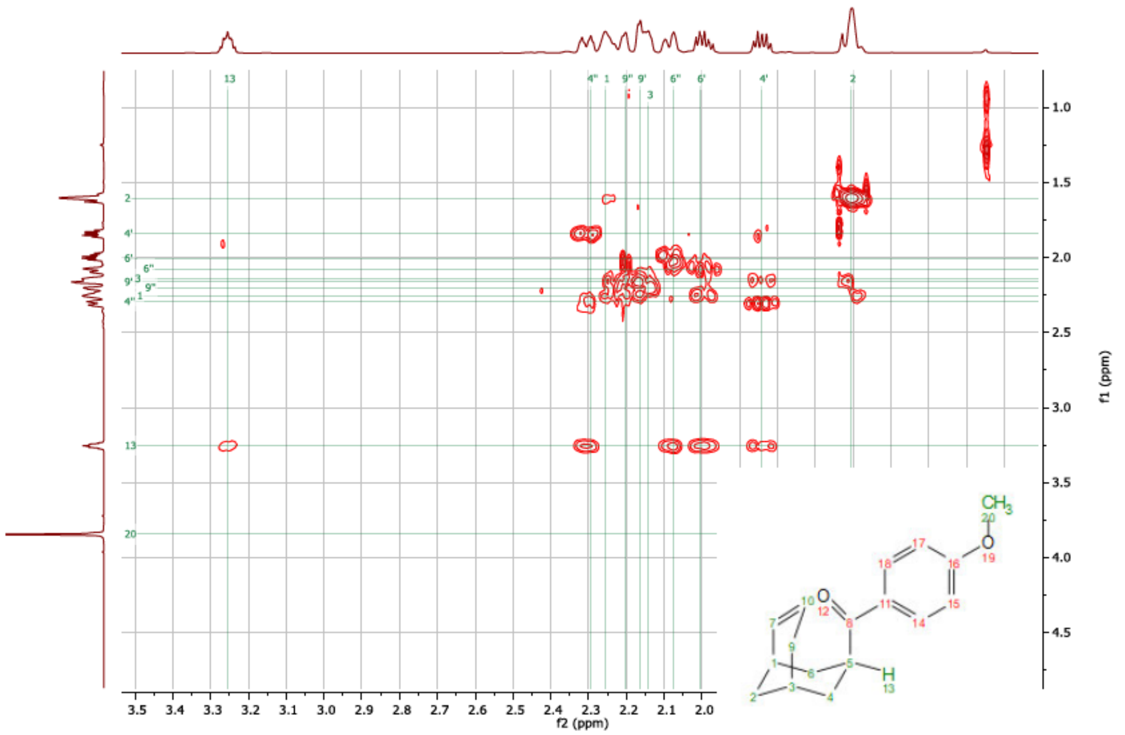

Supplementary Figure 58. ^{1}H-^{1}H COSY spectra of compound of **2q**

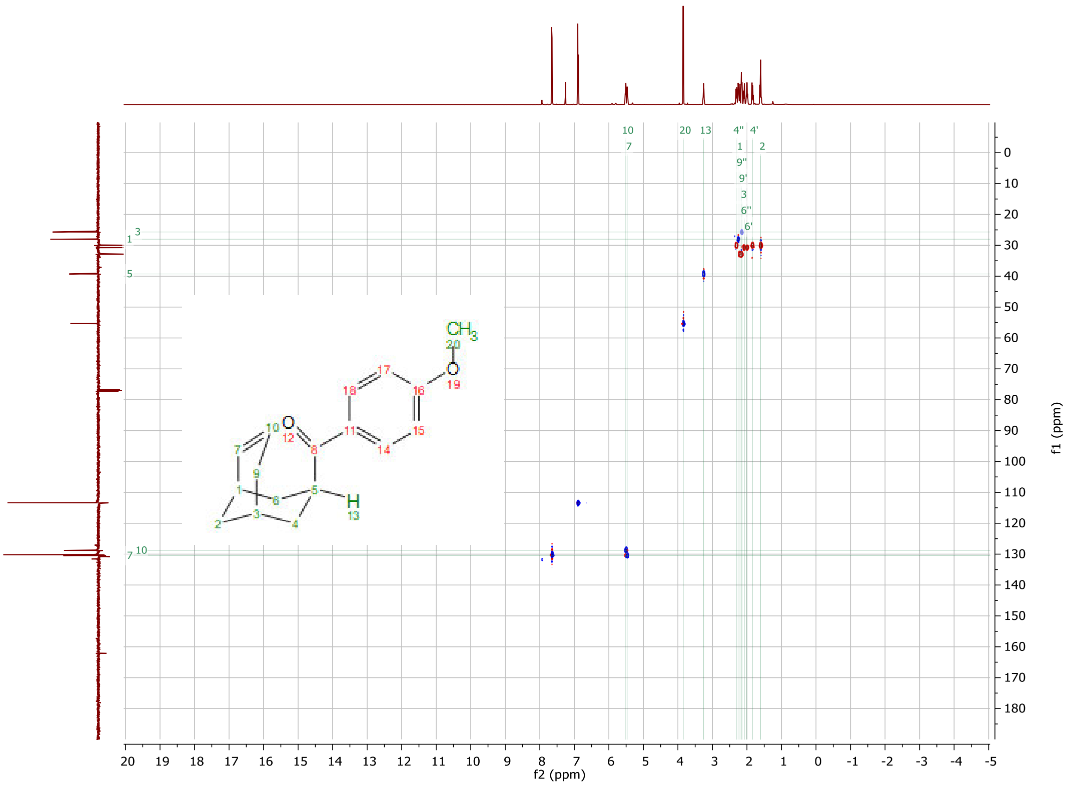

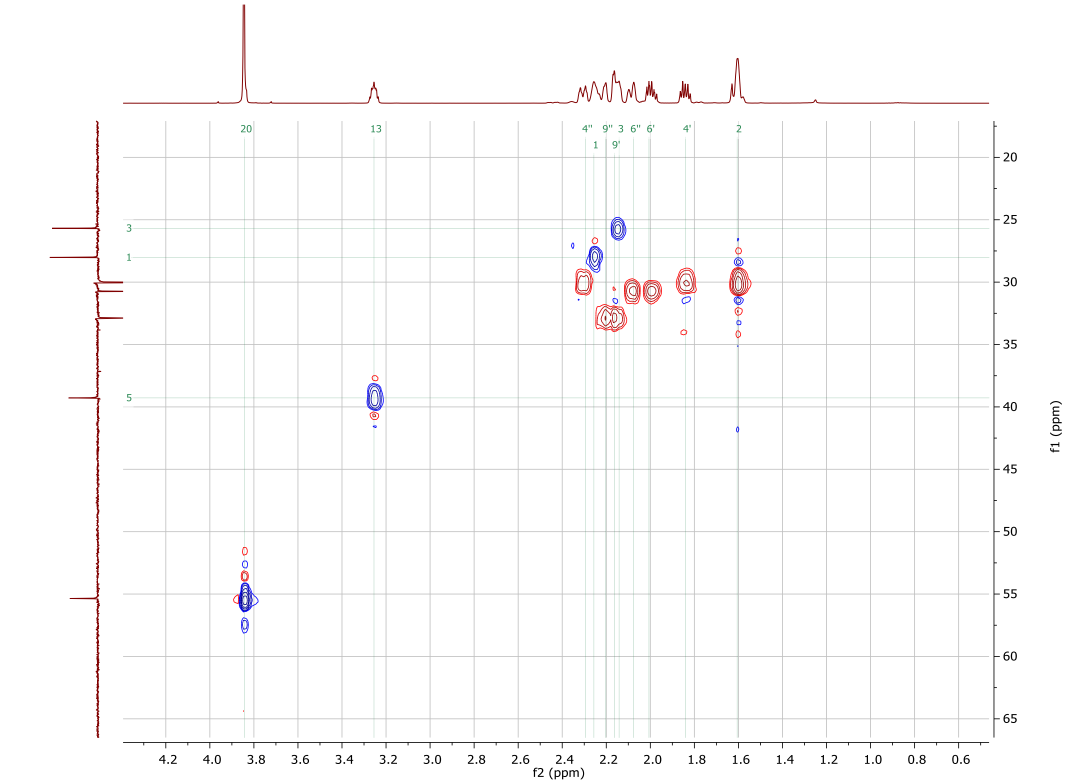

Supplementary Figure 59. ^{1}H-^{13}C HSQC spectra of compound of **2q**

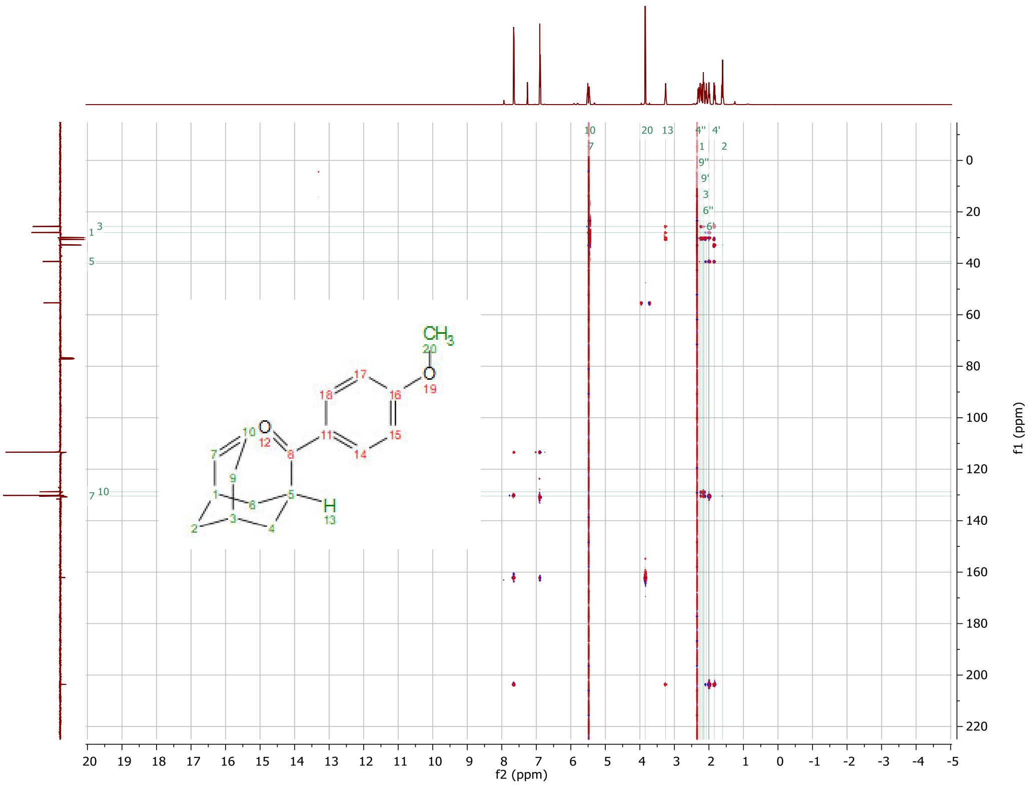

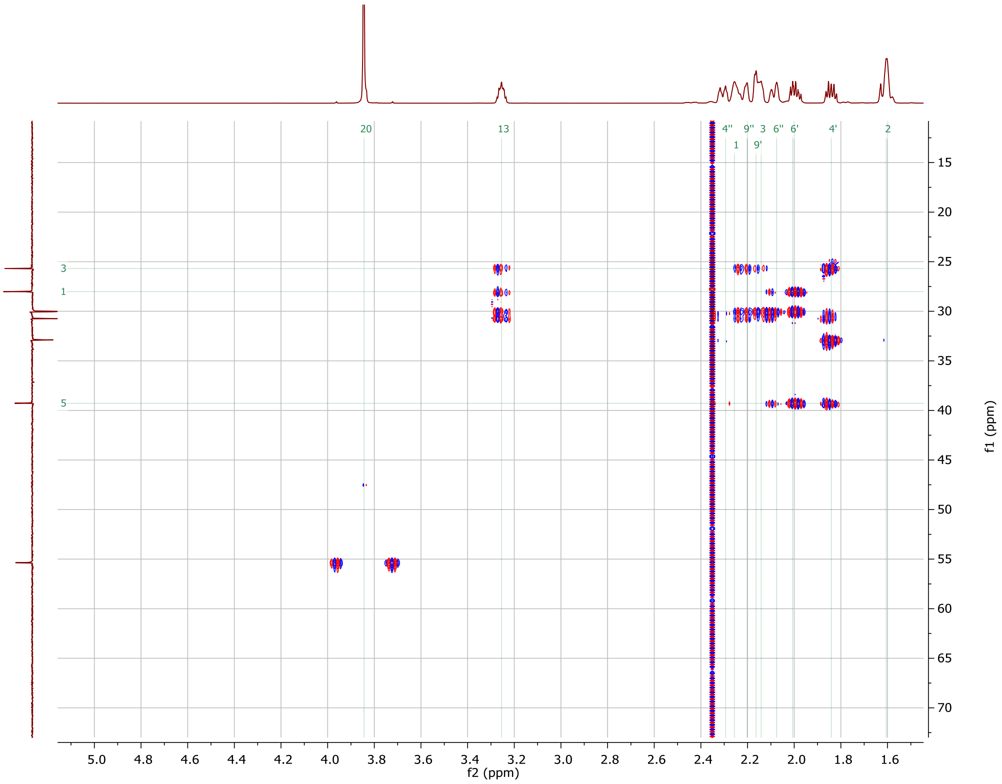

Supplementary Figure 60. ^{1}H-^{13}C HMBC spectra of compound of **2q**

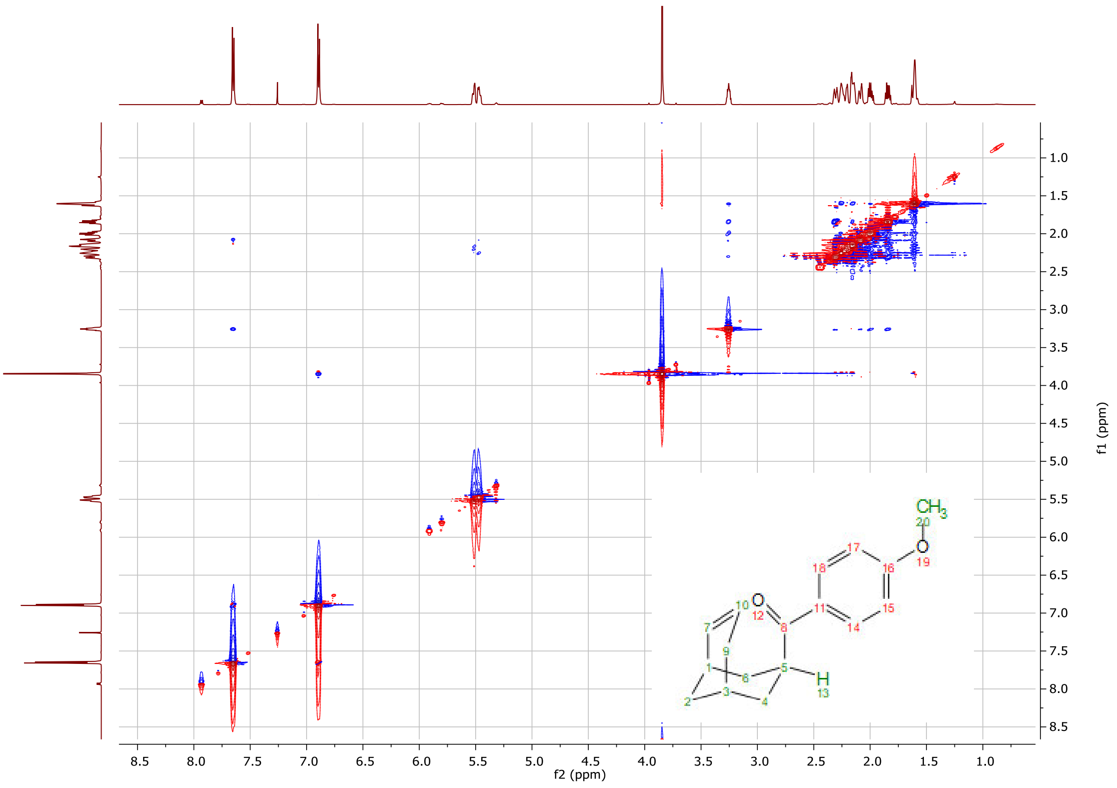

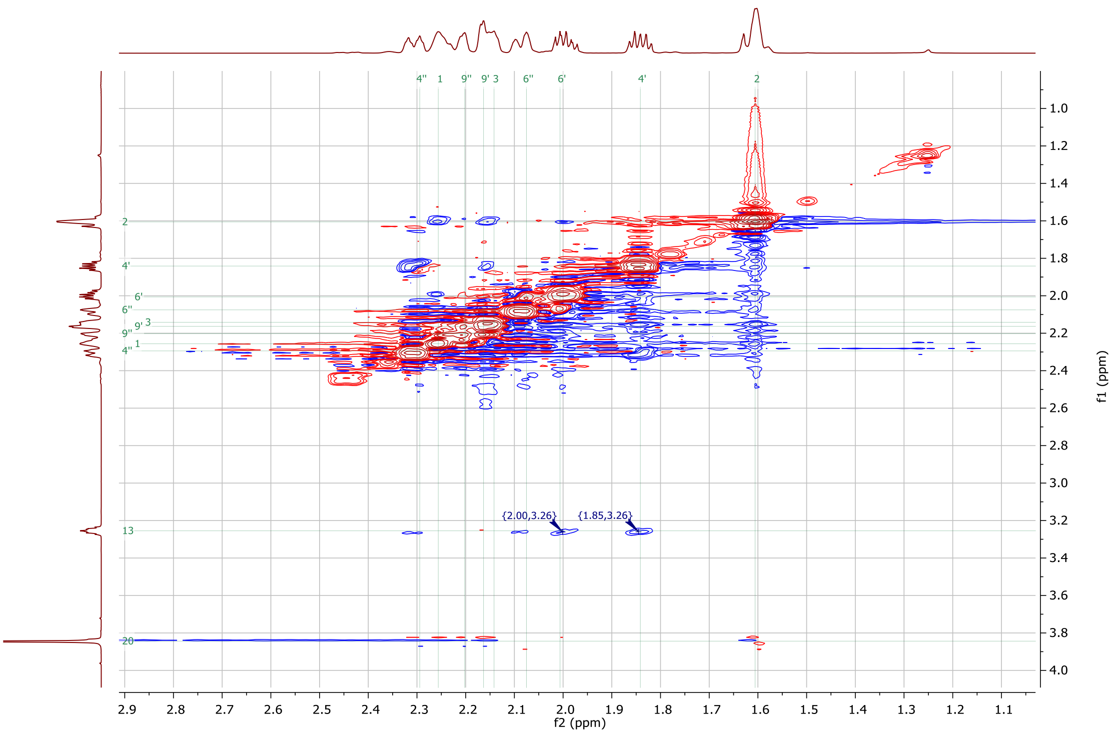

Supplementary Figure 61. ^{1}H-^{1}H NOESY spectra of compound of **2q**

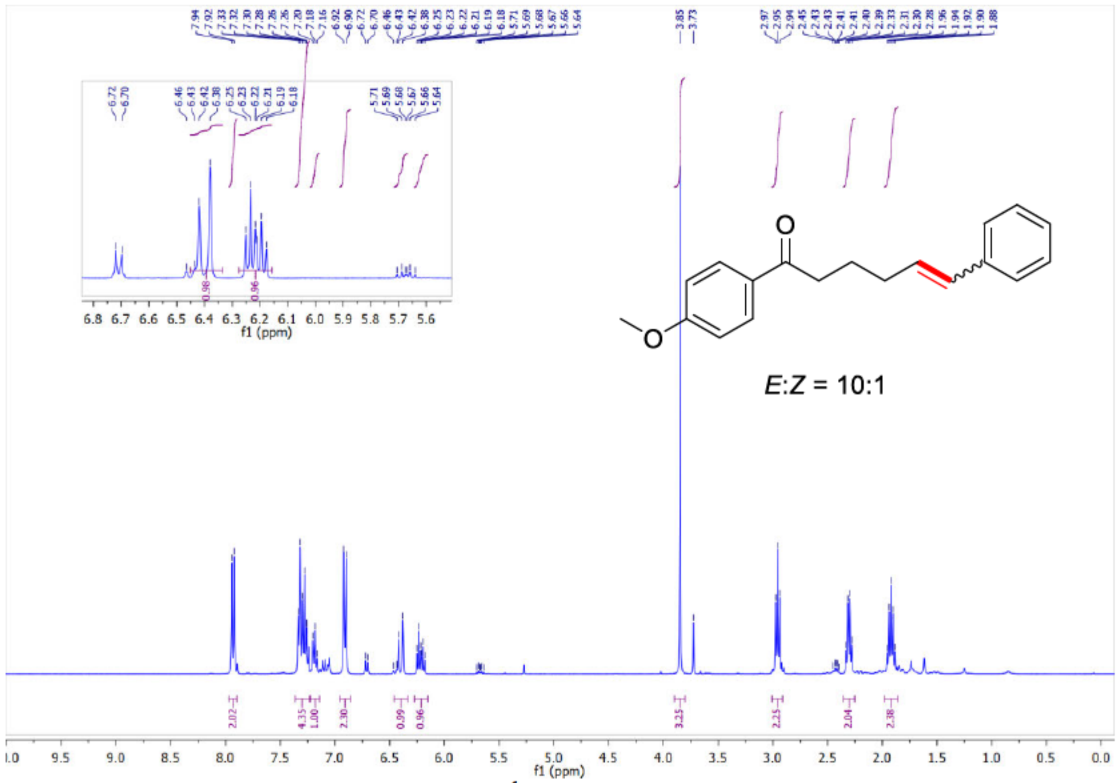

Supplementary Figure 62. ^{1}H NMR spectra of compound of **2r**

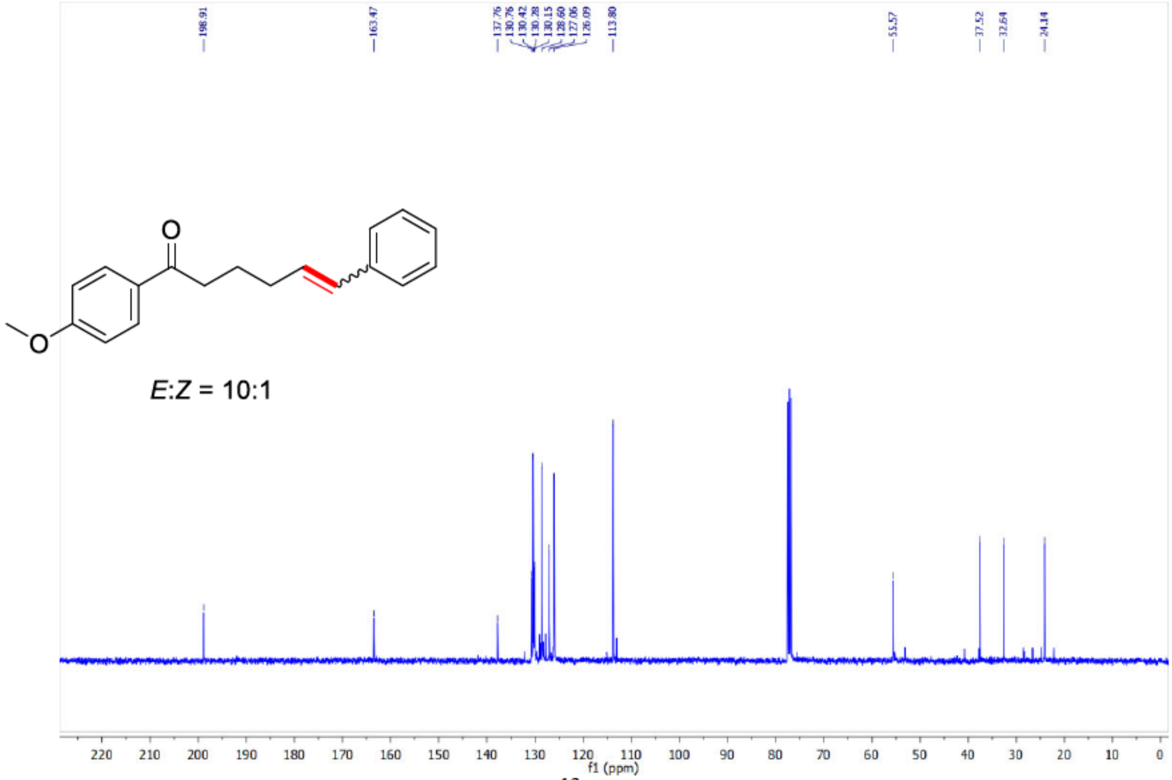

Supplementary Figure 63. ^{13}C NMR spectra of compound of **2r**

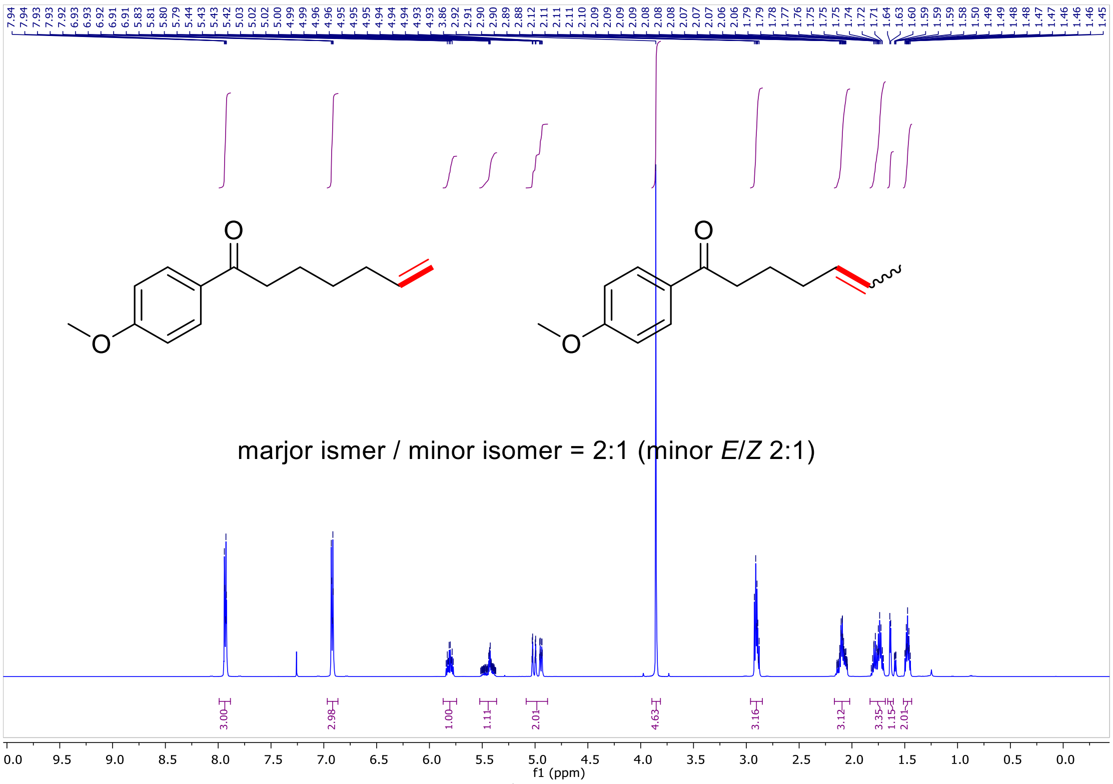

Supplementary Figure 64. ^{1}H NMR spectra of compound of **2s**

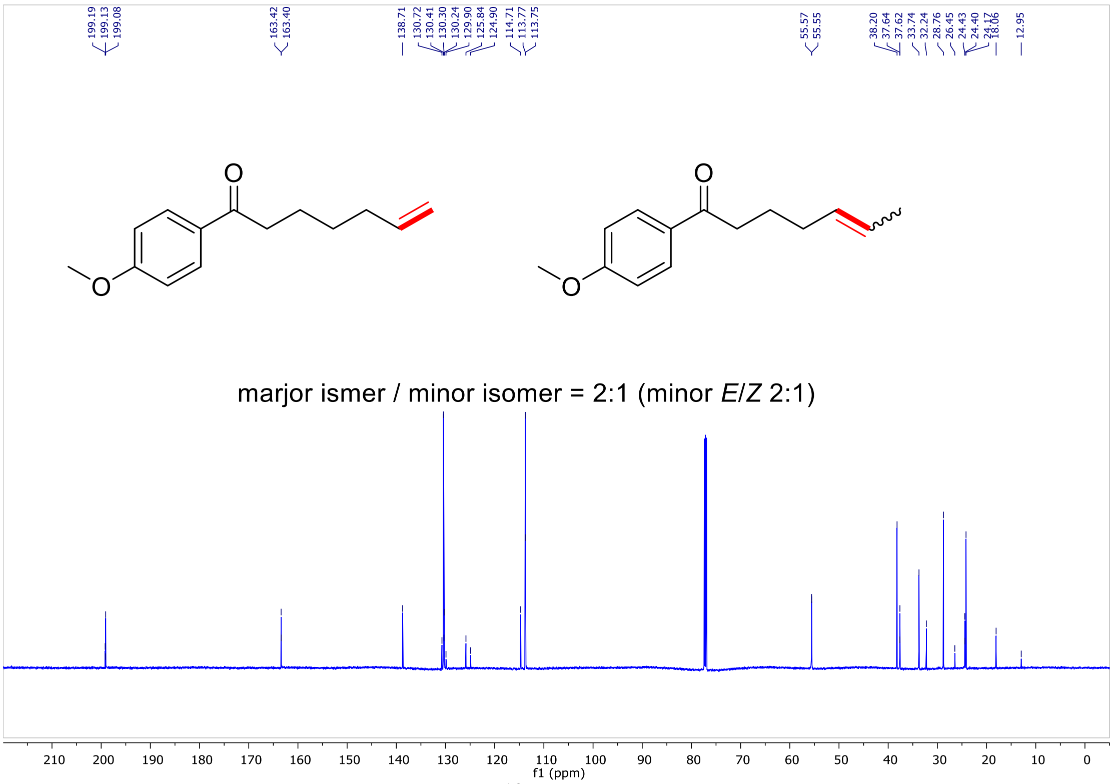

Supplementary Figure 65. ^{13}C NMR spectra of compound of **2s**

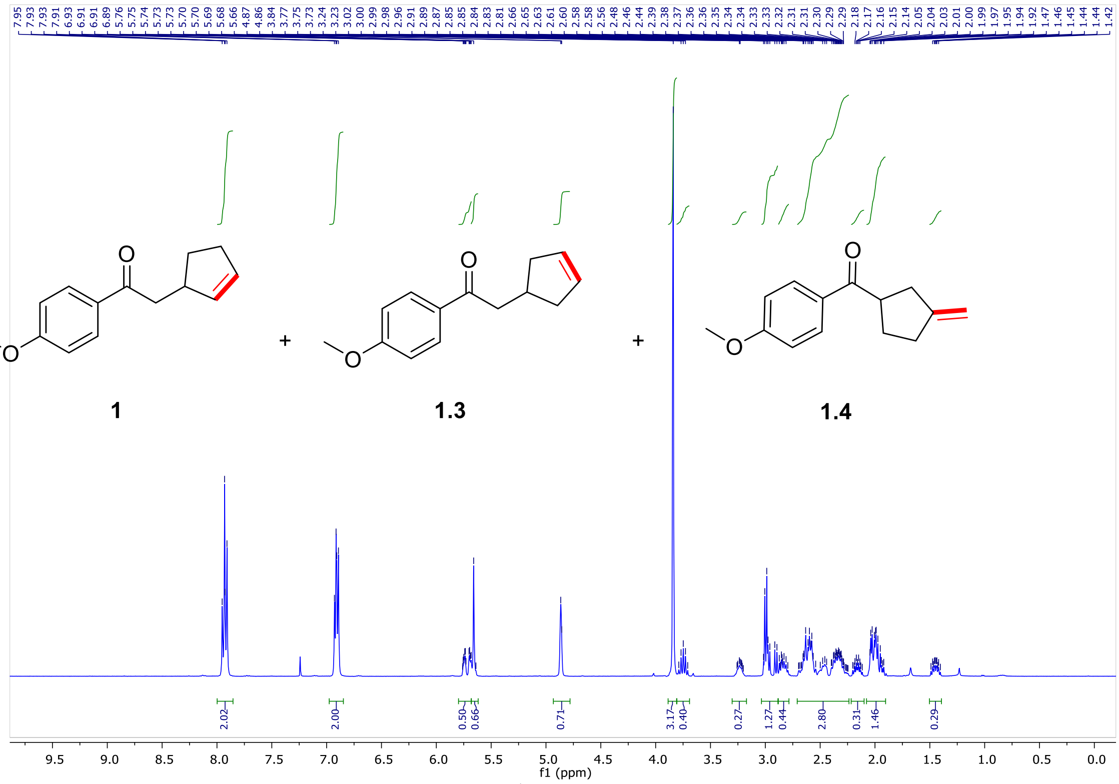

Supplementary Figure 66. ^{1}H NMR spectra of compound of **2t**

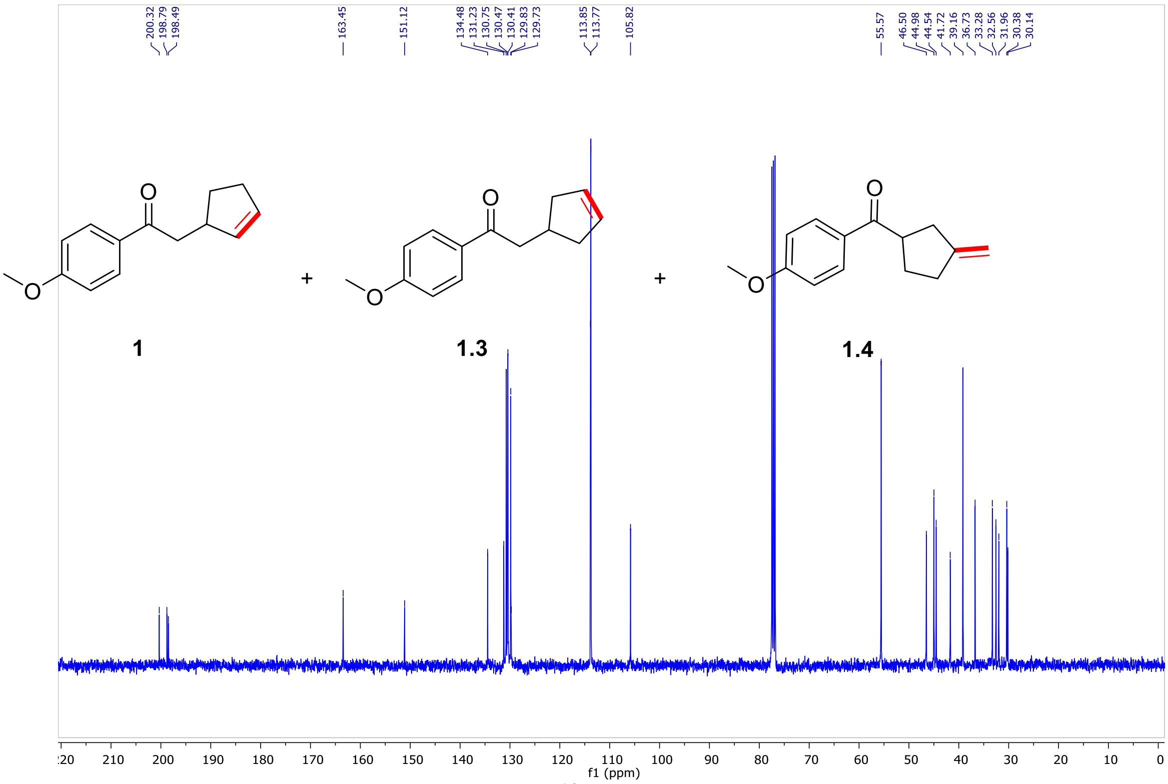

Supplementary Figure 67. ^{13}C NMR spectra of compound of **2t**

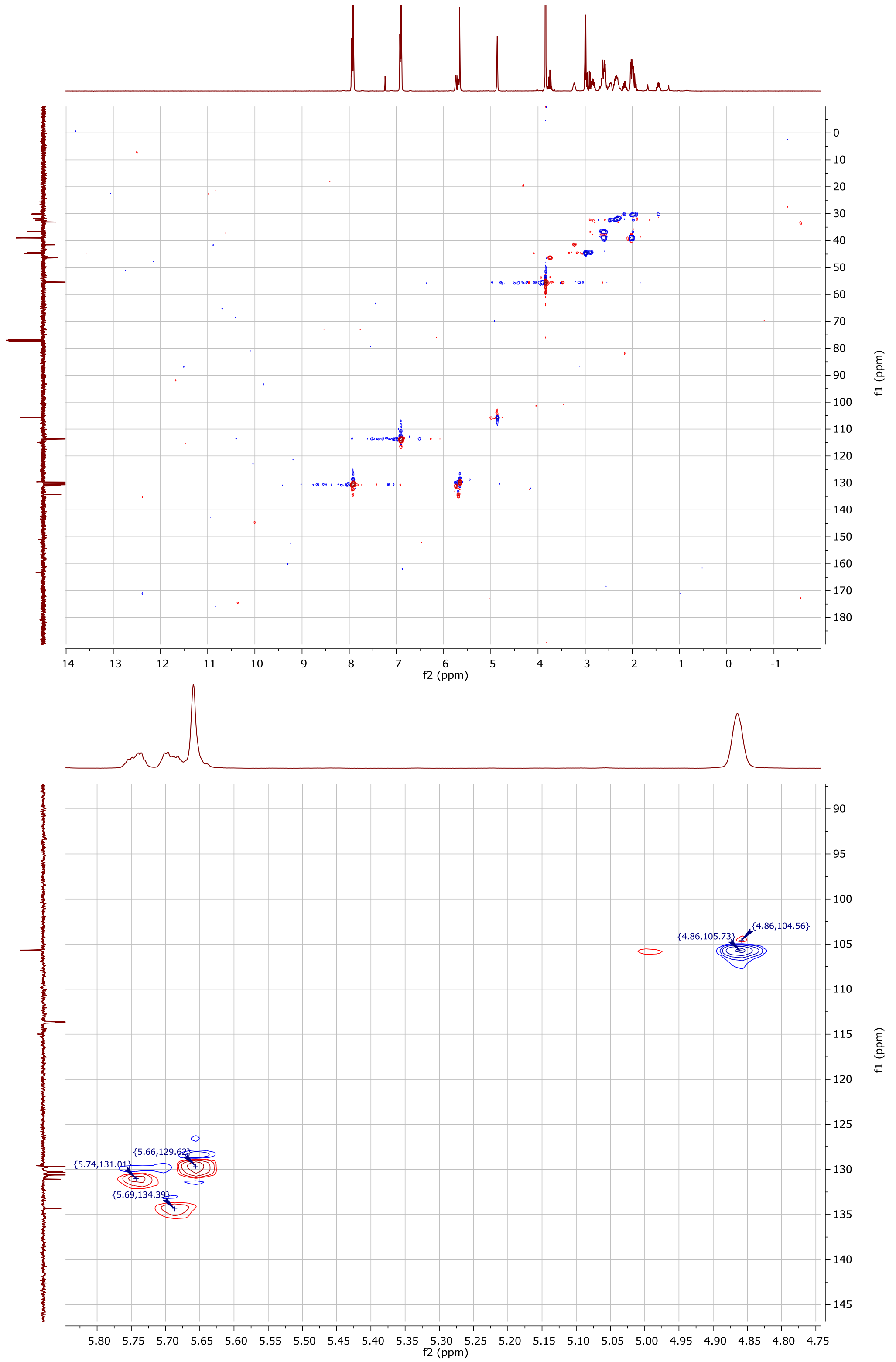

Supplementary Figure 68. ^{1}H-^{13}C HSQC spectra of compound of **2t**

HMBC

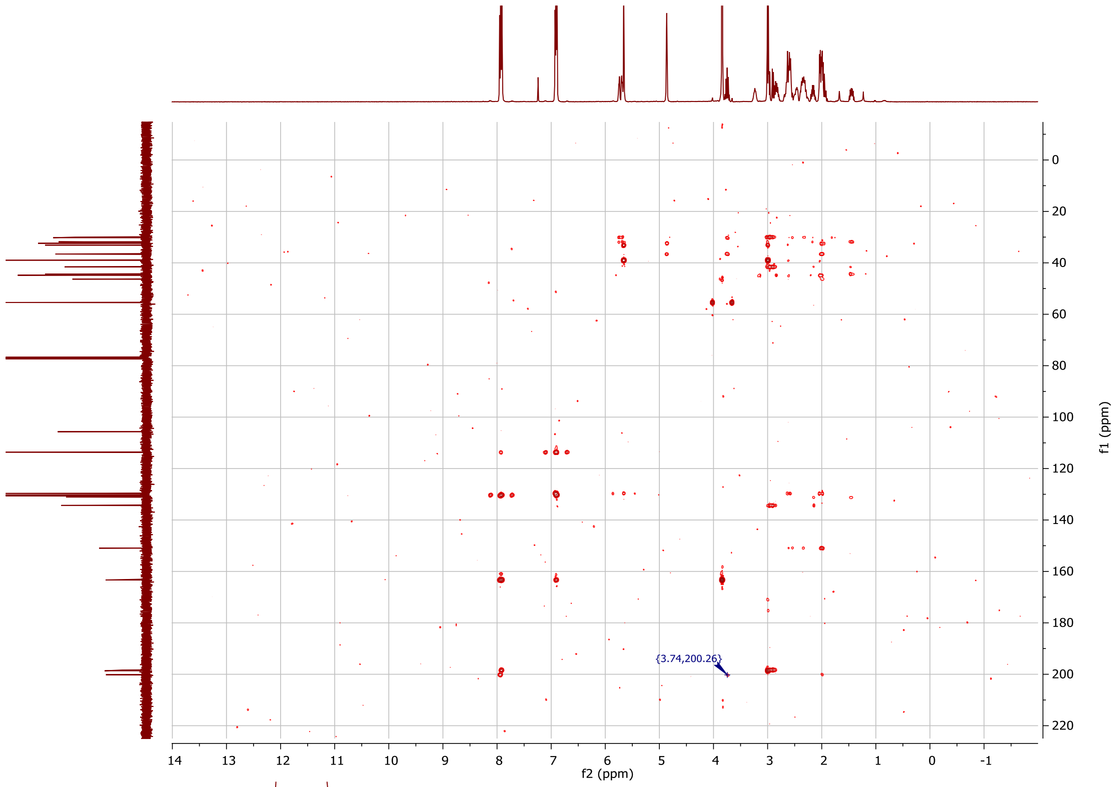

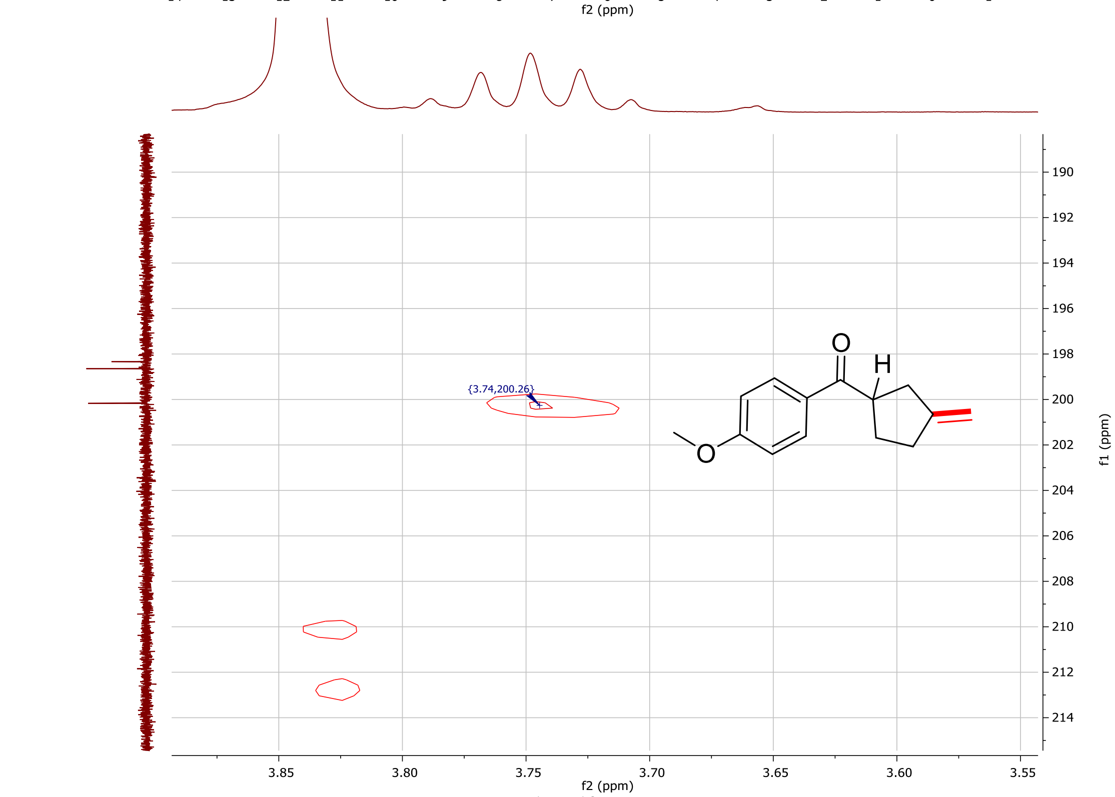

Supplementary Figure 69. ^{1}H-^{13}C HMBC spectra of compound of **2t**

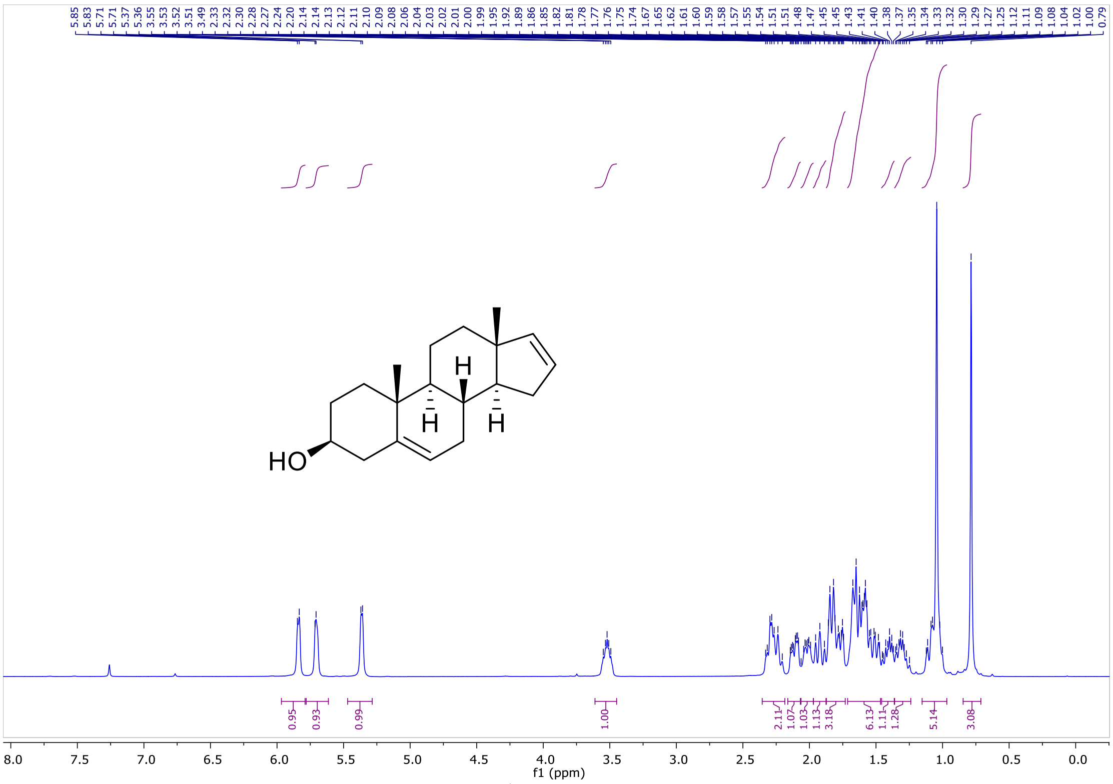

Supplementary Figure 70. ^{1}H NMR spectra of compound of **2u**

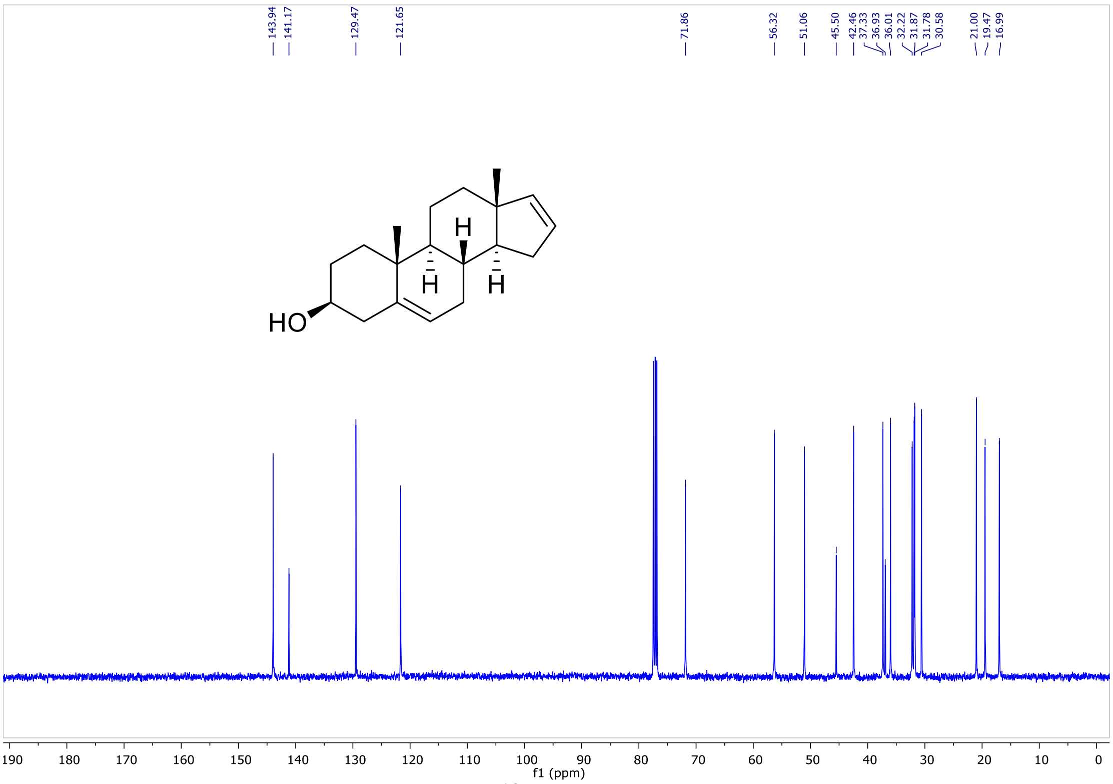

Supplementary Figure 71. ^{13}C NMR spectra of compound of **2u**

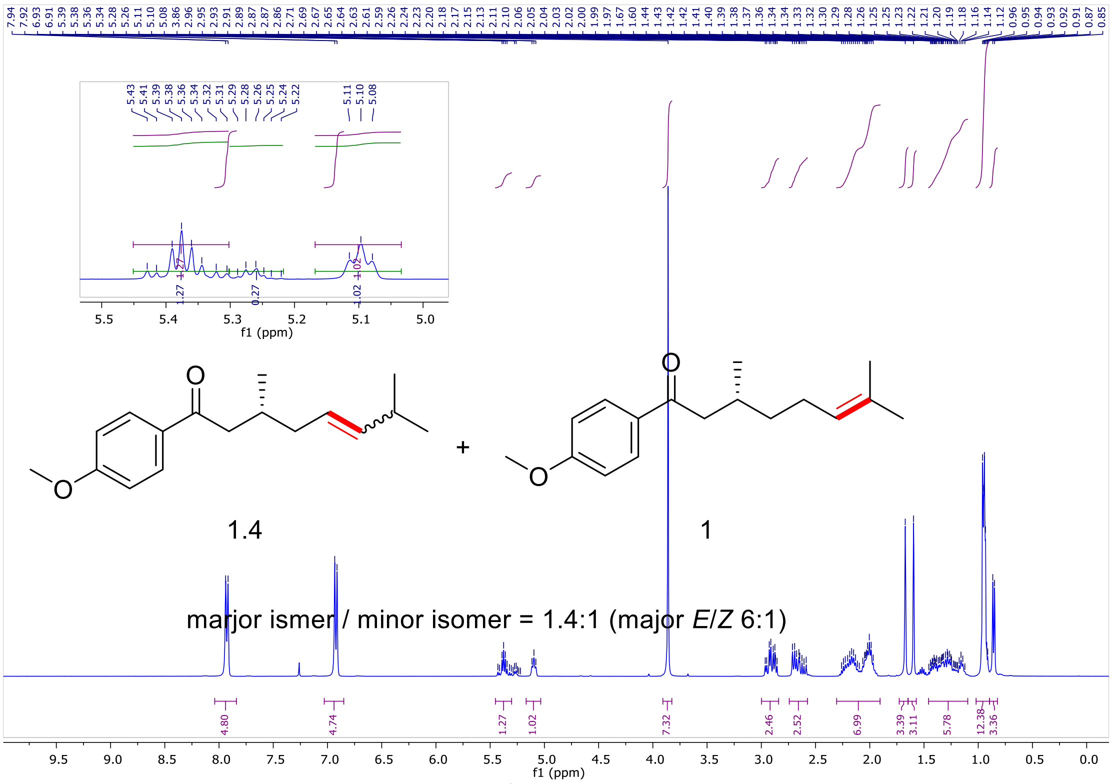

Supplementary Figure 72. ^{1}H NMR spectra of compound of **2v**

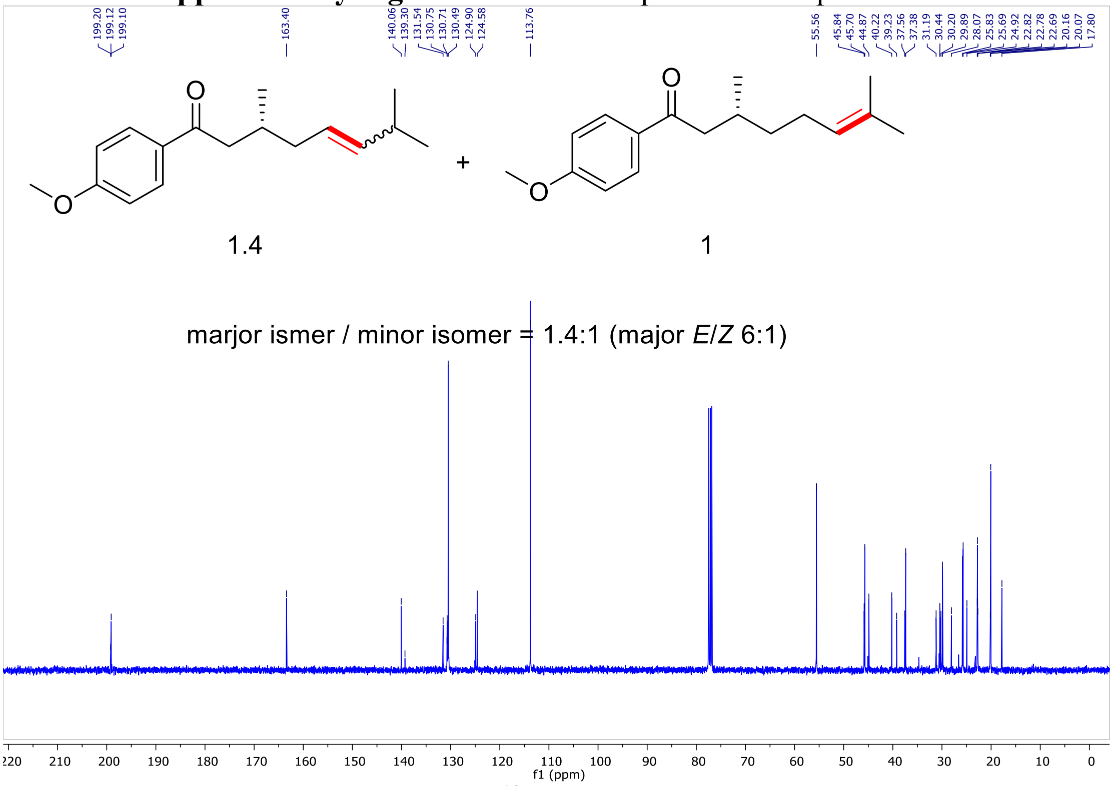

Supplementary Figure 73. ^{13}C NMR spectra of compound of **2v**

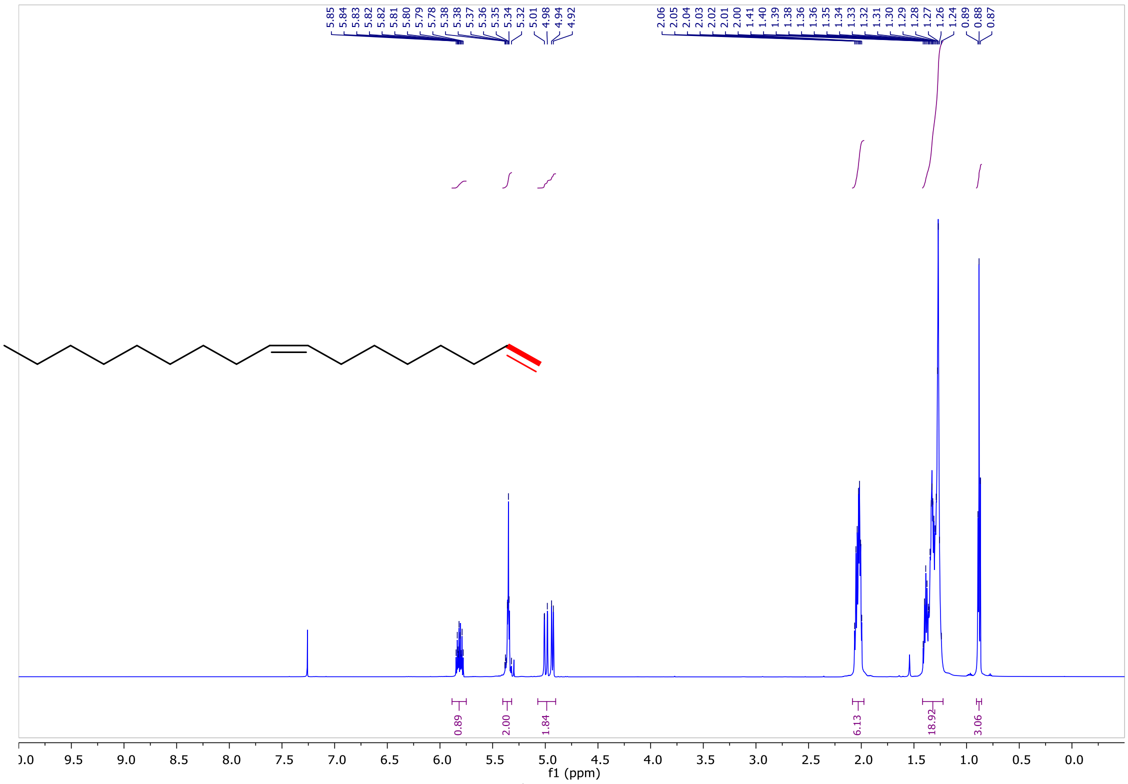

Supplementary Figure 74. ^{1}H NMR spectra of compound of **2w**

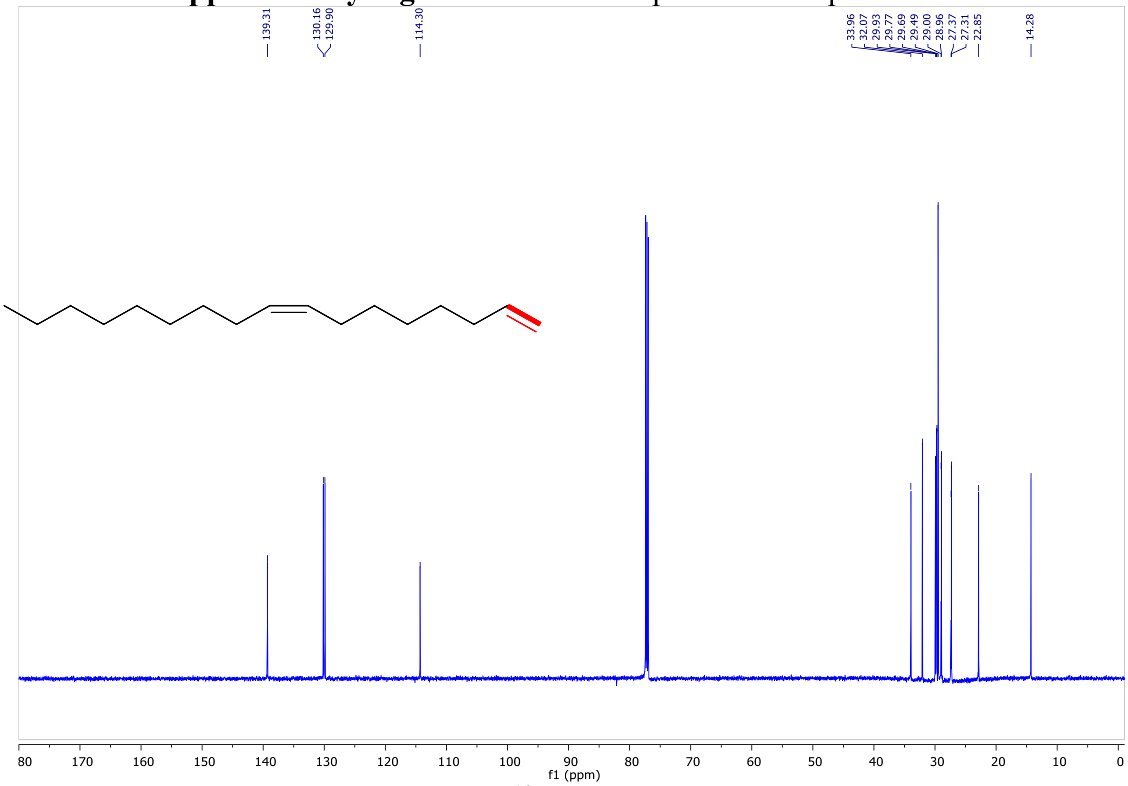

Supplementary Figure 75. ^{13}C NMR spectra of compound of **2w**

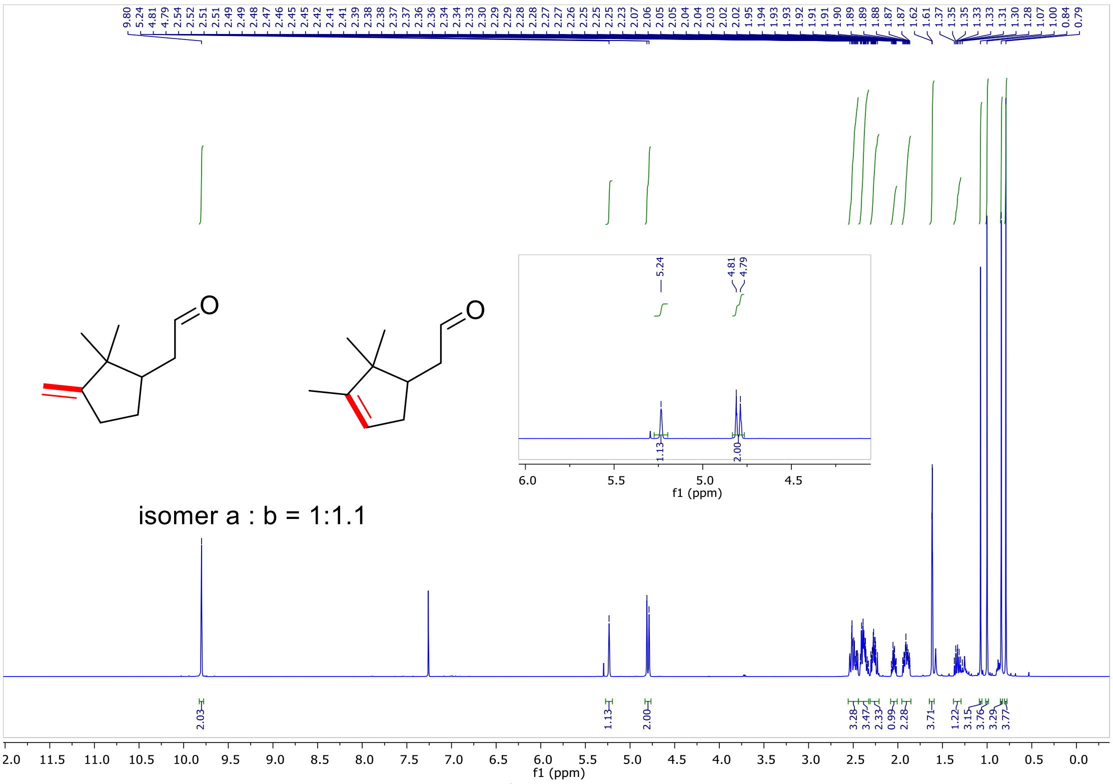

Supplementary Figure 76. ^{1}H NMR spectra of compound of **2x**

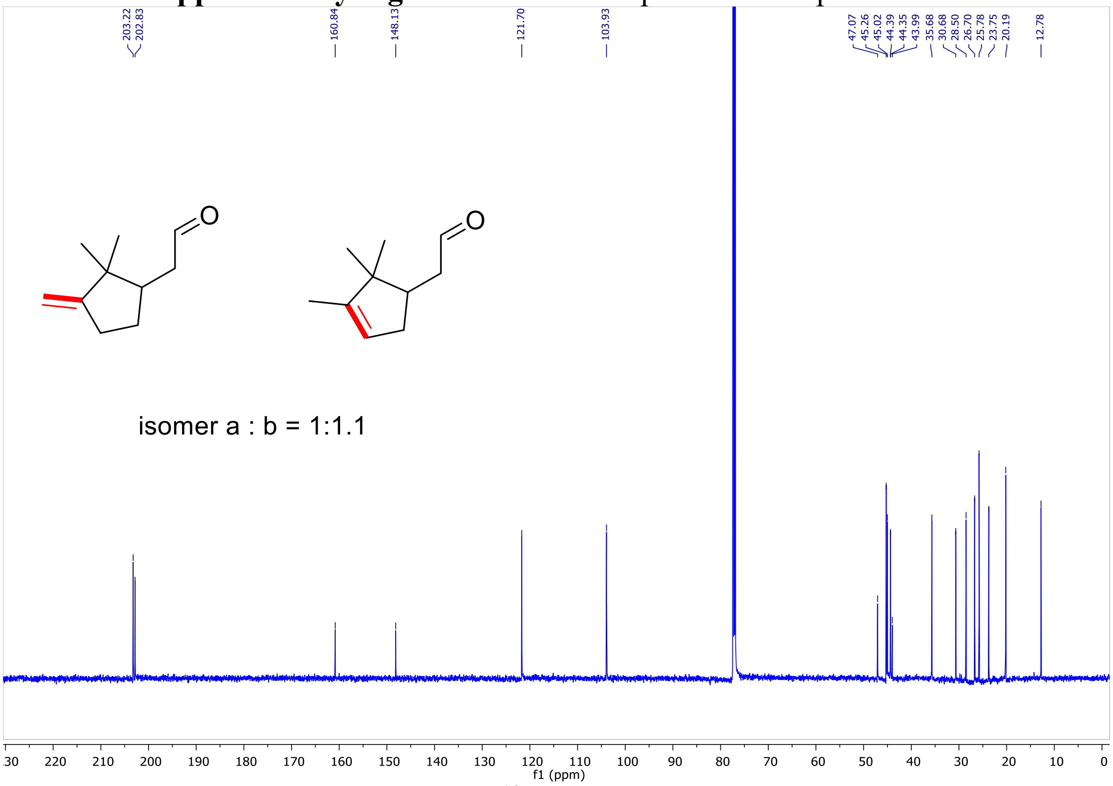

Supplementary Figure 77. ^{13}C NMR spectra of compound of **2x**

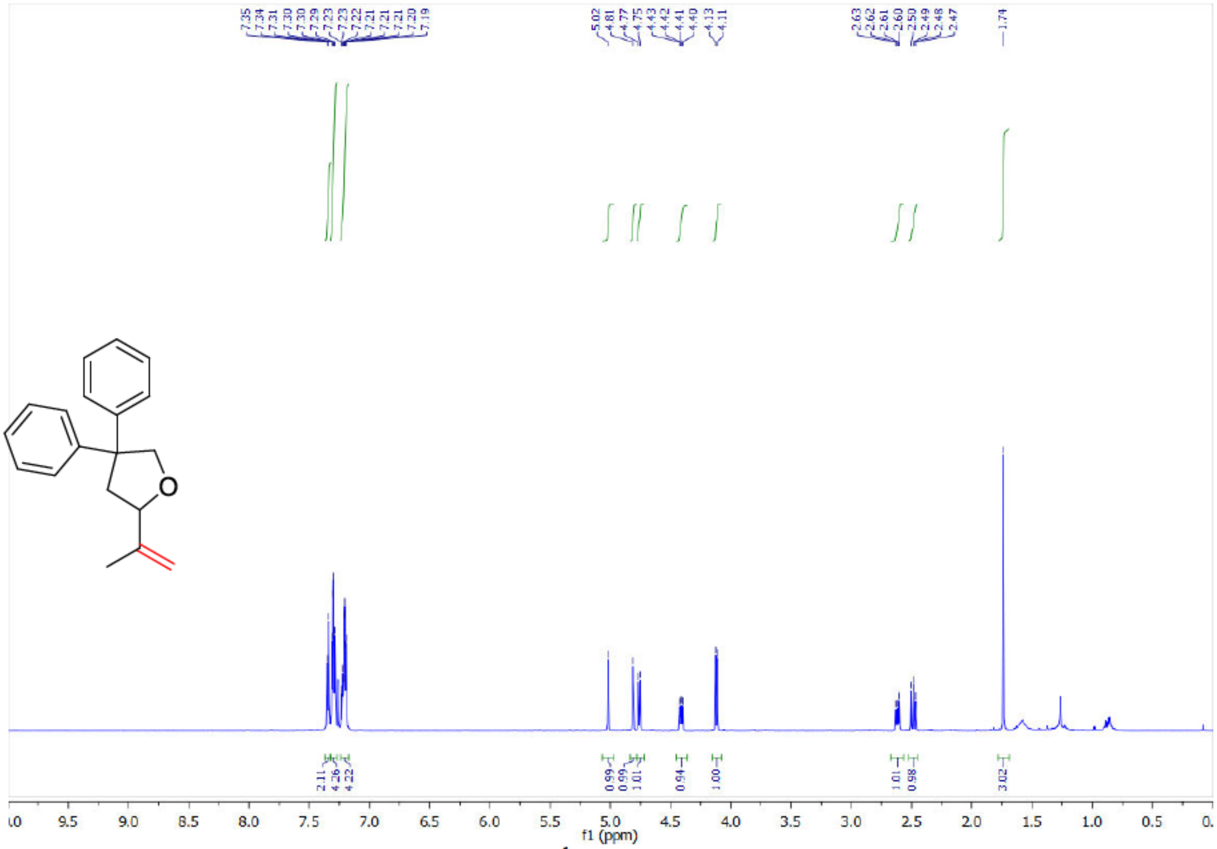

Supplementary Figure 78. ^{1}H NMR spectra of compound of **2z**

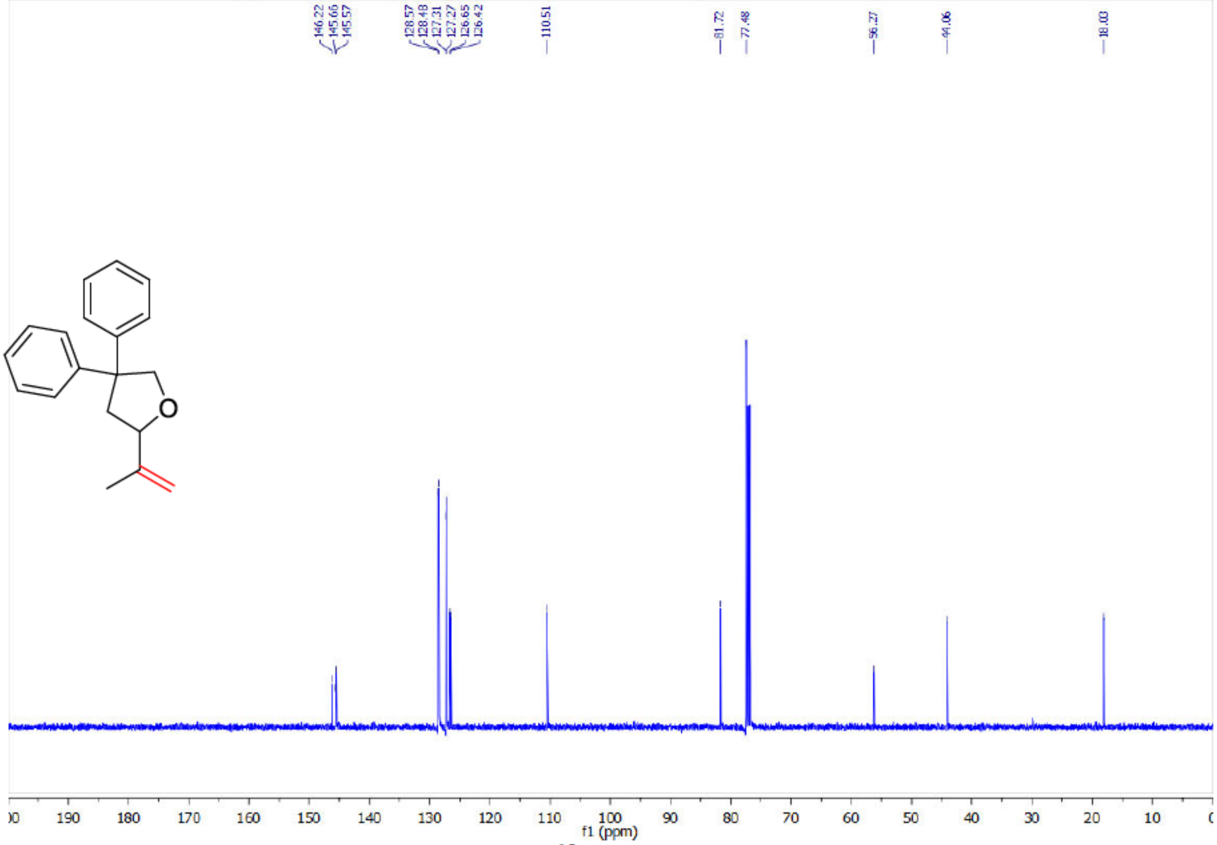

Supplementary Figure 79. ^{13}C NMR spectra of compound of **2z**

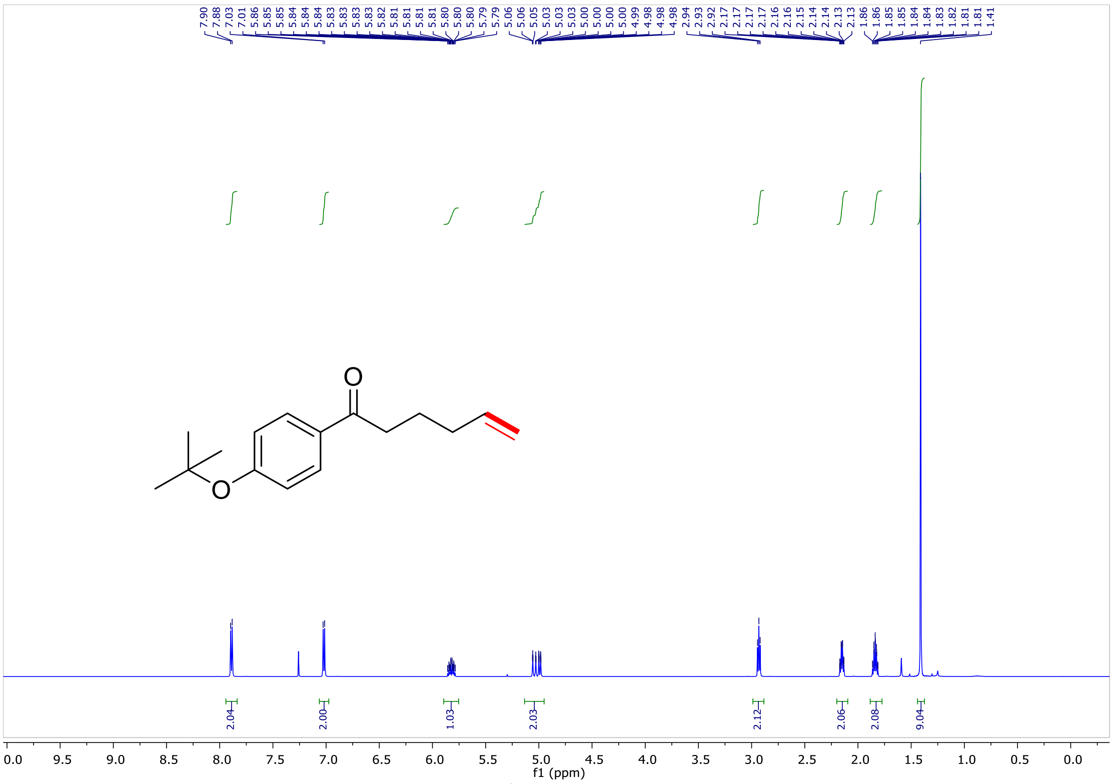

Supplementary Figure 80. ^{1}H NMR spectra of compound of **4a**

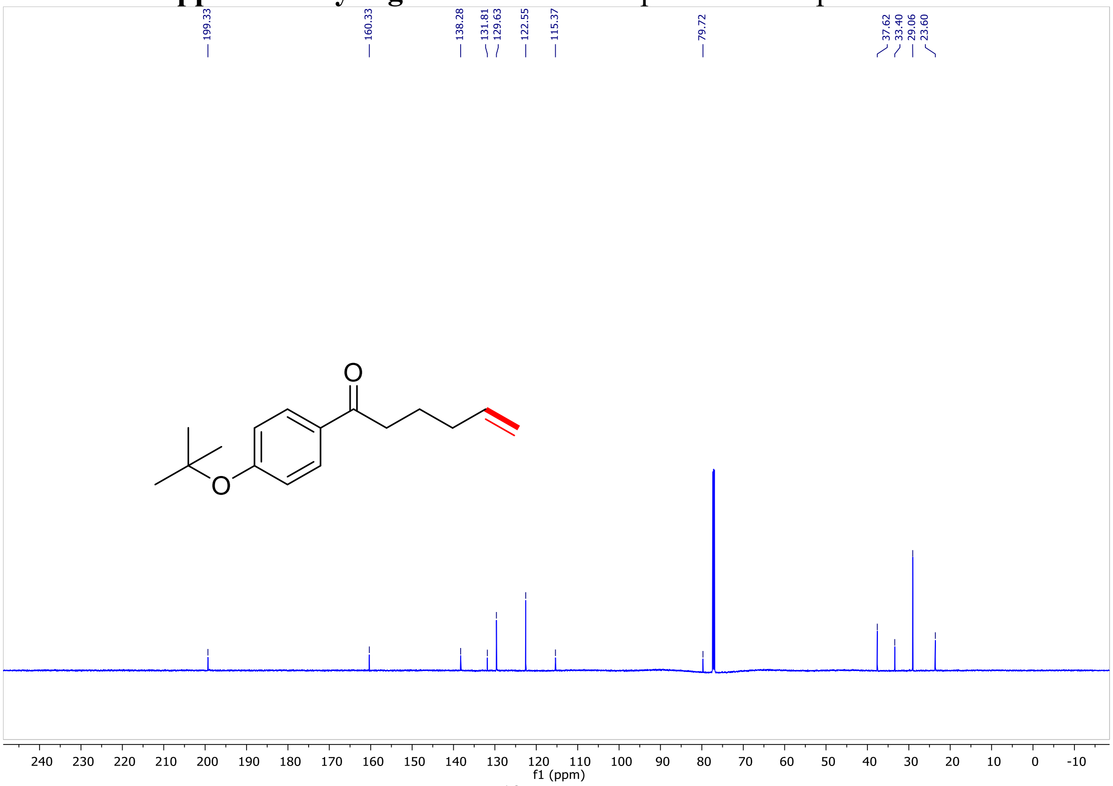

Supplementary Figure 81. ^{13}C NMR spectra of compound of **4a**

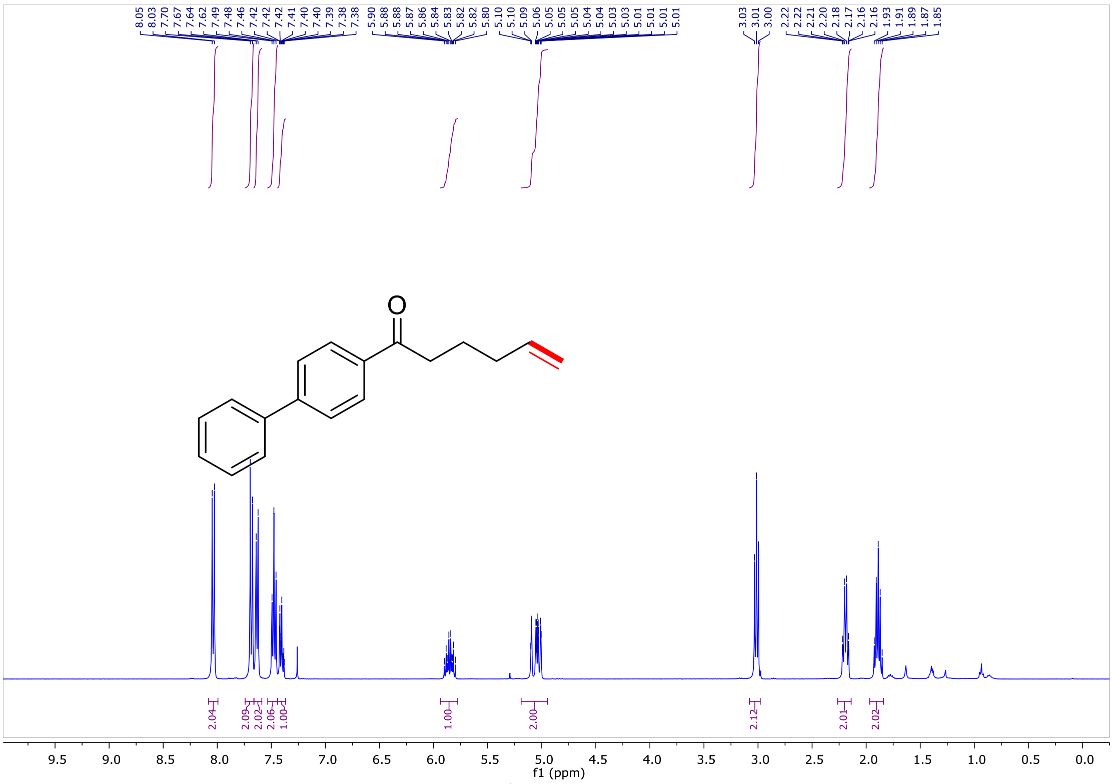

Supplementary Figure 82. ^{1}H NMR spectra of compound of **4b**

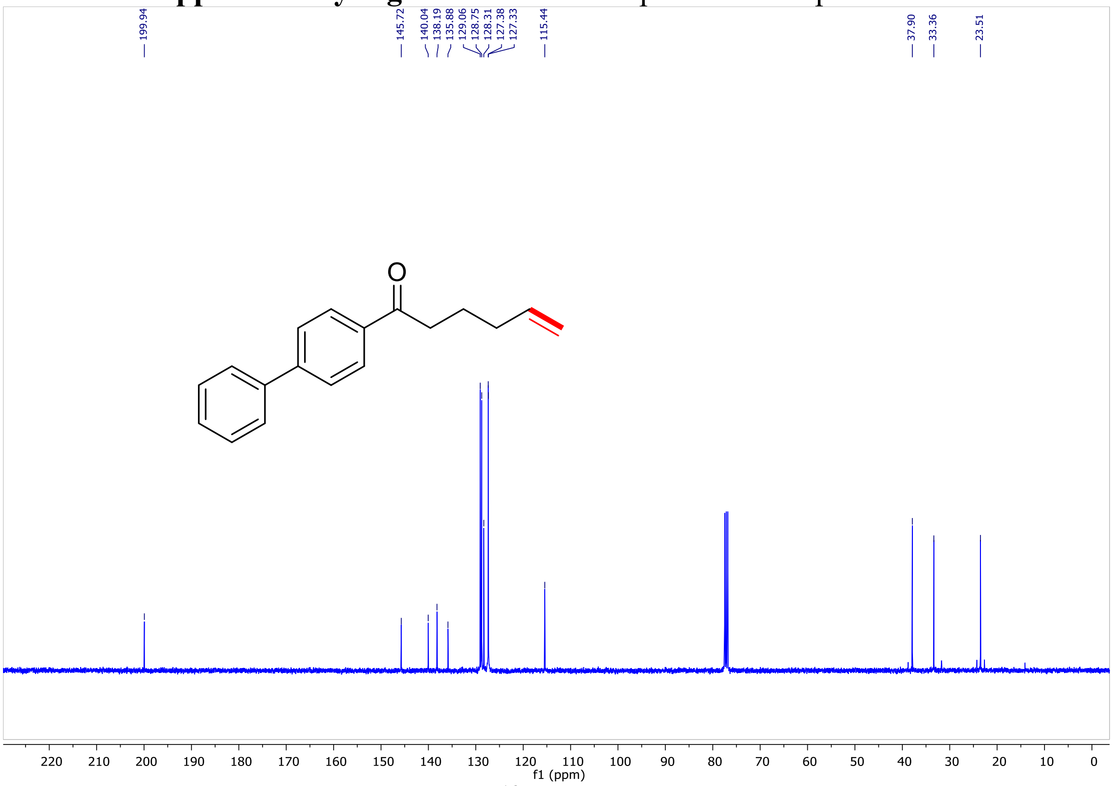

Supplementary Figure 83. ^{13}C NMR spectra of compound of **4b**

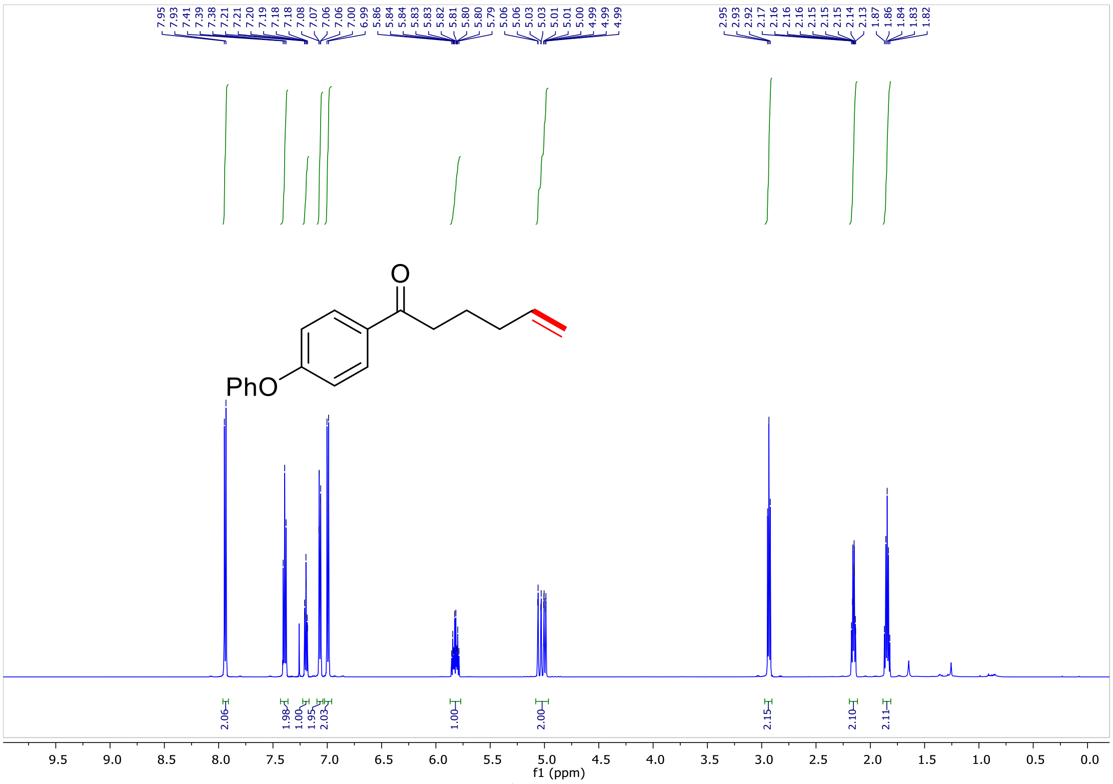

Supplementary Figure 84. ^{1}H NMR spectra of compound of **4c**

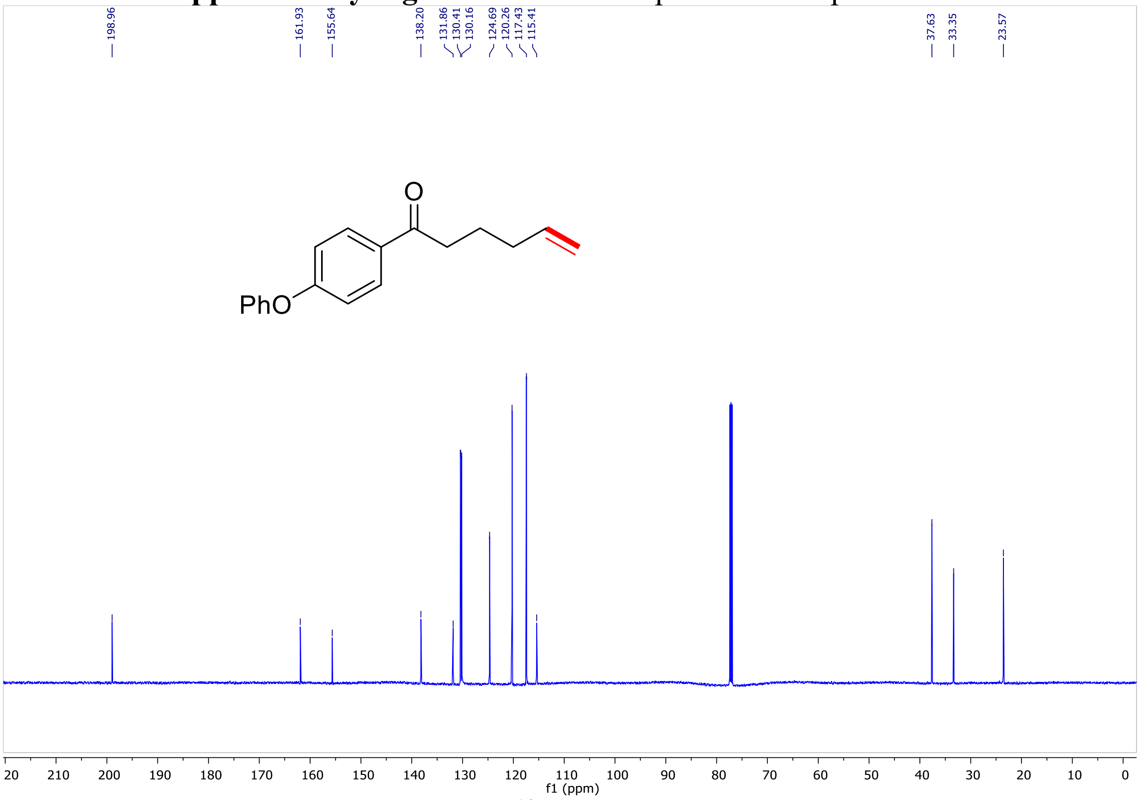

Supplementary Figure 85. ^{13}C NMR spectra of compound of **4c**

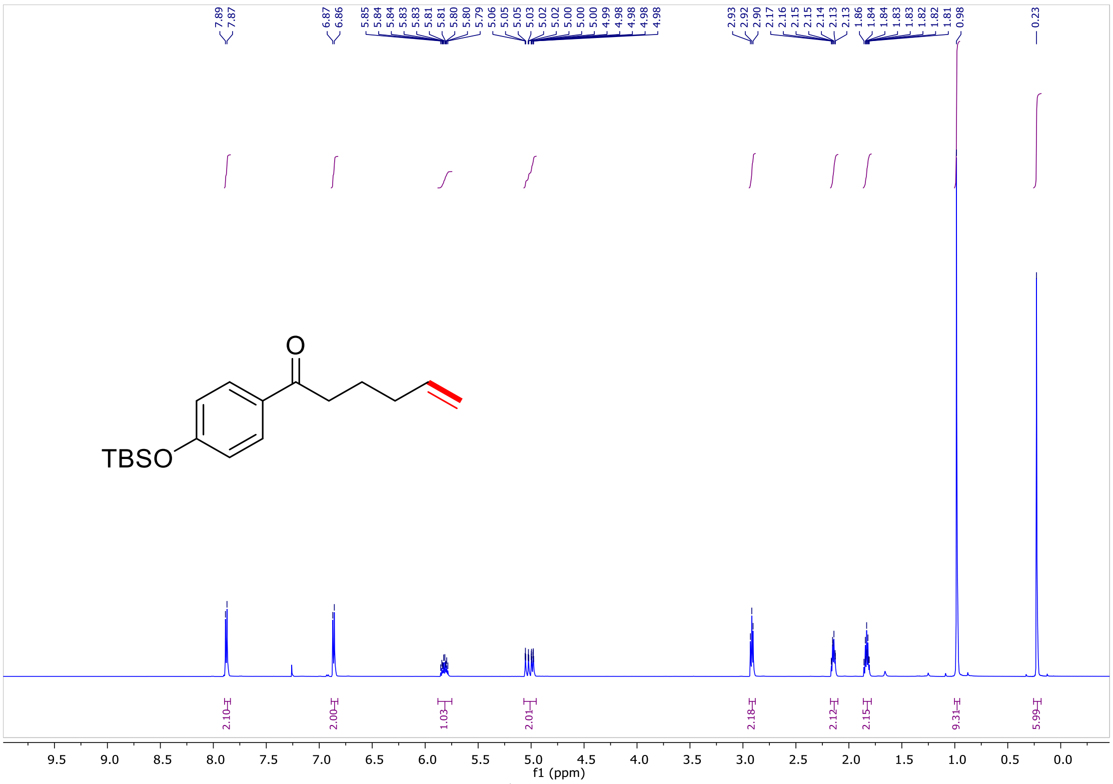

Supplementary Figure 86. ^{1}H NMR spectra of compound of **4d**

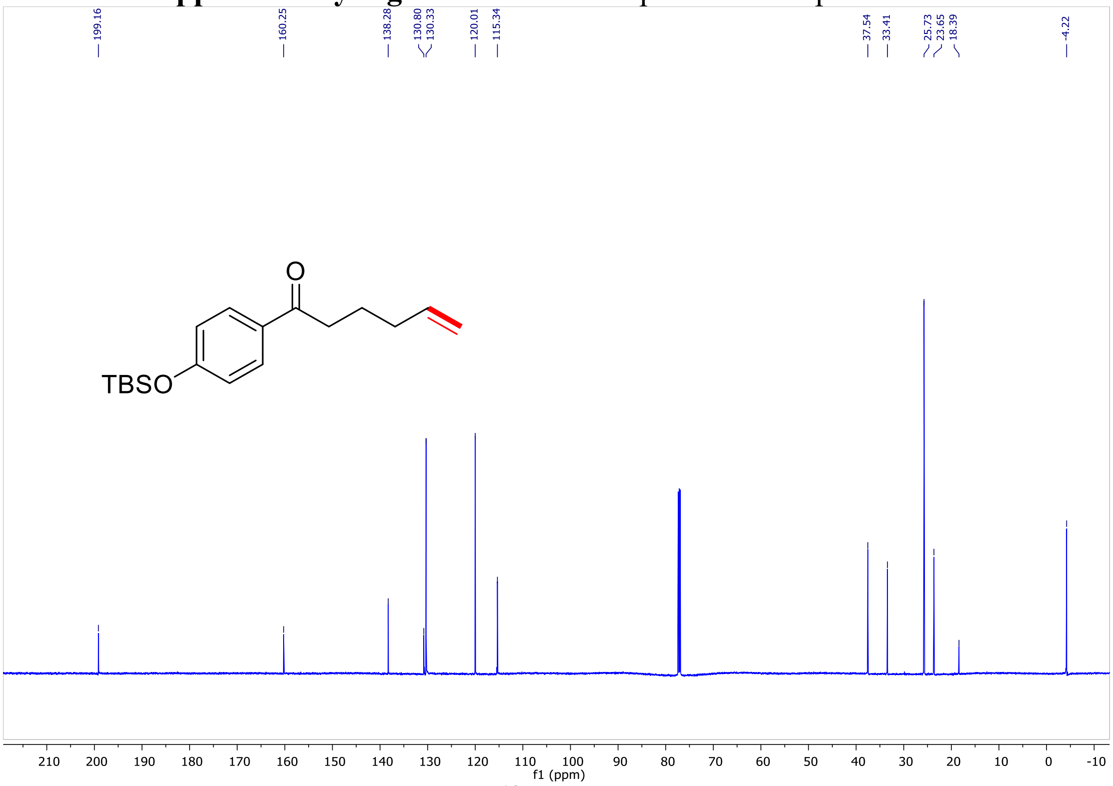

Supplementary Figure 87. ^{13}C NMR spectra of compound of **4d**

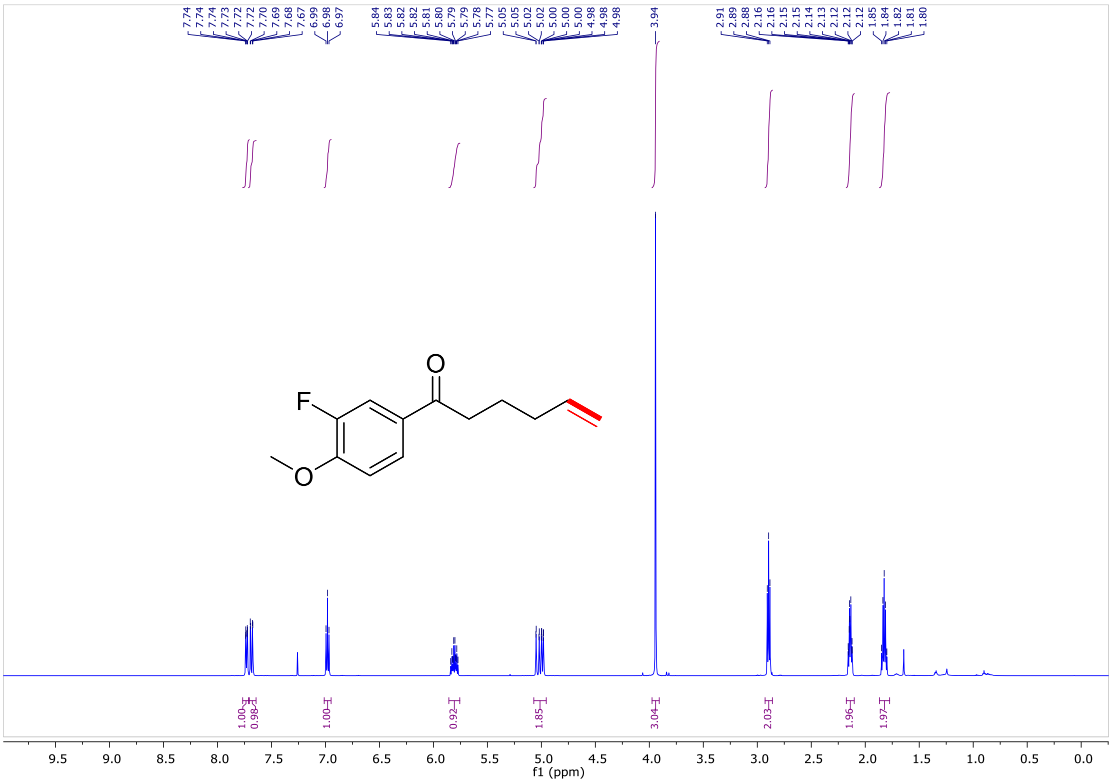

Supplementary Figure 88. ^{1}H NMR spectra of compound of **4e**

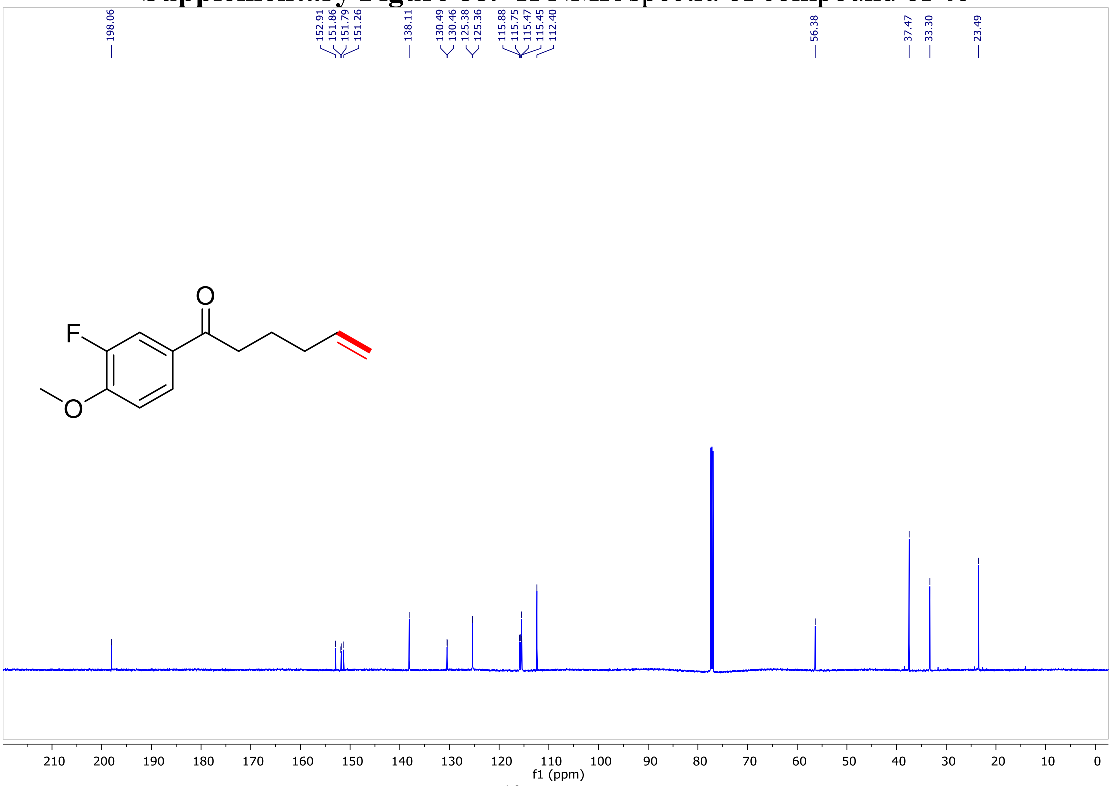

Supplementary Figure 89. ^{13}C NMR spectra of compound of **4e**

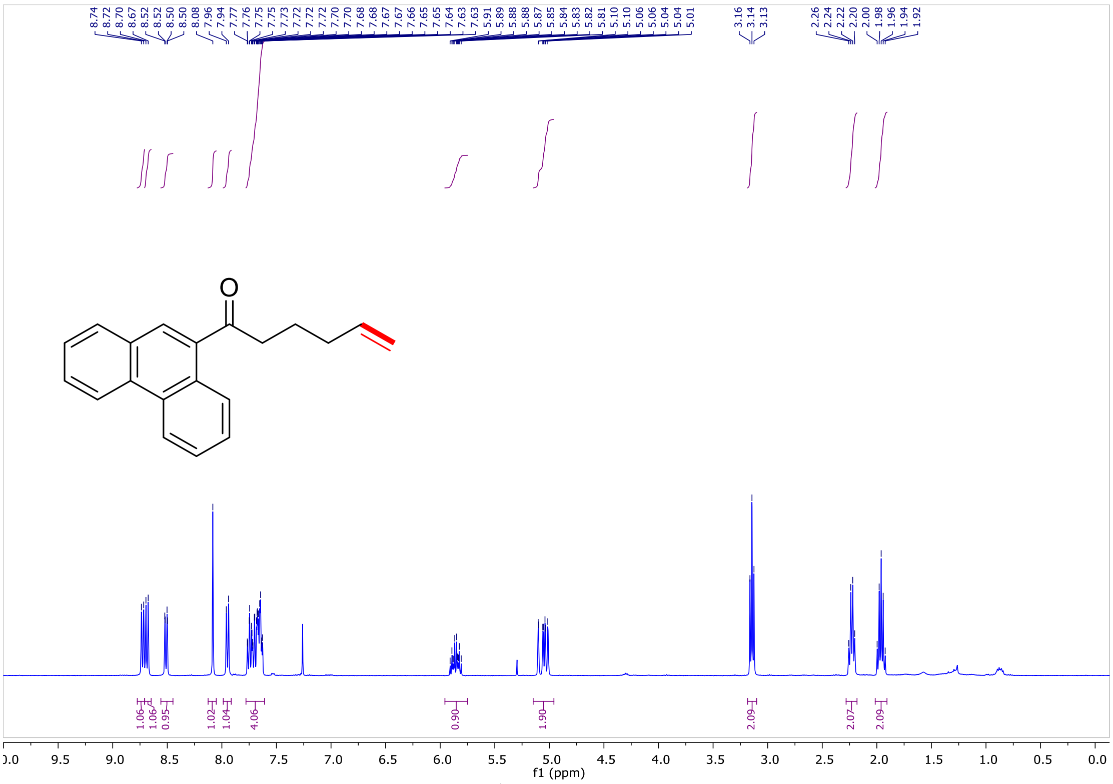

Supplementary Figure 90. ^{1}H NMR spectra of compound of **4f-1**

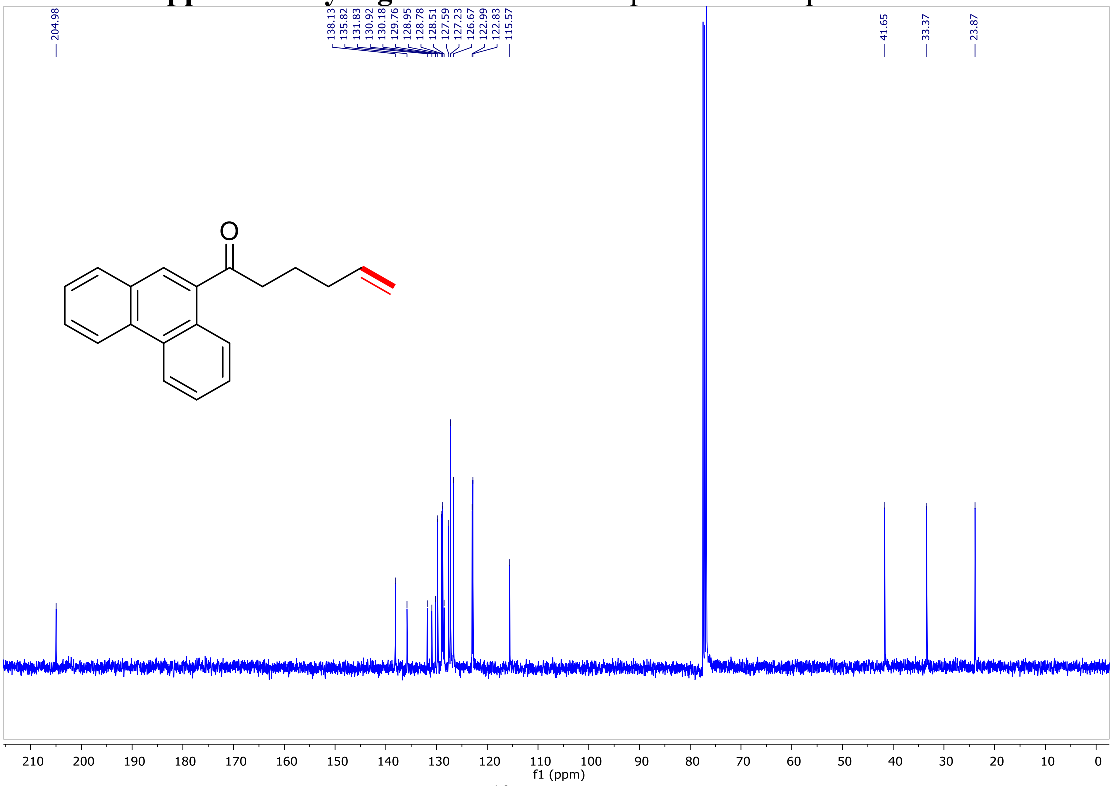

Supplementary Figure 91. ^{13}C NMR spectra of compound of **4f-1**

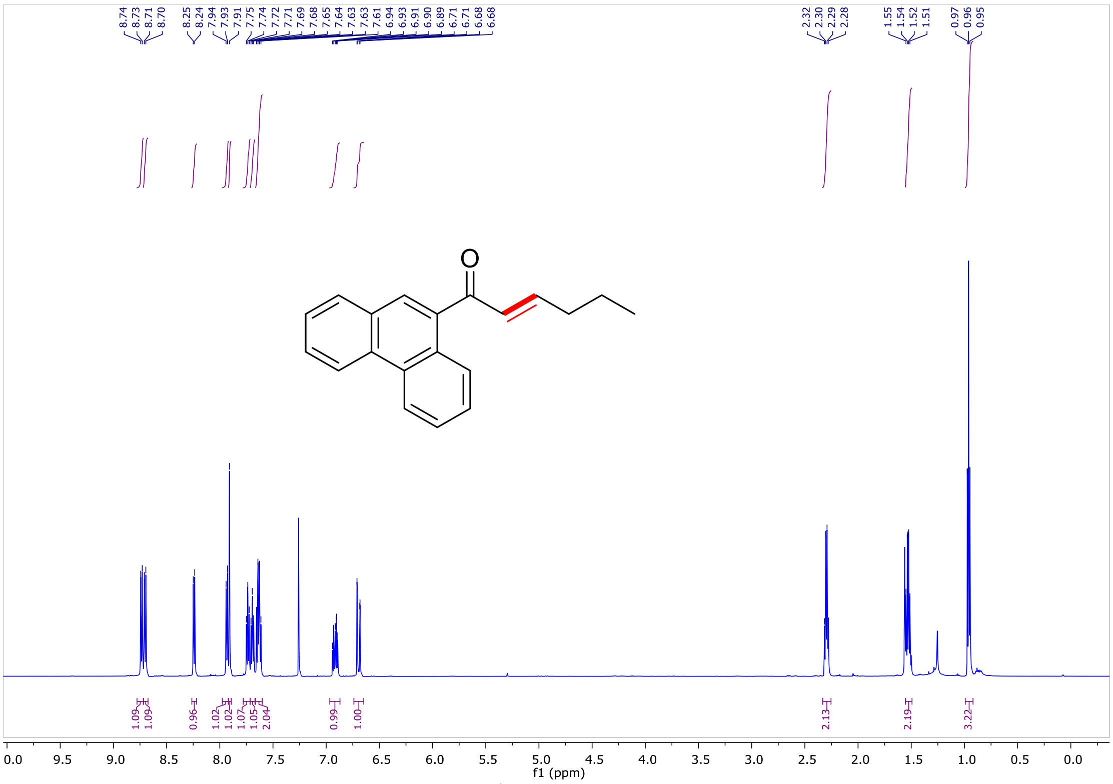

Supplementary Figure 92. ^{1}H NMR spectra of compound of **4f-2**

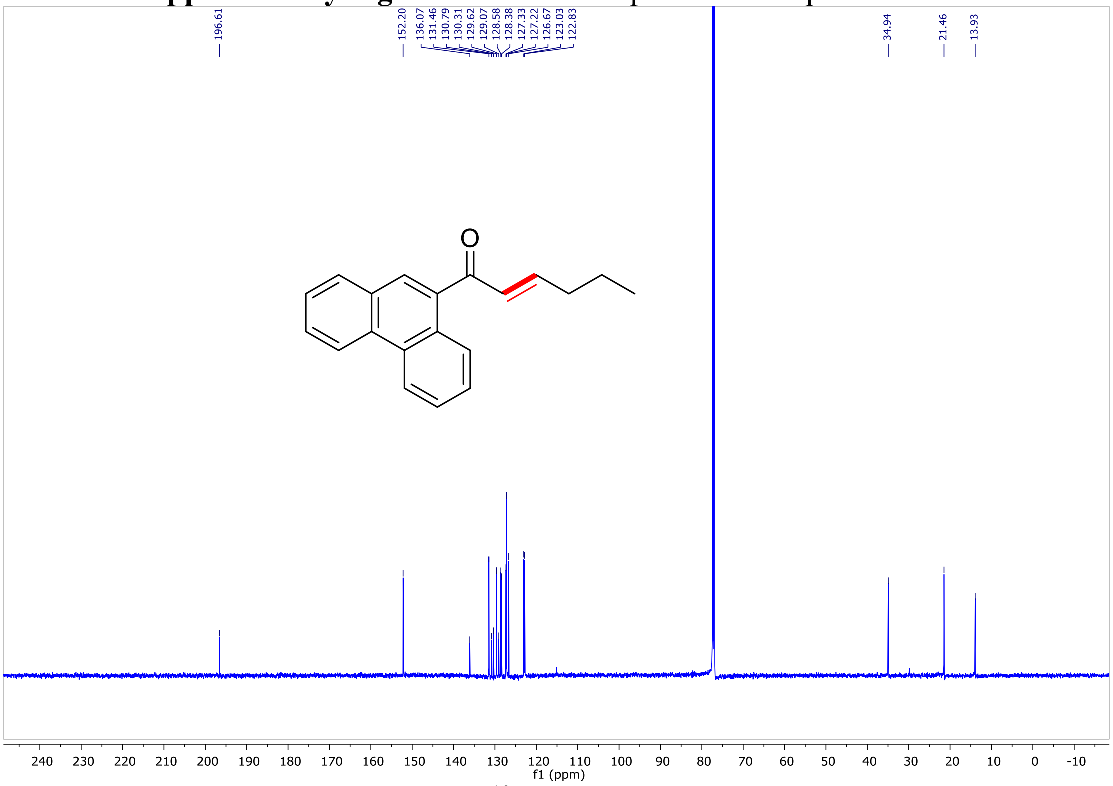

Supplementary Figure 93. ^{13}C NMR spectra of compound of **4f-2**

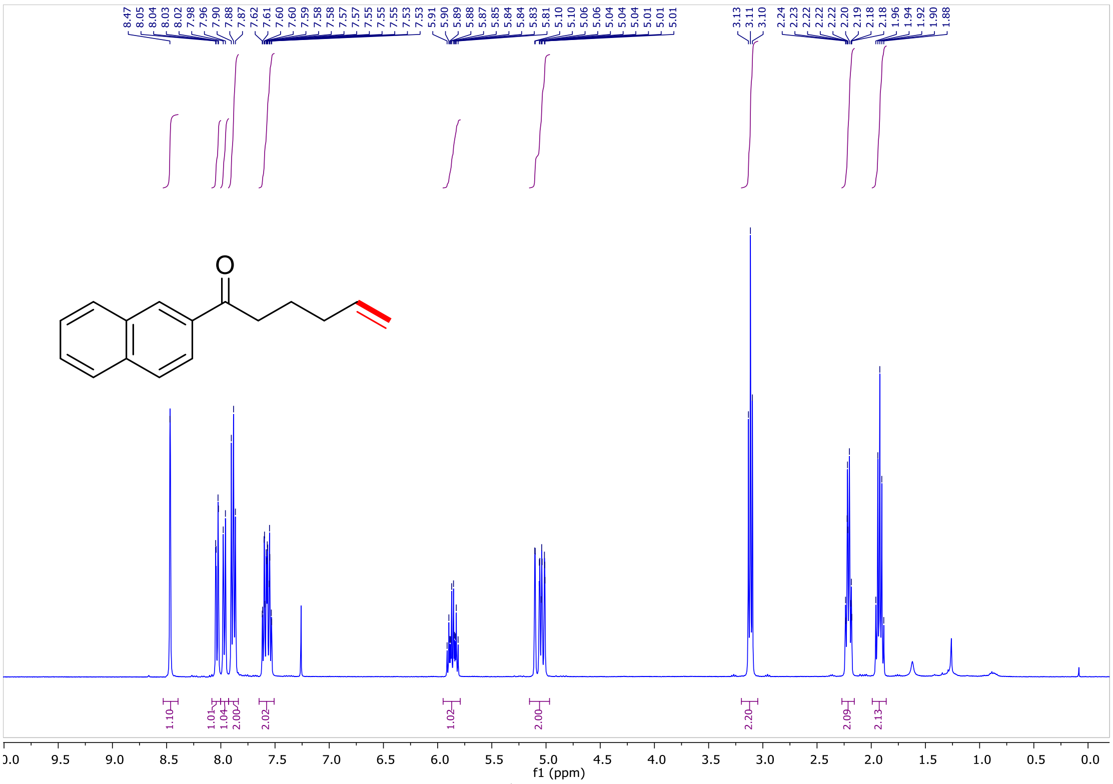

Supplementary Figure 94. ^{1}H NMR spectra of compound of **4g**

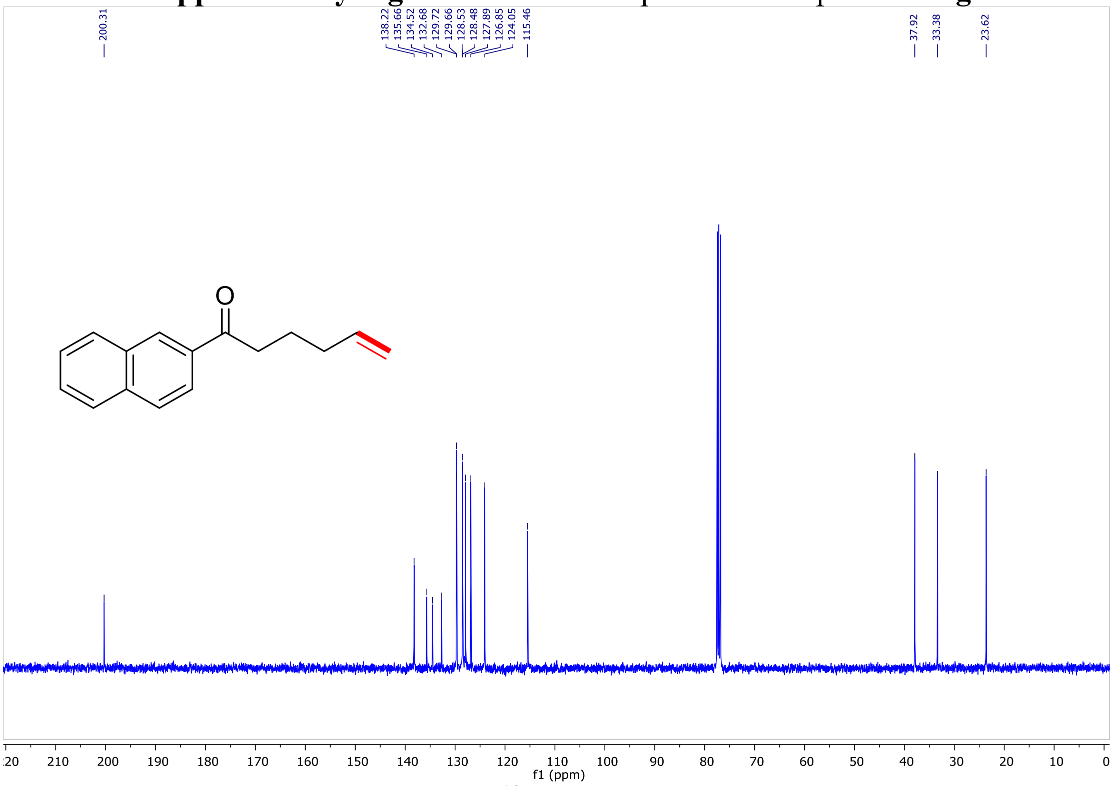

Supplementary Figure 95. ^{13}C NMR spectra of compound of **4g**

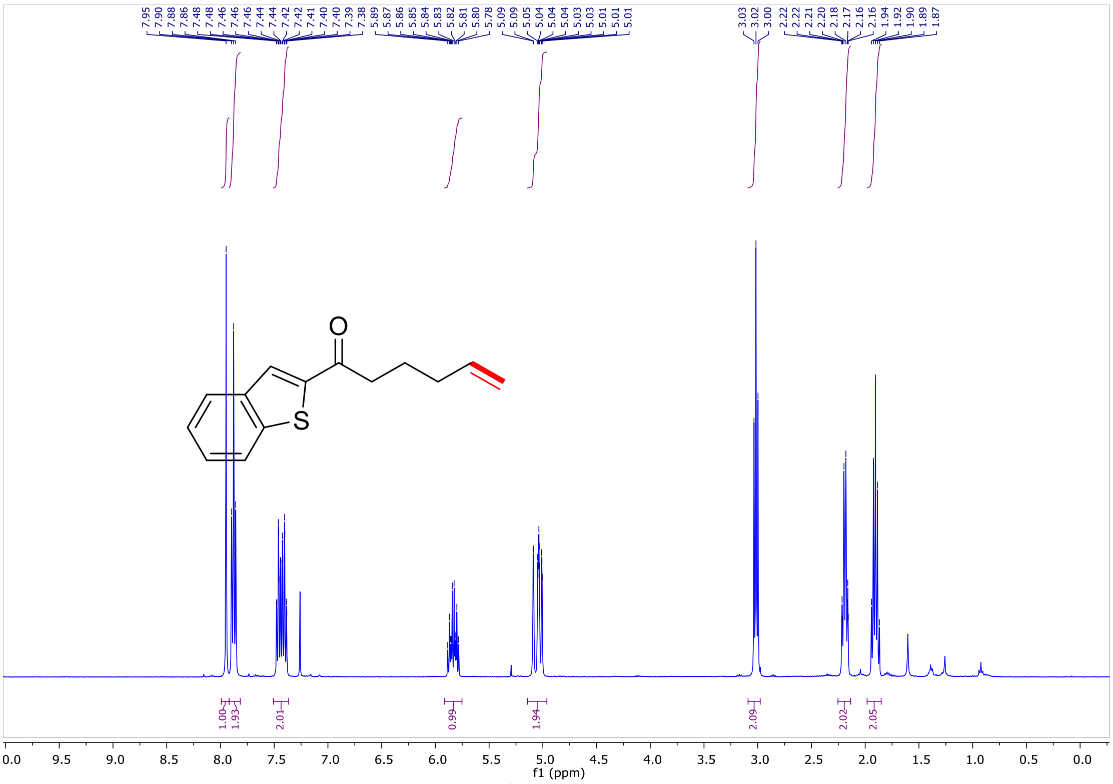

Supplementary Figure 96. ^{1}H NMR spectra of compound of **4h**

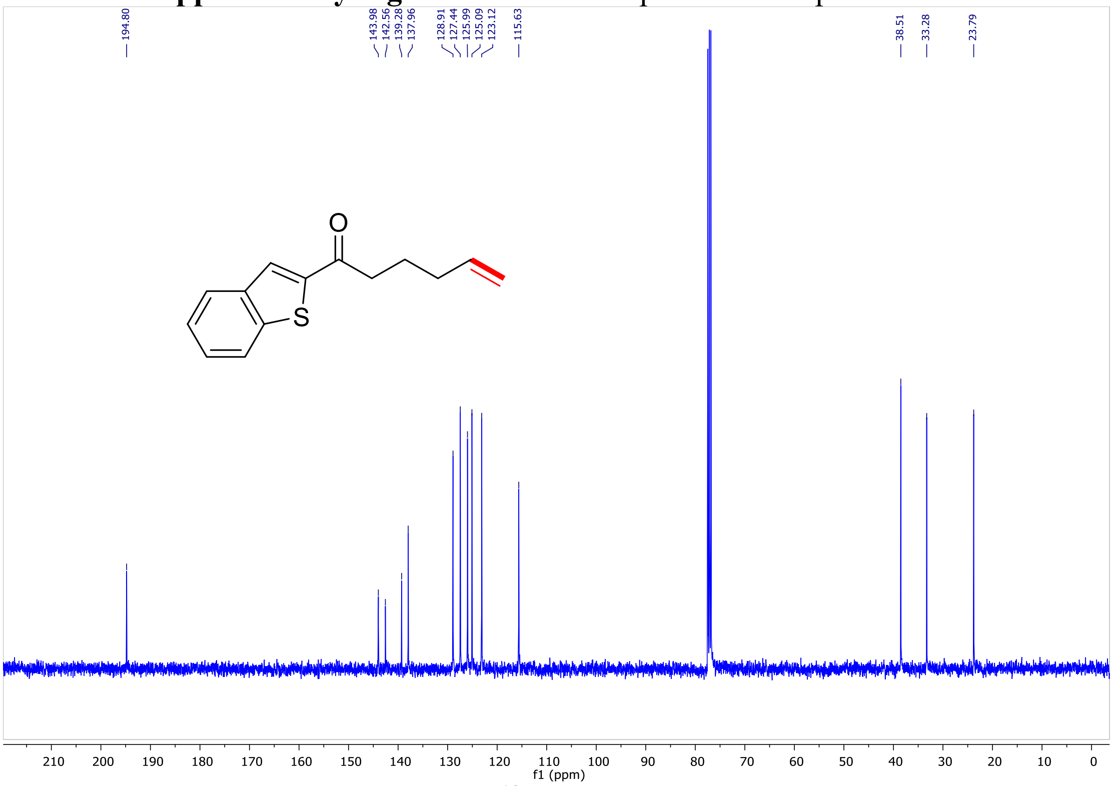

Supplementary Figure 97. ^{13}C NMR spectra of compound of **4h**

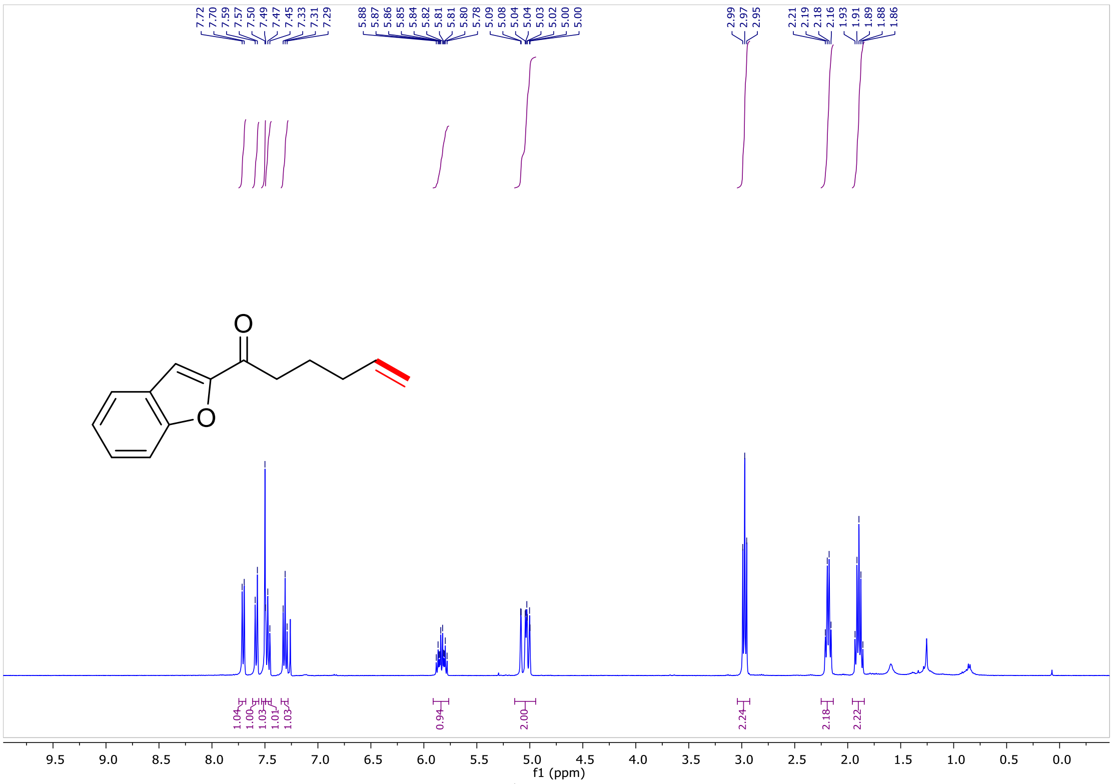

Supplementary Figure 98. ^{1}H NMR spectra of compound of **4i**

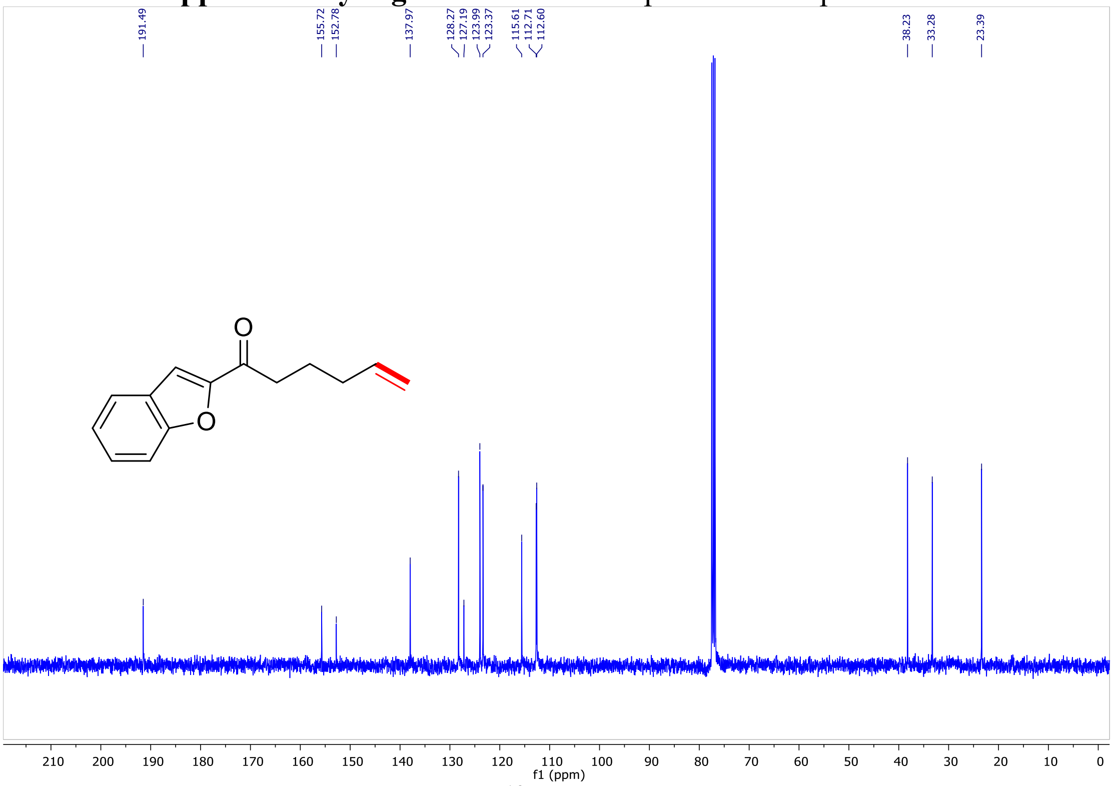

Supplementary Figure 99. ^{13}C NMR spectra of compound of **4i**

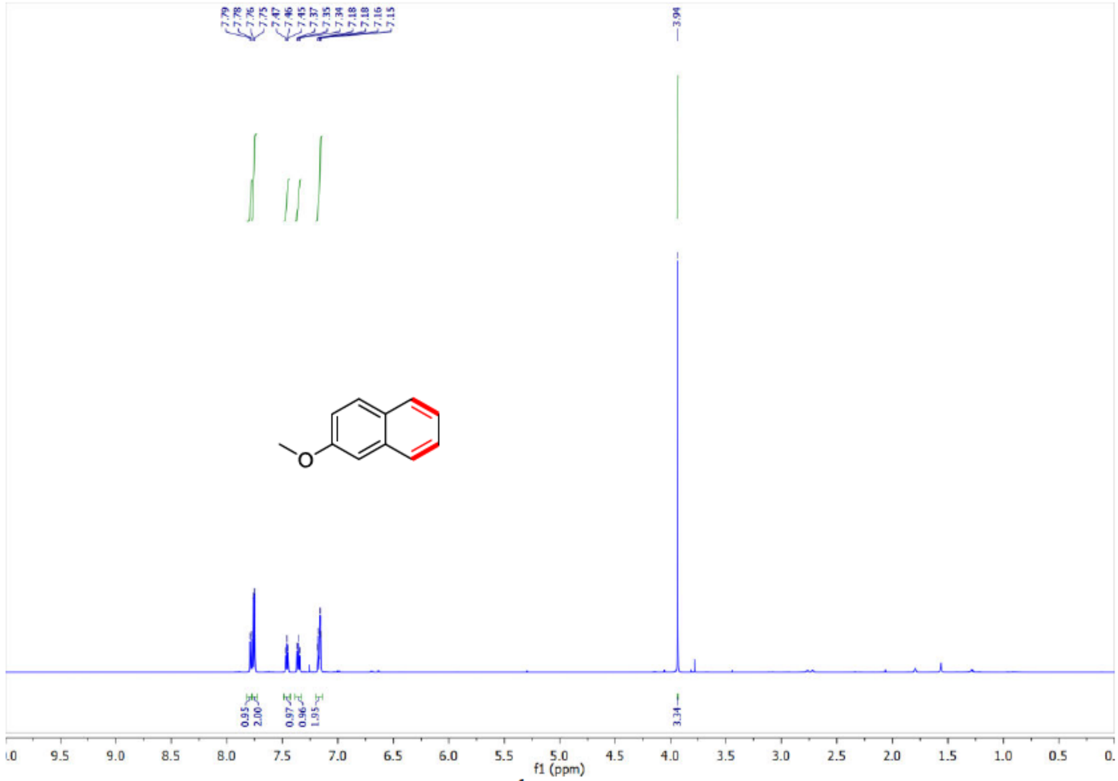

Supplementary Figure 100. ^{1}H NMR spectra of compound of **6a**

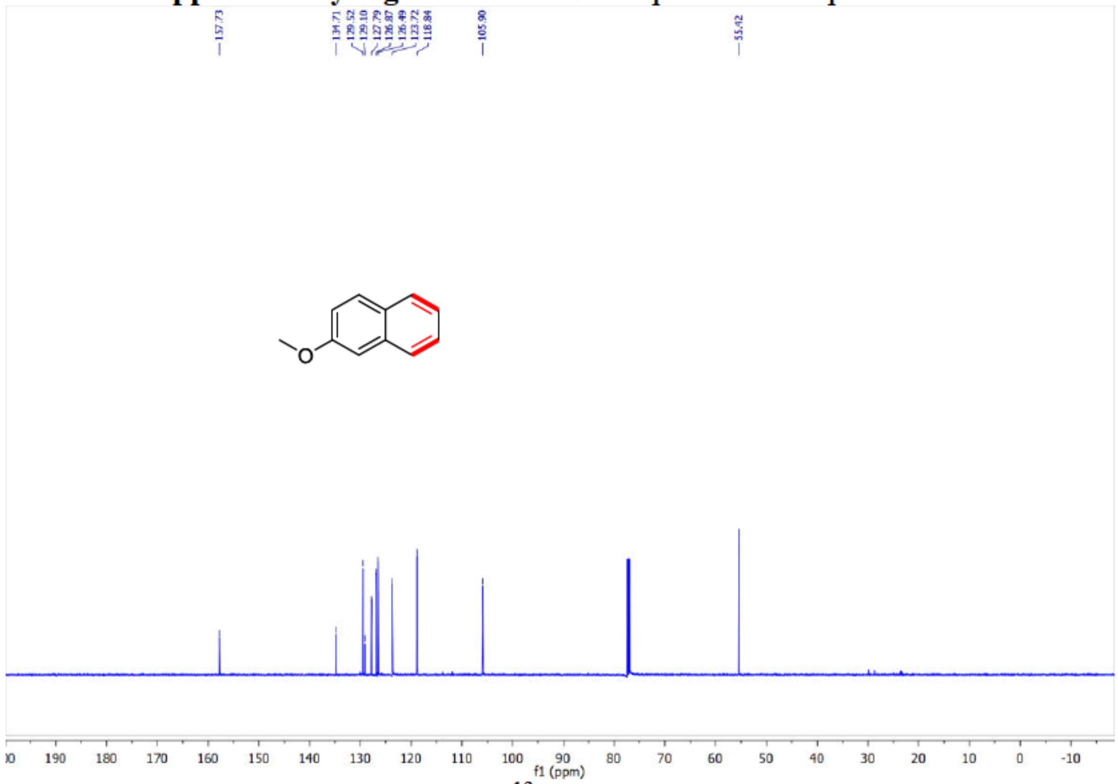

Supplementary Figure 101. ^{13}C NMR spectra of compound of **6a**

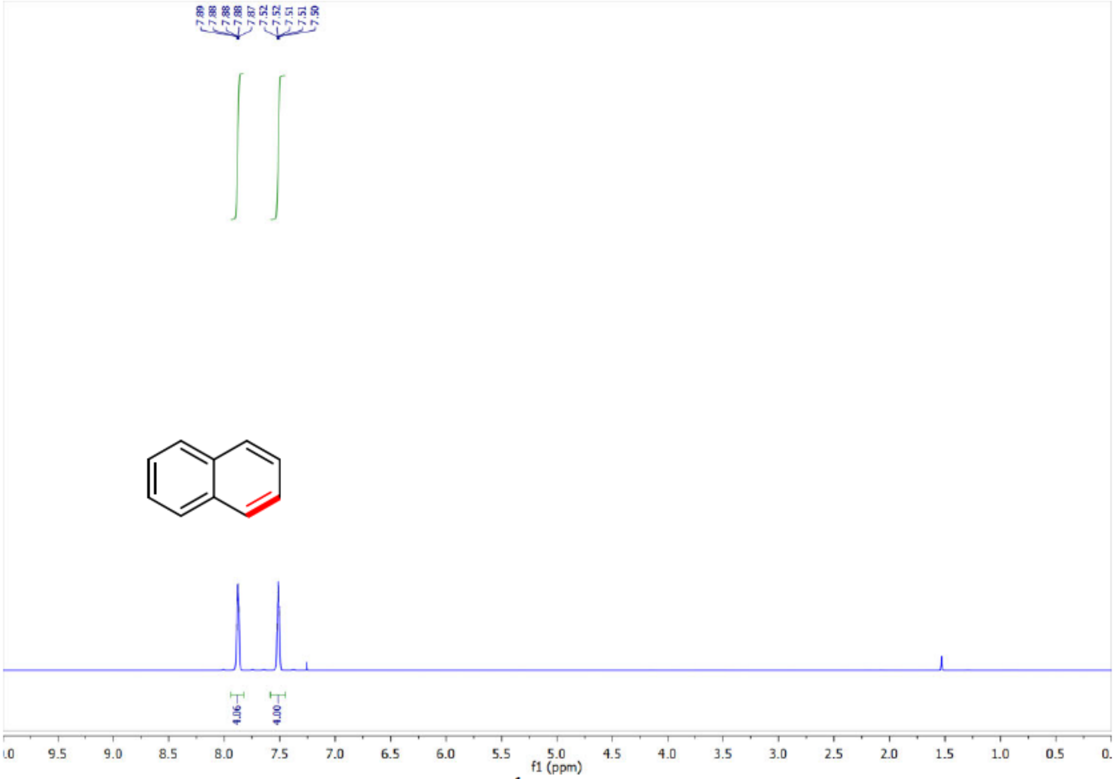

Supplementary Figure 102. ^{1}H NMR spectra of compound of **6b**

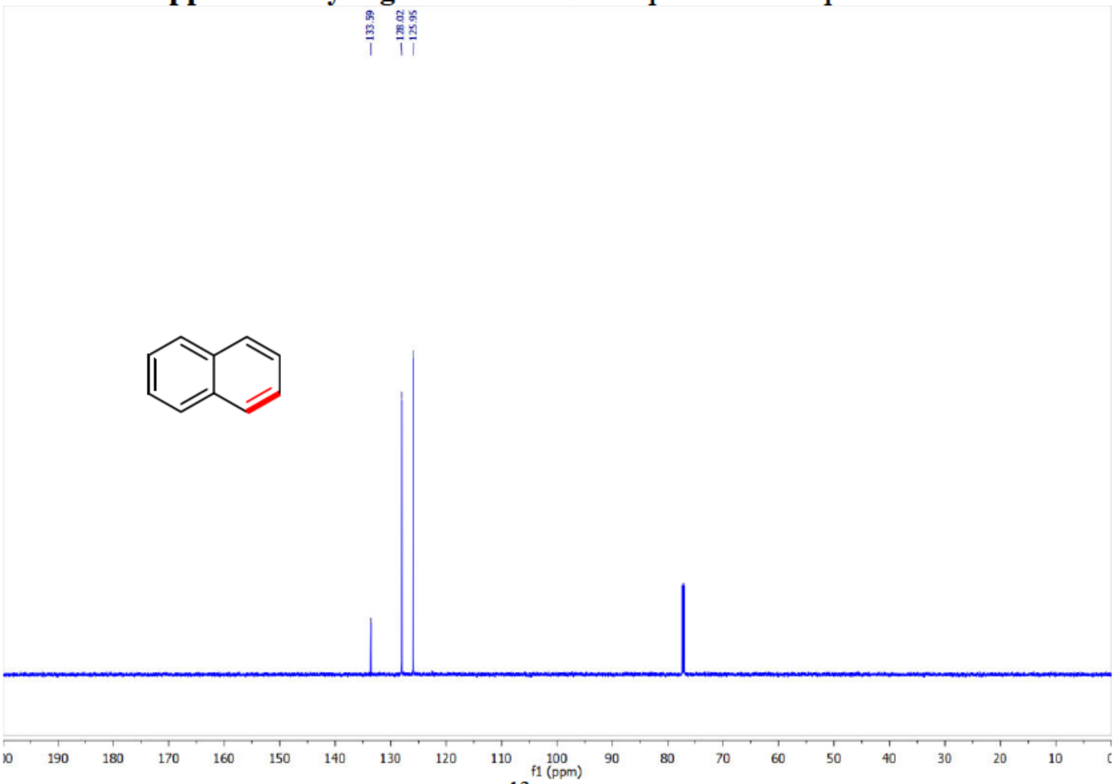

Supplementary Figure 103. ^{13}C NMR spectra of compound of **6b**

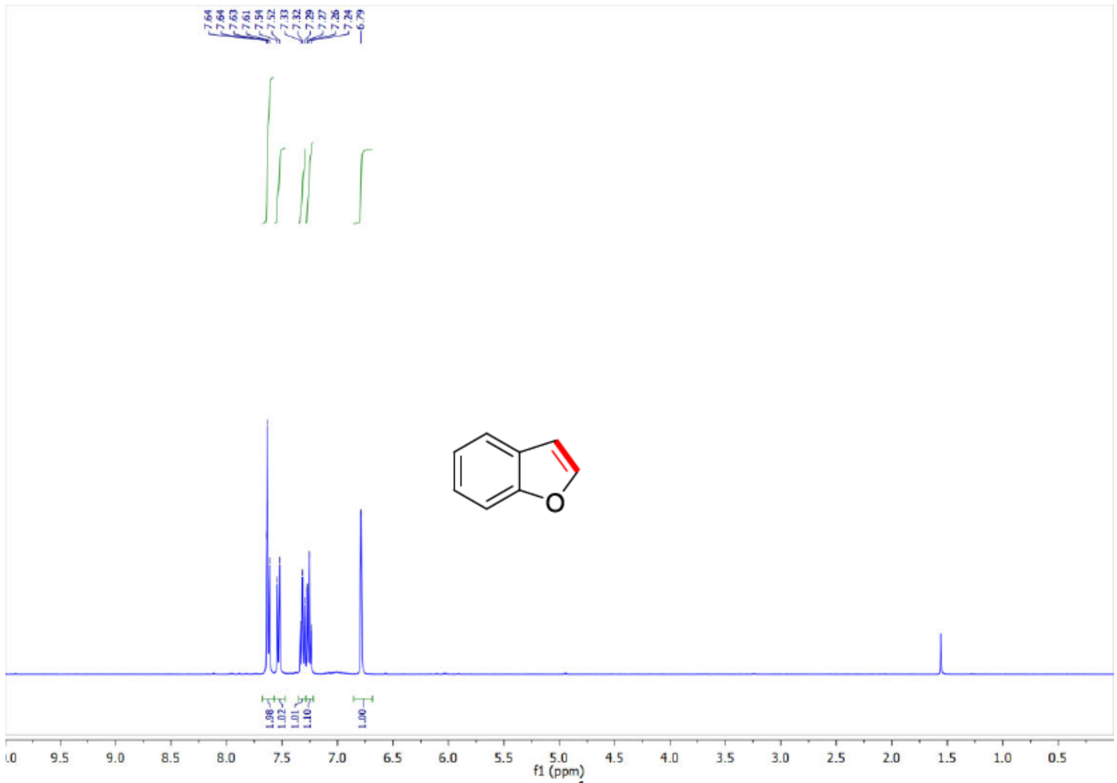

Supplementary Figure 104. ^{1}H NMR spectra of compound of **6c**

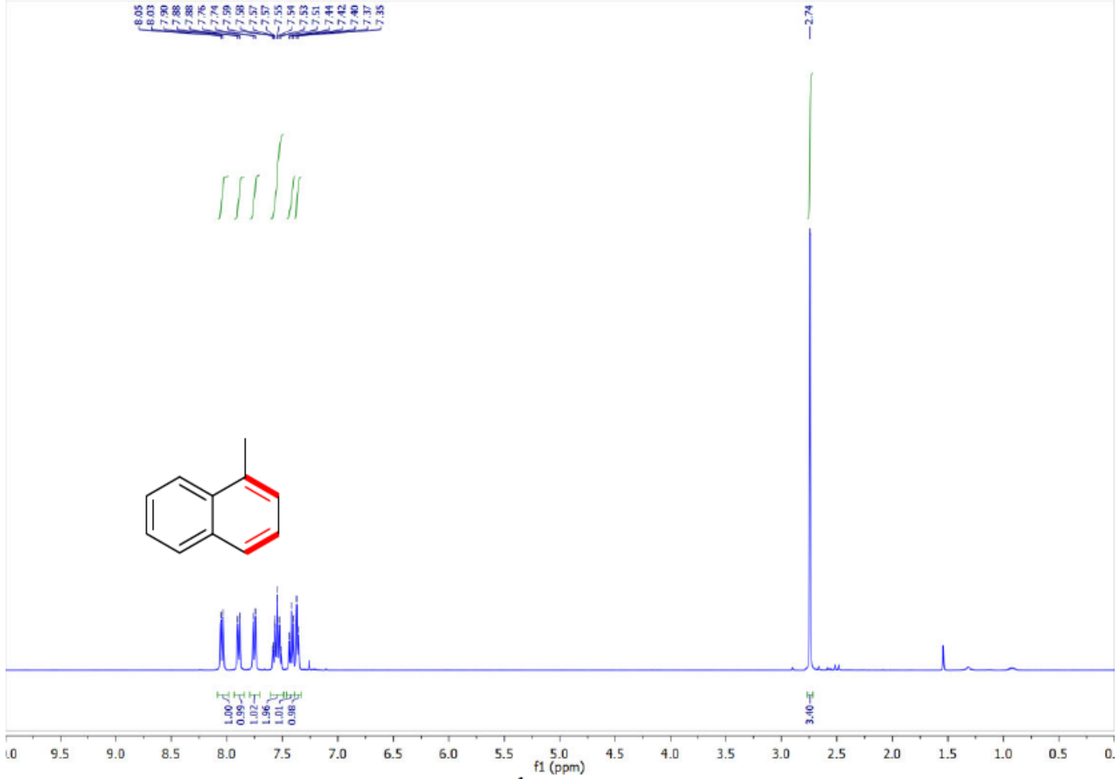

Supplementary Figure 105. ^{1}H NMR spectra of compound of **6d**

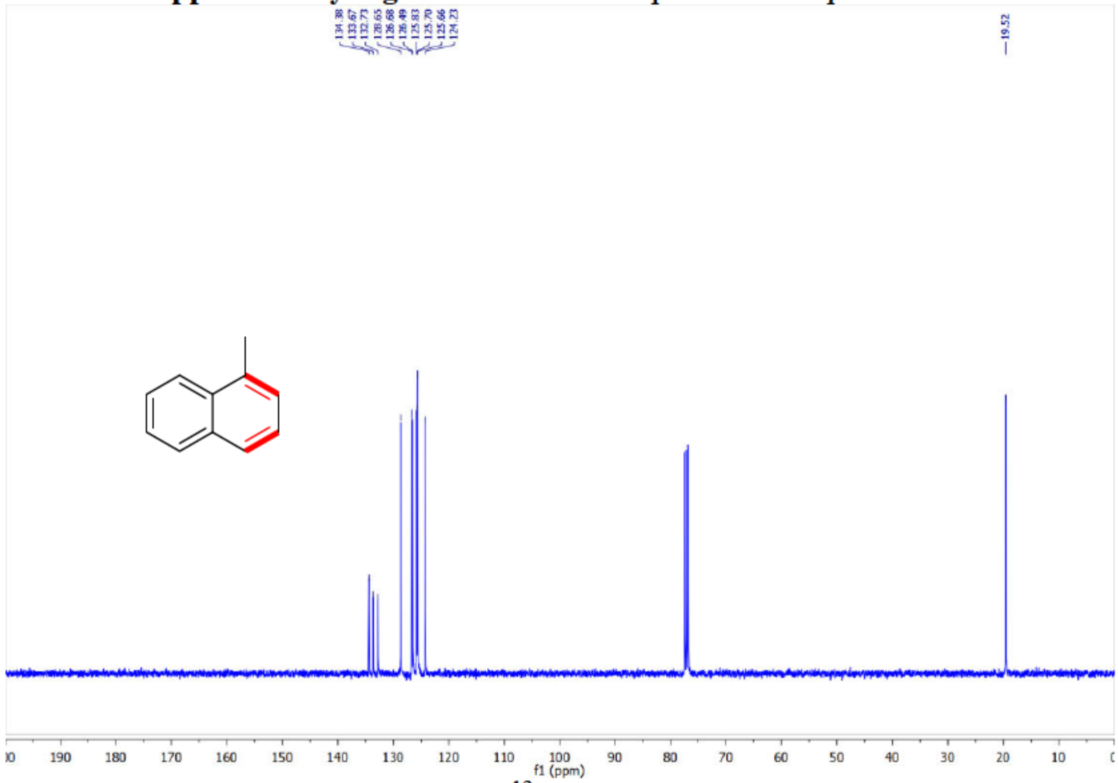

Supplementary Figure 106. ^{13}C NMR spectra of compound of **6d**

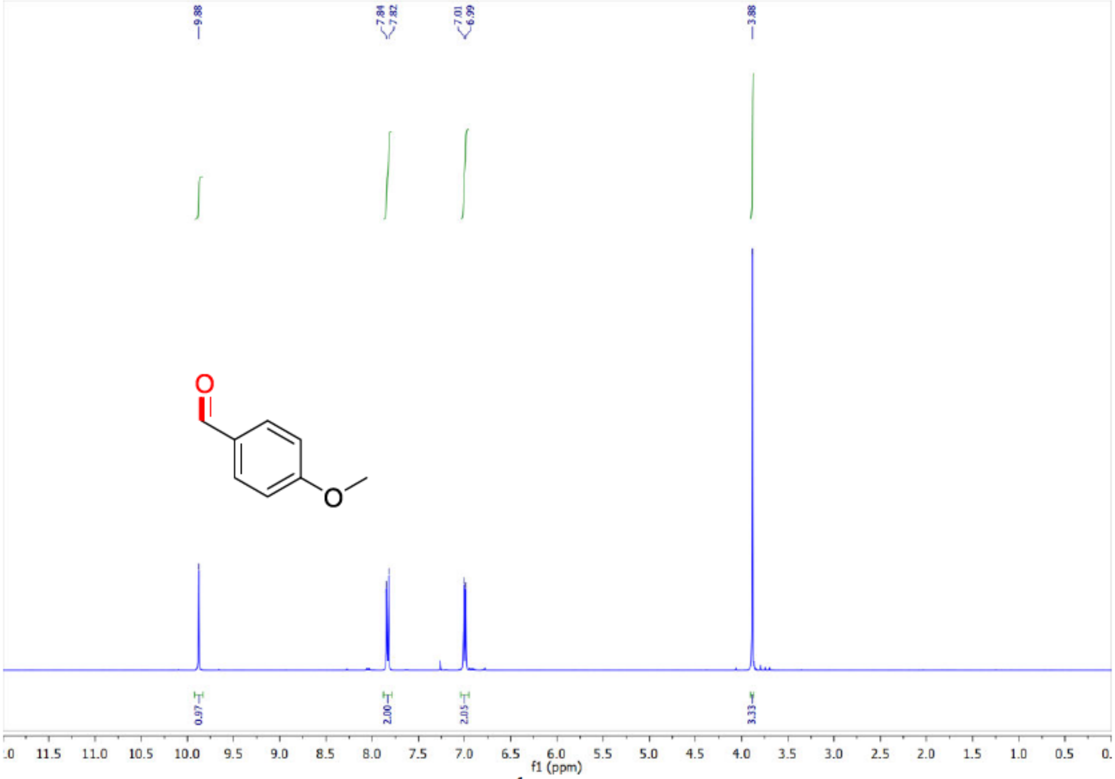

Supplementary Figure 107. ^{1}H NMR spectra of compound of **6e**

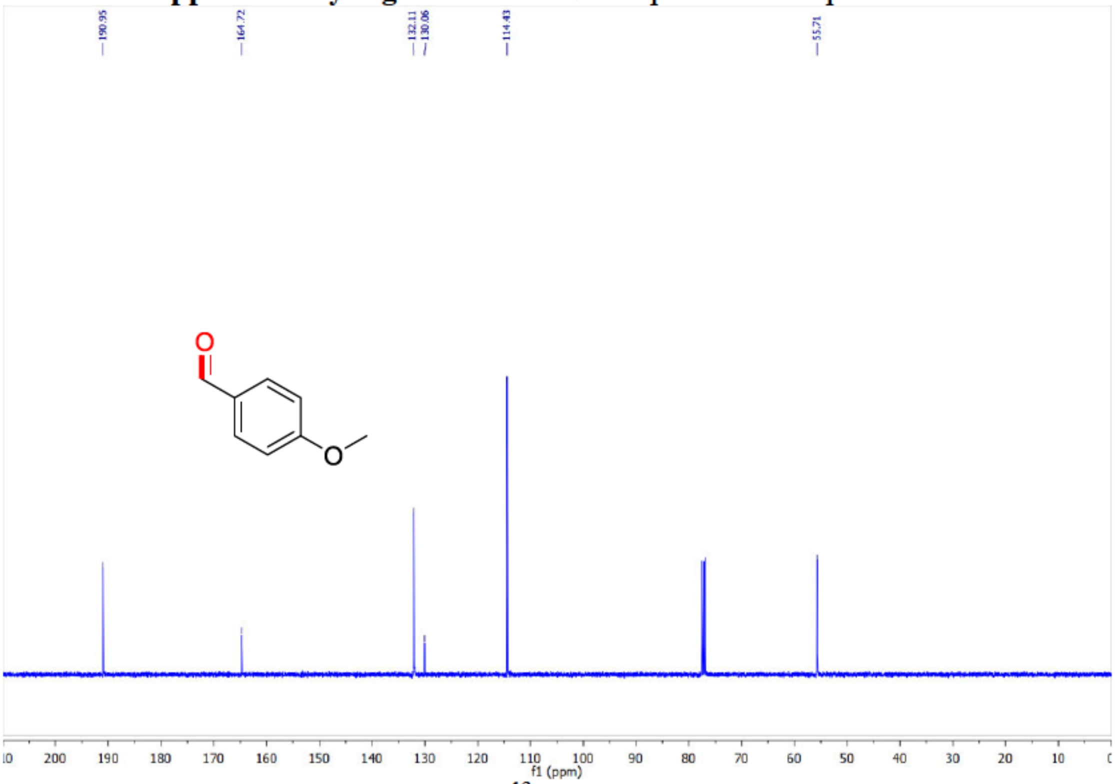

Supplementary Figure 108. ^{13}C NMR spectra of compound of **6e**

8

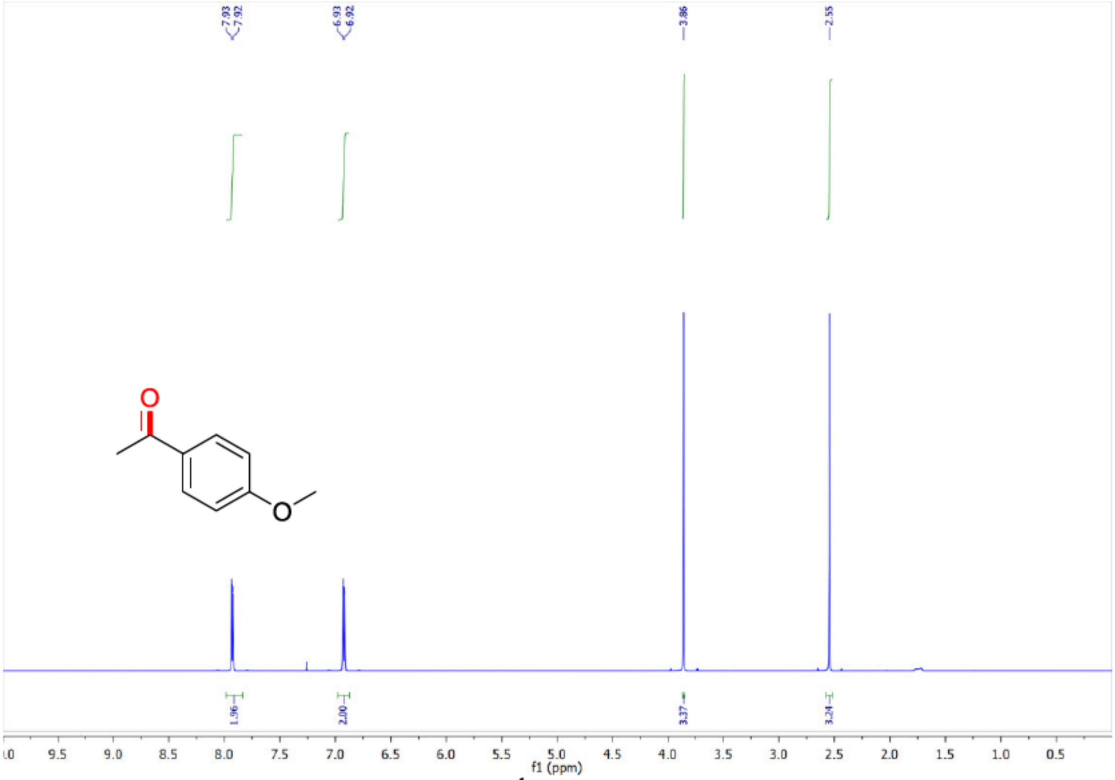

Supplementary Figure 109. ^{1}H NMR spectra of compound of **6f**

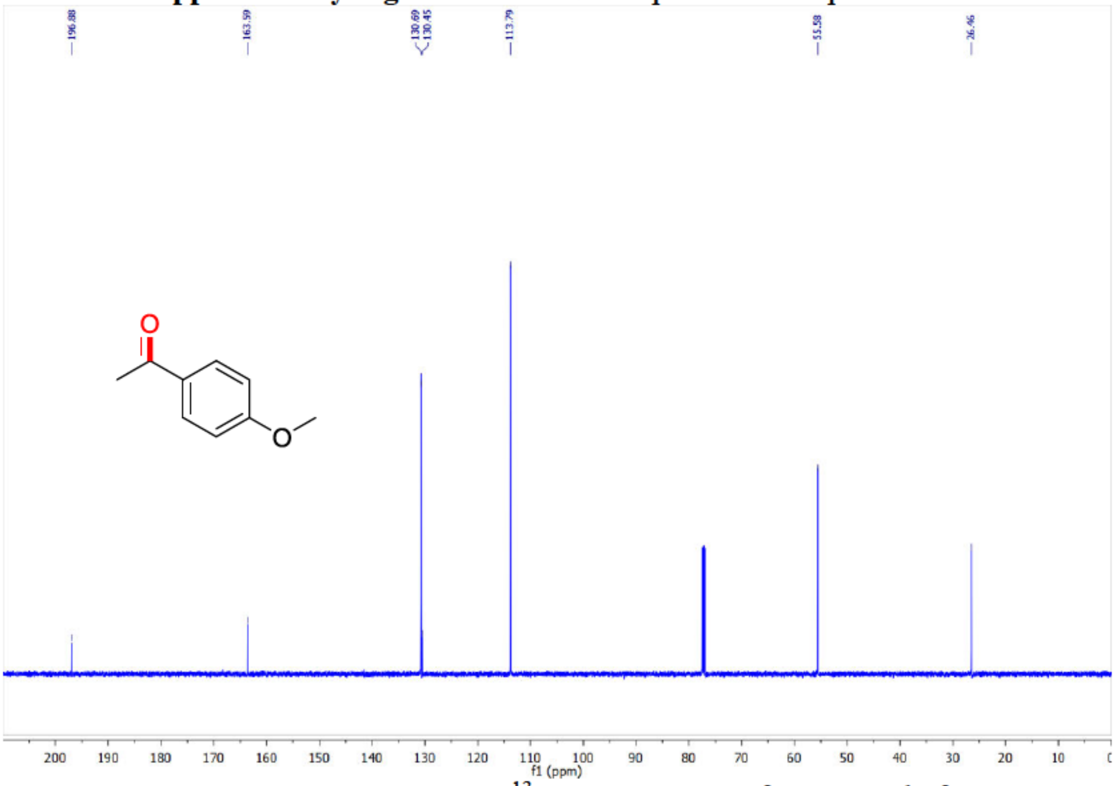

Supplementary Figure 110. ^{13}C NMR spectra of compound of **6f**

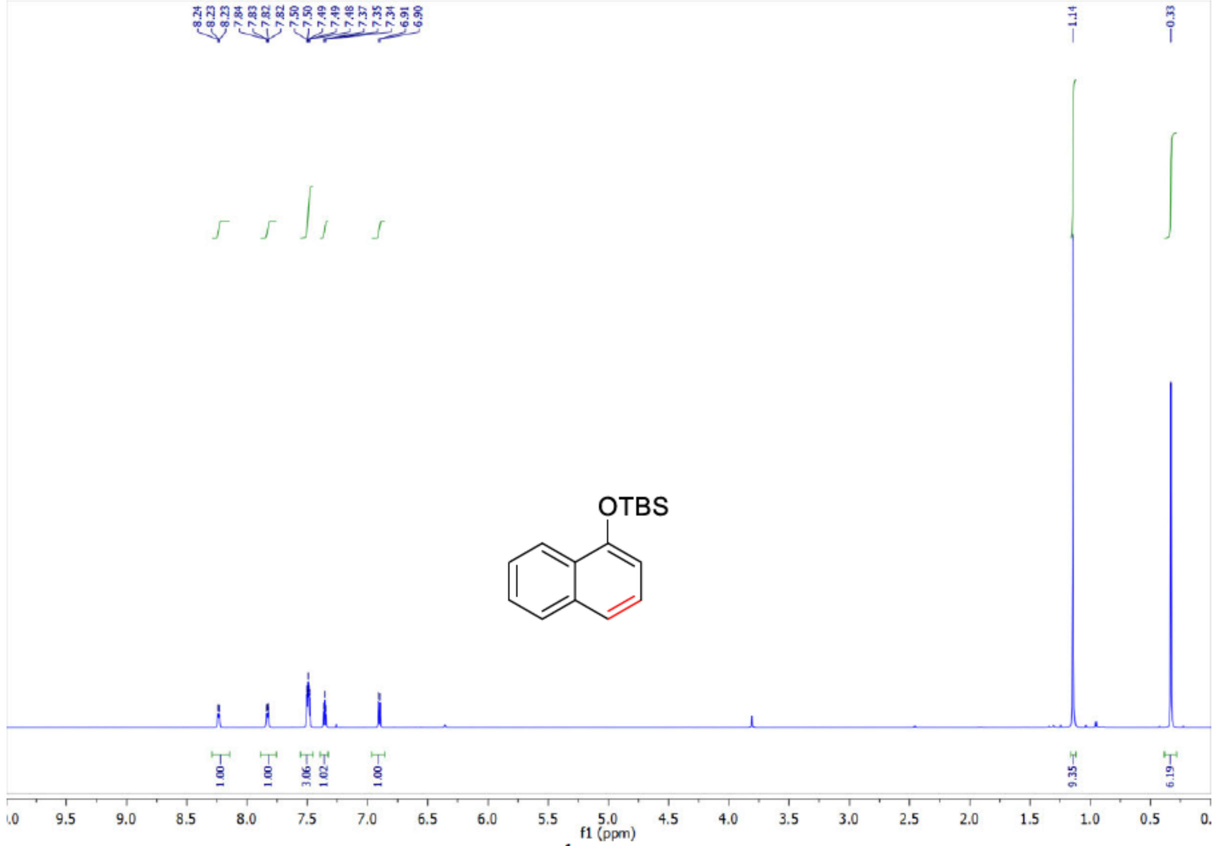

Supplementary Figure 111. ^{1}H NMR spectra of compound of **6g**

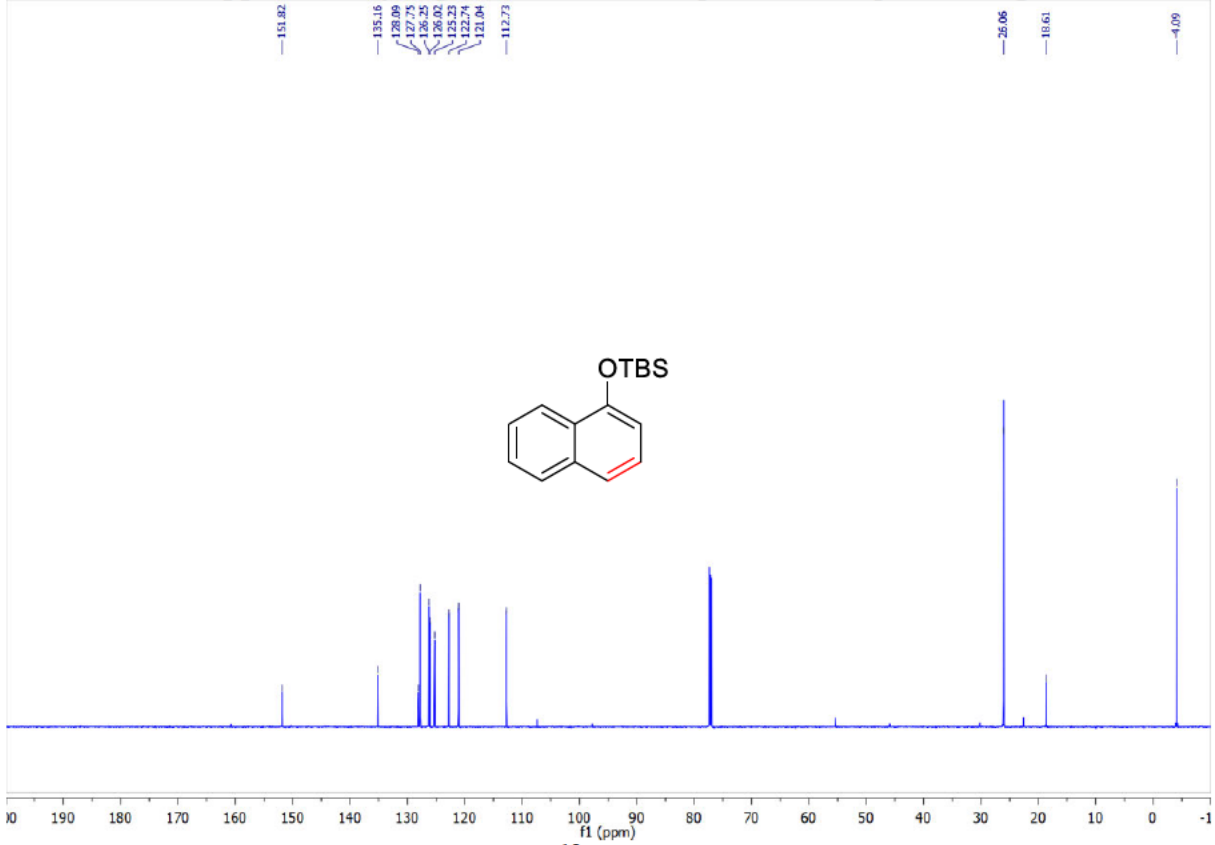

Supplementary Figure 112. ^{13}C NMR spectra of compound of **6g**

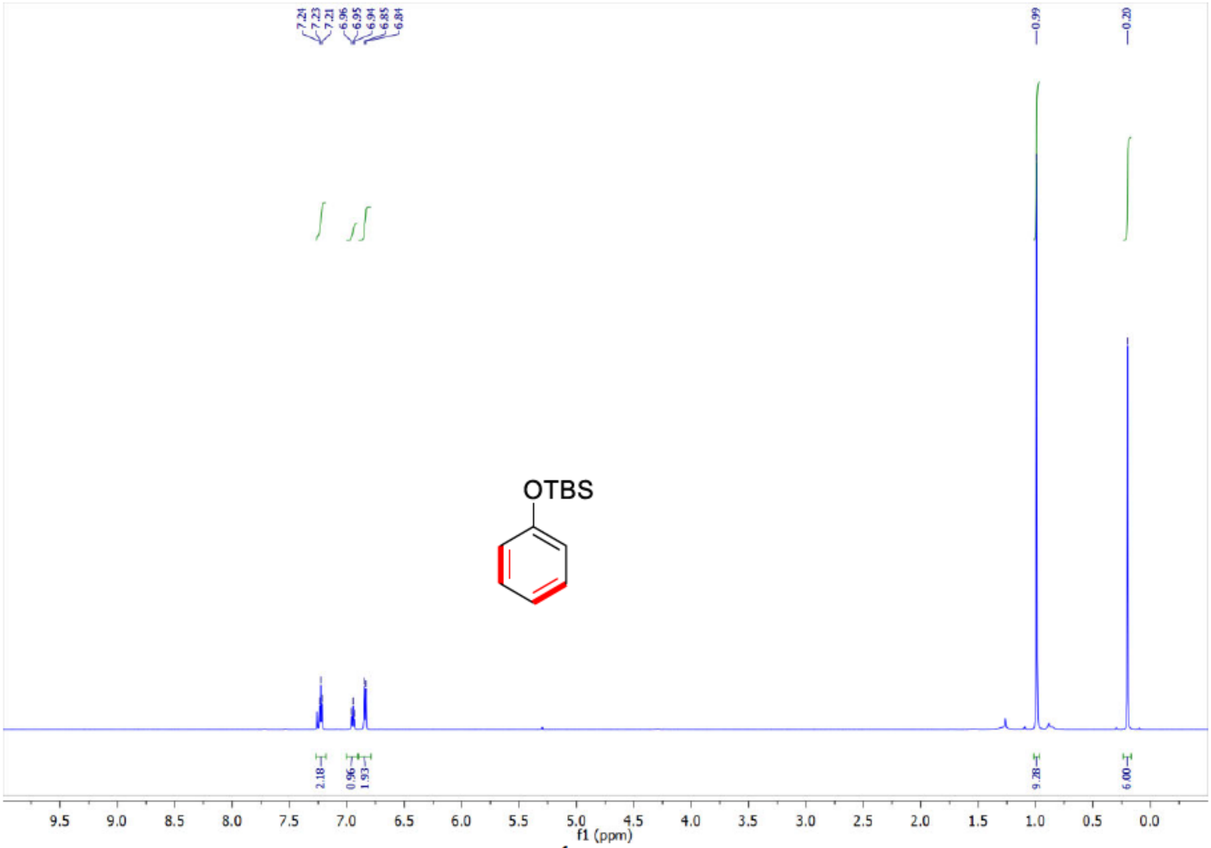

Supplementary Figure 113. ^{1}H NMR spectra of compound of **6h**

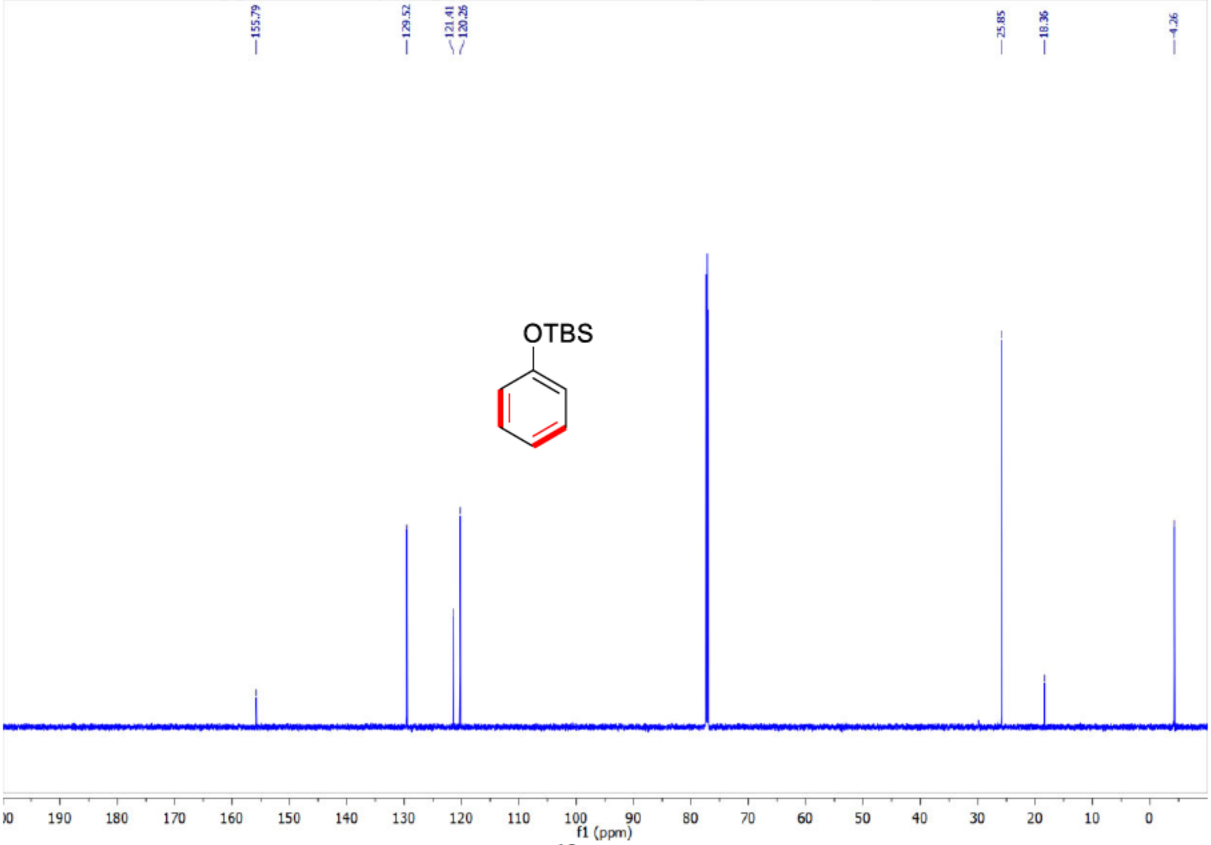

Supplementary Figure 114. ^{13}C NMR spectra of compound of **6h**

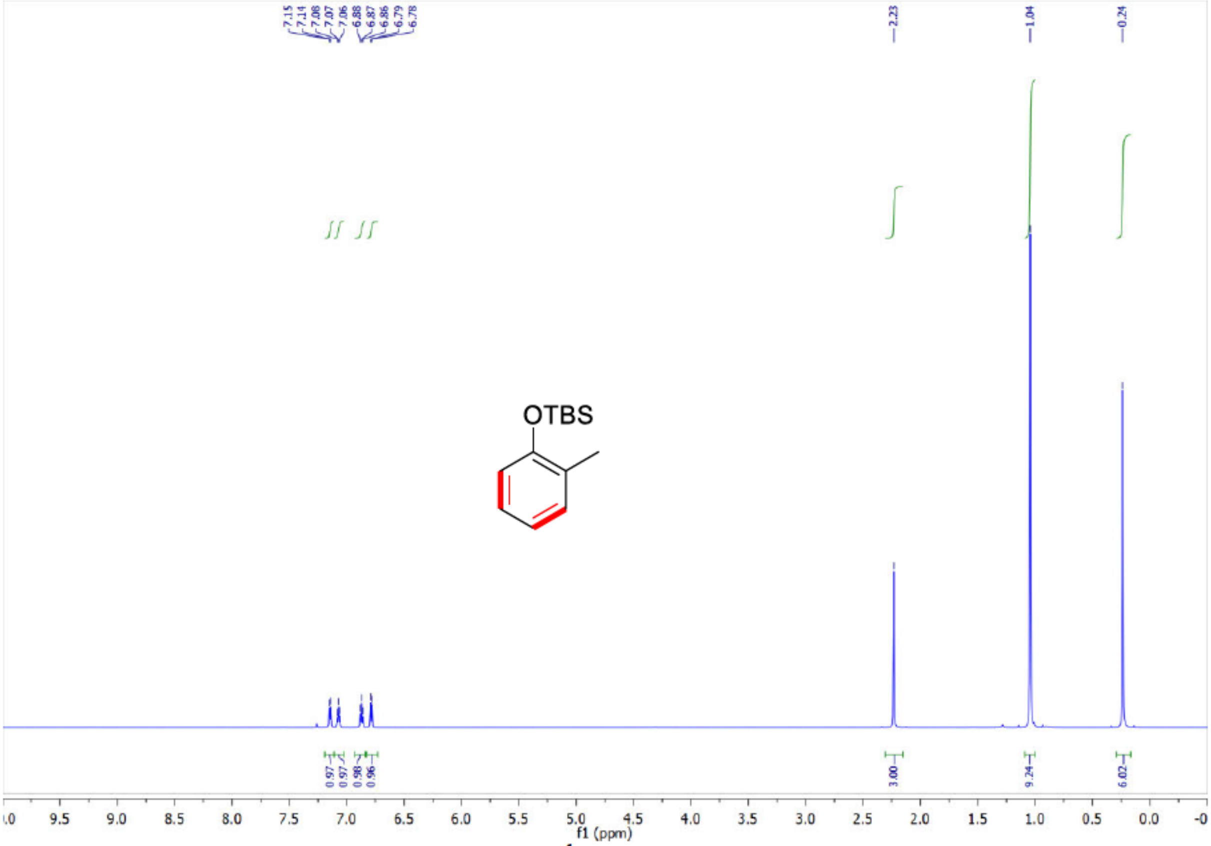

Supplementary Figure 115. ^{1}H NMR spectra of compound of **6i**

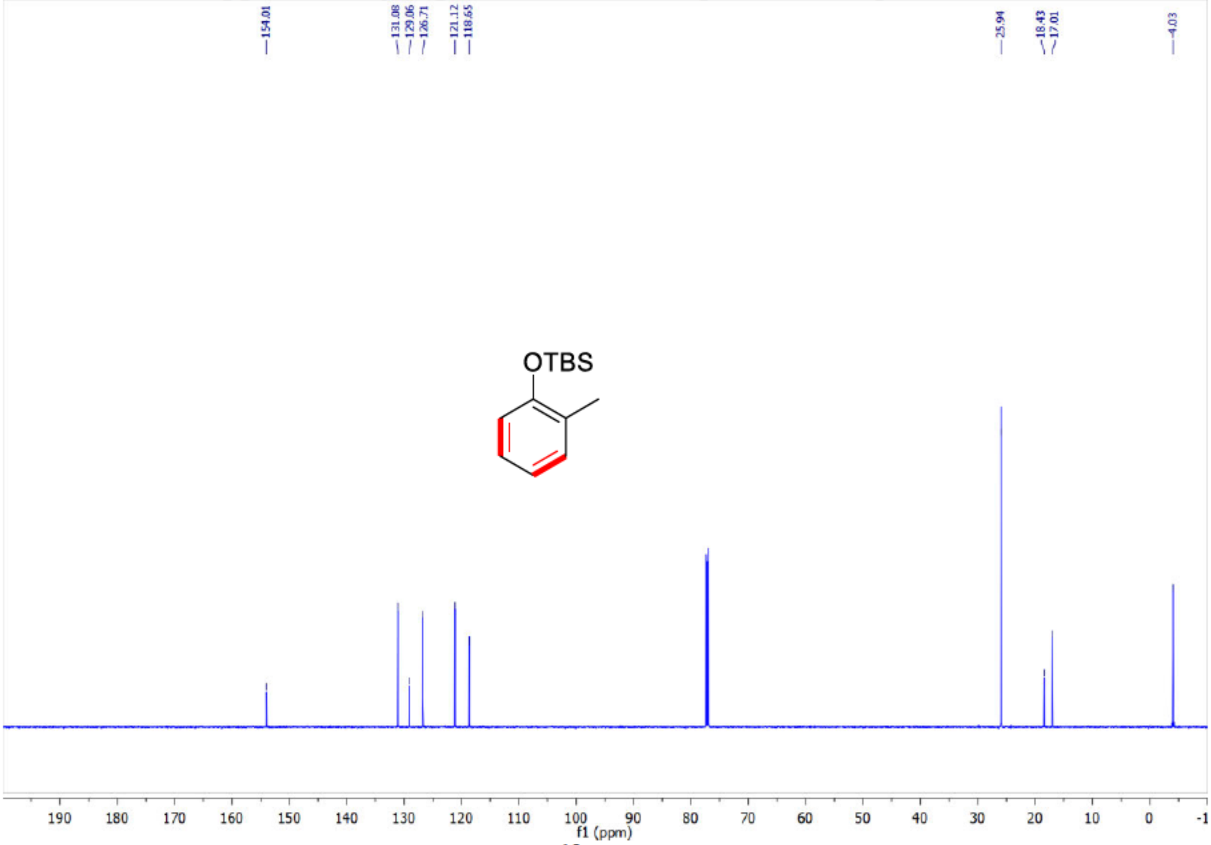

Supplementary Figure 116. ^{13}C NMR spectra of compound of **6i**

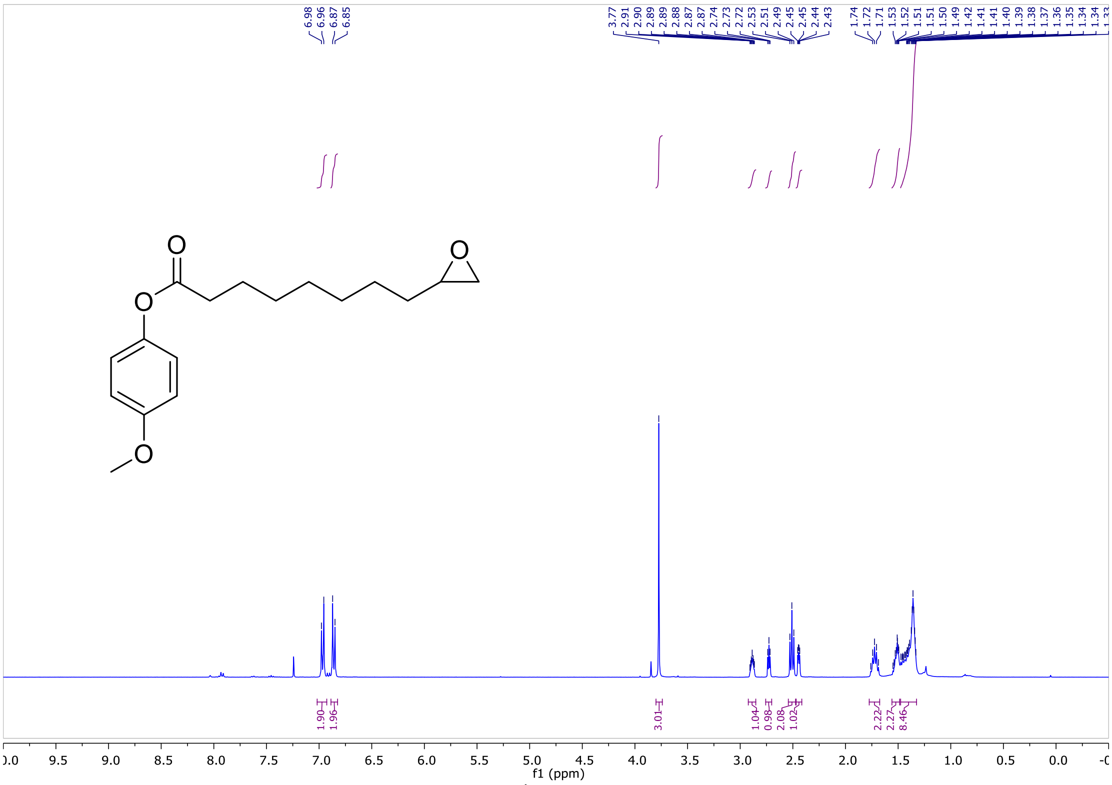

Supplementary Figure 117. ^{1}H NMR spectra of compound of **7**

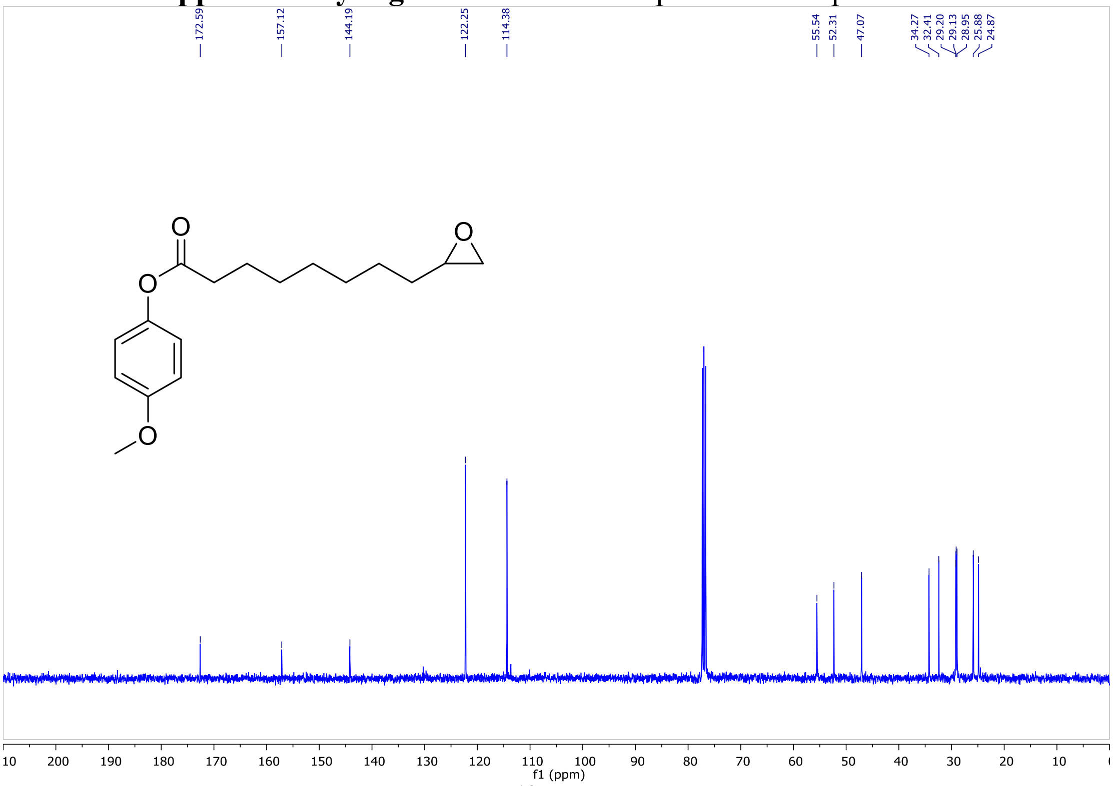

Supplementary Figure 118. ^{13}C NMR spectra of compound of **7**

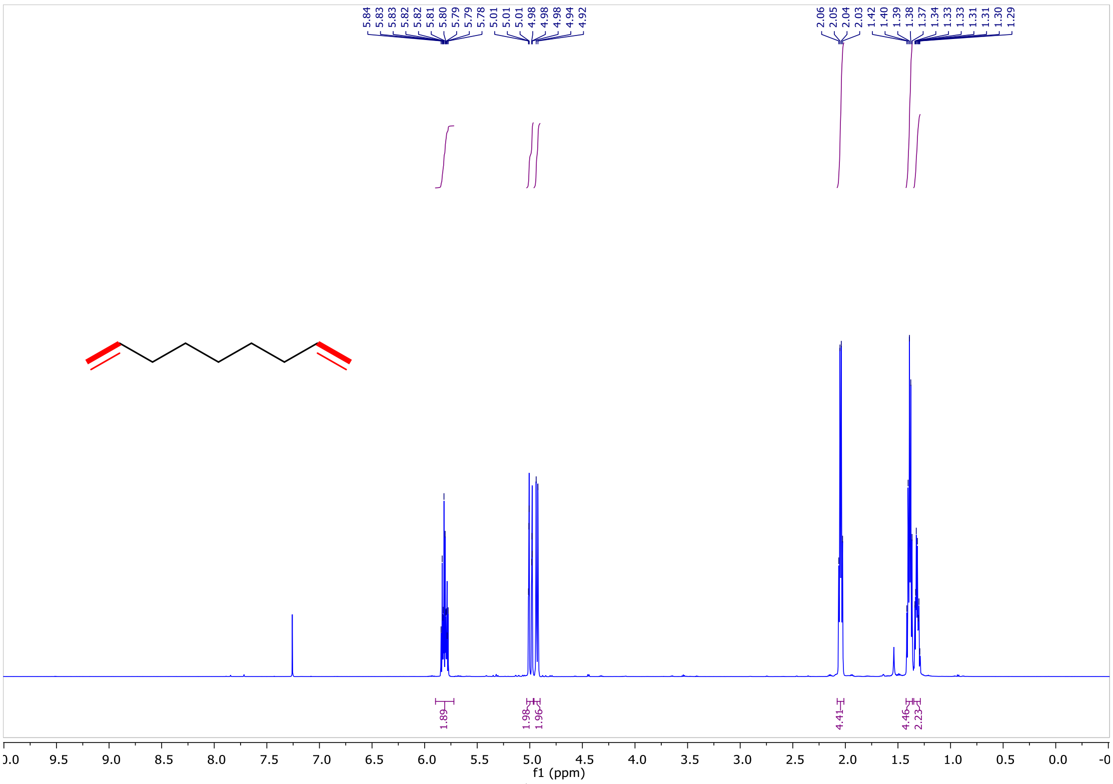

Supplementary Figure 119. ^{1}H NMR spectra of compound of **9**

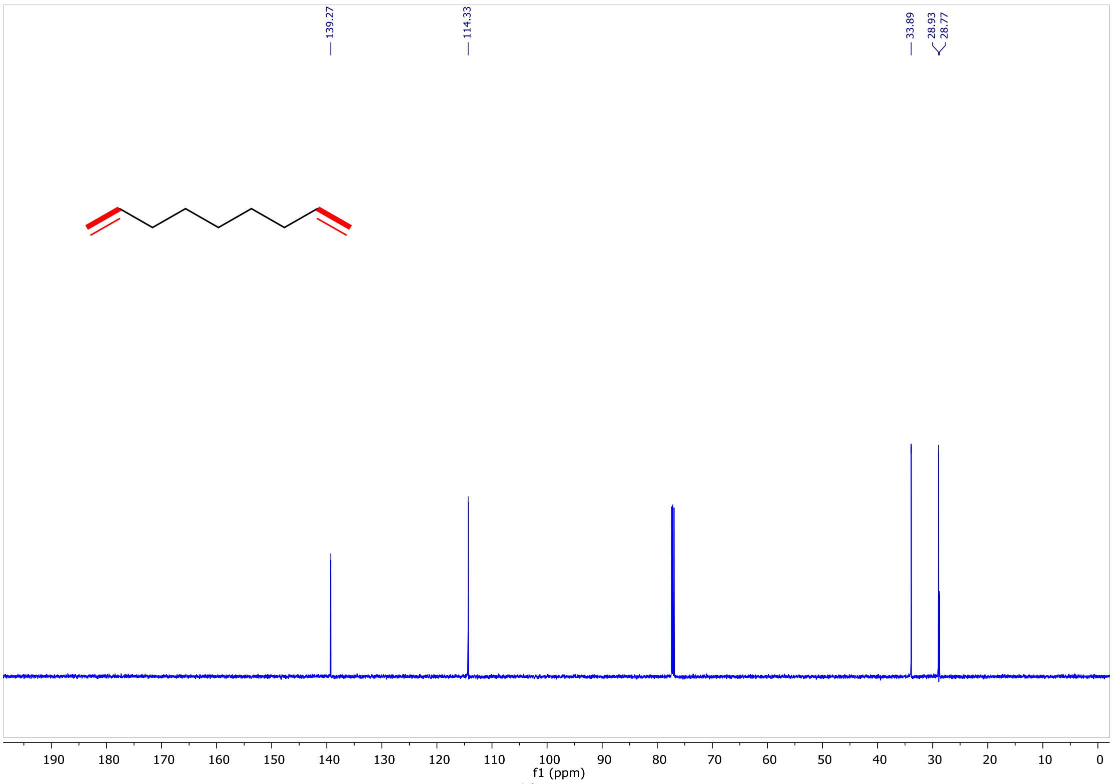

Supplementary Figure 120. ^{13}C NMR spectra of compound of **9**

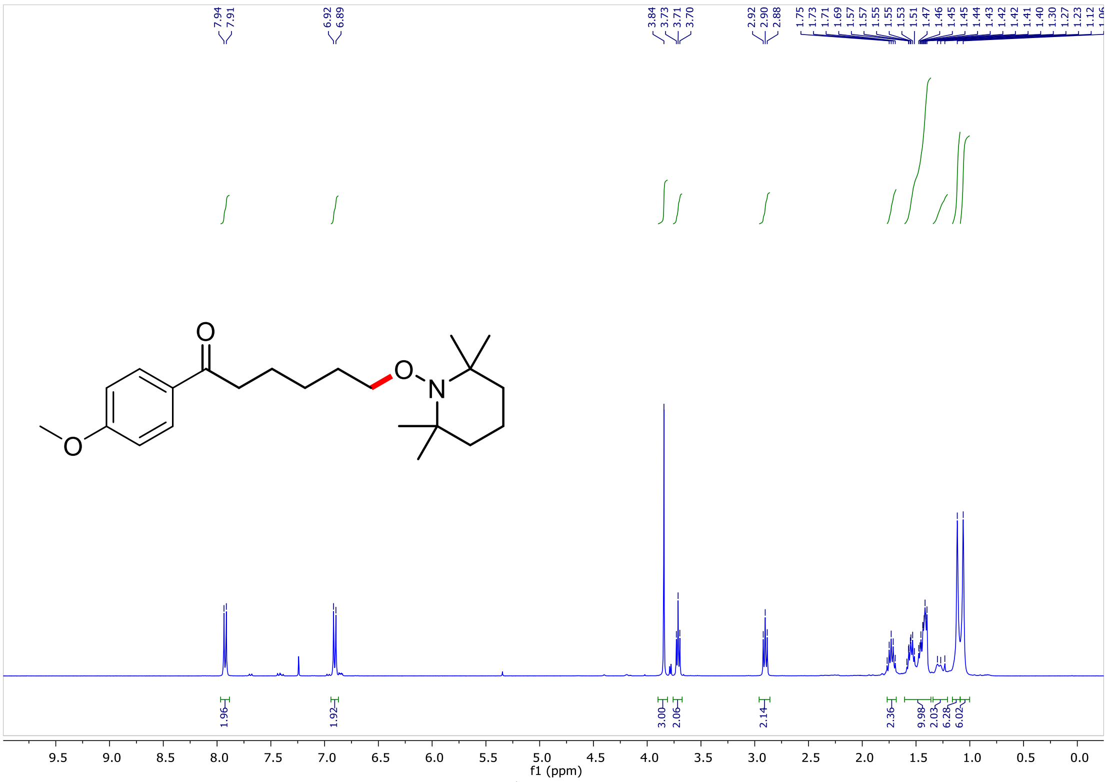

Supplementary Figure 121. ^{1}H NMR spectra of compound of **10**

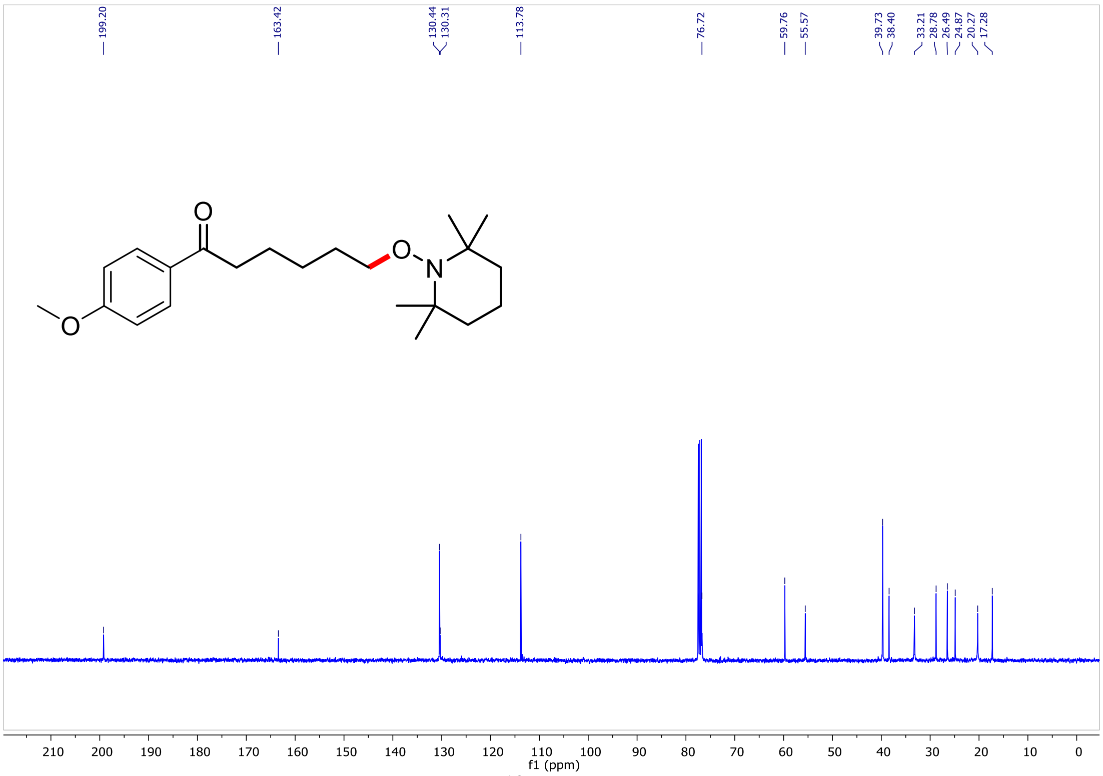

Supplementary Figure 122. ^{13}C NMR spectra of compound of **10**

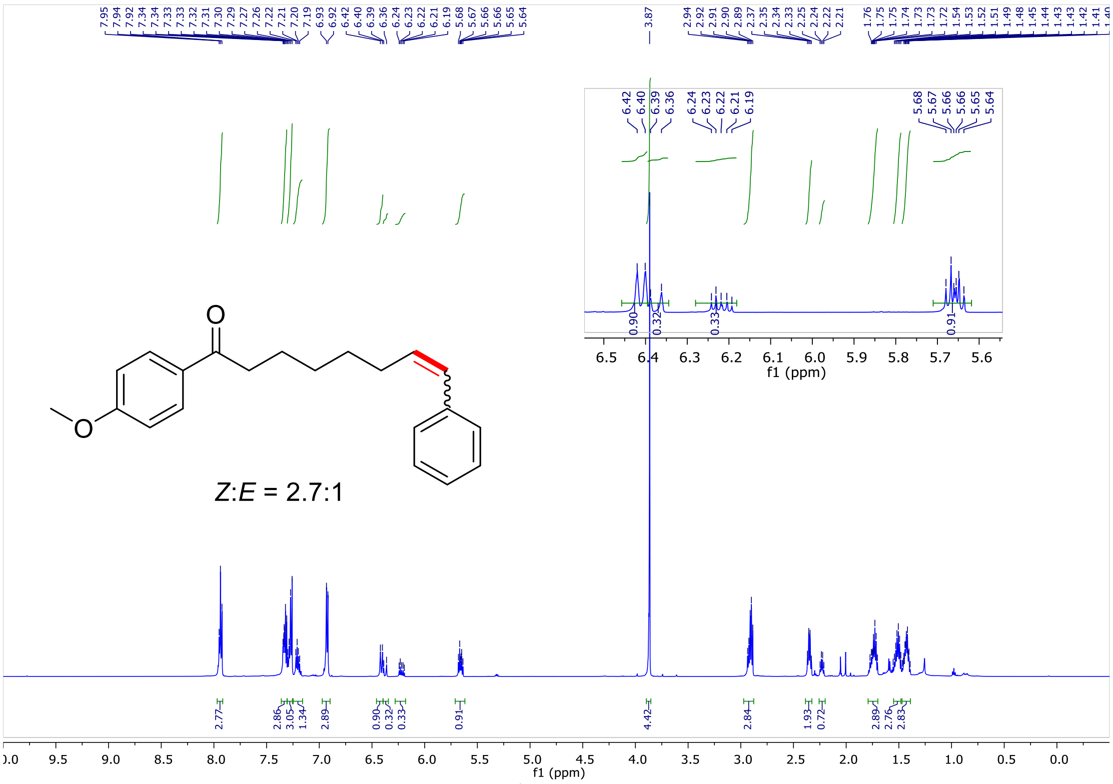

Supplementary Figure 123. ^{1}H NMR spectra of compound of **11**

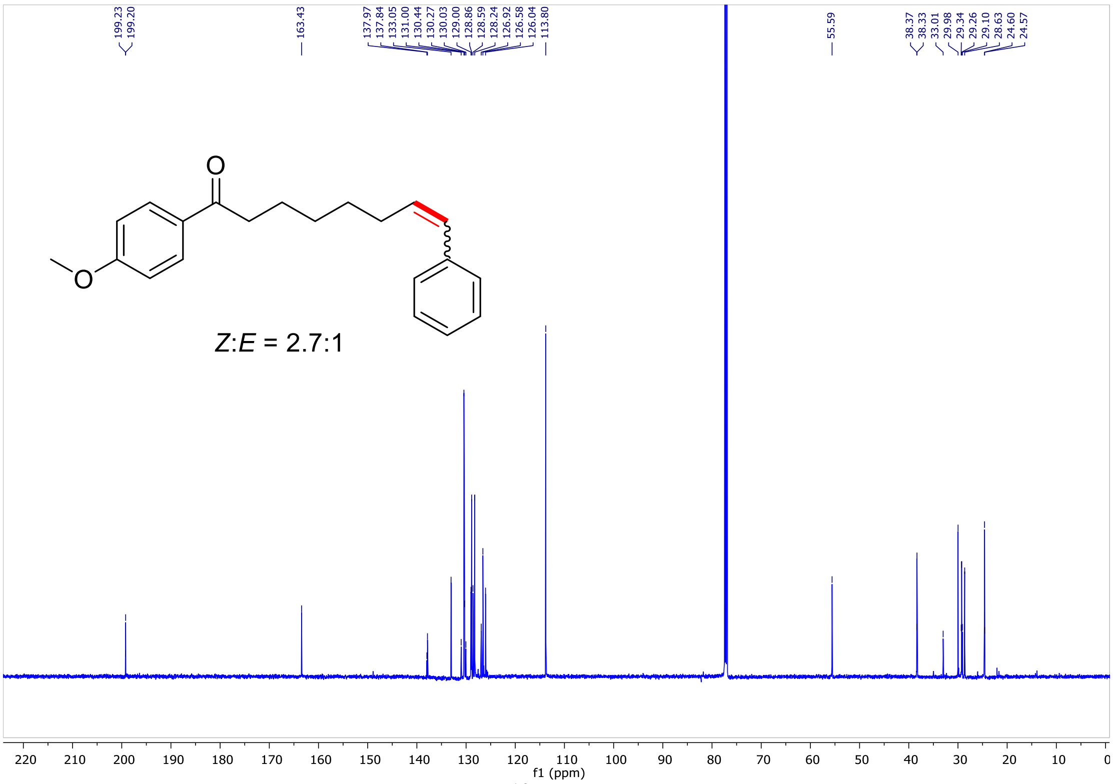

Supplementary Figure 124. ^{13}C NMR spectra of compound of **11**

Supplementary References

1 Yayla, H. G., Wang, H., Tarantino, K. T., Orbe, H. S. & Knowles, R. R. Catalytic Ring-Opening of Cyclic Alcohols Enabled by PCET Activation of Strong O–H Bonds. *J. Am. Chem. Soc.* **138**, 10794-10797 (2016).

2 Huang, L., Ji, T. & Rueping, M. Remote Nickel-Catalyzed Cross-Coupling Arylation via Proton-Coupled Electron Transfer-Enabled C–C Bond Cleavage. *J. Am. Chem. Soc.* **142**, 3532-3539 (2020).

3 Simonin, C. *et al.* Optimization of TRPV6 Calcium Channel Inhibitors Using a 3D Ligand-Based Virtual Screening Method. *Angew. Chem. Int. Ed.* **54**, 14748-14752 (2015).

4 Croft, R. A. *et al.* Catalytic Friedel-Crafts Reactions on Saturated Heterocycles and Small Rings for sp3-sp2 Coupling of Medicinally Relevant Fragments. *Eur. J. Org. Chem.* **2019**, 5385-5395 (2019).

5 Wang, J., Huang, B., Shi, C., Yang, C. & Xia, W. Visible-Light-Mediated Ring-Opening Strategy for the Regiospecific Allylation/Formylation of Cycloalkanols. *J. Org. Chem.* **83**, 9696-9706 (2018).

6 Hamilton, D. S. & Nicewicz, D. A. Direct Catalytic Anti-Markovnikov Hydroetherification of Alkenols. *J. Am. Chem. Soc.* **134**, 18577-18580 (2012).

7 Zhang, Z. *et al.* Oxidative α-C–C Bond Cleavage of 2° and 3° Alcohols to Aromatic Acids with O2 at Room Temperature via Iron Photocatalysis. *Org. Lett.* **23**, 2915-2920 (2021).

8 Rono, L. J., Yayla, H. G., Wang, D. Y., Armstrong, M. F. & Knowles, R. R. Enantioselective Photoredox Catalysis Enabled by Proton-Coupled Electron Transfer: Development of an Asymmetric Aza-Pinacol Cyclization. *J. Am. Chem. Soc.* **135**, 17735-17738 (2013).

9 Eberle, C. *et al.* Improved Inhibitors of Trypanothione Reductase by Combination of Motifs: Synthesis, Inhibitory Potency, Binding Mode, and Antiprotozoal Activities. *ChemMedChem* **6**, 292-301 (2011).

10 Larsen, C. R. & Grotjahn, D. B. Stereoselective Alkene Isomerization over One Position. *J. Am. Chem. Soc.* **134**, 10357-10360 (2012).

11 Chepiga, K. M., Feng, Y., Brunelli, N. A., Jones, C. W. & Davies, H. M. L. Silica-Immobilized Chiral Dirhodium(II) Catalyst for Enantioselective Carbenoid Reactions. *Org. Lett.* **15**, 6136-6139 (2013).

12 Zhang, J., Wang, L., Liu, Q., Yang, Z. & Huang, Y. Synthesis of α,β-unsaturated carbonyl compounds via a visible-light-promoted organocatalytic aerobic oxidation. *Chem. Commun.* **49**, 11662-11664 (2013).

13 Sarabèr, F. C. E., Baranovsky, A., Jansen, B. J. M., Posthumus, M. A. & de Groot, A. New approaches toward the synthesis of (D-homo) steroid skeletons using Mukaiyama reactions. *Tetrahedron* **62**, 1726-1742 (2006).

14 Schmidt, B. & Geißler, D. Ru- and Pd-Catalysed Synthesis of 2-Arylfurans by One-Flask Heck Arylation/Oxidation. *Eur. J. Org. Chem.* **2011**, 4814-4822 (2011).

15 Davies, J., Booth, S. G., Essafi, S., Dryfe, R. A. W. & Leonori, D. Visible-Light-Mediated Generation of Nitrogen-Centered Radicals: Metal-Free Hydroimination and Iminohydroxylation Cyclization Reactions. *Angew. Chem. Int. Ed.* **54**, 14017-14021 (2015).

16 Tarantino, K. T., Liu, P. & Knowles, R. R. Catalytic Ketyl-Olefin Cyclizations Enabled by Proton-Coupled Electron Transfer. *J. Am. Chem. Soc.* **135**, 10022-10025 (2013).

17 Enholm, E. J. & Jia, Z. J. One- and Two-Electron Reactions from the Rearrangement of α-Ketocyclopropanes by O-Stannyl Ketyls. *J. Org. Chem.* **62**, 9159-9164 (1997).

18 Ouvry, G. & Zard, S. Z. New Radical HomoallylationReactions. *Synlett* **2003**, 1627-1630 (2003).

19 Marwah, P., Marwah, A., Lardy, H. A., Miyamoto, H. & Chang, C. C19-Steroids as androgen receptor modulators: Design, discovery, and structure-activity relationship of new steroidal androgen receptor antagonists. *Bioorg. Med. Chem.* **14**, 5933-5947 (2006).

20 Tlahuext-Aca, A., Candish, L., Garza-Sanchez, R. A. & Glorius, F. Decarboxylative Olefination of Activated Aliphatic Acids Enabled by Dual Organophotoredox/Copper Catalysis. *ACS Catal.* **8**, 1715-1719 (2018).

21 Chapuis, C. & Brauchli, R. Preparation of Campholenal Analogues: Chirons for the lipophilic moiety of sandalwood-like odorant alcohols. *Helv. Chim. Acta* **75**, 1527-1546 (1992).

22 Lei, C., Yip, Y. J. & Zhou, J. S. Nickel-Catalyzed Direct Synthesis of Aryl Olefins from Ketones and Organoboron Reagents under Neutral Conditions. *J. Am. Chem. Soc.* **139**, 6086-6089 (2017).

23 Reed, N. L., Lutovsky, G. A. & Yoon, T. P. Copper-Mediated Radical–Polar Crossover Enables Photocatalytic Oxidative Functionalization of Sterically Bulky Alkenes. *J. Am. Chem. Soc.* **143**, 6065-6070 (2021).

24 Harris, M. R., Konev, M. O. & Jarvo, E. R. Enantiospecific Intramolecular Heck Reactions of Secondary Benzylic Ethers. *J. Am. Chem. Soc.* **136**, 7825-7828 (2014).

25 Yue, H., Guo, L., Lee, S.-C., Liu, X. & Rueping, M. Selective Reductive Removal of Ester and Amide Groups from Arenes and Heteroarenes through Nickel-Catalyzed C−O and C−N Bond Activation. *Angew. Chem. Int. Ed.* **56**, 3972-3976 (2017).

26 Álvarez-Bercedo, P. & Martin, R. Ni-Catalyzed Reduction of Inert C−O Bonds: A New Strategy for Using Aryl Ethers as Easily Removable Directing Groups. *J. Am. Chem. Soc.* **132**, 17352-17353 (2010).

27 Kato, S. *et al.* Hybrid Catalysis Enabling Room-Temperature Hydrogen Gas Release from N-Heterocycles and Tetrahydronaphthalenes. *J. Am. Chem. Soc.* **139**, 2204-2207 (2017).

28 Hoover, J. M. & Stahl, S. S. Highly Practical Copper(I)/TEMPO Catalyst System for Chemoselective Aerobic Oxidation of Primary Alcohols. *J. Am. Chem. Soc.* **133**, 16901-16910 (2011).

29 Sun, R., Qin, Y. & Nocera, D. G. General Paradigm in Photoredox Nickel-Catalyzed Cross-Coupling Allows for Light-Free Access to Reactivity. *Angew. Chem. Int. Ed.* **59**, 9527-9533 (2020).

30 Wiensch, E. M., Todd, D. P. & Montgomery, J. Silyloxyarenes as Versatile Coupling Substrates Enabled by Nickel-Catalyzed C–O Bond Cleavage. *ACS Catal.* **7**, 5568-5571 (2017).

31 Gabrielli, L. & Mancin, F. Minimal Self-Immolative Probe for Multimodal Fluoride Detection. *J. Org. Chem.* **81**, 10715-10720 (2016).

32 Zhang, Q. & Takacs, J. M. Click-Connected Ligand Scaffolds: Macrocyclic Chelates for Asymmetric Hydrogenation. *Org. Lett.* **10**, 545-548 (2008).

33 Dennig, A. *et al.* Enzymatic Oxidative Tandem Decarboxylation of Dioic Acids to Terminal Dienes. *Eur. J. Org. Chem.* **2016**, 3473-3477 (2016).
